# Stereodivergent Synthesis of 1,4-Dicarbonyl Compounds through Sulfonium Rearrangement: Mechanistic Investigation, Stereocontrolled Access to γ-Lactones and γ-Lactams, and Total Synthesis of Paraconic Acids

**DOI:** 10.1021/jacs.4c01755

**Published:** 2024-05-13

**Authors:** Nicolas G.-Simonian, Philipp Spieß, Margaux Riomet, Boris Maryasin, Immo Klose, Alexander Beaton Garcia, Laurin Pollesböck, Dainis Kaldre, Uroš Todorovic, Julia Minghua Liu, Daniel Kaiser, Leticia González, Nuno Maulide

**Affiliations:** † Institute of Organic Chemistry, University of Vienna, Währinger Straße 38, 1090 Vienna, Austria; ‡ Institute of Theoretical Chemistry, University of Vienna, Währinger Straße 17, 1090 Vienna, Austria

## Abstract

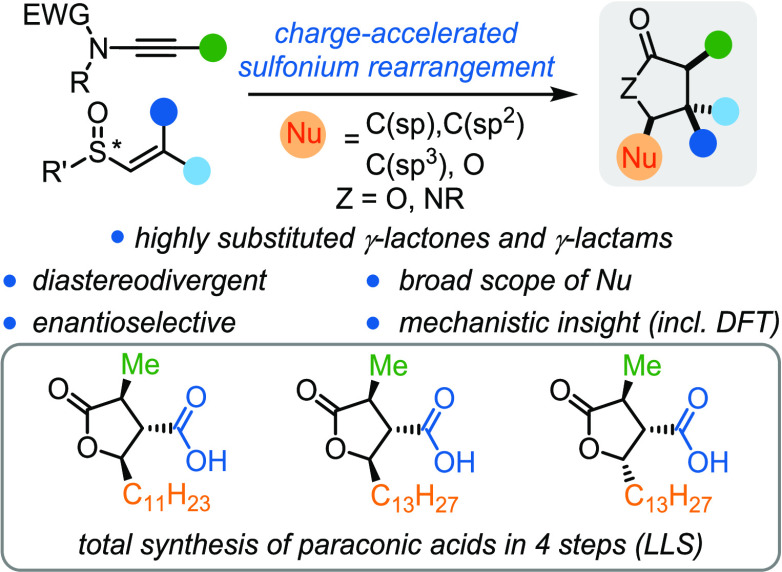

Although simple γ-lactones and γ-lactams have received considerable attention from the synthetic community, particularly due to their relevance in biological and medicinal contexts, stereoselective synthetic approaches to more densely substituted derivatives remain scarce. The in-depth study presented herein, showcasing a straightforward method for the stereocontrolled synthesis of γ-lactones and γ-lactams, builds on and considerably expands the stereodivergent synthesis of 1,4-dicarbonyl compounds by a ynamide/vinyl sulfoxide coupling. A full mechanistic and computational study of the rearrangement was conducted, uncovering the role of all of the reaction components and providing a rationale for stereoselection. The broad applicability of the developed tools to streamlining synthesis is demonstrated by concise enantioselective total syntheses of (+)-nephrosteranic acid, (+)-rocellaric acid, and (+)-nephromopsinic acid.

## Introduction

γ-Lactones and γ-lactams are common structural motifs in bioactive compounds, including molecules with anticancer, antiviral, or antibiotic activities, but also in a number of fragrances.^1−6^ While they have raised significant interest from the synthetic community, the stereodivergent synthesis of highly substituted γ-lactones and γ-lactams still constitutes a formidable challenge.^7−15^

**Figure 1:**
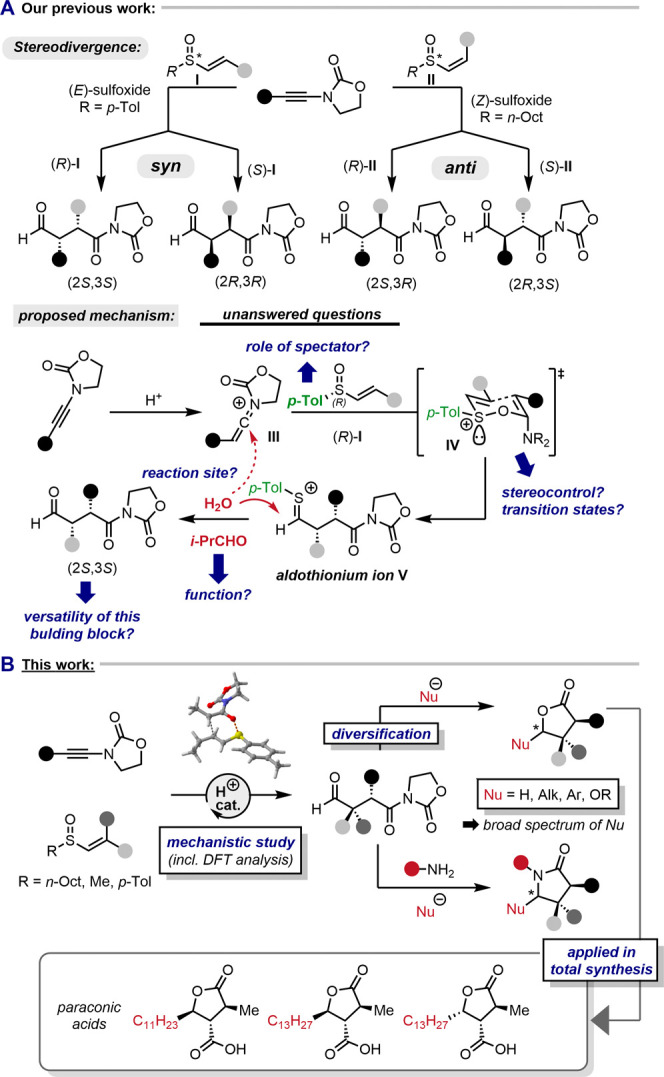
Previous work and goals. (A) Diastereodivergent access to 1,4-dicarbonyl compounds via charge-accelerated [3,3]-sigmatropic sulfonium rearrangement. (B) Our approach toward the synthesis of highly substituted γ-lactones and γ-lactams. *p*-Tol = *para*-tolyl; DFT = density functional theory.

In 2018, our group disclosed a strategy for the synthesis of α,β-disubstituted 1,4-dicarbonyl compounds, allowing highly stereoselective access to all four possible isomers (Figure 1A, *top*).^16,17^ Mechanistically, we proposed initiation by forming keteniminium ion **III** through protonation of the ynamide reactant, followed by the addition of vinyl sulfoxide **I**. This would generate an adduct (**IV**) that undergoes a key charge-accelerated [3,3]-sigmatropic sulfonium rearrangement, ultimately yielding the corresponding aldothionium ion intermediate **V** in a stereoselective fashion.^18−27^ This resulting species is hydrolyzed in situ to afford a 1,4-dicarbonyl compound.

Experimentally, the enantio- and diastereoselectivities of the process are determined exclusively by the vinyl sulfoxide partner ((*S*)- and (*R*)-**I** and **II** (Figure 1A, *top*)). Remarkably, the geometry of the double bond of the vinyl sulfoxide determines the relative configuration of the C2–C3 substituents, with (*E*)-geometry providing the *syn*-configured product, while the (*Z*)-olefin yields the *anti*-configured product. At the same time, the chiral information at the stereogenic sulfur center governs the enantioselectivity of the process. This sets the absolute configuration of the products and allows, as a crowning corollary, the efficient construction of all-carbon quaternary centers.

However, several aspects of this selective and stereodivergent process remain poorly understood. On the one hand, the precise role of the two crucial additives, *iso-*butyraldehyde and water, which drastically improved yields and, to a lesser extent, stereoselectivity, was not elucidated. In particular, it appeared paradoxical that water could benefit a transformation in which a keteniminium ion is involved. On the other hand, the dramatically different performances of different vinyl sulfoxides remained unexplained. Particularly, it was found that the nature of the nonreacting (“spectator”) sulfur substituent was crucial for the performance of (*E*)-vinyl sulfoxides and (*Z*)-vinyl sulfoxides: a *p*-tolyl substituent was found to be optimal for (*E*)-vinyl sulfoxides, while an aliphatic (*n*-octyl) substituent was determined to be crucial for (*Z*)-vinyl sulfoxides.

Furthermore, from a synthetic perspective, a significant potential application of 1,4-dicarbonyls is their ability to serve as precursors for γ-lactones and γ-lactams upon treatment with nucleophiles. We thus anticipated that the diastereoselective addition of a range of nucleophiles to the 1,4-dicarbonyl could yield highly substituted and valuable products (Figure 1B).

In this study, we present in-depth investigations and a better understanding of the mechanism of this transformation, aided by experimental and computational studies. We furthermore describe our efforts to rationalize the origin of stereoselectivity of this method as well as its use for the synthesis of highly substituted γ-lactones and γ-lactams, culminating in short enantioselective total syntheses of three paraconic acids.

## Results and Discussion

**Figure 2:**
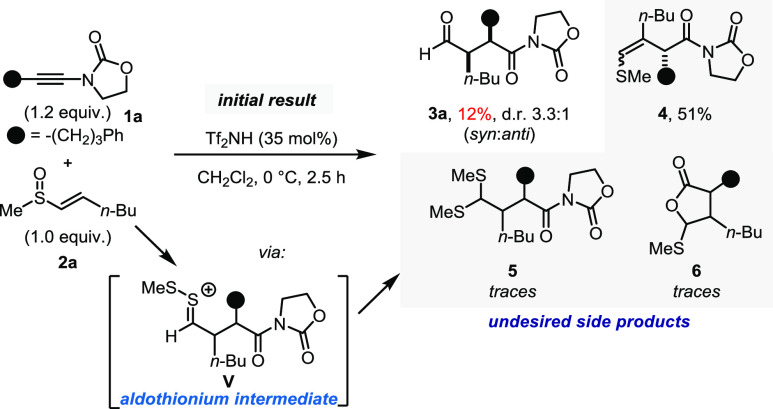
First discovery of the reaction. Tf = triflyl.

The reaction leading to 1,4-dicarbonyl compounds was first discovered by subjecting vinyl sulfoxide **2a** to acidic conditions [Tf_2_NH (35 mol %), CH_2_Cl_2_, 0 °C, 2.5 h] in the presence of ynamide **1a** (Figure 2). Initially, a mixture of products, containing 1,4-dicarbonyl compound **3a**, vinyl sulfide **4**, dithioketal **5**, and the cyclized γ-S lactone **6** was obtained (the latter two only in traces). Intriguingly, all of the observed species could be mechanistically traced back to the initial formation of a common aldothionium intermediate **V**.

**Figure 3:**
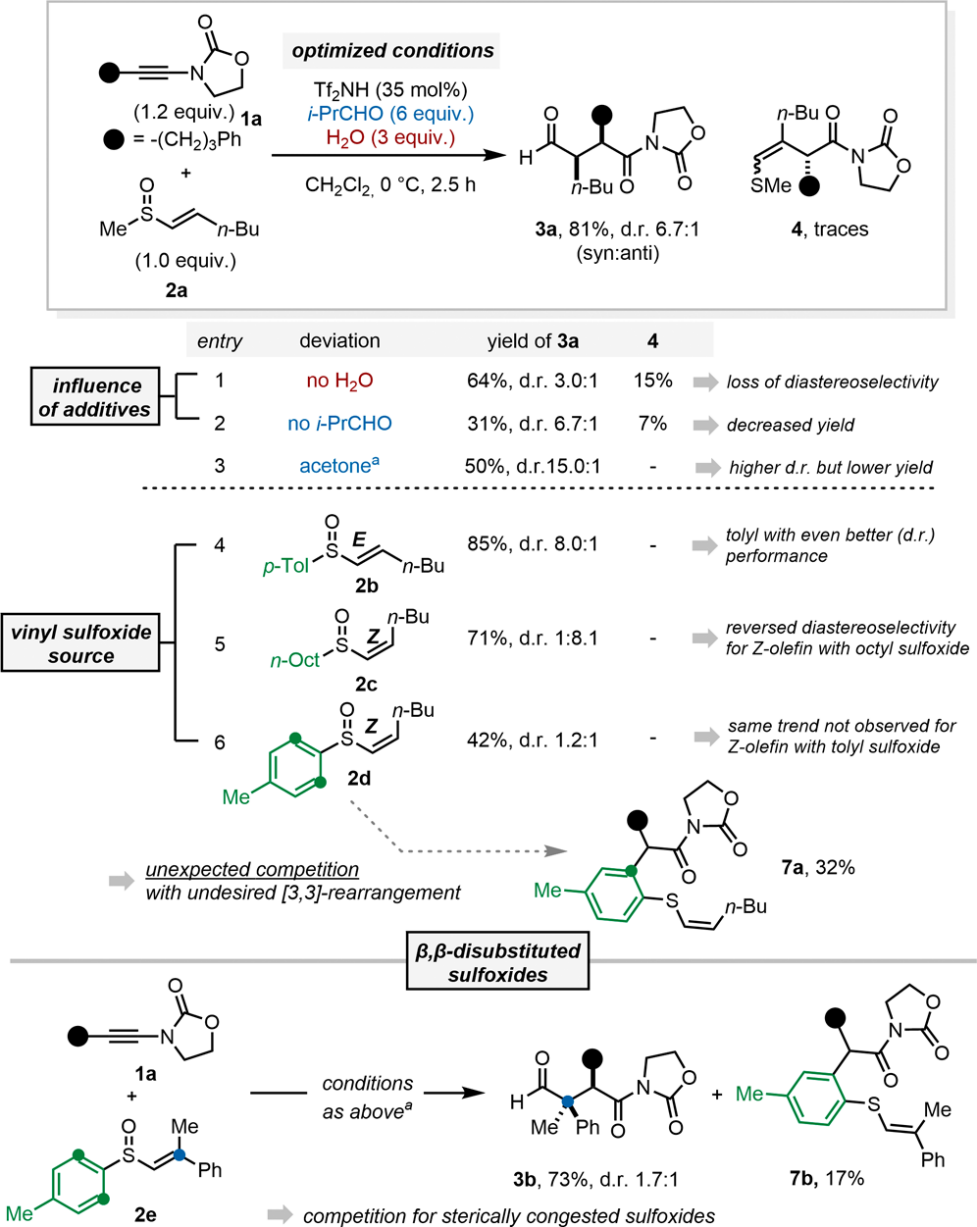
Optimization studies: probing the influence of additives and substitution. All yields were determined by ^1^H NMR analysis of the crude reaction mixture using mesitylene as an internal standard. d.r. = diastereoisomeric ratio; *p*-Tol = *para*-tolyl; and Tf = triflyl. ^a^50 mol % of Tf_2_NH + 2 equiv of **1a**.

In our attempts to funnel the aldothionium intermediate toward a single, enantio- and diastereomerically enriched product, we reasoned that the addition of reagents capable of promoting the in situ hydrolysis of **V** would be beneficial. After extensive optimization, we discovered that the key to reaching a high yield of the 1,4-dicarbonyl compound was the addition of water in combination with a sacrificial aldehyde (*i*-PrCHO) to the reaction mixture (Figure 3, top result).^28^

While the reaction under only anhydrous conditions still led to formation of the desired product in good yield (64%), albeit with lower diastereoselectivity (3.0:1), in the absence of sacrificial aldehyde, only a low amount of the 1,4-dicarbonyl compound was obtained (31%), while the diastereoselectivity remained unchanged (6.7:1) (Figure 3, entries 1, 2).

The observed trends in product distribution suggest that, while water initially ensures that the highly electrophilic thionium intermediate is quickly hydrolyzed, suppressing pathways of elimination or epimerization in the α-position, the sacrificial aldehyde likely scavenges the thiol byproduct released upon hydrolysis, driving the reaction toward increased product formation. Notably, alternative carbonyls such as acetone were also found to be competent thiol scavengers (Figure 3, entry 3), albeit leading to lower reaction yields, but increased diastereoselectivity.

When considering different nonparticipating sulfoxide substituents, other than alkyl groups, *p*-tolyl sulfoxides were at once appealing. Their facile enantioselective synthesis takes place from commercially available menthyl sulfinates^29^ under established conditions. To our delight, even better diastereoselectivity with comparable reaction yield was observed when *p*-tolyl sulfoxide was employed (Figure 3, entry 4).

In an effort to achieve a diastereodivergent rearrangement in which the geometry of the double bond dictates the diastereoselective formation of 1,4-dicarbonyl, we turned our focus to *Z*-vinyl sulfoxides to unlock *anti*-configured 1,4-dicarbonyls. Indeed, we were able to obtain satisfactory results for the desired *anti*-configured 1,4-dicarbonyl using *n*-octyl-substituted sulfoxides (Figure 3, entry 5). To our surprise, however, no significant diastereoselectivity could be achieved with *p*-tolyl-substituted *Z*-vinyl sulfoxides (Figure 3, entry 6). In addition, compared to *n*-octyl-substituted sulfoxides, a significantly lower reaction yield was observed along with the formation of additional byproducts. This was attributed to, in the presence of a *Z*-configured vinyl group, the occurrence of a competing [3,3]-sigmatropic rearrangement onto the aromatic moiety.^30−32^ This suspicion was further corroborated by NMR analysis of the crude material, suggesting as much as a 32% yield of the *ortho*-functionalized aryl vinyl sulfide **7a**. We also observed the occurrence of the undesired [3,3]-sigmatropic rearrangement in β,β-disubstituted vinyl sulfoxides, albeit in reduced quantities (17% for **7b**, Figure 3, bottom). In this case, the more electron-rich double bond might accelerate the rearrangement onto the vinyl group, suppressing the reaction onto the aryl moiety, compared with the disubstituted alkene in **7a**.

The origin of this dramatic influence of the sulfur substituent was then further investigated using density functional theory (DFT) calculations, which are discussed in detail below (*vide infra*, Figure 5).

Overall, the results above show that two different types of sulfoxide moieties must be considered. These are: an aryl group (*p*-tolyl) or an alkyl chain (*n*-Oct or Me; negligible difference between the two was observed).^28^ While the *p*-tolyl group is well suited for (*E*)-vinyl sulfoxides, which are easily accessible in enantiopure form, enantiopure sulfoxides bearing an alkyl substituent are necessary to obtain good results for (*Z*)-vinyl sulfoxides and have also proven to be beneficial for β,β-disubstituted vinyl sulfoxides. While the preparation of the latter is less straightforward than the former, this empirical “rule of thumb” proved helpful in our studies.^16,33^

### Toward an Asymmetric Version

**Figure 4:**
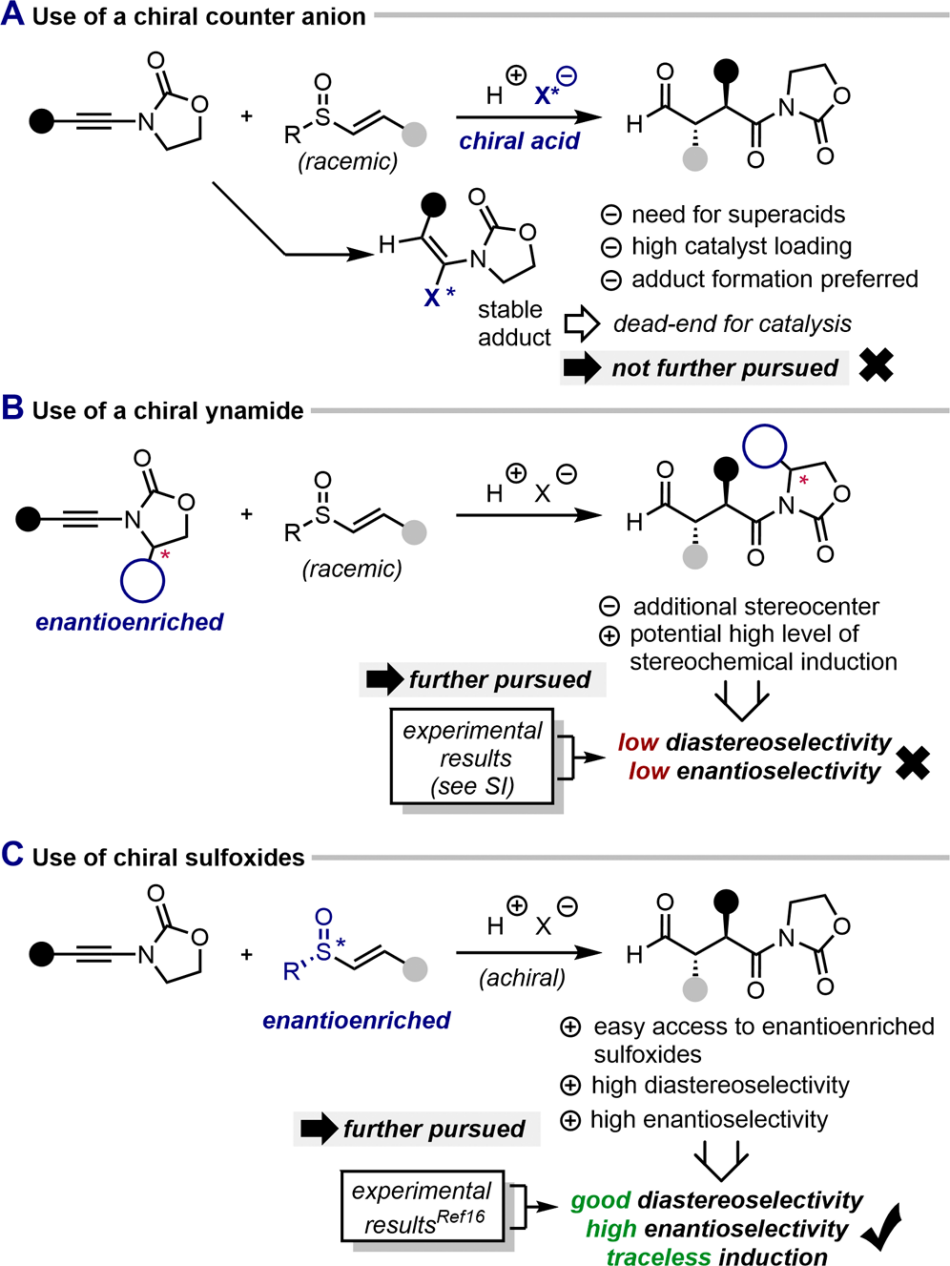
Three options toward an asymmetric version of the [3,3]-sigmatropic sulfonium rearrangement. (A) Use of a chiral counter anion. (B) Use of a chiral ynamide. (C) Use of chiral sulfoxides.

Striving for an asymmetric version of the 1,4-dicarbonyl synthesis, we considered three strategies: (1) the use of a chiral catalyst, which would render the reaction enantioselective (Figure 4A); (2) the use of a chiral auxiliary on the ynamide reaction partner, which would render the reaction diastereoselective (and deliver one enantiomer of the product after auxiliary cleavage) (Figure 4B); or (3) the use of a chiral sulfoxide, which would render the reaction enantiospecific and traceless (Figure 4C).

The first option appeared attractive, especially given the body of literature on chiral acid catalysis.^34,35^ In practice, such a strategy typically relies on a highly acidic and, therefore, non-nucleophilic, chiral acid. In previous reports,^36^ and in our hands, even otherwise poorly nucleophilic (chiral) Brønsted acids quickly resulted in covalent adducts of remarkable stability (see the SI for details).

The second strategy is especially appealing due to the large number of common oxazolidinone derivatives that are used as chiral auxiliaries.^37^ When this approach was put to the test, however, a mixture of diastereoisomers was observed (see the SI for further details) with only a modest preference for a specific isomer. This unusual result most likely stems from the pronounced stereodetermining effect of the vinyl sulfoxide itself.

Gratifyingly, the third strategy—the use of a chiral sulfoxide—immediately succeeded and proceeded with high-level *S*-to-*C* chirality transfer, allowing 1,4-dicarbonyls to be synthesized in essentially enantiopure form.^16^

### Computational Studies

**Figure 5:**
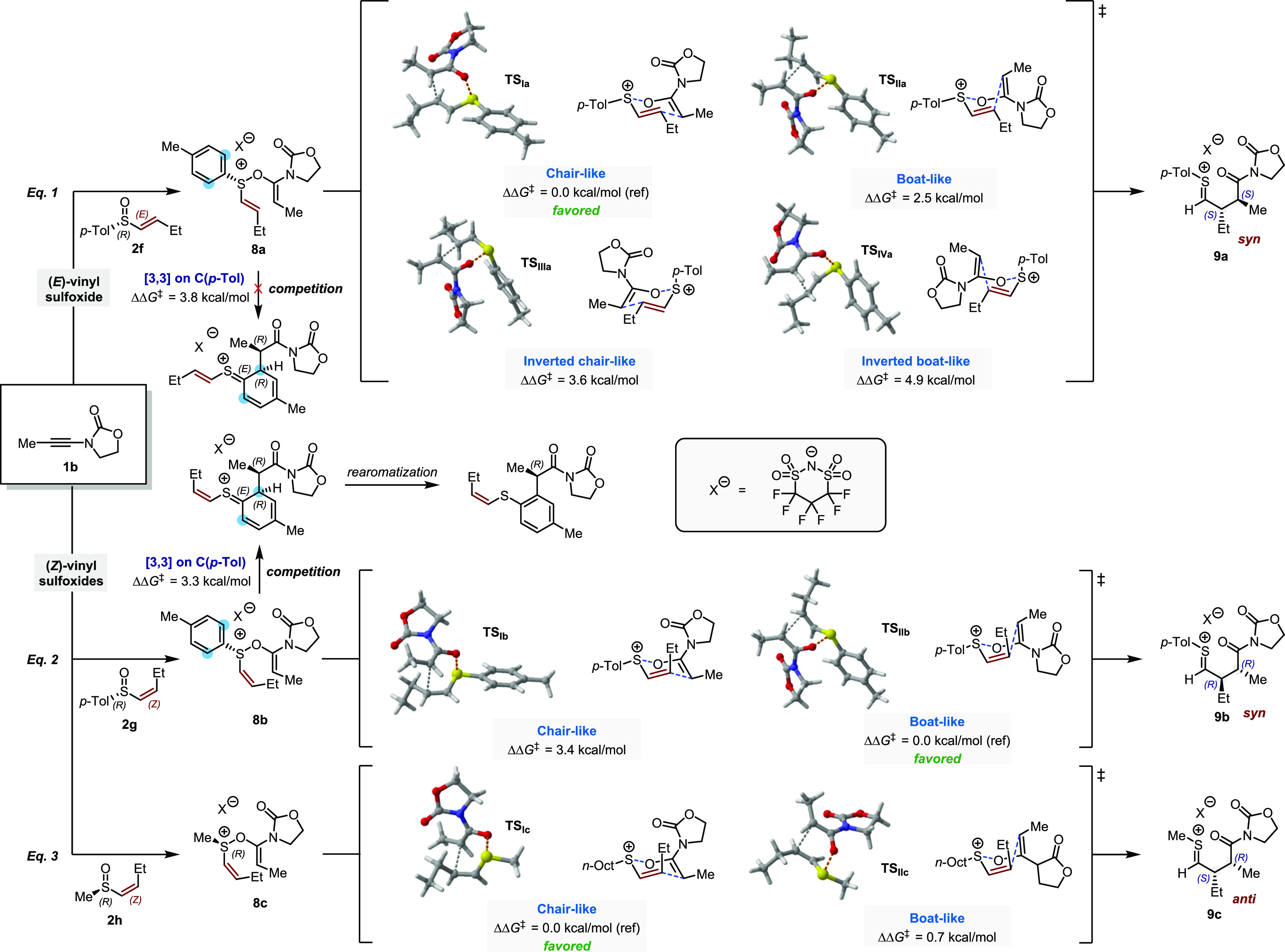
Computational study for the use of chiral sulfoxides: ΔΔ*G*^‡^ Gibbs free energy values relative to the most stabilized transition state (assigned a reference value of 0.0 kcal/mol) for each applied sulfoxide at 273.15 K.

To gain a better understanding of the reaction profile and, more specifically, the enantiodetermining step, we performed a computational analysis of the [3,3]-sigmatropic rearrangement of ynamide sulfoxide adducts **8a**–**c** to thionium ions **9a**–**c** (Figure 5).

Density functional theory (DFT) computations were performed at the PBE0-D3(BJ)/def2-TZVP level of theory (see the Supporting Information for details).^38−44^

It is worth noting that the complexity of elucidating diastereoselectivity necessitated precise structure optimization, exploiting the triple-ζ quality basis set def2-TZVP for relatively large molecular systems (transition state complexes with counterion).^45^ The commonly employed cost-effective approach of combining a double-ζ basis set for structural optimization with a triple-ζ basis set for single-point energy calculations proved to be unsatisfactory for this study.

Starting with **8a**, arising from the addition of *p*-tolyl-substituted, (*R*)-configured (*E*)-vinyl sulfoxide **2f** to ynamide **1b**, we analyzed the four possible transition state families of the sigmatropic rearrangement. These are two chair- and boat-like transition states with the nonparticipating sulfur substituent in a *pseudo*-equatorial alignment, as well as the two analogous structures where the substituent adopts a *pseudo*-axial orientation, which we termed “inverted chair-like” and “inverted boat-like” transition state, respectively (Figure 5, eq 1). As expected, calculations showed the two inverted transition states to be much higher in energy (**TS**_**IIIa**_, + 3.6 kcal/mol and **TS**_**IVa**_, + 4.9 kcal/mol, respectively, compared to the most stable transition state **TS**_**Ia**_) than their counterparts in which the nonparticipating sulfur substituent adopts a *pseudo*-equatorial orientation. Between chair-like and boat-like transition states **TS**_**Ia**_ and **TS**_**IIa**_, the latter was found to be disfavored by 2.5 kcal/mol. These results obtained for this model system correlated well with the stereochemical outcome and the high stereoselectivity observed (theoretical d.r. = 99:1 at 0 °C for ΔΔ*G*^‡^ = 2.5 kcal/mol). The hypothetical dearomative [3,3]-sigmatropic rearrangement involving the *p*-tolyl group, toward an *ortho*-functionalized product, was found to proceed with a relatively high activation barrier (ΔΔ*G*^‡^ = 3.8 kcal/mol) for (*E*)-vinyl sulfoxide, aligning with the experimental findings, where no such aromatic rearrangement was observed.

In agreement with our previous studies of sulfonium sigmatropic rearrangements, all calculated structures were found to be early transition states with a very short S–O bond (*d*_S–O_ = 2.04 vs *d*_S–O_ = 1.66 Å in intermediate **8a**) and a highly elongated C–C bond (*d*_C–C_ = 2.71 Å in **TS**_**Ia**_ vs *d*_C–C_ = 1.55 Å in product **9a**).^32,46^

We next turned our attention toward (*Z*)-vinyl sulfoxides (Figure 5, eq 2). First, we aimed to uncover the origin of the different diastereoisomeric outcomes with *n*-alkyl and *p*-tolyl sulfur substituents. As before, the inverted chair-like and inverted boat-like transition states were found to be considerably higher in energy and are therefore not shown (+6 to 9 kcal/mol, see the Supporting Information). Surprisingly, in the case of *p*-tolyl vinyl sulfoxide **2g**, the boat-like transition state **TS**_**IIb**_, giving the (*R,R*)-1,4-dicarbonyl product (*ergo* a *syn-*1,4-dicarbonyl), was found to be favored over the corresponding chair-like transition state **TS**_**Ib**_ (+3.4 kcal/mol). Conversely, the sulfoxide carrying an *n*-methyl group showed a preference of 0.7 kcal/mol for chair-like transition state **TS**_**Ic**_ (Figure 5, eq 3), leading to the (*R,S*)-1,4-dicarbonyl (i.e., an *anti*-1,4-dicarbonyl), being favored over a boat-like transition state **TS**_**IIc**_, a result that is in good agreement with the divergent stereochemical outcome observed.

Notably, for (*Z*)-vinyl sulfoxide **2g**, the barrier of the dearomative [3,3]-sigmatropic rearrangement, leading to arylated byproduct, becomes competitive. Indeed, it is comparable to and even slightly lower than the alternative pathway via chair-like **TS**_**Ib**_ (ΔΔ*G*^‡^ ([3,3]) = 3.3 kcal/mol relative to the most stable transition state **TS**_**IIb**_), making this side reaction more feasible. This could explain the reduced yields of the main product **9b** when (*Z*)-configured vinyl sulfoxides are used (the structures of the transition states for these side reactions are not shown, see the Supporting Information for details).

### Synthesis of γ-C Lactones

Having explored the factors governing the diastereoselectivity of our 1,4-dicarbonyl synthesis,^16^ we were further intrigued by the possibility of converting the oxazolidinone-bearing adducts of this transformation into further valuable products.

**Figure 6:**
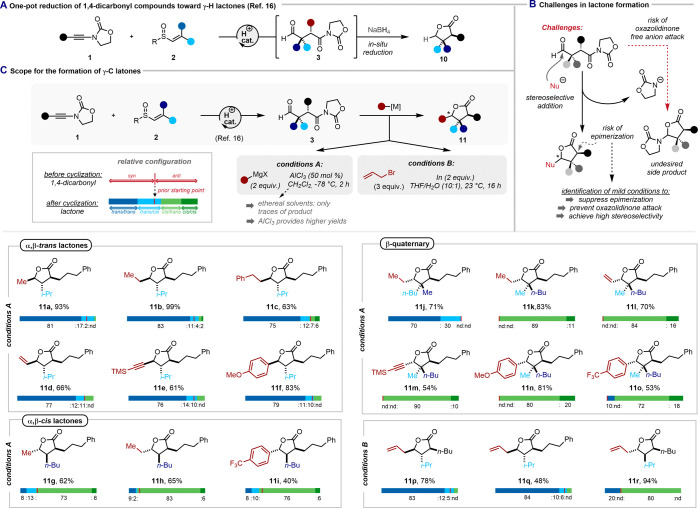
Synthesis of γ-H and γ-C lactones. (A) Reduction of 1,4-dicarbonyl compounds by in situ reaction with sodium borohydride. (B) Challenges in lactone formation. (C) Scope for the reaction between 1,4-dicarbonyl compounds and organometallics. The red bar represents the diastereoisomeric ratio of 1,4-dicarbonyl (the starting material for the cyclization); the green/blue bars represent the diastereoisomeric distribution of the lactone after the cyclization.

While we previously showed that the direct reduction of products such as **3** with sodium borohydride smoothly afforded the corresponding γ-H lactone **10** with good stereoselectivity, a feature that also proved useful for analytical purposes (Figure 6A, see the SI for details),^16^ we were eager to explore other modes of functionalization. Here, the identification of mild reaction conditions, suppressing epimerization of the acidified stereocenters adjacent to the carbonyls, was crucial (Figure 6B). Additionally, early attempts had revealed that the oxazolidinone anion, released upon lactone or lactam cyclization, is also a suitable nucleophile and could compete with, and even outcompete, the desired nucleophile (Figure 6B).^16^

Focusing on carbon-centered nucleophiles,^47,48^ we commenced our studies by assessing the ability of common organometallic reagents to induce lactone formation (Figure 6C).^49^

In light of the aforementioned challenges, initial experiments with Grignard reagents in ethereal solvents quickly resulted in complex product mixtures with only small amounts of the desired lactone products (see the SI for further details). The desired breakthrough was achieved only when DCM was employed as a cosolvent, which simultaneously ensured good diastereoselectivities and high yields. Furthermore, our optimization studies revealed that the addition of AlCl_3_ provided a slightly higher reaction yield compared to the additive-free version, which could be explained by its Lewis acidity, corresponding to its ability to activate the aldehyde/oxazolidinone and/or to bind the released oxazolidone anion.^50,51^

Under these reaction conditions, we were pleased to observe that a *syn-*configured 1,4-dicarbonyl could be smoothly converted to the *trans*/*trans* γ-C-substituted lactone **11a** in excellent yield (93%) and with good diastereoselectivity (d.r. = 81:17:2:nd).^52^ We further found that C(sp^3^)-, C(sp^2^)- and C(sp)-hybridized Grignard reagents could be used as nucleophiles, yielding γ-alkyl (**11b**, **11c**), γ-vinyl (**11d**), γ-aryl (**11f**), or γ-alkynyl (**11e**) lactones in moderate to excellent yields and, notably, without detectable erosion of the diastereomeric ratio.

Similarly, under the same reaction conditions, *anti*-configured 1,4-dicarbonyls led to the corresponding γ-substituted *cis*/*trans*-γ-lactones (**11g**–**11i**) without a loss of diastereomeric excess. Additionally, aldehydes featuring an adjacent quaternary center yielded the β-quaternary lactones steroeoselectively (**11j**–**11o**), with a preferred addition of the nucleophile from the same side as the sterically less demanding substituent.^53^ While the reaction was amenable to more encumbered C(sp^2^)-hybridized Grignard reagents, such as aryl magnesium bromides, secondary C(sp^3^)-Grignard reagents mainly afforded products of reduction via competing hydride transfer from the organometallic reagent, a common observation.^54−56^ We further considered organoindium reagents as desirable due to their tolerance for protic media (such as water or alcohols; see conditions **B** in Figure 6C). Indeed, organoindium (in situ generated from allyl bromide and elemental indium under Barbier conditions) allylations proceeded smoothly with high selectivity. Comparably to the previous method, both *trans*/*trans* and *cis*/*trans* γ-lactones were accessible in moderate to excellent yields and with good selectivities.

### Synthesis of γ-O Lactones

**Figure 7:**
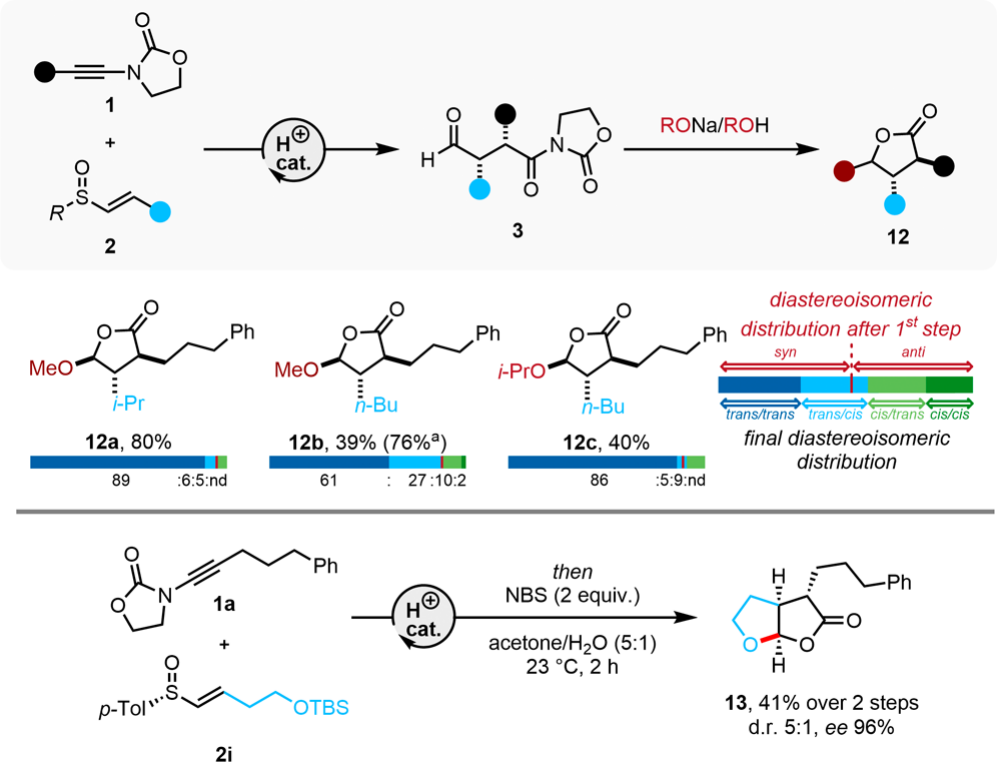
Synthesis of γ-O-lactones. See the Supporting Information for the exact reaction conditions. ^a^Yields were determined by ^1^H NMR analysis of the reaction crude using mesitylene as an internal standard. Numbers in parentheses represent the NMR yield. d.r. = diastereoisomeric ratio; ee = enantiomeric excess; NBS = *N*-bromosuccinimide; *p*-Tol = *para*-tolyl.

Encouraged by the stereoselective introduction of carbon substituents, we additionally explored the formation of γ-alkoxy lactones (Figure 7).

Gratifyingly, no epimerization of the dicarbonyl precursor was observed under the basic conditions employed. Interestingly, bicyclic lactones can be obtained through domino cyclization when the vinyl sulfoxide partner (**2i**) carries a tethered protected oxygen. In this case, the intermediate thionium ion was intramolecularly captured by the tether, with bicyclic lactone product **13** resulting after treatment with *N*-bromosuccinimide (see the Supporting Information for a proposed mechanism).

### Synthesis of γ-Lactams

**Figure 8:**
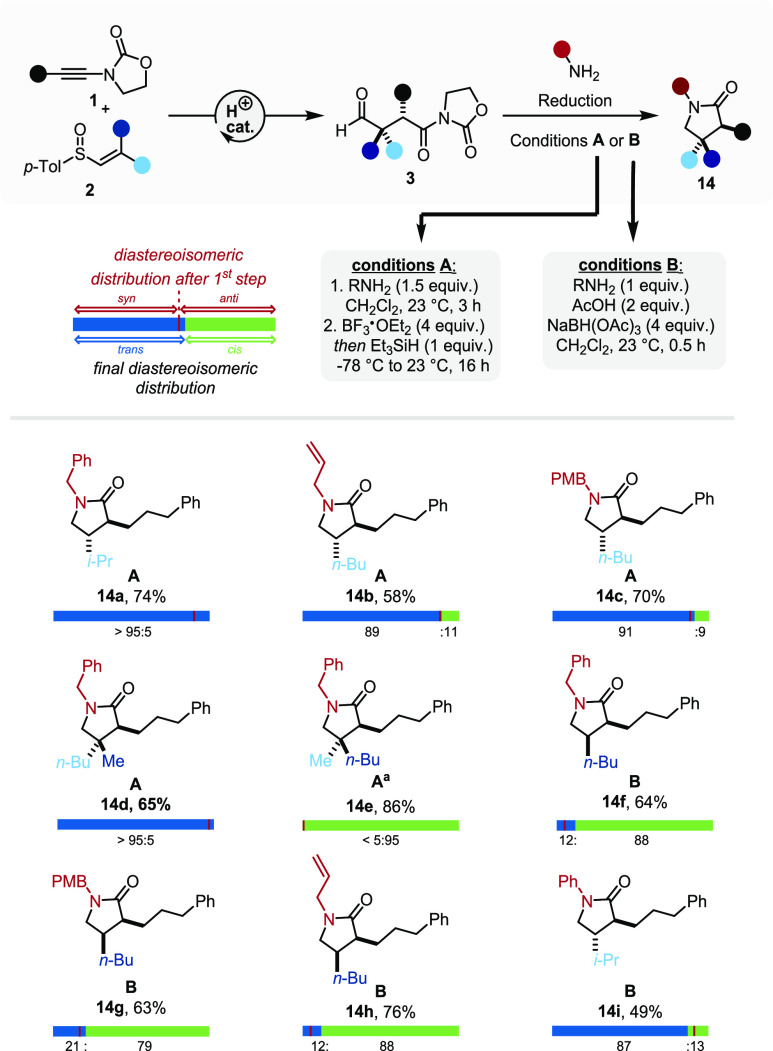
Synthesis of γ-lactams. ^a^The β-quaternary aldehyde was left to react with the corresponding amine for 16 h; PMB = *para*-methoxybenzyl.

Knowing that an aza-variant of the previously described transformations would provide access to the respective γ-lactams,^57,58^ we investigated different protocols (Figure 8). In the event, two sets of suitable conditions were identified: cyclization to a γ-hydroxy lactam prior to the addition of a suitable reductant (conditions **A**) or direct reduction of the imine with concomitant spontaneous cyclization (conditions **B**).

Using the first set of conditions (conditions **A**) proved to be ideally suited for the synthesis of γ-lactams bearing α,β-*trans*-substitution. We presume that putative equilibration via acyliminium/enamide species leads to an enhancement of the starting diastereomeric ratio, favoring the thermodynamic *trans*-product (**14a**, **14c**).^59^

Nonepimerizable β-quaternary aldehydes were also converted to the corresponding β-quaternary γ-lactams under these reaction conditions, affording the products with perfect stereospecificity (**14d**, **14e**). On the other hand, when targeting α,β-*cis*-substitution, direct reductive amination conditions in acidic media were superior and avoided the aforementioned equilibration (conditions **B**). To this end, NaBH(OAc)_3_ and acetic acid allowed the synthesis of *cis*-configured γ-lactams with good yields and only a minor decrease in the diastereomeric ratio (**14f**–**14h**). These conditions were also found to be suitable for aniline, a far less nucleophilic nitrogen, and the corresponding *N-*phenyllactam (**14i**) was obtained in good yield.

**Figure 9:**
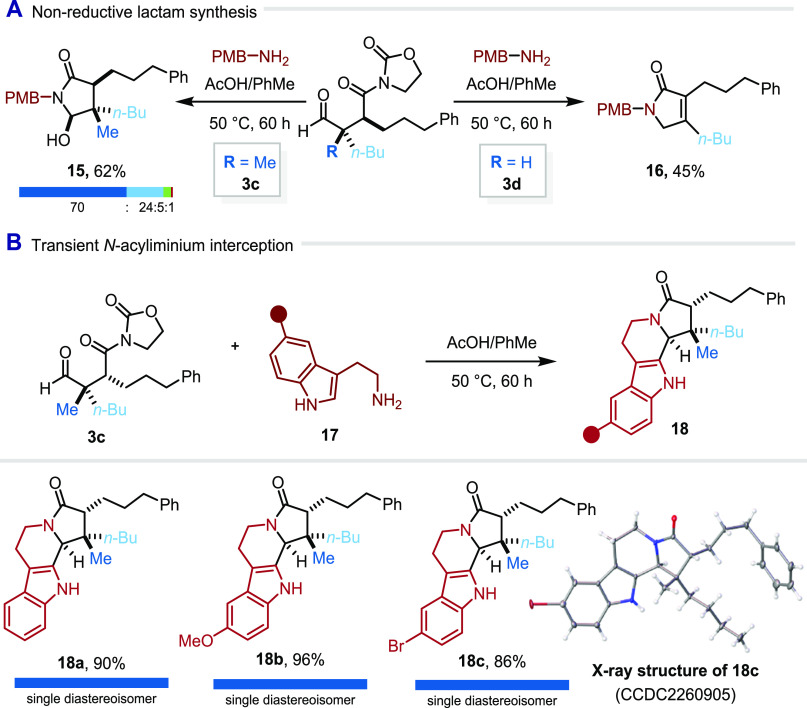
Nonreductive lactam formation. (A) With benzylamines. (B) With tryptamine derivatives; PMB = *para*-methoxybenzyl.

Aiming to gain insight into the intermediates and side products involved in this process, we performed the reaction in the absence of hydride donors or nucleophiles (Figure 9A). Interestingly, two different products were obtained, depending on the nature of the substrate. For substrates lacking hydrogens α-to the aldehyde (i.e., quaternary center), γ-hydroxy lactams such as **15** were obtained in good yield and with moderate diastereoselectivity on the additional hemiaminal stereocenter. On the other hand, when an α-tertiary aldehyde was used, α,β-unsaturated lactam **16** was obtained, likely formed through elimination to an enamide followed by isomerization.

Eager to explore the reactivity of amines bearing an additional tethered nucleophile, we found that tryptamine derivatives (**17**) deliver β-carbolines (**18**) under acidic conditions (Figure 9B). These tetracyclic compounds were obtained in excellent yields (86–96%) and diastereoselectivities (single diastereoisomer detected in all cases). The relative configuration of the products was confirmed by single-crystal X-ray analysis of **18c**.

### Application—Total Synthesis of Paraconic Acids

**Figure 10:**
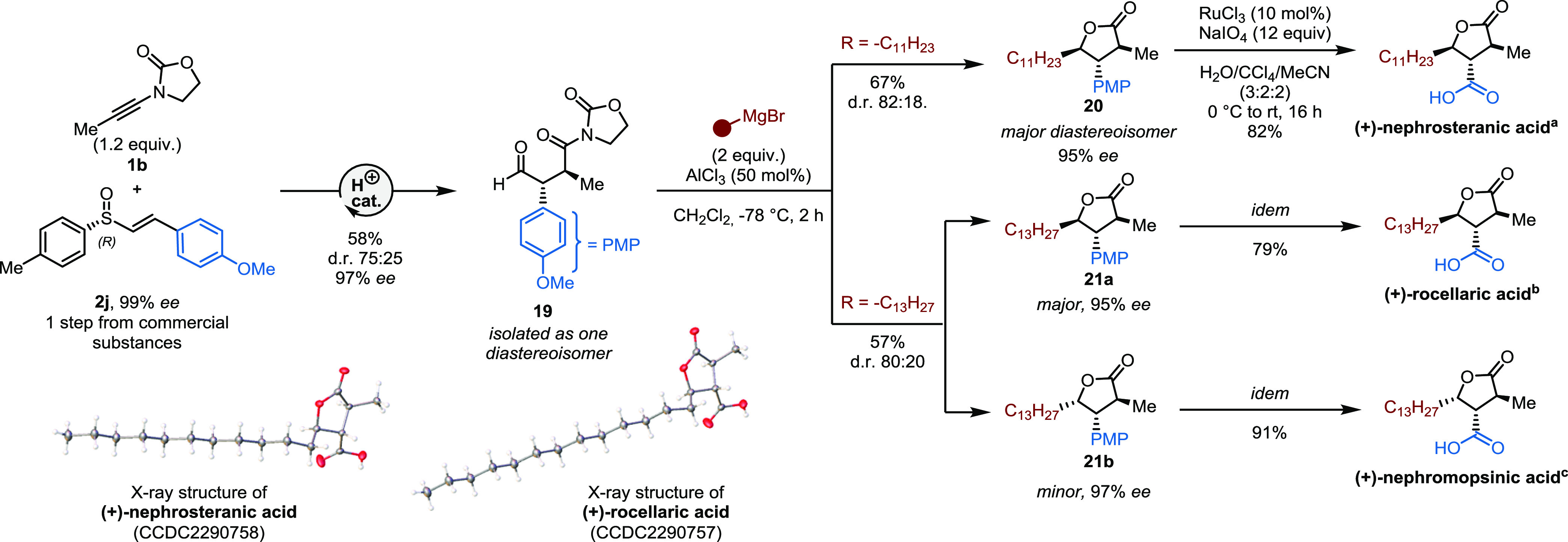
Total synthesis of paraconic acids using charge-accelerated sulfonium rearrangement followed by lactone formation and elaboration. d.r. = diastereoisomeric ratio. ^a^[α]_D_^20^ = +24.7 (*c* = 0.4, CHCl_3_), lit. [α]_D_^23^ = +23.3 (*c* = 0.2, CHCl_3_ for 96% ee). ^b^[α]_D_^20^ = +21.7 (*c* = 1.1, CHCl_3_), lit. [α]_D_^20^ = +16.7 (*c* = 0.2, CHCl_3_ for 96% ee). ^c^[α]_D_^20^ = −76.0 (*c* = 0.25, CHCl_3_), lit. [α]_D_^23^ = −85.7 (*c* = 0.5, CHCl_3_ for 95% ee); PMP = *para*-methoxyphenyl.

To further illustrate the potential of our strategy, we deployed it in the enantioselective total syntheses of paraconic acids (Figure 10). Paraconic acids are natural products, isolated from lichens, which possess antineoplastic and antibiotic properties, making them desirable targets for synthesis.^3,60−68^ We set out to prepare these compounds through an unconventional, divergent route. The key 1,4-dicarbonyl compound **19** was smoothly obtained with high enantioselectivity, but moderate diastereoselectivity,^69^ employing readily available starting materials (**1b**, **2j**).^70,71^ This intermediate could then be exposed to alkyl Grignard reagents, using the lessons learned above, to give the corresponding γ-C-lactones **20** and **21** in good yields and with excellent enantiospecificity. Ultimately, **20** and **21a** were subjected to a ruthenium-catalyzed aromatic oxidation,^63^ providing (+)-nephrosteranic acid and (+)-rocellaric acid, as also confirmed by X-ray structural determination.^72,73^ The minor isomer (**21b**), obtained during the synthesis of **21a**, could be oxidized to another paraconic acid, (+)-nephromopsinic acid. This constitutes a concise (4 steps in the longest linear sequence), high-yielding, stereoselective, and divergent route toward these targets.

## Conclusions

In the first part of this work, we detail our comprehensive investigation of various factors affecting the outcome of sulfonium-accelerated rearrangements triggered by the combination of ynamides and enantioenriched vinyl sulfoxides and retrace the development of a stereodivergent and highly efficient synthesis of 1,4-dicarbonyl compounds. Through extensive density functional theory (DFT) studies, we have clarified the factors responsible for the (empirically observed) choice of the nonparticipating substituent for both (*E*)- and (*Z*)-vinyl sulfoxides. In the second part of our work, we describe a new platform for the preparation of highly substituted γ-C-lactones, γ-O-lactones, and γ-lactams from the 1,4-dicarbonyl products, including domino processes (to obtain β-carbolines or bicyclic lactones). These cyclizations exhibit high stereoselectivity under mild and straightforward reaction conditions and pave the way for a relevant case study: the enantioselective four-step total syntheses of (+)-nephrosteranic acid, (+)-rocellaric acid, and (+)-nephromopsinic acid.

## Supporting Information Available

Experimental procedures and characterization data for all new compounds and computational details (PDF)
