# Supplementary material for: Stereodivergent Synthesis of 1,4-Dicarbonyl Compounds through Sulfonium Rearrangement: Mechanistic Investigation, Stereocontrolled Access to γ-Lactones and γ-Lactams, and Total Synthesis of Paraconic Acids
Source: J Am Chem Soc. 2024 May 13;146(20):13914–23. doi: 10.1021/jacs.4c01755 (PMC11117187; doi:10.1021/jacs.4c01755)
Supplement: Supplementary file 1 — ja4c01755_si_001.pdf [file ja4c01755_si_001.pdf]

Supplementary information

Stereodivergent Synthesis of 1,4-Dicarbonyl Compounds through Sulfonium Rearrangement: Mechanistic Investigation, Stereocontrolled Access to γ-Lactones and γ-Lactams and Total Synthesis of Paraconic Acids

Nicolas G.-Simonian[#,a], Philipp Spieß[#,a], Margaux Riomet [a], Boris Maryasin [a,b], Immo Klose [a], Alexander Beaton Garcia [a], Laurin Pollesböck [a], Dainis Kaldre [a], Uroš Todorovic [a], Julia Minghua Liu [b], Daniel Kaiser [a], Leticia González [b] and Nuno Maulide [a,*]

[a]Institute of Organic Chemistry, University of Vienna, Währinger Straße 38, 1090 Vienna, Austria

[b]Institute of Theoretical Chemistry, University of Vienna, Währinger Straße 14, 1090 Vienna, Austria

Contents

1 General Information 4

2 Experimental Section 5

2.1 Preparation of 1,4-dicarbonyl compounds 5

2.1.1 General procedure A: Preparation of 1,4-dicarbonyl compounds 5

2.1.2 Observation of undesired [3,3]-rearrangement product for **7a** 6

2.2 Chiral Brønsted-Acid Attempts 7

2.3 Chiral ynamide in [3,3]-sigmatropic rearrangement 8

2.3.1 Preparation of starting material 8

2.3.2 Results of [3,3]-sigmatropic rearrangement 10

2.4 Preparation of γ-C lactones 12

2.4.1 General Procedure B: Formation of γ-C lactones 12

2.4.2 Optimization results 12

2.4.3 Explanations for stereochemical outcomes 13

2.4.4 General Procedure C: Formation of γ-allyl lactones 14

2.4.5 Characterization of products 15

2.5 Formation of *O*-lactones 33

2.6 Synthesis of γ-lactams 38

2.6.1 General procedure D: Synthesis of lactam using Et_3SiH 38

2.6.2 General procedure E: Synthesis of lactam using $NaBH(OAc)_3$ 39

2.6.3 Characterization of products 39

2.7 Non-reductive formation of lactams 49

2.8 Pictet-Spengler type annulation with tryptamines 51

2.8.1 General procedure F: Pictet-Spengler type annulation 51

2.8.2 Characterization of products 51

2.9 Total synthesis of paraconic acids ... 54

2.9.1 Synthesis of Me-ynamide (**1b**) ... 54

2.9.2 Synthesis of E-vinyl sulfoxides (**2j**) ... 54

2.9.3 Synthesis of 1,4-dicarbonyls (**19**) ... 56

2.9.4 Synthesis of lactones (**20**, **21**) ... 59

2.9.5 Synthesis of Paraconic Acids ... 66

3 DFT study ... 69

3.1 Computational details ... 69

3.2 Kinetics of alternative diastereoselective pathways – computation of transition states ... 69

3.3 Analysis of the stereoselectivity of sulfoxide addition – *E*- vs. *Z*-enolonium ion formation ... 71

3.4 XYZ coordinates for the most stabilized conformations ... 73

4 X-ray structural analysis data ... 83

5 NMR spectra ... 90

6 References ... 138

1 General Information

General procedures. All reactions were performed in round bottom flasks or vials fitted with rubber septa with magnetic stirring, unless otherwise stated. Reaction vessels were flushed with argon prior to use, unless otherwise stated. Liquids and solutions were transferred via syringe. All reactions were performed using anhydrous solvents from Acros Organics, TCI or Sigma-Aldrich. Reaction progress was monitored by thin layer chromatography (TLC) performed on aluminum plates coated with silica gel F_{254} with 0.2 mm thickness. Chromatograms were visualized by fluorescence quenching with UV light at 254 nm or by staining using potassium permanganate followed by heating. Flash column chromatography was carried out on 230–400 mesh silica gel (Merck and co.) using reagent grade solvents.

Materials. All commercial reagents and solvents were used without further purification.

Instrumentation. ^{1}H NMR and ^{13}C NMR spectra were recorded using a Bruker AV-400, AV-500, AV-600 or AV-700 spectrometer at 300 K. Chemical shifts (δ) are given in parts per million (ppm), referenced to the solvent peak of $CDCl_3$, defined at δ = 7.26 ppm (^{1}H NMR) and δ = 77.16 ppm (^{13}C NMR). Coupling constants (J) are reported in Hertz (Hz). ^{1}H NMR splitting patterns are designated as singlet (s), doublet (d), triplet (t), quartet (q), quintuplet (quint.) as they appeared in the spectrum. If the appearance of a signal differs from the expected splitting pattern, the observed pattern is designated as apparent (app). Splitting patterns that could not be interpreted or easily visualized are designated as multiplet (m) or broad (br). Infrared (IR) spectra were obtained using Perkin-Elmer Spectrum 100 FT-IR spectrometer. Wavenumbers ($\nu = \lambda^{-1}$) are reported in cm^{-1}. Mass spectra were obtained using a Bruker maXis UHR-TOF spectrometer (70 eV), using electrospray ionization (ESI) or an Agilent 7200B GC/Q-TOF spectrometer (70 eV), using electron ionization (EI). Optical rotations were measured on a Perkin Elmer 341 polarimeter using a 100 mm path-length cell at 589 nm (c given in g / (100 mL)). Chiral HPLC was performed using an AGILENT Infinity 1260 with Chiralpak IC, Lux-3 Cellulose-3 or Chiracel OD-H columns. Details of chromatographic conditions are indicated under each compound.

2 Experimental Section

2.1 Preparation of 1,4-dicarbonyl compounds

1,4-Dicarbonyl compounds were all prepared using a slightly modified procedure from Kaldre, D.; Klose, I., Maulide, N. *Science* **2018**, *361*, 664-667, which also report all analyses of the 1,4-dicarbonyl compounds which were used in this study.[1]

2.1.1 General procedure A: Preparation of 1,4-dicarbonyl compounds

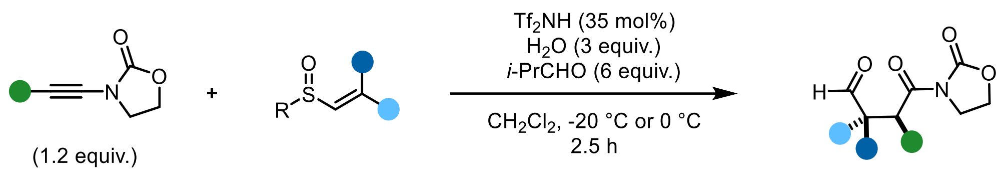

To a solution of the sulfoxide (1 equiv.), the ynamide (1.2 equiv.) and *i*-PrCHO (6 equiv.) in CH_2Cl_2 (0.2 M) was added water (3 equiv.) under vigorous stirring and the resulting mixture was cooled to the appropriate temperature (-20 or 0 °C). A solution of Tf_2NH (0.1 M in CH_2Cl_2, 20 or 35 mol%) was added over 30 min using a syringe pump. After the addition was complete, the mixture was left stirring at the same temperature for 2 h before being quenched with aq. $NaHCO_3$ (made by mixing 1:1 volume of water and sat. aq. $NaHCO_3$). The phases were separated and the aqueous phase was extracted with CH_2Cl_2. The combined organic phases were dried over $mgSO_4$, filtered and carefully concentrated *in vacuo*. The crude product was purified by flash column chromatography on silica gel (typically EtOAc/Heptane 5:95 to 30:70 using *i*-PrOH 1% as a modifier) to afford pure 1,4-dicarbonyl compound.

Troubleshooting:

- Tf_2NH is better stored in a glove box. Solutions of Tf_2NH in CH_2Cl_2 were made right before use.
- Water needs to be well dispersed in the medium. For good reproductivity, it is better to add water in the reaction mixture with a micro syringe with the tip immerged, while stirring vigorously. Sonication of the reaction mixture (1 min, before adding Tf_2NH) can also help.
- To ease purification, it is important to carefully evaporate the crude material (at least 1 h at 10^{-2} mbar).
- β,β-Disubstituted vinylsulfoxides give better d.r. when reaction is performed at -20 °C with 20 mol% Tf_2NH.

2.1.2 Observation of undesired [3,3]-rearrangement product for **7a**

Following general procedure A with **2d** as the sulfoxide source and **1a** as the ynamide, an unwanted alpha-arylated amide was observed in the crude NMR (32%), which was further confirmed by HRMS. However, we were not able to isolate it by column chromatography to fully characterize it as the compound decomposed.

^{1}H NMR (400 MHz, CDCl$_3$) of reaction crude

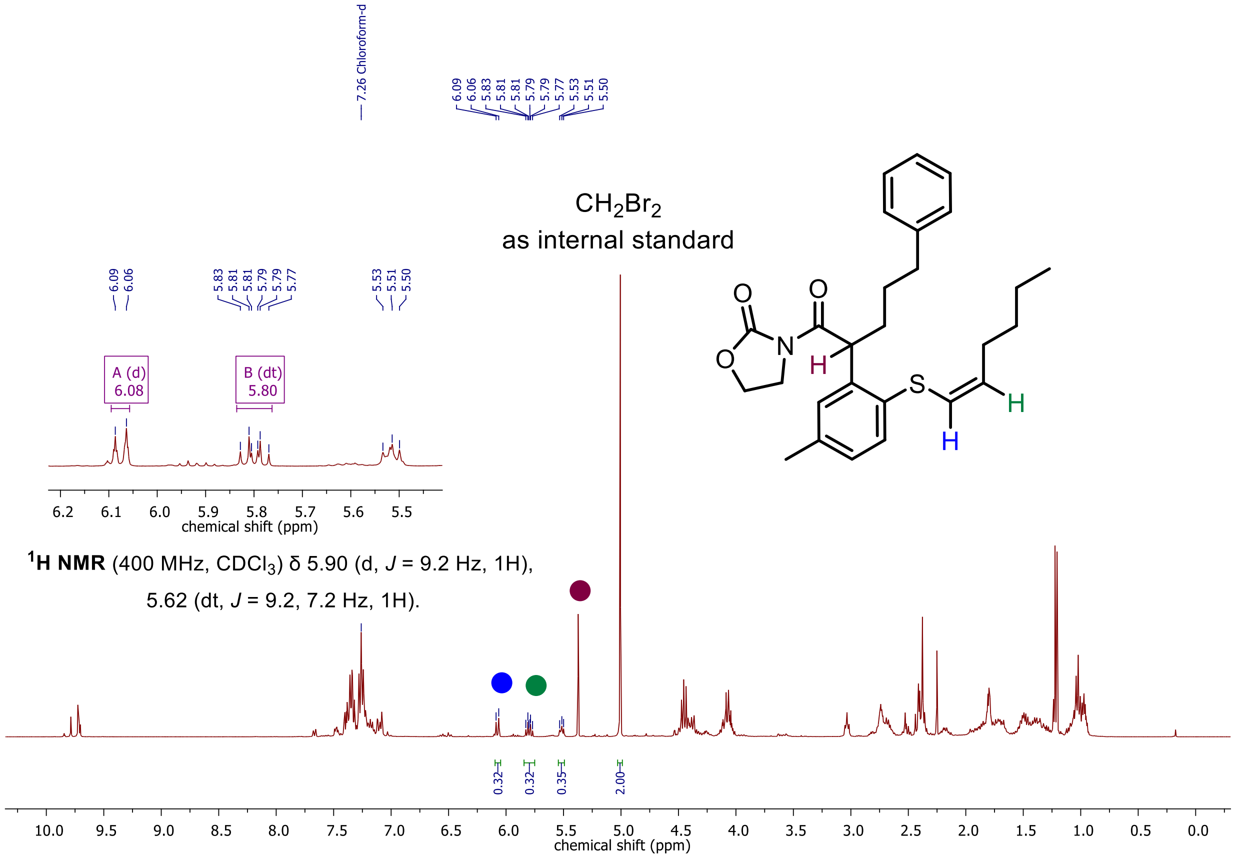

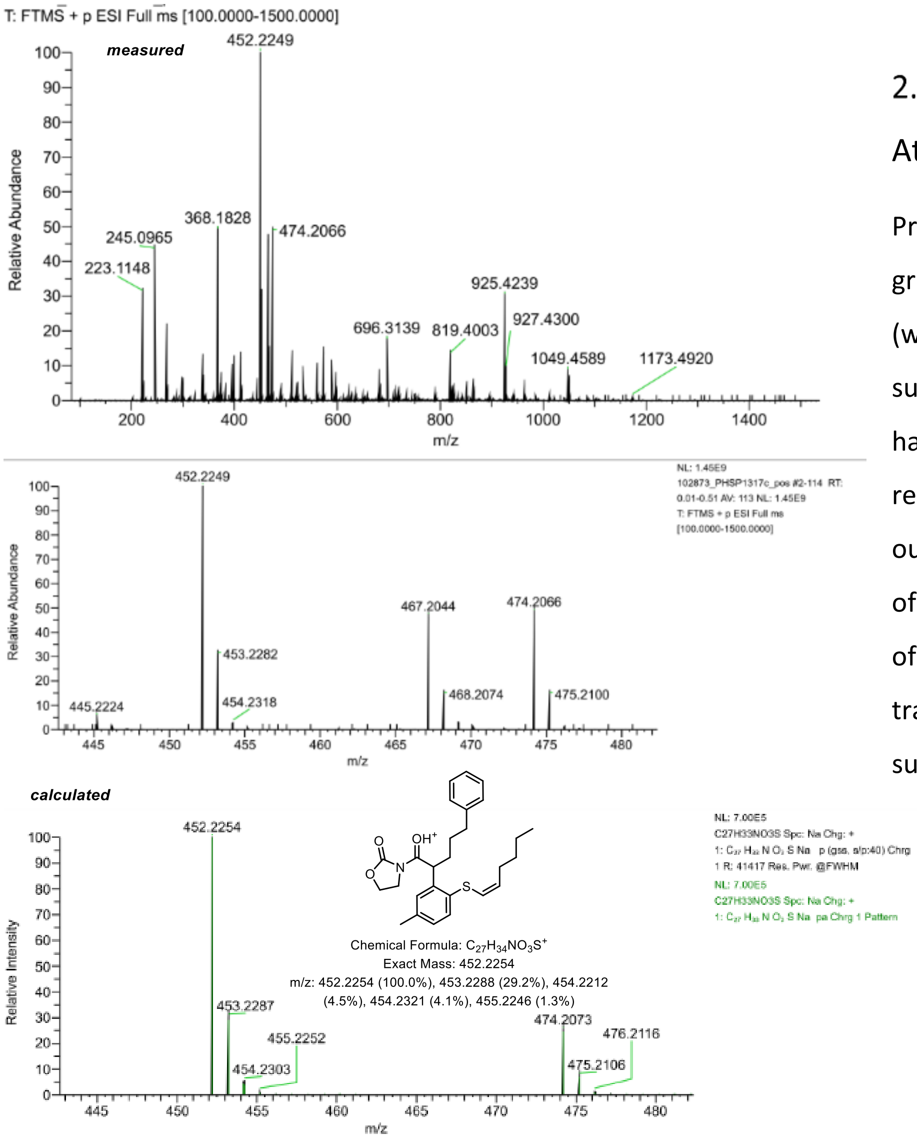

2.2 Chiral Brønsted-Acid Attempts

Previous experiments of our group with related sulfoxides (which also trigger [3,3]-sulfonium rearrangements) have shown the following results. Given these outcomes, the development of an enantioselective variant of the described transformation using vinyl sulfoxides was deemed exceedingly difficult:

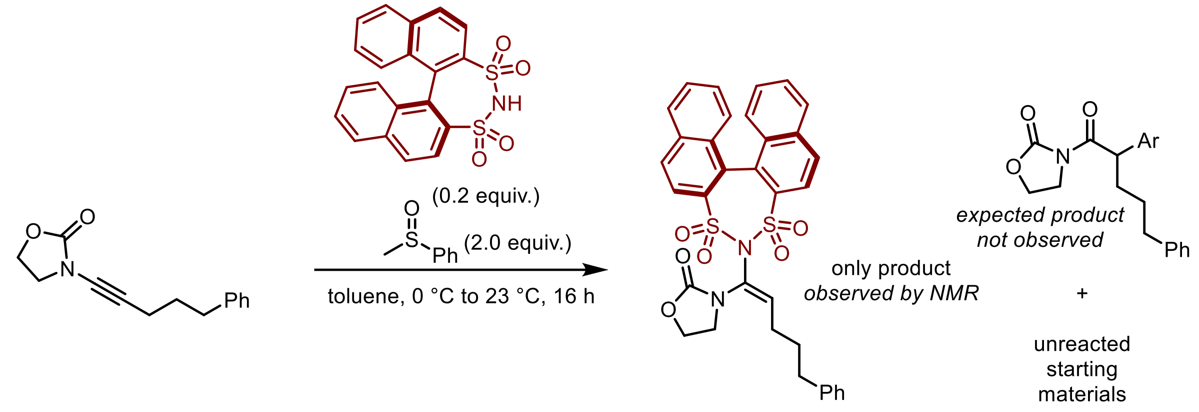

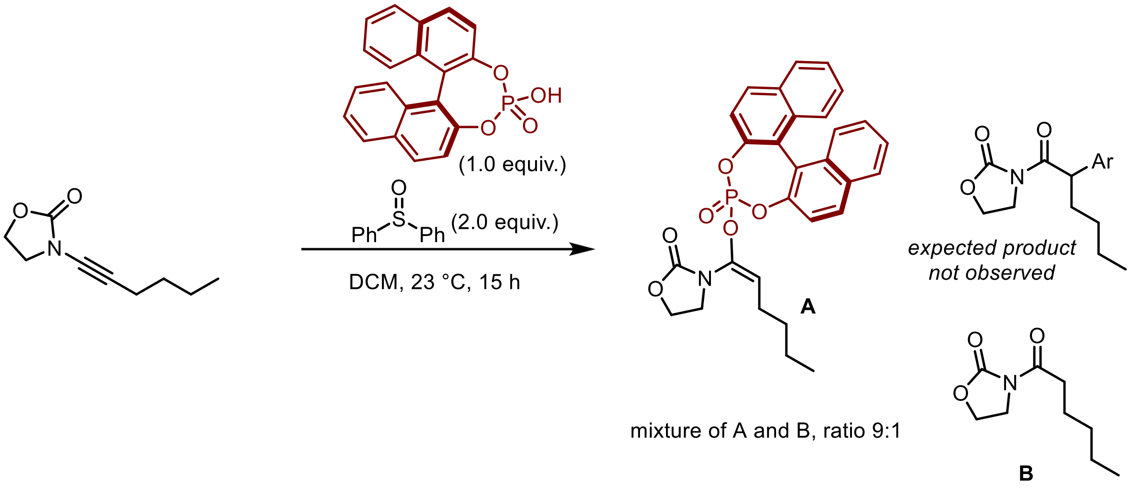

2.3 Chiral ynamide in [3,3]-sigmatropic rearrangement

2.3.1 Preparation of starting material

(*S*)-4-(*tert*-Butyl)-3-(5-phenylpent-1-yn-1-yl)oxazolidin-2-one (S1)

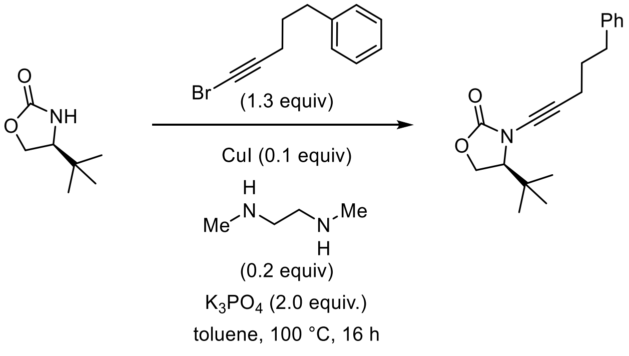

Formula: $C_{18}H_{23}NO_2$

MW = 285.4 g/mol

Bromoalkyne was prepared in 83% yield in a scale of 20 mmol according to a procedure described in the literature.[2]

A flame-dried Schlenk flask under argon atmosphere was loaded with bromoalkyne (580 mg, 2.6 mmol, 1.3 equiv.), (*S*)-4-*tert*-butyl-2-oxazolidinone (286 mg, 2.00 mmol, 1.0 equiv.), anhydrous potassium phosphate (849 mg, 4 mmol, 2.0 equiv.), copper(I) iodide (38.3 mg, 0.2 mmol, 0.1 equiv.), *N*,*N*'-dimethylethylenediamine (35.3 mg, 0.40 mmol, 0.2 equiv.). Toluene (10 mL) was added and the mixture was heated to 100 °C for 16 hours. After this time and cooling to rt, the mixture was passed through a short Celite® plug using EtOAc as eluent. After evaporation of solvent, the crude material was purified by flash column chromatography on silica gel (heptane to heptane/EtOAc (7:3)), providing the desired compound as colorless oil (377 mg, 1.32 mmol, 66%).

^{1}H NMR (600 MHz, $CDCl_3$): δ 7.28 (t, *J* = 7.6 Hz, 2H), 7.22 – 7.14 (m, 3H), 4.34 (t, *J* = 9.0 Hz, 1H), 4.19 (dd, *J* = 9.2, 5.3 Hz, 1H), 3.72 (dd, *J* = 8.7, 5.3 Hz, 1H), 2.72 (t, *J* = 7.7 Hz, 2H), 2.34 (t, *J* = 7.0 Hz, 2H), 1.89 – 1.68 (m, 2H), 1.06 (s, 9H);

^{13}C NMR (151 MHz, $CDCl_3$): δ 157.5, 141.7, 128.7 (2C), 128.5 (2C), 126.1, 72.1, 71.6, 66.1, 65.4, 35.1, 34.9, 30.5, 25.5 (3C), 18.2;

FTIR (neat): 2961, 1766, 1405, 1187, 1113, 749, 699 cm^{-1};

HRMS (ESI): Calculated for ($C_{18}H_{23}NO_2Na^+$) $[M+Na]^+$: 308.1621, Found: 308.1618;

$[\alpha]^{20}_{D}$ = +4.0 (c = 0.82, $CHCl_3$).

2.3.2 Results of [3,3]-sigmatropic rearrangement

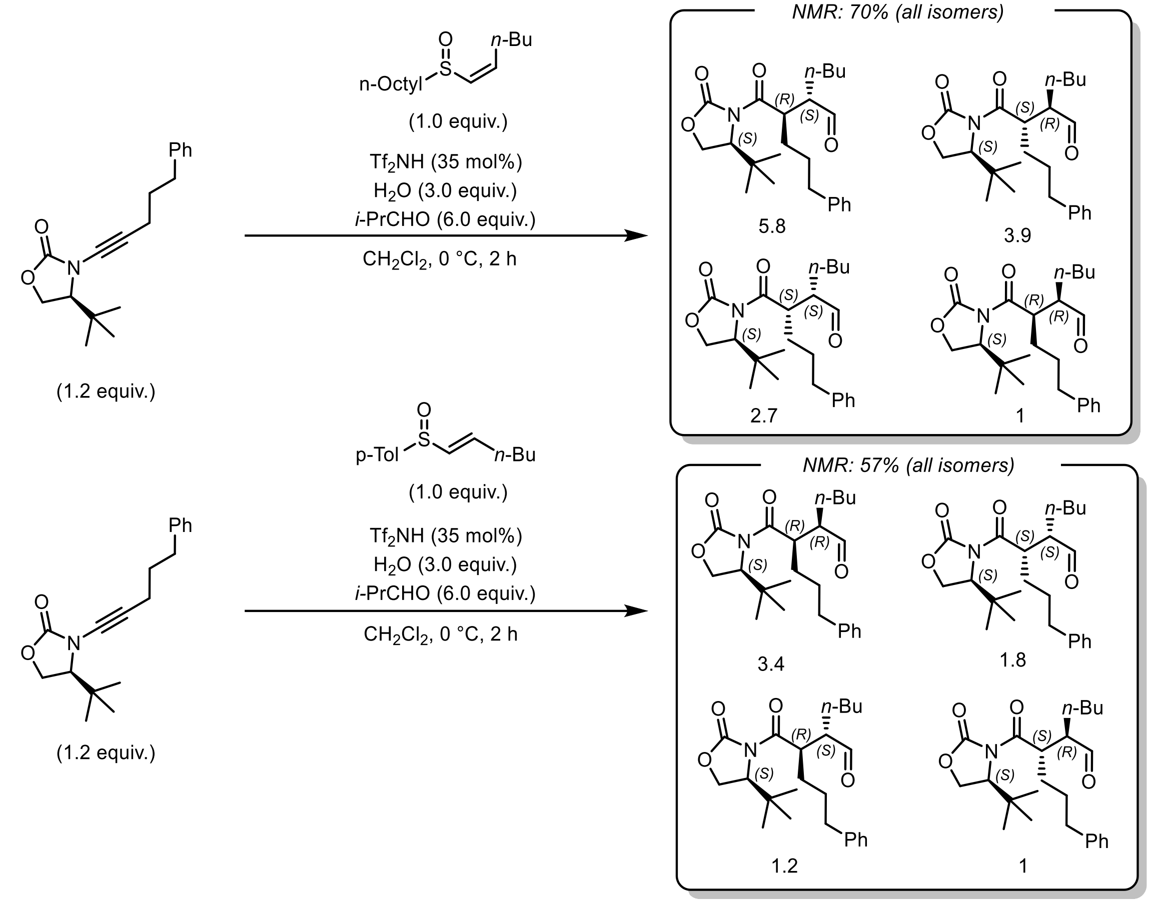

Following general procedure A (0.1 mmol scale), using **S1** and the corresponding vinyl sulfoxide (either *E* or *Z*). The crude materials were analyzed by NMR (^{13}C and ^{1}H). 4 isomers of the 1,4-dicarbonyl compound were detected. To shed light on the isomeric distribution (as shown in the scheme below), the crude material had to be subjected to a reductive cyclization. Hereby, the crude reaction mixture was diluted with MeOH (1 mL) and $NaBH_4$ (1 mmol, 37.8 mg, 10 equiv.) was added at 0 °C. The reaction mixture was stirred at this temperature for 1 h before aq. sol. of HCl (1 M) was added. The phases were separated and the aqueous phase was extracted with CH_2Cl_2. The combined organic phases were dried over $mgSO_4$, filtered and concentrated *in vacuo*. The crude product was purified by flash column chromatography on silica gel to afford the desired lactone as mixture of 2 inseparable diastereoisomers which were further analyzed by chiral HPLC to determine enantiomeric enrichment. Based on this result, we were able to determine the stereochemistry for each 1,4-dicarbonyl compounds.

Assignment of each diasteroisomer via reductive cyclization:

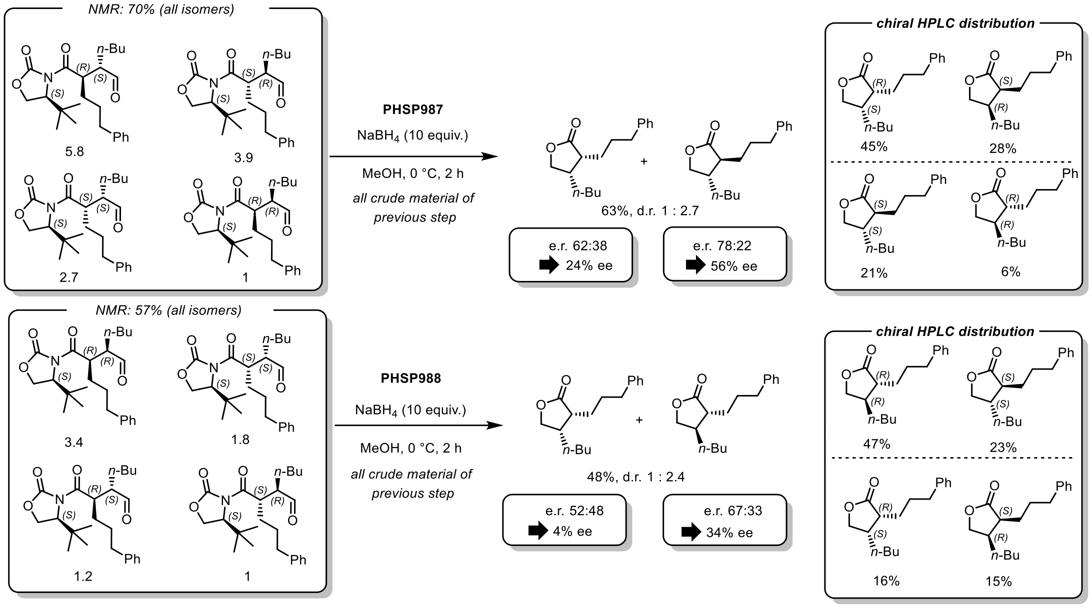

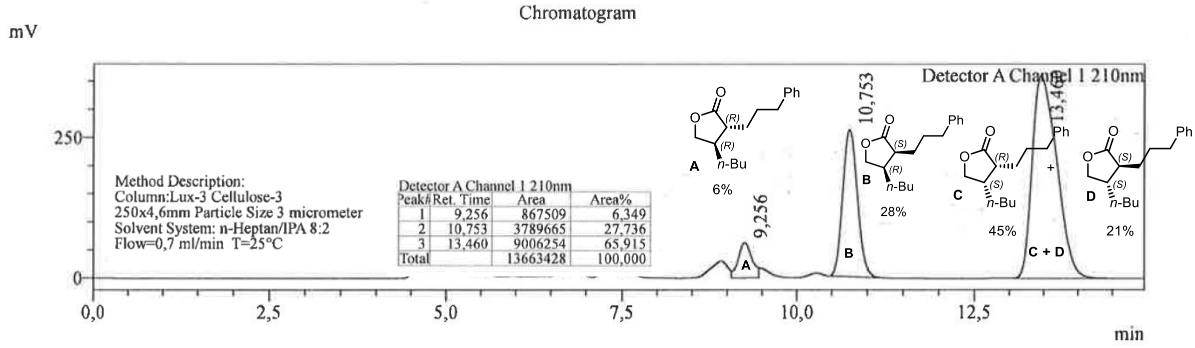

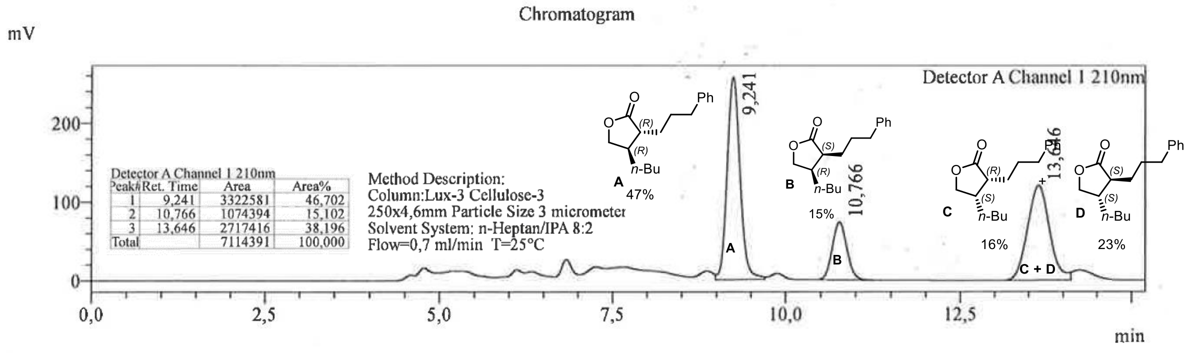

The absolute configuration of each isomer was determined by comparison with our previous work.[1] Since the peaks of the isomers C and D overlapped, the d.r. determined by NMR was used to back-calculate the ratio of each isomer.

2.4 Preparation of γ-C lactones

2.4.1 General Procedure B: Formation of γ-C lactones

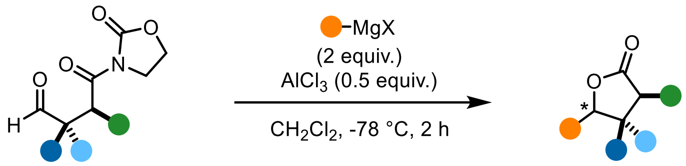

To a -78 °C solution of *aldehyde* (1 equiv.) in CH_2Cl_2 (0.1 M) was added $AlCl_3$ (0.5 equiv.) followed by addition of a solution of the Grignard reagent (2 equiv.) dropwise and the mixture was left stirring at -78 °C for 2 h. A sat. aq. solution of NH_4Cl (*ca* 2 mL for a 0.1 mmol scale) was added followed by a sat. aq. solution of Rochelle's salt (*ca* 2 mL for a 0.1 mmol scale) and the mixture was left stirring at rt for 10 min. The mixture was then filtrated over Celite® rinsing with CH_2Cl_2 and water. The phases were separated and the aqueous phase was extracted with CH_2Cl_2 (3x). The combined organic phases were washed with brine, dried over $mgSO_4$, filtered and concentrated *in vacuo*. The crude product was subjected to flash column chromatography on silica gel (EtOAc/Heptane, usually 5% to 30% in 25 cv) to afford the corresponding lactone as mixture of diastereoisomers.

2.4.2 Optimization results

The following results were particularly important in our optimization that we want to highlight:

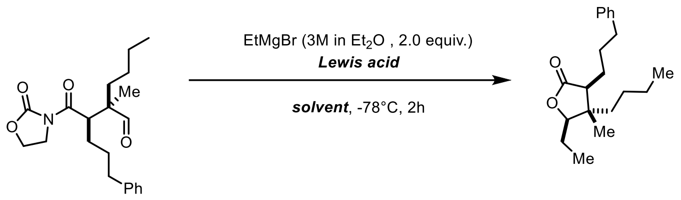

tested conditions	$AlCl_3$ (50 mol%)<br>DCM (0.1M)	$AlCl_3$ (50 mol%)<br>THF (0.1M)	NO Lewis acid<br>DCM (0.1M)
result	NMR: **84%**<br>d.r. 2:3:1<br>70 : 30<br>*above isomer major isomer*	NMR: **14% of**<br>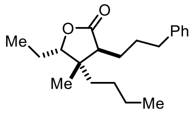<br>as major diastereoisomer	NMR: **70%**<br>d.r. 2:3:1<br>70 : 30<br>*above isomer major isomer*

2.4.3 Explanations for stereochemical outcomes

Explanation for *trans*-addtion favored over *cis*-addtion

illustrative example **11a:**

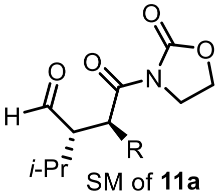
SM of **11a**

R = $(CH_2)_3$-Ph
shown for this enantiomer

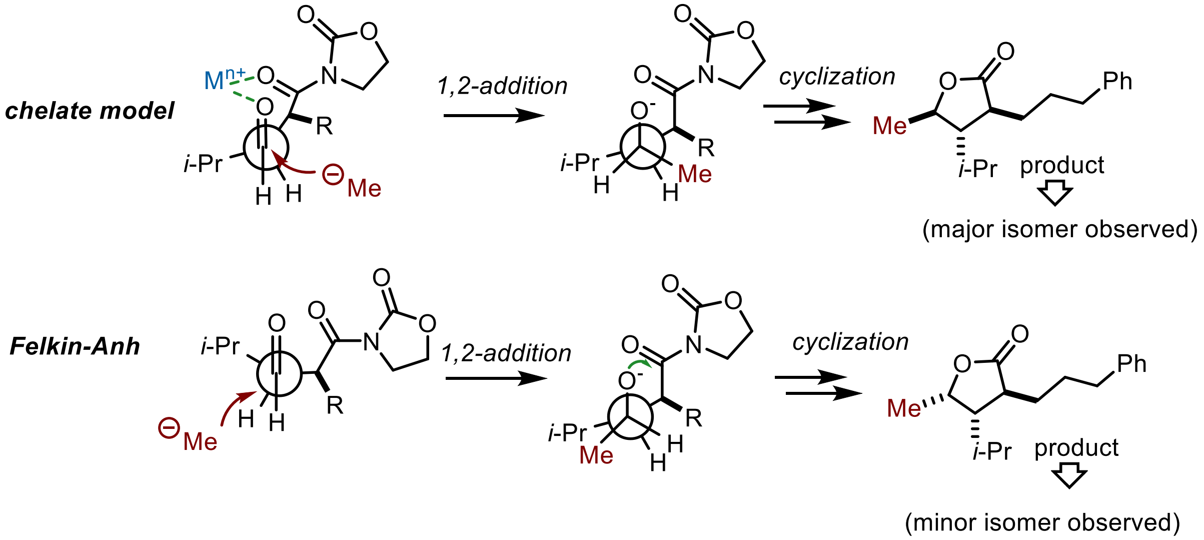

examples **11j** & **11k:**

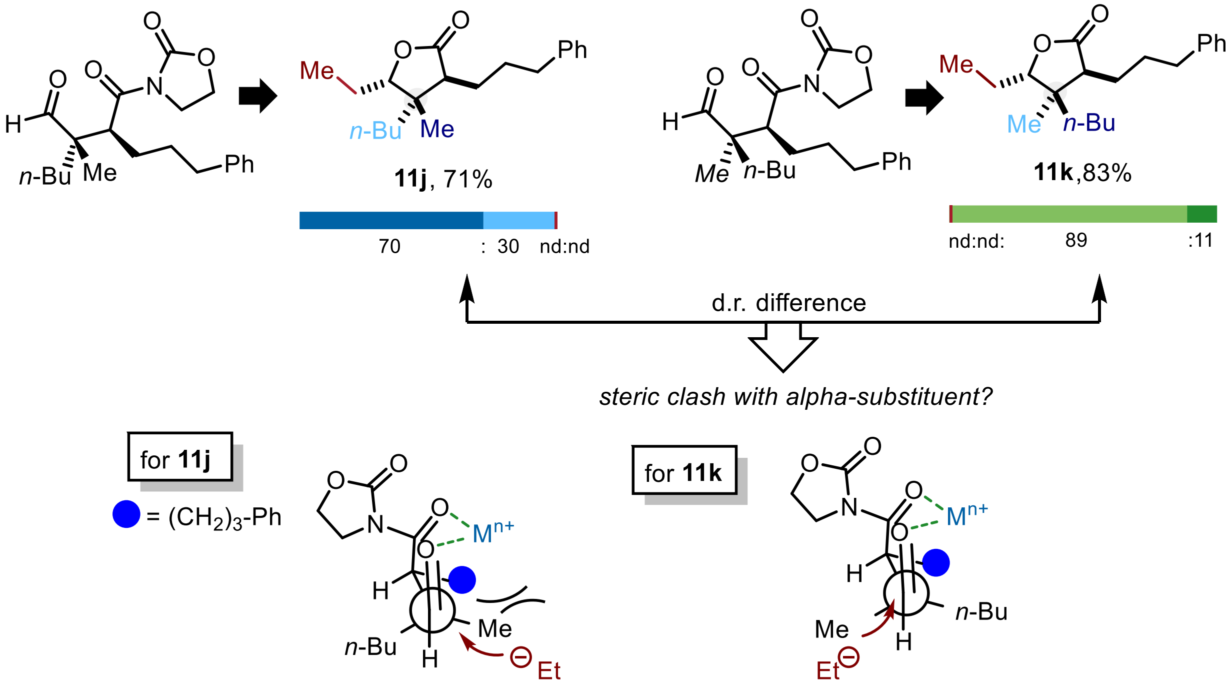

2.4.4 General Procedure C: Formation of γ-allyl lactones

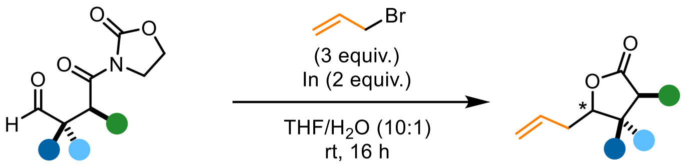

To a rt solution of *aldehyde* (1 equiv.) in a THF/H_2O mixture (10:1, 0.1 M) was added allyl bromide (3 equiv.) and solid indium beads (2 equiv.) and the reaction mixture was stirred at rt for 16 h. The mixture was extracted with EtOAc and the combined organic extracts were dried over $mgSO_4$, filtered and concentrated *in vacuo*. The crude product was purified by flash column chromatography on silica gel (EtOAc/Heptane, usually 5% to 30% in 25 cv) to afford the corresponding lactone as mixture of diastereoisomers.

2.4.5 Characterization of products

(±)-(3*S*,4*S*,5*R*)-4-Isopropyl-5-methyl-3-(3-phenylpropyl)dihydrofuran-2(3*H*)-one (11a)

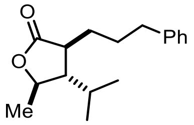

Formula: $C_{17}H_{24}O_2$

MW = 260.4 g/mol

11a was prepared from (±)-(2*S*,3*S*)-2-isopropyl-3-(2-oxooxazolidine-3-carbonyl)-6-phenylhexanal (33 mg, 0.10 mmol, 1 equiv., d.r. = 88:12) and methylmagnesium bromide (2.53 M in Et_2O, 79 µL, 0.20 mmol, 2 equiv.) following the general procedure **B**. The crude product (d.r. = 81:17:2:nd) was purified by flash column chromatography on silica gel (EtOAc/heptane, 5% to 30%) to afford **11a** (24.3 mg, 93.3 µmol, 93%, d.r. = 79:16:5:nd) as a colorless oil.

Data are reported for the major diastereoisomer.

^{1}H NMR (600 MHz, $CDCl_3$): δ = 7.30 – 7.25 (m, 2H), 7.21 – 7.16 (m, 3H), 4.28 (quint, *J* = 6.3 Hz, 1H), 2.71 (m, 2H), 2.43 (dd, *J* = 13.6, 5.9 Hz, 1H), 1.90 – 1.65 (m, 6H), 1.38 (d, *J* = 6.3 Hz, 3H), 0.93 (d, *J* = 6.9 H, 6H);

^{13}C NMR (151 MHz, $CDCl_3$): δ = 178.9, 141.8, 128.34 (2C), 128.32 (2C), 125.8, 77.9, 53.1, 43.7, 35.8, 31.0, 30.1, 28.2, 22.4, 20.0, 19.7;

FTIR (neat): 2961, 2932, 2873, 1766, 1496, 1467, 1453, 1389, 1190, 1048, 749, 699 cm^{-1};

HRMS (ESI): Calculated for $C_{17}H_{24}O_2Na^+$ $[M+Na]^+$: 283.1669, Found: 283.1670;

(±)-(3*S*,4*S*,5*R*)-5-Ethyl-4-isopropyl-3-(3-phenylpropyl)dihydrofuran-2(3*H*)-one (11b)

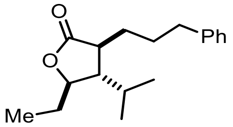

Formula: $C_{18}H_{26}O_2$

MW = 274.4 g/mol

11b was prepared from (±)-(2*S*,3*S*)-2-isopropyl-3-(2-oxooxazolidine-3-carbonyl)-6-phenylhexanal (33 mg, 0.10 mmol, 1 equiv., d.r. = 88:12) and ethylmagnesium bromide (2.12 M in Et_2O, 94 µL, 0.20 mmol, 2 equiv.) following the general procedure **B**. The crude product (d.r. = 83:11:4:2) was purified by flash column chromatography on silica gel (EtOAc/heptane, 5% to 30%) to afford **11b** (28 mg, 0.10 mmol, quant., d.r. = 83:11:4:2) as a colorless oil.

Data are reported for the major diastereoisomer.

^{1}H NMR (600 MHz, $CDCl_3$): δ = 7.31 – 7.25 (m, 2H), 7.21 – 7.16 (m, 3H), 4.08 (dt, *J* = 8.7, 4.4 Hz, 1H), 2.72 – 2.57 (m, 2H), 2.40 (q, *J* = 6.2 Hz, 1H), 1.88 – 1.49 (m, 8H), 1.01 (t, *J* = 7.2 Hz, 3H), 0.92 (d, *J* = 6.0 Hz, 6H);

^{13}C NMR (151 MHz, $CDCl_3$): δ = 179.4, 141.9, 128.49 (2C), 128.48 (2C), 126.0, 83.4, 50.9, 46.9, 35.9, 31.7, 30.8, 30.1, 28.6, 19.81, 19.77, 10.3;

FTIR (neat): 2963, 2936, 2876, 1763, 1496, 1456, 1364, 1189, 971, 749, 699 cm^{-1};

HRMS (ESI): Calculated for $C_{18}H_{26}O_2Na^+$ $[M+Na]^+$: 297.1825, Found: 297.1828;

(±)-(3*S*,4*S*,5*R*)- 4-Isopropyl-5-phenethyl-3-(3-phenylpropyl)dihydrofuran-2(3*H*)-one (11c)

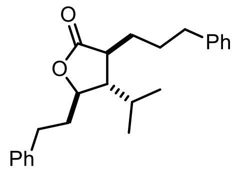

Formula: $C_{24}H_{30}O_2$

MW = 350.5 g/mol

11c was prepared from (±)-(2*S*,3*S*)-2-isopropyl-3-(2-oxooxazolidine-3-carbonyl)-6-phenylhexanal (24.9 mg, 75 µmol, 1 equiv., d.r. = 88:12) and phenethylmagnesium chloride (0.95 M in THF, 0.20 mL, 0.15 mmol, 2 equiv.) following the general procedure **B**. The crude product (d.r. = 75:12:7:6) was purified by flash column chromatography on silica gel (EtOAc/heptane, 5% to 30%) to afford **11c** (16.7 mg, 47.6 µmol, 63%, d.r. = 82:9:5:4) as a colorless oil.

Data are reported for the major diastereoisomer.

^{1}H NMR (600 MHz, $CDCl_3$): δ = 7.33 (m, 4H), 1.23 – 7.15 (m, 6H), 4.13 (ddd, *J* = 9.3, 5.7, 3.5 Hz, 1H), 2.88 (ddd, *J* = 13.7, 9.7, 4.8 Hz, 1H), 2.73 – 2.60 (m, 3H), 2.41 (q, *J* = 6.3 Hz, 1H), 1.96 – 1.65 (m, 8H), 0.88 (d, *J* = 6.6 Hz, 3H), 0.86 (d, *J* = 6.5 Hz, 3H);

^{13}C NMR (151 MHz, $CDCl_3$): δ = 179.2, 141.9, 141.1, 128.65 (2C), 128.60 (2C), 128.51 (4C), 126.3, 126.0, 81.0, 51.4, 43.5, 39.1, 35.9, 32.3, 31.5, 30.7, 28.5, 19.87, 19.85;

FTIR (neat): 3026, 2960, 2929, 2859, 1764, 1496, 1453, 1391, 1369, 1192, 1151, 1029, 979, 939 cm^{-1};

HRMS (ESI): Calculated for $C_{24}H_{30}O_2Na^+$ $[M+Na]^+$: 373.2138, Found: 373.2136;

Selected *nOe* correlation:

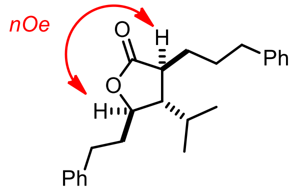

(±)-(3*S*,4*S*,5*R*)-4-Isopropyl-3-(3-phenylpropyl)-5-vinyldihydrofuran-2(3*H*)-one (11d)

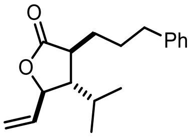

Formula: $C_{18}H_{24}O_2$

MW = 272.4 g/mol

11d was prepared from (±)-(2*S*,3*S*)-2-isopropyl-3-(2-oxooxazolidine-3-carbonyl)-6-phenylhexanal (33 mg, 0.10 mmol, 1 equiv., d.r. = 88:12) and vinylmagnesium bromide (0.75 M in THF, 0.27 mL, 0.20 mmol, 2 equiv.) following the general procedure **B**. The crude product (d.r. = 77:12:11:nd) was purified by flash column chromatography on silica gel (EtOAc/heptane, 5% to 30%) to afford **11d** (18.1 mg, 66.4 µmol, 66%, fraction 1 (6.2 mg): d.r. = 18:39:43:nd, fraction 2 (11.9 mg), d.r. = 88:9:3:nd) as colorless oils.

Data are reported for the major diastereoisomer.

^{1}H NMR (700 MHz, CDCl$_3$): δ = 7.30 – 7.26 (m, 2H), 7.20 – 7.16 (m, 3H), 5.81 (ddd, *J* = 17.0, 10.5, 6.4 Hz, 1H), 5.34 (dt, *J* = 17.0, 1.0 Hz, 1H), 5.22 (dt, *J* = 10.5, 1.0 Hz, 1H), 4.58 (tt, *J* = 6.5, 1.0 Hz, 1H), 2.71 – 2.58 (m, 2H), 2.42 (q_{app}, *J* = 6.6 Hz, 1H), 1.89 – 1.66 (m, 6H), 0.95 (d, *J* = 6.6 Hz, 3H), 0.94 (d, *J* = 6.6 Hz, 3H);

^{13}C NMR (176 MHz, CDCl$_3$): δ = 178.9, 141.9, 137.0, 128.51 (2C), 128.50 (2C), 126.0, 117.6, 81.7, 51.8, 43.1, 35.9, 30.9, 30.1, 28.4, 19.8 (2C);

FTIR (neat): 3026, 2961, 2930, 2874, 1766, 1495, 1466, 1187, 1148, 1029, 983, 932, 749, 699 cm^{-1};

HRMS (ESI): Calculated for $C_{18}H_{24}O_2Na^+$ $[M+Na]^+$: 295.1669, Found: 295.1669.

Selected *nOe* correlations:

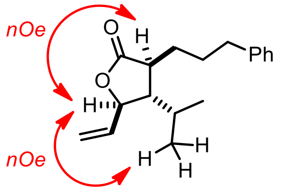

(±)-(3*S*,4*S*,5*S*)-4-Butyl-3-(3-phenylpropyl)-5-[(trimethylsilyl)ethynyl]dihydrofuran-2(3*H*)-one (11e)

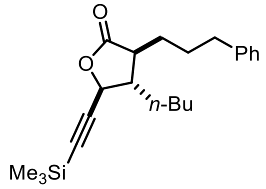

Formula: $C_{22}H_{32}O_2Si$

MW = 356.6 g/mol

[(Trimethylsilyl)ethynyl]magnesium bromide was prepared following a known procedure.[3] To a 0 °C solution of trimethylsilylacetylene (0.34 mL, 2.4 mmol, 1.2 equiv.) in THF (1 mL) was added ethylmagnesium bromide (2.12 M in Et_2O, 0.94 mL, 2 mmol, 1 equiv.) dropwise and the mixture was left stirring at the same temperature for 1 h. The reaction was considered to be quantitative and the concentration of the solution was considered to be 0.9 M.

11e was prepared from (±)-(2*S*,3*S*)-2-butyl-3-(2-oxooxazolidine-3-carbonyl)-6-phenylhexanal (34.5 mg, 0.10 mmol, 1 equiv., d.r. = 86:14) and [(Trimethylsilyl)ethynyl]magnesium bromide (0.9 M in Et_2O/THF, 0.22 mL, 0.2 mmol, 2 equiv.) following the general procedure **B**. The crude product (d.r. = 76:14:10:nd) was purified by flash column chromatography on silica gel (EtOAc/heptane, 5% to 30%) to afford **11e** (21.8 mg, 61.1 µmol, 61%, d.r. = 79:13:8:nd) as a colorless oil.

Data are reported for the major diastereoisomer.

^{1}H NMR (600 MHz, CDCl$_3$): δ = 7.30 – 7.25 (m, 2H), 7.21 – 7.16 (m, 3H), 5.07 (d, *J* = 7.6 Hz, 1H), 2.73 – 2.59 (m, 2H), 2.34 (m, 1H), 2.25 (m, 1H), 1.86 (m, 1H), 1.80 – 1.54 (m, 5H), 1.39 – 1.15 (m, 4H), 0.92 (t, *J* = 7.1 Hz, 3H), 0.18 (s, 9H);

^{13}C NMR (151 MHz, CDCl$_3$): δ = 178.1, 141.9, 128.51 (3C), 128.49 (2C), 126.0, 98.3, 95.2, 71.3, 44.3, 43.7, 36.0, 29.7, 29.3, 27.8, 22.9, 14.0, -0.23 (3C);

FTIR (neat): 2957, 2931, 2860, 1781, 1454, 1342, 1250, 1138, 987, 841, 759, 633 cm^{-1};

HRMS (ESI): Calculated for $C_{22}H_{32}O_2SiNa^+$ $[M+Na]^+$: 379.2064, Found: 379.2064.

(±)-(3*S*,4*S*,5*R*)-4-Isopropyl-5-(4-methoxyphenyl)-3-(3-phenylpropyl)dihydrofuran-2(3*H*)-one (11f)

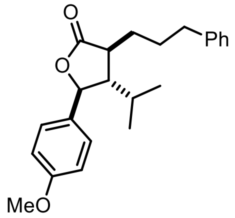

Formula: $C_{23}H_{28}O_3$

MW = 352.5 g/mol

11f was prepared from (±)-(2*S*,3*S*)-2-isopropyl-3-(2-oxooxazolidine-3-carbonyl)-6-phenylhexanal (33 mg, 0.10 mmol, 1 equiv., d.r. = 88:12) and 4-methoxyphenylmagnesium bromide (0.82 M in THF, 0.24 mL, 0.2 mmol, 2 equiv.) following the general procedure **B**. The crude product (d.r. = 79:11:10:nd) was purified by flash column chromatography on silica gel (EtOAc/heptane, 5% to 30%) to afford **11f** (29.1 mg, 82.6 µmol, 83%, fraction 1 (6.3 mg): d.r. = 35:37:28:nd, fraction 2 (22.8 mg), d.r. = 96:2:2:nd) as colorless oils.

Data are reported for the major diastereoisomer.

^{1}H NMR (600 MHz, CDCl$_3$): δ = 7.30 – 7.25 (m, 2H), 7.23 – 7.15 (m, 5H), 6.88 (d, *J* = 8.8 Hz, 2H), 5.06 (d, *J* = 7.3 Hz, 1H), 3.81 (s, 3H), 2.71 – 5.59 (m, 2H), 2.54 (dt, *J* = 8.7, 5.8 Hz, 1H), 2.18 (m, 1H), 1.92 – 1.80 (m, 2H), 1.79 – 1.96 (m, 3H), 0.93 (d, *J* = 6.9 Hz, 3H), 0.90 (d, *J* = 6.9 Hz, 3H);

^{13}C NMR (151 MHz, CDCl$_3$): δ = 178.9, 159.8, 141.9, 132.0, 128.51 (2C), 128.48 (2C), 127.9 (2C), 126.0, 114.2 (2C), 82.4, 55.4, 53.9, 43.4, 35.9, 30.4, 29.4, 28.3, 20.1, 19.7;

FTIR (neat): 2960, 2932, 1765, 1613, 1514, 1465, 1298, 1249, 1171, 1032, 978, 833, 773, 699 cm^{-1};

HRMS (ESI): Calculated for $C_{23}H_{28}O_3Na^+$ $[M+Na]^+$: 375.1931, Found: 375.1931.

Selected *nOe* correlation:

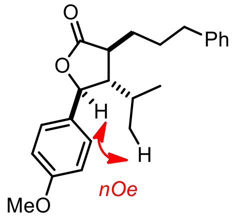

(±)-(3*S*,4*R*,5*S*)-4-Butyl-5-methyl-3-(3-phenylpropyl)dihydrofuran-2(3*H*)-one (11g)

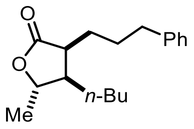

Formula: $C_{18}H_{26}O_2$

MW = 274.4 g/mol

11g was prepared from (±)-(2*R*,3*S*)-2-butyl-3-(2-oxooxazolidine-3-carbonyl)-6-phenylhexanal (34.5 mg, 100 µmol, 1 equiv., d.r. = 22:78) and methylmagnesium bromide (1.72 M in Et_2O, 0.12 mL, 0.20 mmol, 2 equiv.) following the general procedure **B**. The crude product (d.r. = 8:13:76:6) was purified by flash column chromatography on silica gel (EtOAc/heptane, 5% to 30%) to **11g** (17 mg, 61.9 mmol, 62%, fraction 1 (15.1 mg), d.r. = 9:14:76:1, fraction 2 (1.9 mg), d.r. = nd:nd:nd:100) as colorless oils.

Data are reported for the major diastereoisomer.

^{1}H NMR (700 MHz, $CDCl_3$): δ =7.30 – 7.26 (m, 2H), 7.20 – 7.15 (m, 3H), 4.28 (p_{app}, *J* = 6.3 Hz, 1H), 2.67 (t, *J* = 7.4 Hz, 2H), 2.62 (dd, *J* = 15.1, 7.7 Hz, 1H), 2.07 – 1.99 (m, 1H), 1.91 – 1.84 (m, 1H), 1.76 – 1.61 (m, 2H), 1.55 – 1.48 (m, 1H), 1.34 (d, *J* = 6.3 Hz, 3H), 1.32 – 1.18 (m, 6H), 0.88 (t, *J* = 7.1 Hz, 3H);

^{13}C NMR (176 MHz, $CDCl_3$): δ = 178.6, 141.9, 128.5 (2C), 128.5 (2C), 126.0, 79.4, 45.7, 42.2, 35.8, 29.7, 29.1, 26.6, 24.6, 22.9, 19.9, 14.0;

FTIR (neat): 2957, 2929, 2859, 1770, 1496, 1454, 1187, 958, 747 cm^{-1};

HRMS (ESI): Calculated for $C_{18}H_{26}O_2H^+$ $[M+H]^+$: 275.2006, Found: 275.2004.

(±)-(3*S*,4*R*,5*S*)-4-Butyl-5-ethyl-3-(3-phenylpropyl)dihydrofuran-2(3*H*)-one (11h)

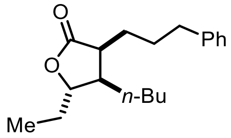

Formula: $C_{19}H_{28}O_2$

MW = 288.4 g/mol

11h was prepared from (±)- (2*R*,3*S*)-2-butyl-3-(2-oxooxazolidine-3-carbonyl)-6-phenylhexanal (17.3 mg, 50.0 µmol, 1 equiv.) and ethylmagnesium bromide (3 M in Et_2O, 33 µL, 0.10 mmol, 2 equiv.) following the general procedure **B**. The crude product (d.r. = 9:2:83:6) was purified by flash column chromatography on silica gel (EtOAc/heptane, 5% to 30%) to afford **11h** (9.4 mg, 33 µmol, 65%, d.r. = 9:4:76:11) as a colorless oil.

Data are reported for the major diastereoisomer.

^{1}H NMR (700 MHz, $CDCl_3$): δ = 7.29 – 7.25 (m, 2H), 7.20 – 7.16 (m, 3H), 4.06 (m, 1H), 2.71 – 2.62 (m, 2H), 2.59 (q, *J* = 7.6 Hz, 1H), 2.09 (m, 1H), 1.84 (m, 1H), 1.77 – 1.65 (m, 2H), 1.65 – 1.59 (m, 2H), 1.55 – 1.44 (m, 1H), 1.35 – 1.14 (m, 6H), 0.99 (t, *J* = 7.4 Hz, 3H), 0.88 (t, *J* = 7.2 Hz, 3H)

^{13}C NMR (176 MHz, $CDCl_3$): δ = 178.8, 141.9, 128.55 (2C), 128.52 (2C), 126.0, 84.6, 42.9, 42.2, 35.9, 29.6, 29.3, 27.1, 26.9, 24.7, 23.1, 14.0, 10.3;

FTIR (neat): 2931, 2860, 1768, 1455, 1186, 967, 746, 700 cm^{-1};

HRMS (ESI): Calculated for: $C_{19}H_{28}O_2H^+$ $[M+H]^+$: 289.2162, Found: 289.2162.

(±)-(3*S*,4*R*,5*R*)-4-Butyl-3-(3-phenylpropyl)-5-[4-(trifluoromethyl)phenyl]dihydrofuran-2(3*H*)-one (11i)

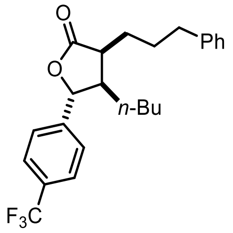

Formula: $C_{24}H_{27}F_3O_2$

MW = 404.5 g/mol

Magnesium turnings (255 mg, 10.5 mmol, 1.5 equiv.) were placed in a dry flask and covered by a small amount of dry degassed THF (degassed by sparging with argon for 30 min prior to use) without stirring. Only a few drops of 1-bromo-4-(trifluoromethyl)benzene (overall 1.0 mL, 7.0 mmol, 1 equiv.) were added slowly without stirring followed by a crystal of iodine. The mixture was carefully stirred (1 or 2 rotations of the stirring bar at a time) and warmed with the palm of the hand until the reaction began (noticed by disappearance of the iodine coloration and an exothermic reaction). At this point the rest of the bromide was dissolved in THF (10 mL) and the resulting solution was added dropwise so as the exothermic reaction is maintained. The resulting mixture is left stirring at rt for 1 h and is then decanted and transferred to a dry flask by the mean of a canula. The resulting red solution of [4-(trifluoromethyl)phenyl]magnesium bromide was titrated with Love's reagent (V = 8 mL, c = 0.71 M, 5.7 mmol, 81%).[4]

11i was prepared from (±)-(2*R*,3*S*)-2-butyl-3-(2-oxooxazolidine-3-carbonyl)-6-phenylhexanal (24.2 mg, 70.0 µmol, 1 equiv., d.r. = 22:78) and [4-(trifluoromethyl)phenyl]magnesium bromide (0.63 M, 0.22 mL, 0.14 mmol, 2 equiv.) following the general procedure **B**. The crude product (d.r. = 8:10:76:6) was purified by flash column chromatography on silica gel (EtOAc/heptane, 5% to 30%) to afford **11i** (11.2 mg, 27.7 µmol, 40%, fraction 1 (9.0 mg), d.r. = nd:12:88:nd, fraction 2 (2.2 mg), d.r. = 72:nd:nd:28) as colorless oils.

Data are reported for the major diastereoisomer.

^{1}H NMR (700 MHz, CDCl$_3$): δ = 7.64 (d, *J* = 8.1 Hz, 2H), 7.39 (d, *J* = 8.8 Hz, 2H), 7.28 – 7.25 (m, 2H), 7.22 – 7.15 (m, 3H), 5.20 (d, *J* = 5.1 Hz, 1H), 2.67 (t, *J* = 7.6 Hz, 2H), 2.59 (q, *J* = 7.6 Hz, 1H), 2.34 (m, 1H), 1.86 (m, 1H), 1.79 – 1.65 (m, 2H), 1.57 (m, 1H), 1.45 (m, 1H), 1.39 – 1.23 (m, 5H), 0.89 (t, *J* = 7.1 Hz, 3H);

^{13}C NMR (176 MHz, CDCl$_3$): δ = 178.1, 143.1, 141.7, 130.7 (q, *J* = 31.6 Hz), 128.5 (m, 4C), 125.8 (m, 5C), 123.9 (q, *J* = 272.1 Hz), 82.3, 47.4, 41.4, 35.6, 29.5, 28.8, 26.2, 24.2, 22.6, 13.9;

^{19}F NMR (659 MHz, CDCl$_3$): δ = -62.6 (3F);

FTIR (neat): 2960, 2931, 2859, 1777, 1325, 1166, 1126, 1068, 1015, 841, 772, 749, 699, 669 cm^{-1};

HRMS (ESI): Calculated for $C_{24}H_{27}F_3O_2Na^+$ [M+Na]$^+$: 427.1855, Found: 427.1851.

(±)-(3*S*,4*S*,5*R*)-4-Butyl-5-ethyl-4-methyl-3-(3-phenylpropyl)dihydrofuran-2(3*H*)-one (11j)

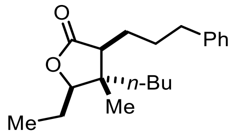

Formula: $C_{20}H_{30}O_2$

MW = 302.4 g/mol

11j was prepared from (±)-(2*S*,3*S*)-2-butyl-2-methyl-3-(2-oxooxazolidine-3-carbonyl)-6-phenylhexanal (18.0 mg, 50.0 µmol, 1 equiv) and ethylmagnesium bromide (3 M in Et$_2$O, 33 µL, 0.10 mmol, 2 equiv.) following the general procedure **B**. The crude product (d.r. = 70:30) was purified by flash column chromatography on silica gel (EtOAc/heptane, 0% to 20%) to afford **11j** (10.8 mg, 36 µmol, 71%, d.r. = 70:30) as a colorless oil.

Data are reported for the both diastereoisomers.

^{1}H NMR (400 MHz, CDCl$_3$): δ 7.31 – 7.26 (m, 2H), 7.22 – 7.16 (m, 3H), 3.98 – 3.91 (m, 1H), 2.71 – 2.62 (m, 2H), 2.33 (dd, *J* = 8.6, 4.2 Hz, 0.3H), 2.25 (dd, *J* = 9.3, 4.7 Hz, 0.7H), 2.15 – 2.00 (m, 1H), 1.75 – 1.18 (m, 11H), 1.10 – 1.02 (m, 3H), 0.96 (s, 2H), 0.91 (t, *J* = 7.1 Hz, 3H), 0.79 (s, 1H).

^{13}C NMR (101 MHz, CDCl$_3$): δ 179.0, 178.7, 142.1, 142.0, 128.6, 128.5 (2C), 125.9, 89.3, 87.2, 49.8, 48.5, 45.6, 44.6, 37.2, 36.1, 36.0, 34.9, 29.8, 29.6, 26.7, 26.4, 25.3, 24.8, 23.6, 23.5, 22.7, 19.5, 15.4, 14.10 (2C), 11.7, 11.3;

FTIR (neat): 2957, 2932, 2861, 1765, 1455, 968, 745, 699 cm^{-1};

HRMS (ESI): Calculated for $C_{20}H_{30}O_2Na^+$ [M+Na]$^+$: 325.2138, Found: 325.2136.

(±)-(3*S*,4*R*,5*R*)-4-Butyl-5-ethyl-4-methyl-3-(3-phenylpropyl)dihydrofuran-2(3*H*)-one (11k)

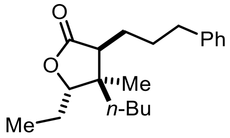

Formula: $C_{20}H_{30}O_2$

MW = 302.5 g/mol

11k was prepared from (2*R*,3*S*)-2-butyl-2-methyl-3-(2-oxooxazolidine-3-carbonyl)-6-phenylhexanal (18.0 mg, 50.0 µmol, 1 equiv., d.r. < 5:95) and ethylmagnesium bromide (3 M in Et_2O, 33 µL, 0.10 mmol, 2 equiv.) following the general procedure **B**. The crude product (d.r. = nd:nd:89:11) was purified by flash column chromatography on silica gel (EtOAc/heptane, 5% to 30%) to afford **11k** (12.5 mg, 41.3 µmol, 83%, d.r. = nd:nd:91:9) as a colorless oil.

Data are reported for the major diastereoisomer.

^{1}H NMR (700 MHz, $CDCl_3$): δ = 7.30 – 7.26 (m, 2H), 7.20 – 7.16 (m, 3H), 4.05 – 3.99 (m, 1H), 2.73 – 2.61 (m, 2H), 2.20 (dd, *J* = 9.7, 4.9 Hz, 1H), 2.07 – 1.98 (m, 1H), 1.77 – 1.68 (m, 1H), 1.65 – 1.58 (m, 1H), 1.54 – 1.41 (m, 3H), 1.32 – 1.18 (m, 5H), 1.16 – 1.08 (m, 1H), 1.05 (t, *J* = 7.3 Hz, 3H), 0.98 (s, 3H), 0.87 (t, *J* = 7.3 Hz, 3H);

^{13}C NMR (176 MHz, $CDCl_3$): δ = 178.9, 142.0, 128.6 (2C), 128.5 (2C), 126.0, 87.0, 49.8, 44.4, 35.9, 34.2, 29.3, 26.5, 24.7, 23.5, 23.1, 20.5, 14.1, 11.3;

FTIR (neat): 2960, 2932, 2858, 1766, 1465, 1455, 1345, 1197, 1150, 1138, 1075, 968, 747, 699 cm^{-1};

HRMS (ESI): Calculated for: $C_{20}H_{30}O_2Na^+$ $[M+Na]^+$: 325.2138, Found: 325.2135.

(±)-(3*S*,4*R*,5*S*)-4-Butyl-4-methyl-3-(3-phenylpropyl)-5-vinyldihydrofuran-2(3*H*)-one (11l)

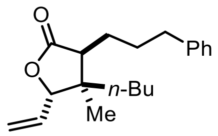

Formula: $C_{20}H_{28}O_2$

MW = 300.4 g/mol

11l was prepared from (±)-(2*R*,3*S*)-2-butyl-2-methyl-3-(2-oxooxazolidine-3-carbonyl)-6-phenylhexanal (35.9 mg, 100 µmol, 1 equiv., d.r. < 5:95) and vinylmagnesium bromide (0.44 M in THF, 0.46 mL, 0.20 mmol, 2 equiv.) following the general procedure **B**. The crude product (d.r. = nd:nd:84:16) was purified by flash column chromatography on silica gel (EtOAc/heptane, 5% to 30%) to afford **11l** (22.0 mg, 70.0 µmol, 70%, fraction 1 (17 mg), d.r. = nd:nd:100:nd, fraction 2 (5 mg), d.r. = nd:nd:59:41) as colorless oils.

Data are reported for the major diastereoisomer.

^{1}H NMR (600 MHz, $CDCl_3$): δ = 7.28 (t, *J* = 7.91 Hz, 2H), 7.20 – 7.16 (m, 3H), 5.80 (ddd, *J* = 17.4, 10.5, 6.3 Hz, 1H), 5.34 (d, *J* = 17.4 Hz, 1H), 5.29 (d, *J* = 10.5 Hz, 1H), 4.57 (d, *J* = 6.3 Hz, 1H), 2.71 – 2.62 (m, 2H), 2.23 (dd, *J* = 9.39, 5.2 Hz, 1H), 2.07 – 1.98 (m, 1H), 1.77 – 1.68 (m, 1H), 1.67 – 1.59 (m, 1H), 1.51 – 1.43 (m, 1H), 1.33 – 1.11 (m, 6H), 0.98 (s, 3H), 0.88 (t, *J* = 7.1 Hz, 3H);

^{13}C NMR (151 MHz, $CDCl_3$): δ = 178.6, 141.9, 132.5, 128.54 (2C), 128.48 (2C), 126.0, 118.5, 85.0, 49.1, 45.2, 35.9, 34.1, 29.3, 26.4, 24.6, 23.4, 21.2, 14.1;

FTIR (neat): 2958, 2932, 2859, 1769, 1603, 1496, 1195, 1157, 1138, 1111, 990, 970, 930, 749, 699 cm^{-1};

HRMS (ESI): Calculated for $C_{20}H_{28}O_2Na^+$ $[M+Na]^+$: 323.1982, Found: 323.1984.

(±)-(3*S*,4*R*,5*R*)-4-Butyl-4-methyl-3-(3-phenylpropyl)-5-[(trimethylsilyl)ethynyl]dihydrofuran-2(3*H*)-one (11m)

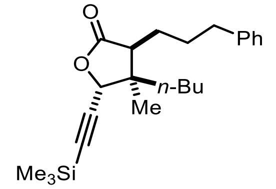

Formula: $C_{23}H_{34}O_2Si$

MW = 370.6 g/mol

[(Trimethylsilyl)ethynyl]magnesium bromide (0.88 M in THF/Et_2O) was prepared as for the synthesis of **11e**.

11m was prepared from (±)-(2*R*,3*S*)-2-butyl-2-methyl-3-(2-oxooxazolidine-3-carbonyl)-6-phenylhexanal (35.9 mg, 100 µmol, 1 equiv., d.r. < 5:95) and [(Trimethylsilyl)ethynyl]magnesium bromide (0.88 M in THF/Et_2O, 0.23 mL, 0.20 mmol, 2 equiv.) following the general procedure **B**. The crude product (d.r. = nd:nd:90:10) was purified by flash column chromatography on silica gel (EtOAc/heptane, 5% to 30%) to afford **11m** (20.0 mg, 54.0 µmol, 54%, only one diastereoisomer) as a colorless oil.

Data are reported for the major diastereoisomer.

^{1}H NMR (700 MHz, $CDCl_3$): δ = 7.30 – 7.25 (m, 2H), 7.21 – 7.16 (m, 3H), 4.79 (s, 1H), 2.72 – 2.63 (m, 2H), 2.40 (dd, *J* = 8.5, 5.3 Hz, 1H), 2.09 – 2.00 (m, 1H), 1.77 – 1.70 (m, 1H), 1.65 – 1.59 (m, 1H), 1.48 – 1.41 (m, 1H), 1.30 – 1.16 (m, 6H), 1.18 (s, 3H), 0.88 (t, *J* = 7.6 Hz, 3H), 0.18 (s, 9H);

^{13}C NMR (176 MHz, $CDCl_3$): δ = 178.0, 141.9, 128.53 (2C), 128.49 (2C), 126.0, 99.3, 95.1, 74.9, 49.1, 45.8, 35.9, 33.5, 29.5, 26.5, 24.4, 23.4, 21.6, 14.1, -0.20 (3C);

FTIR (neat): 2959, 2933, 2860, 1785, 1496, 1455, 1250, 1133, 1032, 984, 856, 761 cm^{-1};

HRMS (ESI): Calculated for $C_{23}H_{34}O_2SiNa^+$ $[M+Na]^+$: 393.2220, Found: 393.2218.

(±)-(3*S*,4*R*,5*R*)-4-Butyl-5-(4-methoxyphenyl)-4-methyl-3-(3-phenylpropyl)dihydrofuran-2(3*H*)-one (11n)

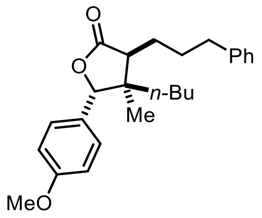

Formula: $C_{25}H_{32}O_3$

MW = 380.5 g/mol

11n was prepared from (±)-(2*R*,3*S*)-2-butyl-2-methyl-3-(2-oxooxazolidine-3-carbonyl)-6-phenylhexanal (35.9 mg, 100 µmol, 1 equiv., d.r. < 5:95) and 4-methoxyphenylmagnesium bromide (0.82 M in THF, 0.24 mL, 0.20 mmol, 2 equiv.) following the general procedure **B**. The crude product (d.r. = nd:nd:80:20) was purified by flash column chromatography on silica gel (EtOAc/heptane, 5% to 30%) to afford **11n** (30.8 mg, 80.9 µmol, 81%, fraction 1 (23.8 mg), d.r. = nd:nd:97:3, fraction 2 (7.0 mg), d.r. = nd:nd:23:77) as colorless oils.

Data are reported for the major diastereoisomer.

^{1}H NMR (600 MHz, CDCl$_3$): δ = 7.28 (t, *J* = 7.7 Hz, 2H), 7.21 – 7.16 (m, 3H), 7.11 (t, *J* = 8.6 Hz, 2H), 6,88 (d, *J* = 8.6 Hz, 2H), 5.16 (s, 1H), 3.81 (s, 3H), 2.73 – 2.63 (m, 2H), 2.34 (dd, *J* = 9.4, 4.6 Hz, 1H), 2.11 – 2.01 (m, 1H), 1.80 – 1.66 (m, 2H), 1.56 – 1.48 (m, 1H), 1.46 – 1.13 (m, 6H), 0.89 (t, *J* = 7.3 Hz, 3H), 0.69 (s, 3H);

^{13}C NMR (151 MHz, CDCl$_3$): δ = 178.9, 159.6, 141.9, 128.54 (2C), 128.48 (2C), 128.1, 127.4 (2C), 126.0, 113.7 (2C), 86.1, 55.4, 49.1, 46.2, 35.8, 34.2, 29.2, 26.5, 24.7, 23.4, 21.7, 14.1;

FTIR (neat): 2958, 2933, 2860, 1770, 1613, 1514, 1456, 1383,1302, 1252, 1178, 1140, 1117, 1034, 1002, 837, 772, 750 cm^{-1};

HRMS (ESI): Calculated for $C_{25}H_{32}O_3Na^+$ $[M+Na]^+$: 403.2244, Found: 403.2242.

(±)-(3*S*,4*R*,5*R*)-4-Butyl-4-methyl-3-(3-phenylpropyl)-5-[4-(trifluoromethyl)phenyl]dihydrofuran-2(3*H*)-one (11o)

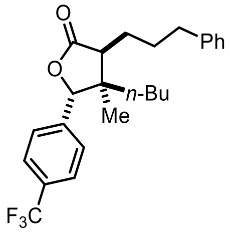

Formula: $C_{25}H_{29}F_3O_2$

MW = 418.5 g/mol

[4-(Trifluoromethyl)phenyl]magnesium bromide (0.63 M in THF) was prepared as for the synthesis of **11f**.

11o was prepared from (±)-(2*R*,3*S*)-2-butyl-2-methyl-3-(2-oxooxazolidine-3-carbonyl)-6-phenylhexanal (35.9 mg, 100 µmol, 1 equiv., d.r. < 5:95) and [4-(trifluoromethyl)phenyl]magnesium bromide (0.63 M in THF, 0.32 mL, 0.20 mmol, 2 equiv.) following the general procedure **B**. The crude product (d.r. = 10:nd:72:18) was purified by flash column chromatography on silica gel (EtOAc/heptane, 5% to 30%) to afford **11o** (22.2 mg, 53.1 µmol, 53%, fraction 1 (20 mg), d.r. = nd:nd:80:nd, fraction 2 (2.2 mg), d.r. = 15:nd:nd:85) as colorless oils.

Data are reported for the major diastereoisomer.

^{1}H NMR (600 MHz, $CDCl_3$): δ = 7.62 (d, *J* = 8.2 Hz, 2H), 7.32 (d, *J* = 8.2 Hz, 2H), 7.30 – 7.26 (m, 2H), 7.21 – 7.17 (m, 3H), 5.23 (s, 1H), 2.74 – 2.64 (m, 2H), 2.31 (dd, *J* = 9.7, 4.4 Hz, 1H), 2.07 (m, 1H), 1.81 – 1.67 (m, 2H), 1.58 – 1.44 (m, 2H), 1.37 (m, 1H), 1.34 – 1.24 (m, 3H), 1.18 (m, 1H), 0.89 (t, *J* = 7.2 Hz, 3H), 0.68 (s, 3H);

^{13}C NMR (151 MHz, $CDCl_3$): δ = 178.3, 141.7, 140.3, 130.6 (q, *J* = 32.4 Hz), 128.5 (br, 4C), 126.6 (2C), 126.0, 125.3 (q, *J* = 4.3 Hz, 2C), 124.0 (q, *J* = 272.9 Hz), 85.4, 49.2, 46.4, 35.7, 34.1, 29.0, 26.5, 24.7, 23.4, 21.6, 14.1;

^{19}F NMR (565 MHz, $CDCl_3$): δ = -62.6 (3 F);

FTIR (neat): 2960, 2933, 2858, 1775, 1325, 1166, 1126, 1068, 1009, 840, 750 cm^{-1};

HRMS (ESI): Calculated for $C_{25}H_{29}F_3O_2H^+$ $[M+H]^+$: 419.2192, Found: 419.2194.

Selected nOe correlations:

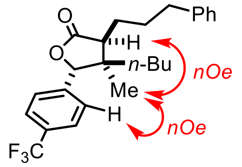

(±)–(3*S*,4*S*,5*R*)-5-Allyl-3-butyl-4-isopropyldihydrofuran-2(3*H*)-one (11p)

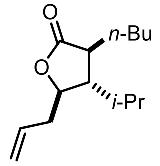

Formula: $C_{14}H_{24}O_2$

MW = 224.3 g/mol

11p was prepared from (±)-(2*S*,3*S*)-2-isopropyl-3-(2-oxooxazolidine-3-carbonyl)heptanal (40.0 mg, 149 µmol, 1 equiv., d.r. = 90:10) following the general procedure **C**. The crude product (d.r. = 83:12:5:nd) was purified by flash column chromatography on silica gel (EtOAc/heptane, 5% to 30%) to afford **11p** (26.0 mg, 116 µmol, 78%, d.r. = 83:9:6:2) as a colorless oil.

Data are reported for the major diastereoisomer.

^{1}H NMR (600 MHz, $CDCl_3$): δ = 5.92 – 5.71 (m, 1H), 5.22 – 5.09 (m, 2H), 4.28 – 4.16 (m, 1H), 1.48 – 1.34 (m, 3H), 1.83 (m, 1H), 1.78 – 1.70 (m, 2H), 1.68 – 1.61 (m, 1H), 1.49 – 1.41 (m, 1H), 1.40 – 1.30 (m, 3H), 0.97 – 0.89 (m, 9H);

^{13}C NMR (151 MHz, $CDCl_3$): δ = 179.3, 133.0, 118.8, 81.0, 50.1, 43.4, 40.8, 31.8, 30.8, 29.1, 22.8, 19.9, 19.8, 14.0;

FTIR (neat): 2958, 2931, 2873, 1770, 1643, 1467, 1391, 1373, 1275, 1184, 1073, 1000, 918 cm^{-1};

HRMS (ESI): Calculated for $C_{14}H_{24}O_2Na^+$ $[M+Na]^+$: 247.1669, Found: 247.1668.

(±)-(3*S*,4*S*,5*R*)-5-Allyl-4-isopropyl-3-(3-phenylpropyl)dihydrofuran-2(3*H*)-one (11q)

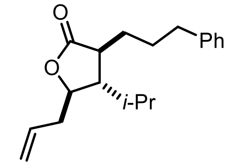

Formula: $C_{19}H_{26}O_2$

MW = 286.4 g/mol

11q was prepared from (±)-(2*S*,3*S*)-2-isopropyl-3-(2-oxooxazolidine-3-carbonyl)-6-phenylhexanal (33.1 mg, 100 μmol, 1 equiv., d.r. = 91:9) following the general procedure **C**. The crude product (d.r. = 84:10:6:nd) was purified by flash column chromatography on silica gel (EtOAc/heptane, 5% to 30%) to afford **11q** (13.8 mg, 48.2 μmol, 48%, d.r. = 84:8:6:2) as a colorless oil.

Data are reported for the major diastereoisomer.

^{1}H NMR (600 MHz, $CDCl_3$): δ = 7.28 (dd, *J* = 14.0, 6.8 Hz, 2H), 7.19 (t, *J* = 7.3 Hz, 3H), 5.88 – 5.70 (m, 1H), 5.20 – 5.06 (m, 2H), 4.22 (dd, *J* = 12.0, 5.9 Hz, 1H), 2.73 – 2.57 (m, 2H), 2.45 – 2.38 (m, 2H), 2.38 – 2.32 (m, 1H), 1.88 – 1.65 (m, 6H), 0.92 (dd, *J* = 6.8, 1.2 Hz, 6H);

^{13}C NMR (151 MHz, $CDCl_3$): δ = 179.1, 141.9, 132.9, 128.52 (2C), 128.51 (2C), 126.0, 118.9, 81.0, 50.1, 43.3, 40.7, 35.9, 31.5, 30.7, 28.6, 19.84, 19.77;

FTIR (neat): 3027, 2957, 2929, 2867, 1765, 1643, 1604, 1496, 1454, 1391, 1365, 1339, 1317, 1183, 1078, 1087, 983, 917 cm^{-1};

HRMS (ESI): Calculated for $C_{19}H_{26}O_2Na^+$ $[M+Na]^+$: 309.1825, Found: 309.1826.

(±)–(3*S*,4*R*,5*S*)-5-Allyl-3,4-dibutyldihydrofuran-2(3*H*)-one (11r)

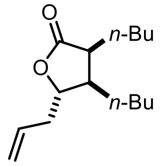

Formula: $C_{15}H_{26}O_2$

MW = 238.4 g/mol

11r was prepared from (±)-(2*R*,3*S*)-2-butyl-3-(2-oxooxazolidine-3-carbonyl)heptanal (30.0 mg, 106 µmol, 1 equiv., d.r. = 21:79) following the general procedure **C**. The crude product (d.r. = 20:nd:80:nd) was purified by flash column chromatography on silica gel (EtOAc/heptane, 5% to 30%) to afford **11r** (23.7 mg, 99.4 µmol, 94%, d.r. = 20:nd:80:nd) as a colorless oil.

Data are reported for the major diastereoisomer.

^{1}H NMR (600 MHz, $CDCl_3$): δ = 5.90 – 5.73 (m, 1H), 5.21 – 5.09 (m, 2H), 4.22 (td, *J* = 6.3, 4.7 Hz, 1H), 2.59 (dd, *J* = 15.2, 7.5 Hz, 1H), 2.43 – 2.37 (m, 2H), 2.24 – 2.14 (m, 1H), 1.71 – 1.58 (m, 1H), 1.52 – 1.17 (m, 11H), 0.94 – 0.88 (m, 6H);

^{13}C NMR (151 MHz, $CDCl_3$): δ = 178.8, 132.9, 118.8, 82.2, 42.4, 42.2, 38.3, 29.8, 29.5, 26.8, 24.9, 22.85, 22.81, 14.1, 14.0;

FTIR (neat): 2958, 2925, 2855, 1773, 1466, 1213, 1017, 915, 739 cm^{-1};

HRMS (ESI): Calculated for $C_{15}H_{26}O_2Na^+$ $[M+Na]^+$: 261.1825, Found: 261.1827.

2.5 Formation of *O*-lactones

(±)-(3*S*,4*S*,5*S*)-4-Isopropyl-5-methoxy-3-(3-phenylpropyl)dihydrofuran-2(3*H*)-one (12a)

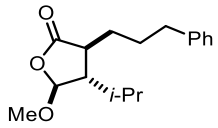

Formula: $C_{17}H_{24}O_3$

MW = 276.4 g/mol

To a -78 °C solution of (±)-(2*S*,3*S*)-2-isopropyl-3-(2-oxooxazolidine-3-carbonyl)-6-phenylhexanal (15 mg, 45 µmol, 1.0 equiv., d.r. = 95:5) in THF (2 mL) was added MeOH (18 µl, 0.453 mmol, 10 equiv.) and NaOMe (2.5 mg, 45 µmol, 1.0 equiv.). The mixture was warmed to 0 °C and left stirring for 2 h. The reaction was quenched with sat. aq. NH_4Cl and extracted with Et_2O (2 x 5 mL). The combined organic phases were dried over Na_2SO_4, filtered and concentrated *in vacuo*. The crude product (d.r. = 89:6:5:nd) was purified by column chromatography on silica gel (EtOAc/Heptane, 2% to 10%) to afford **12a** (10 mg, 36 µmol, 80%, d.r. > 20:1) as a colorless oil.

Data are reported for the major diastereoisomer.

^{1}H NMR (600 MHz, CDCl$_3$): δ = 7.30 (t, *J* = 7.6 Hz, 2H), 7.26 – 7.15 (m, 3H), 5.09 (d, *J* = 2.3 Hz, 1H), 3.50 (s, 3H), 2.66 (dd, *J* = 15.0, 6.6 Hz, 2H), 2.36 (dd, *J* = 7.5, 4.6 Hz, 1H), 1.93 (ddd, *J* = 6.7, 4.5, 2.3 Hz, 1H), 1.89 – 1.82 (m, 1H), 1.83 – 1.70 (m, 4H), 0.95 (d, *J* = 6.8 Hz, 3H), 0.94 (d, *J* = 6.8 Hz, 3H);

^{13}C NMR (151 MHz, CDCl$_3$): δ =178.9, 141.9, 128.51 (2C), 128.49 (2C), 126.0, 107.5, 57.1, 52.6, 43.0, 35.8, 31.8, 29.8, 29.0, 19.6 (2C);

FTIR (neat): 2960, 2939, 1775, 1454, 1119, 948 cm^{-1};

HRMS (ESI): Calculated for $C_{17}H_{24}O_3Na^+$ $[M+Na]^+$: 299.1623, Found: 299.1618.

(±)–(3*S*,4*S*,5*S*)-4-Butyl-5-methoxy-3-(3-phenylpropyl)dihydrofuran-2(3*H*)-one (12b)

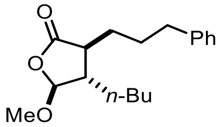

Formula: $C_{18}H_{26}O_3$

MW = 290.3 g/mol

To a -20 °C solution of (±)-(2*S*,3*S*)-2-butyl-3-(2-oxooxazolidine-3-carbonyl)-6-phenylhexanal (15 mg, 43 µmol, 1 equiv., d.r. = 88:12) and MeOH (18 µL, 0.43 mmol, 10 equiv.) in CH_2Cl_2 (0.4 mL) was added NaOMe (1.2 mg, 22 µmol, 0.5 equiv.) and the mixture was slowly allowed to warm to rt and stirred for 16 h. The reaction was quenched with sat. aq. NH_4Cl and the aqueous phase was extracted with CH_2Cl_2. The combined organic phases were dried over $mgSO_4$, filtered and concentrated *in vacuo*. The crude product (d.r. = 61:27:10:2) was purified by flash column chromatography on silica gel (EtOAc/Heptane, 20% to 30%) to afford **12b** (5.0 mg, 17.2 µmol, 40%, d.r. = 26:54:19:nd) as a colorless oil.

Data are reported for the major diastereoisomer.

^{1}H NMR (600 MHz, $CDCl_3$): δ = 7.31 – 7.26 (m, 2H), 7.21 – 7.15 (m, 3H), 5.00 (d, *J* = 3.0 Hz, 1H), 3.49 (s, 3H), 2.73 – 2.58 (m, 2H), 2.23 (dt, *J* = 7.2, 5.7 Hz, 1H), 2.01 (ddd, *J* = 10.4, 7.7, 3.1 Hz, 1H), 1.88 – 1.64 (m, 4H), 1.50 – 1.39 (m, 2H), 1.36 – 1.18 (m, 4H), 0.90 (t, *J* = 7.2 Hz, 3H);

^{13}C NMR (151 MHz, $CDCl_3$): δ = 178.2, 142.0, 128.49 (4C), 126.0, 109.3, 57.3, 46.6, 46.1, 35.8, 32.4, 30.9, 29.2, 29.0, 22.7, 14.0;

FTIR (neat): 2930, 2859, 2357, 1769, 1603, 1454, 1384, 1182, 1051, 959, 748, 700 cm^{-1};

HRMS (ESI): Calculated for $C_{18}H_{26}O_3Na^+$ $[M+Na]^+$: 313.1774, Found: 313.1777.

(±)–(3*S*,4*S*,5*S*)-4-Butyl-5-isopropoxy-3-(3-phenylpropyl)dihydrofuran-2(3*H*)-one (12c)

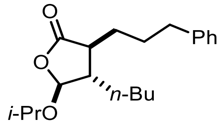

Formula: $C_{20}H_{30}O_3$

MW = 318.5 g/mol

To a rt solution of KO*t*-Bu (5.0 mg, 43 µmol, 0.5 equiv.) in *i*-PrOH (0.8 mL) was added (±)-(2*S*,3*S*)-2-butyl-3-(2-oxooxazolidine-3-carbonyl)-6-phenylhexanal (30 mg, 86.8 µmol, 1 equiv., d.r. = 89:11) and the mixture was left stirring at rt for 16 h. The reaction was quenched with sat. aq. NH_4Cl and the aqueous phase was extracted with CH_2Cl_2. The combined organic phases were dried over mgSO$_4$, filtered and concentrated *in vacuo*. The crude product (d.r. = 86:9:5:nd) was purified by flash column chromatography on silica gel (EtOAc/Heptane 20% to 30%) to afford **12c** (11.0 mg, 34.7 µmol, 40%, d.r. = 92:4:3:nd) as a colorless oil.

Data are reported for the major diastereoisomer.

^{1}H NMR (400 MHz, CDCl$_3$): δ = 7.34 – 7.11 (m, 5H), 5.18 (d, *J* = 3.8 Hz, 1H), 3.95 (dt, *J* = 12.4, 6.2 Hz, 1H), 2.65 (td, *J* = 6.3, 3.2 Hz, 2H), 2.22 (dd, *J* = 13.0, 6.3 Hz, 1H), 1.99 (qd, *J* = 7.0, 3.9 Hz, 1H), 1.89 – 1.62 (m, 4H), 1.51 – 1.38 (m, 2H), 1.38 – 1.24 (m, 4H), 1.20 (dd, *J* = 9.5, 6.2 Hz, 6H), 0.90 (t, *J* = 6.9 Hz, 3H);

^{13}C NMR (101 MHz, CDCl$_3$): δ = 178.1, 142.0, 128.54 (2C), 128.49 (2C), 126.0, 106.5, 72.4, 46.8, 46.2, 35.9, 32.2, 30.4, 29.0, 29.1, 23.4, 22.7, 21.8, 14.0;

FTIR (neat): 2929, 2859, 2353, 1770, 1604, 1145, 1110, 938, 747, 699 cm^{-1};

HRMS (ESI): Calculated for $C_{20}H_{30}O_3Na^+$ $[M+Na]^+$: 341.2087, Found: 341.2089.

(3*S*,3a*R*,6a*S*)-3-(3-Phenylpropyl)tetrahydrofuro[2,3-b]furan-2(3*H*)-one (13)

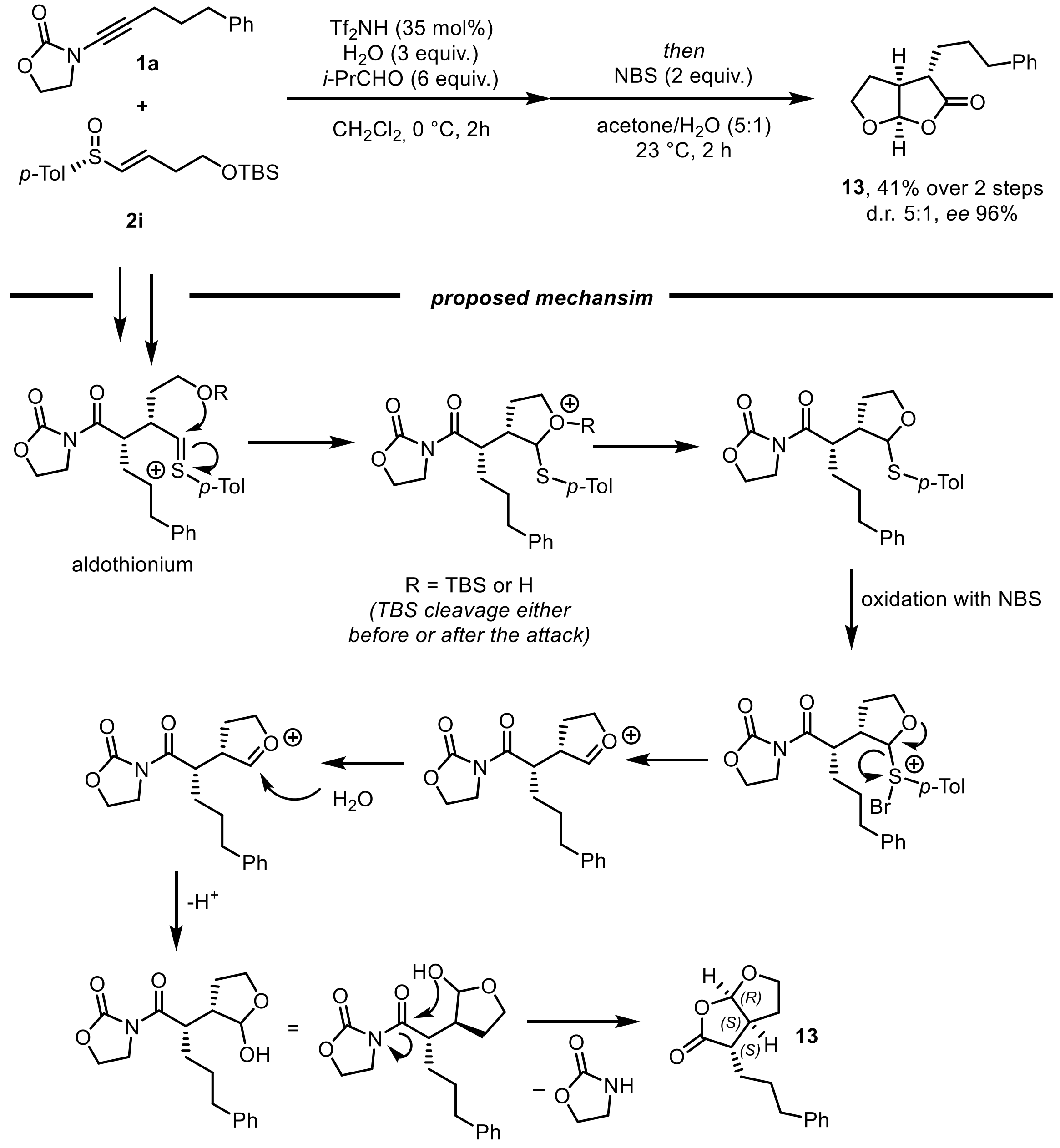

Formula: $C_{15}H_{18}O_3$

MW = 246.3 g/mol

To a vigorously stirred 0 °C solution of 3-(5-phenylpent-1-yn-1-yl)oxazolidin-2-one (69 mg, 0.30 mmol, 2.0 equiv.), (*R*,*E*)-*tert*-butyldimethyl[(4-(p-tolylsulfinyl)but-3-en-1-yl)oxy]silane (49 mg, 0.15 mmol, 1.0 equiv.), *i*-PrCHO (82 µL, 0.9 mmol, 6.0 equiv.) and H_2O (8.1 µL, 0.45 mmol, 3.0 equiv.) in CH_2Cl_2 (1.5 mL) was added a solution of Tf_2NH (15 mg, 53 µmol, 35 mol%) in CH_2Cl_2 (0.75 mL) over 30 min using a syringe

pump. After addition, the reaction mixture was stirred at 0 °C for 2 h. The reaction was then quenched by addition of a aq. $NaHCO_3$ solution (made by mixing equal volumes of sat. aq. $NaHCO_3$ and distilled water) and the phases were separated. The aqueous phase was extracted with CH_2Cl_2 and the combined organic phases were dried over Na_2SO_4, filtered and concentrated *in vacuo*. The crude product was dissolved in acetone/H_2O (5:1), NBS (53 mg, 0.30 mmol, 2.0 equiv.) was added at rt and the mixture was stirred for 2 h. Sat. aq. $NaHCO_3$ was added and the mixture was vigorously stirred for further 2 h. Et_2O was added and the phases were separated. The organic phase was washed (3 x 10 mL) with sat. aq. $NaHCO_3$, dried over Na_2SO_4, filtered and concentrated *in vacuo*. The crude product (d.r. = 5:1) was purified by flash column chromatography on silica gel (EtOAc/Heptane, 5 to 25%) to afford **13** (ee 96%, 15 mg, 61 µmol, 41%, d.r. = 5:1) as a colorless oil.

Data are reported for the major diastereoisomer.

^{1}H NMR (600 MHz, $CDCl_3$): δ = 7.32 (t, *J* = 7.5 Hz, 2H), 7.25 – 7.19 (m, 3H), 6.02 (d, *J* = 5.5 Hz, 1H), 4.15 – 4.07 (m, 1H), 3.96 – 3.88 (m, 1H), 2.88 – 2.78 (m, 1H), 2.69 (t, *J* = 7.5 Hz, 2H), 2.53 – 2.44 (m, 1H), 2.26 – 2.16 (m, 1H), 1.96 – 1.87 (m, 1H), 1.85 – 1.74 (m, 3H), 1.69 – 1.61 (m, 1H);

^{13}C NMR (151 MHz, $CDCl_3$): δ = 177.9, 141.6, 128.6 (2C), 128.5 (2C), 126.2, 107.0, 67.5, 46.5, 45.0, 35.8, 32.4, 31.7, 29.0;

FTIR (neat): 2931, 2860, 1768, 1494, 1357, 966 cm^{-1};

HRMS (ESI): Calculated for $C_{15}H_{18}O_3Na^+$: 269.1148, Found: 269.1100;

$[\alpha]_D^{20}$ – 35.5 (c = 1.00, $CHCl_3$). Enantiomeric excess 96% was determined by chiral HPLC analysis: Chiracel OD-H, *n*-heptane/IPA 8:2, 0.7 mL/min, 25 °C, detection at 210 nm, retention time (min): 16.9 (major) and 18.8 (minor).

Selected nOe correlations:

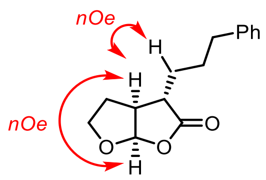

HPLC chromatograms:

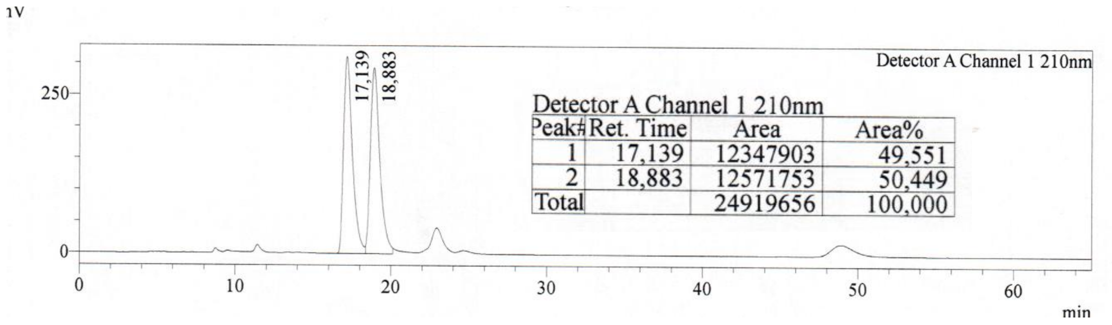

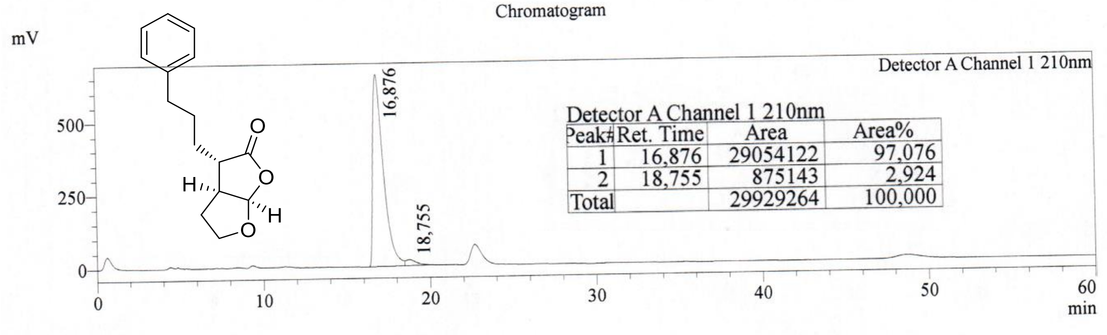

2.6 Synthesis of γ-lactams

2.6.1 General procedure D: Synthesis of lactam using Et_3SiH

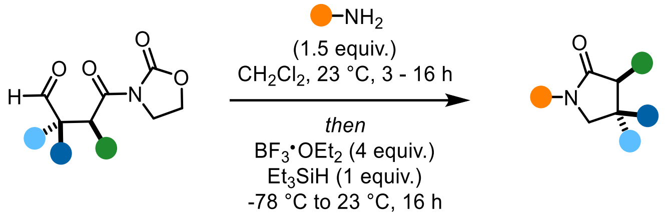

To a rt solution of the *aldehyde* (1 equiv.) in CH_2Cl_2 (0.1 M) was added the *amine* (1.5 equiv.) and the mixture was left stirring for 3 h at the same temperature (16 h for β-quaternary aldehydes). The mixture was cooled to -78 °C and $BF_3{\cdot}OEt_2$ (4 equiv.) was added, followed by Et_3SiH (1 equiv.). The reaction mixture was allowed to gradually warm to rt over 16 h while stirring. A sat. aq. solution of $NaHCO_3$ was added and the phases were separated. The aqueous phase was extracted with CH_2Cl_2. The combined organic phases were dried over $mgSO_4$, filtered and concentrated *in vacuo*. The crude product was subjected to flash column chromatography on silica gel (EtOAc/Heptane, usually 5% to 30% over 25 cv) to afford the corresponding lactam.

2.6.2 General procedure E: Synthesis of lactam using $NaBH(OAc)_3$

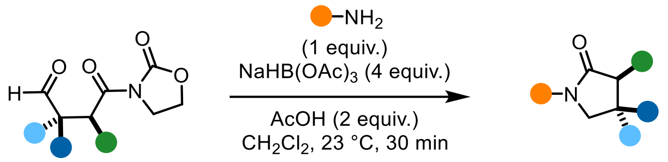

To a rt solution of the *aldehyde* (1 equiv.) in CH_2Cl_2 (0.1 M) was added AcOH (2 equiv.), $NaBH(OAc)_3$ (4 equiv.), the *amine* (1 equiv.) and the mixture was left stirring at the same temperature for 30 min. A sat. aq. solution of $NaHCO_3$ was added, the phases were separated and the aqueous phase was extracted with CH_2Cl_2. The combined organic phases were dried over $mgSO_4$, filtered and concentrated *in vacuo*. The crude product was subjected to flash column chromatography on silica gel (EtOAc/Heptane, usually 5% to 30% over 25 cv) to afford the corresponding lactam.

2.6.3 Characterization of products

(±)–(3*S*,4*S*)-1-Benzyl-4-isopropyl-3-(3-phenylpropyl)pyrrolidin-2-one (14a)

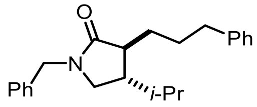

Formula: $C_{23}H_{29}NO$

MW = 335.5 g/mol

14a was prepared from (±)-(2*S*,3*S*)-2-isopropyl-3-(2-oxooxazolidine-3-carbonyl)-6-phenylhexanal (d.r. = 90:10, 30 mg, 90 µmol, 1 equiv.) following the general procedure **D**. The crude product (d.r. > 95:5) was purified by flash column chromatography on silica gel (EtOAc/Pentane, 5% to 30%) to afford **14a** (22 mg, 67 µmol, 74%, d.r. > 95:5).

Data are reported for the major diastereoisomer.

^{1}H NMR (600 MHz, $CDCl_3$): δ = 7.30 (m, 5H), 7.23 – 7.16 (m, 5H), 4.49 (d, *J* = 14.6 Hz, 1H), 4.37 (d, *J* = 14.6Hz, 1H), 3.20 (dd, *J* = 9.9, 8.6 Hz, 1H), 2.88 (dd, *J* = 10.0, 5.7 Hz, 1H), 2.70 – 2.60 (m, 2H), 2.31 (dd, *J* = 11.5, 5.9 Hz, 1H), 1.84 (dq, *J* = 8.5, 6.1 Hz, 1H), 1.80 – 1.58 (m, 5H), 0.84 (d, *J* = 6.8 Hz, 3H), 0.80 (d, *J* = 6.7 Hz, 3H);

^{13}C NMR (151 MHz, $CDCl_3$): δ = 176.6, 142.4, 136.7, 128.8 (2C), 128.5 (2C), 128.4 (2C), 128.3 (2C), 127.6, 125.9, 48.2, 46.7, 45.6, 42.6, 36.3, 31.6, 31.5, 28.6, 20.2, 19.0;

FTIR (neat): 3027, 2953, 2926, 2869, 1681, 1603, 1494, 1441, 1389, 1359, 1320, 1294, 1257, 1203, 1178, 1078, 1029, 944, 808 cm^{-1};

HRMS (ESI): Calculated for $C_{23}H_{29}NONa^+$ $[M+Na]^+$: 358.2141, Found: 358.2142.

(±)–(3*S*,4*S*)-1-Allyl-4-butyl-3-(3-phenylpropyl)pyrrolidin-2-one (14b)

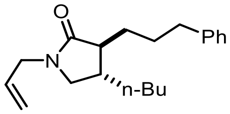

Formula: $C_{20}H_{29}NO$

MW = 299.5 g/mol

14b was prepared from (±)-(2*S*,3*S*)-2-butyl-3-(2-oxooxazolidine-3-carbonyl)-6-phenylhexanal (d.r. = 88:12, 30 mg, 90 µmol, 1 equiv.) following the general procedure **D**. The crude product (d.r. = 89:11) was purified by flash column chromatography on silica gel (EtOAc/Pentane, 5% to 30%) to afford **14b** (17.3 mg, 58 µmol, 58%) as a single diastereoisomer as a colorless oil.

Data are reported for the major diastereoisomer.

^{1}H NMR (600 MHz, CDCl$_3$): δ = 7.29 – 7.26 (m, *J* = 7.6 Hz, 2H), 7.20 – 7.15 (m, 3H), 5.70 (ddt, *J* = 16.6, 10.5, 6.1 Hz, 1H), 5.19 – 5.13 (m, 2H), 3.89 (dd, *J* = 15.2, 6.0 Hz, 1H), 3.83 (dd, *J* = 15.2, 6.1 Hz, 1H), 3.36 (dd, *J* = 9.4, 8.4 Hz, 1H), 2.87 (dd, *J* = 9.7, 6.7 Hz, 1H), 2.64 (t, *J* = 7.4 Hz, 2H), 2.11 (dd, *J* = 12.0, 7.1 Hz, 1H), 2.00 – 1.93 (m, 1H), 1.79 – 1.67 (m, 3H), 1.61 – 1.52 (m, 2H), 1.35–1.28 (m, 3H), 1.27 – 1.23 (m, 2H), 0.89 (t, *J* = 7.1 Hz, 3H);

^{13}C NMR (151 MHz, CDCl$_3$): δ = 176.3, 142.5, 132.8, 128.6 (2C), 128.4 (2C), 125.9, 117.9, 51.2, 48.3, 45.4, 37.6, 36.3, 34.5, 30.6, 29.6, 28.9, 22.9, 14.1;

FTIR (neat): 2925, 1686, 1494, 1453, 1266, 1030, 991, 746, 700 cm^{-1};

HRMS (ESI): Calculated for $C_{20}H_{29}NONa^{+}$ $[M+Na]^{+}$: 322.2141, Found: 322.2145.

(±)–(3*S*,4*S*)-4-Butyl-1-(4-methoxybenzyl)-3-(3-phenylpropyl)pyrrolidin-2-one (14c)

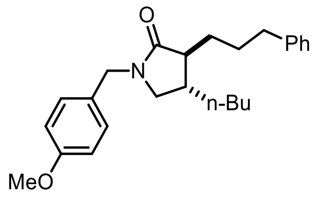

Formula: $C_{25}H_{33}NO_2$

MW = 379.5 g/mol

14c was prepared from (±)-(2*S*,3*S*)-2-butyl-3-(2-oxooxazolidine-3-carbonyl)-6-phenylhexanal (d.r. = 88:12, 34.5 mg, 0.10 mmol, 1 equiv.) following the general procedure **D**. The crude product (d.r. = 91:9) was purified by flash column chromatograph on silica gel (EtOAc/Pentane, 5% to 30%) to afford **14c** (26.6 mg, 70.1 µmol, 70%) as a colorless oil.

Data are reported for the major diastereoisomer.

^{1}H NMR (600 MHz, $CDCl_3$): δ = 7.29–7.27 (m, 2H), 7.20 – 7.16 (m, 3H), 7.15–7.10 (m, 2H), 6.86 – 6.81 (m, 2H), 4.39 (d, *J* = 14.5 Hz, 1H), 4.33 (d, *J* = 14.5 Hz, 1H), 3.79 (s, 3H), 3.25 (dd, *J* = 9.7, 8.1 Hz, 1H), 2.76 (dd, *J* = 9.7, 6.6 Hz, 1H), 2.67–2.62 (m, 2H), 2.13 (td, *J* = 7.1, 5.1 Hz, 1H), 1.95–1.88 (m, 1H), 1.81 – 1.71 (m, 3H), 1.63 – 1.59 (m, 1H), 1.53 – 1.47 (m, 1H), 1.31 – 1.22 (m, 3H), 1.21 – 1.15 (m, 2H), 0.85 (t, *J* = 7.2 Hz, 3H);

^{13}C NMR (151 MHz, $CDCl_3$): δ = 176.4, 159.1, 142.5, 129.6 (2C), 128.9, 128.6 (2C), 128.4 (2C), 125.9, 114.2 (2C), 55.4, 50.9, 48.3, 46.1, 37.5, 36.3, 34.5, 30.5, 29.5, 28.9, 22.9, 14.1;

FTIR (neat): 2925, 1771, 1511, 1494, 1453, 1244, 1175, 1033, 748, 700 cm^{-1};

HRMS (ESI): Calculated for $C_{25}H_{33}NO_2H^+$ $[M+H]^+$: 380.2582, Found: 330.2584.

(±)-(3*S*,4*S*)-1-Benzyl-4-butyl-4-methyl-3-(3-phenylpropyl)pyrrolidin-2-one (14d)

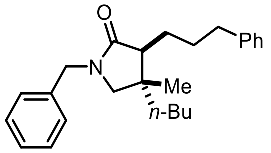

Formula: $C_{25}H_{33}NO$

MW = 363.5 g/mol

14d was prepared from (±)-(2*S*,3*S*)-2-butyl-2-methyl-3-(2-oxooxazolidine-3-carbonyl)-6-phenylhexanal (d.r. = 3:97, 31 mg, 87 μmol, 1 equiv.) following the general procedure **D**. The crude product (d.r. > 95:5) was purified by flash column chromatograph on silica gel (EtOAc/Pentane, 5% to 30%) to afford **14d** (20.6 mg, 56.7 μmol, 65%) as only one diastereoisomer, as a colorless oil.

Data are reported for the major diastereoisomer.

^{1}H NMR (400 MHz, CDCl$_3$): δ = 7.25 – 7.16 (m, 5H), 7.16 – 7.07 (m, 5H), 4.42 (d, *J* = 14.6 Hz, 1H), 4.28 (d, *J* = 14.6 Hz, 1H), 2.89 (d, *J* = 9.5 Hz, 1H), 2.66 (d, *J* = 9.5 Hz, 1H), 2.63 – 2.53 (m, 2H), 2.07 (t, *J* = 6.4 Hz, 1H), 2.03 – 1.90 (m, 1H), 1.75 – 1.60 (m, 2H), 1.45 – 1.31 (m, 2H), 1.27 – 1.07 (m, 4H), 1.04 – 0.93 (m, 1H), 0.85 – 0.74 (m, 6H);

^{13}C NMR (101 MHz, CDCl$_3$): δ = 176.4, 142.5, 136.9, 128.7 (2C), 128.6 (2C), 128.4 (2C), 128.4 (2C), 127.6, 125.8, 57.0, 51.8, 46.7, 40.4, 40.0, 36.4, 30.3, 26.8, 26.0, 23.5, 20.0, 14.2;

FTIR (neat): 2927, 2855, 1682, 1494, 1424, 1379, 1299, 1078, 746, 698 cm^{-1};

HRMS (ESI): Calculated for $C_{25}H_{33}NOH^+$ $[M+H]^+$: 364.2635, Found: 364.2634.

(±)-(3*S*,4*R*)-1-Benzyl-4-butyl-4-methyl-3-(3-phenylpropyl)pyrrolidin-2-one (14e)

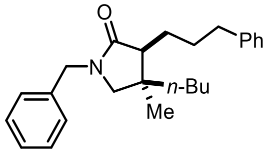

Formula: $C_{25}H_{33}NO$

MW = 363.5 g/mol

14e was prepared from (±)-(2*R*,3*S*)-2-butyl-2-methyl-3-(2-oxooxazolidine-3-carbonyl)-6-phenylhexanal (d.r. > 95:5, 35.9 mg, 0.10 mmol, 1 equiv.) following the general procedure **D**. The crude product (d.r. < 5:95) was purified by flash column chromatograph on silica gel (EtOAc/Pentane, 5% to 30%) to afford **14e** (31.2 mg, 85.8 µmol, 86%) as only one diastereoisomer, as a colorless oil.

Data are reported for the major diastereoisomer.

^{1}H NMR (400 MHz, $CDCl_3$): δ = 7.33 – 7.25 (m, 5H), 7.23 – 7.15 (m, 5H), 4.50 (d, *J* = 14.5 Hz, 1H), 4.31 (d, *J* = 14.5 Hz, 1H), 2.95 (d, *J* = 9.7 Hz, 1H), 2.77 (d, *J* = 9.7 Hz, 1H), 2.73 – 2.60 (m, 2H), 2.14 – 2.08 (m, 1H), 2.06 – 1.92 (m, 1H), 1.82 – 1.61 (m, 2H), 1.47 (ddt, *J* = 13.6, 11.6, 5.6 Hz, 1H), 1.23 – 1.09 (m, 4H), 1.02 (s, 3H), 1.10 – 0.89 (m, 2H), 0.79 (t, *J* = 7.3 Hz, 3H);

^{13}C NMR (101 MHz, $CDCl_3$): δ = 176.6, 142.5, 136.9, 128.7 (2C), 128.6 (2C), 128.5 (2C), 128.4 (2C), 127.6, 125.8, 55.6, 53.1, 46.6, 39.6, 36.3, 34.4, 30.0, 26.6, 25.7, 25.3, 23.5, 14.1;

FTIR (neat): 3027, 2956, 2854, 1689, 1494, 1454, 1424, 1266, 745, 699 cm^{-1};

HRMS (ESI): Calculated for $C_{25}H_{33}NOH^{+}$ $[M+H]^{+}$: 364.2635, Found: 364.2636.

(±)-(3*S*,4*R*)-1-Benzyl-4-butyl-3-(3-phenylpropyl)pyrrolidin-2-one (14f)

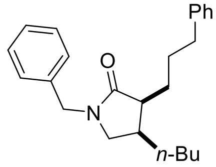

Formula: $C_{24}H_{31}NO$

MW = 349.5 g/mol

14f was prepared from (±)-(2*R*,3*S*)-2-butyl-3-(2-oxooxazolidine-3-carbonyl)-6-phenylhexanal (d.r. = 5:95, 34.5 mg, 0.10 mmol, 1 equiv.) following the general procedure **E**. The crude product (d.r. = 12:88) was purified by flash column chromatography on silica gel (EtOAc/Heptane, 5% to 30%) to afford **14f** (d.r. = 14:86, 29 mg, 84 µmol, 84%) as a colorless oil.

Data are reported for the major diastereoisomer.

^{1}H NMR (700 MHz, $CDCl_3$): δ = 7.34 – 7.27 (m, 5H), 7.22 – 7.17 (m, 5H), 4.51 (d, *J* = 14.7 Hz, 1H), 4.35 (d, *J* = 14.6 Hz, 1H), 3.18 (dd, *J* = 9.7, 6.9 Hz, 1H), 2.86 (dd, *J* = 9.8, 5.3 Hz, 1H), 2.72 – 2.63 (m, 2H), 2.47 (dd, *J* = 14.2, 7.6 Hz, 1H), 2.30 – 2.22 (m, 1H), 1.86 – 1.78 (m, 1H), 1.75 – 1.66 (m, 2H), 1.56 – 1.48 (m, 1H), 1.33 – 1.08 (m, 6H), 0.83 (t, *J* = 7.3 Hz, 3H);

^{13}C NMR (176 MHz, $CDCl_3$): δ = 176.6, 142.4, 136.9, 128.7 (2C), 128.6 (2C), 128.4 (2C), 128.2 (2C), 127.6, 125.8, 50.0, 46.6, 45.4, 36.1, 35.9, 29.7, 29.5, 27.6, 25.4, 22.8, 14.1;

FTIR (neat): 2928, 2857, 1686, 1495, 1453, 1416, 1264, 744, 699 cm^{-1};

HRMS (ESI): Calculated for $C_{24}H_{31}NONa^+$ $[M+Na]^+$: 372.2298, Found: 372.2298.

(±)-(3*S*,4*R*)-4-Butyl-1-(4-methoxybenzyl)-3-(3-phenylpropyl)pyrrolidin-2-one (14g)

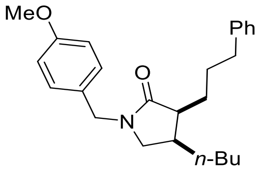

Formula: $C_{25}H_{33}NO_2$

MW = 379.5 g/mol

14g was prepared from (±)-(2*R*,3*S*)-2-butyl-3-(2-oxooxazolidine-3-carbonyl)-6-phenylhexanal (d.r. = 17:83, 40.1 mg, 0.12 mg, 1 equiv.) following the general procedure **E**. The crude product (d.r. = 21:79) was purified by flash column chromatography on silica gel (EtOAc/Heptane, 5% to 30%) to afford **14g** (d.r. = 7:93, 27.8 mg, 73.2 µmol, 63%) as a colorless oil.

Data are reported for the major diastereoisomer.

^{1}H NMR (500 MHz, $CDCl_3$): δ = 7.30 – 7.25 (m, 2H), 7.21 – 7.15 (m, 3H), 7.14–7.10 (m, 2H), 6.87 – 6.79 (m, 2H), 4.45 (d, *J* = 14.5 Hz, 1H), 4.27 (d, *J* = 14.5 Hz, 1H), 3.79 (s, 3H), 3.16 (dd, *J* = 9.7, 6.9 Hz, 1H), 2.83 (dd, *J* = 9.7, 5.4 Hz, 1H), 2.66 (app.t, *J* = 7.3 Hz, 2H), 2.45 (dd, *J* = 14.1, 7.6 Hz, 1H), 2.28 – 2.19 (m, 1H), 1.86 – 1.64 (m, 3H), 1.56 – 1.44 (m, 1H), 1.32 – 1.04 (m, 6H), 0.82 (t, *J* = 7.3 Hz, 3H);

^{13}C NMR (126 MHz, $CDCl_3$): δ = 176.5, 159.1, 142.4, 129.6 (2C), 129.0, 128.6 (2C), 128.4 (2C), 125.8, 114.1 (2C), 55.4, 49.9, 46.0, 45.5, 36.2, 35.9, 29.8, 29.5, 27.6, 25.5, 22.8, 14.1;

FTIR (neat): 2928, 2856, 1681, 1511, 1453, 1244, 1174, 1033, 845, 747 cm^{-1};

HRMS (ESI): Calculated for $C_{25}H_{33}NO_2H^+$ [M+H]$^+$: 380.2584, Found: 380.2587.

(±)-(3*S*,4*R*)-1-Allyl-4-butyl-3-(3-phenylpropyl)pyrrolidin-2-one (14h)

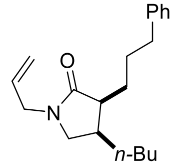

Formula: $C_{20}H_{29}NO$

MW = 299.5 g/mol

14h was prepared from (±)-(2*R*,3*S*)-2-butyl-3-(2-oxooxazolidine-3-carbonyl)-6-phenylhexanal (d.r. = 5:95, 34.5 mg, 0.10 mmol, 1 equiv.) following the general procedure **E**. The crude product (d.r. = 12:88) was purified by flash column chromatography on silica gel (EtOAc/Heptane, 5% to 30%) to afford **14h** (d.r. = 10:90, 22.8 mg, 76.1 µmol, 76%) as a colorless oil.

Data are reported for the major diastereoisomer.

^{1}H NMR (600 MHz, CDCl$_3$): δ = 7.28 – 7.24 (m, 2H), 7.20 – 7.14 (m, 3H), 5.69 (ddt, *J* = 16.6, 10.6, 6.1 Hz, 1H), 5.18 – 5.16 (m, 1H), 5.14 (app. dq, *J* = 8.6, 1.4 Hz, 1H), 3.90 (ddt, *J* = 15.2, 5.9, 1.3 Hz, 1H), 3.82 (dd, *J* = 15.2, 6.2 Hz, 1H), 3.27 (dd, *J* = 9.7, 6.9 Hz, 1H), 2.97 (dd, *J* = 9.7, 5.4 Hz, 1H), 2.70 – 2.59 (m, 2H), 2.43 (dd, *J* = 14.3, 7.7 Hz, 1H), 2.32 – 2.25 (m, 1H), 1.84 – 1.64 (m, 3H), 1.52 – 1.46 (m, 1H), 1.37 – 1.12 (m, 6H), 0.88 (t, *J* = 7.2 Hz, 3H);

^{13}C NMR (151 MHz, CDCl$_3$): δ = 176.4, 142.4, 132.9, 128.6 (2C), 128.4 (2C), 125.8, 117.9, 50.3, 45.5, 45.2, 36.1, 36.0, 29.9, 29.5, 27.6, 25.5, 22.9, 14.1;

FTIR (neat): 2927, 2857, 1684, 1489, 1434, 1415, 1264, 923, 745, 698 cm^{-1};

HRMS (ESI): Calculated for $C_{20}H_{29}NOH^+$ $[M+H]^+$: 300.2322, Found: 300.2323.

(±)-(3*S*,4*S*)-4-Isopropyl-1-phenyl-3-(3-phenylpropyl)pyrrolidin-2-one (14i)

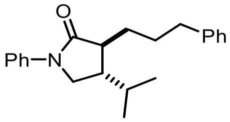

Formula: $C_{22}H_{27}NO$

MW = 321.5 g/mol

14i was prepared from (±)-(2*S*,3*S*)-2-isopropyl-3-(2-oxooxazolidine-3-carbonyl)-6-phenylhexanal (d.r. = 91:9, 20 mg, 60.3 µmol, 1 mmol) following the general procedure **E**. The crude product (d.r. = 87:12) was purified by flash column chromatography on silica gel (EtOAc/Heptane, 5% to 30%) to afford **14i** (d.r. = 90:10, 9.4 mg, 29.2 µmol, 49%) as a colorless oil.

Data are reported for the major diastereoisomer.

^{1}H NMR (600 MHz, $CDCl_3$): δ = 7.63 (t, *J* = 9.3 Hz, 2H), 7.36 (t, *J* = 8.0 Hz, 2H), 7.31 – 7.24 (m, 2H), 7.18 (dd, *J* = 14.5, 7.3 Hz, 3H), 7.13 (dd, *J* = 13.5, 6.2 Hz, 1H), 3.82 (dd, *J* = 9.5, 8.5 Hz, 1H), 3.50 (dd, *J* = 9.8, 5.8 Hz, 1H), 2.72 – 2.63 (m, 2H), 2.45 (q, *J* = 6.0 Hz, 1H), 2.01 (dq, *J* = 12.4, 6.2 Hz, 1H), 1.87 – 1.70 (m, 5H), 0.96 (d, *J* = 6.8 Hz, 3H), 0.94 (d, *J* = 6.7 Hz, 3H);

^{13}C NMR (151 MHz, $CDCl_3$): δ = 176.1, 142.3, 139.6, 128.9 (2C), 128.6 (2C), 128.5 (2C), 125.9, 124.5, 120.0 (2C), 49.9, 47.0, 42.2, 36.2, 31.4, 31.2, 28.8, 20.2, 19.0;

FTIR (neat): 3026, 2955, 2928, 2870, 1771, 1598, 1496, 1455, 1396, 1361, 1301, 1276, 1224, 1179, 1156, 1121, 1030 cm^{-1};

HRMS (ESI): Calculated for $C_{22}H_{27}NOH^+$ $[M+H]^+$: 322.2165, Found: 322.2165.

2.7 Non-reductive formation of lactams

(±)-(3*S*,4*S*)-4-Butyl-5-hydroxy-1-(4-methoxybenzyl)-4-methyl-3-(3-phenylpropyl)pyrrolidin-2-one (15)

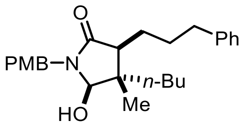

Formula: $C_{26}H_{35}NO_3$

MW = 409.6 g/mol

To a stirred suspension of (±)-(2*S*,3*S*)-2-butyl-2-methyl-3-(2-oxooxazolidine-3-carbonyl)-6-phenylhexanal (8.8 mg, 25 µmol, 1 equiv.) in a AcOH/PhMe mixture (1:1, 3 mL) was added 4-methoxybenzylamine (9.7 µL, 75 µmol, 3 equiv.) and the mixture was warmed at 50 °C. The mixture was stirred at the same temperature for 60 h. The mixture was allowed to cool to rt and was washed with water and sat. aq. $NaHCO_3$. The combined aqueous phases were extracted with CH_2Cl_2. The combined organic phases were dried over $mgSO_4$, filtered and concentrated *in vacuo*. The crude product (d.r. = 70:24:5:1) was purified by flash column chromatography on silica gel (EtOAc/heptane, 5% to 30%) to afford **15** (6.3 mg, 15 µmol, 62%) as one diastereoisomer, as a colorless oil.

^{1}H NMR (600 MHz, $CDCl_3$): δ = 7.29 – 7.26 (m, 2H), 7.22 – 7.15 (m, 5H), 6.83 (d, *J* = 8.6 Hz, 2H), 4.75 (d, *J* = 14.5 Hz, 1H), 4.54 (s, 1H), 4.10 (d, *J* = 14.5 Hz, 1H), 3.78 (s, 3H), 2.73 – 2.58 (m, 2H), 2.35 (t, *J* = 6.6 Hz, 1H), 2.07 – 1.97 (m, 1H), 1.87 (s, 1H), 1.78 – 1.67 (m, 2H), 1.42 – 1.35 (m, 1H), 1.18 – 1.12 (m, 1H), 1.11 – 1.06 (m, 1H), 1.05 (s, 3H), 1.04 – 1.01 (m, 1H), 1.01 – 0.97 (m, 1H), 0.97 – 0.91 (m, 1H), 0.81 – 0.75 (m, 1H), 0.72 (t, *J* = 7.3 Hz, 3H);

^{13}C NMR (151 MHz, $CDCl_3$): δ = 176.4, 159.3, 142.4, 130.1 (2C), 129.0, 128.6 (2C), 128.4 (2C), 125.8, 114.2 (2C), 85.8, 55.4, 49.8, 43.7, 43.6, 36.3, 34.3, 30.4, 26.4, 24.7, 23.4, 20.1, 14.0;

FTIR (neat): 3361, 2932, 2859, 1664, 1612, 1513, 1455, 1246, 1036, 745, 700 cm^{-1};

HRMS (ESI): Calculated for $C_{26}H_{35}NO_3Na^+$ $[M+Na]^+$: 432.2509, Found: 432.2508.

4-Butyl-1-(4-methoxybenzyl)-3-(3-phenylpropyl)-1,5-dihydro-2*H*-pyrrol-2-one (16)

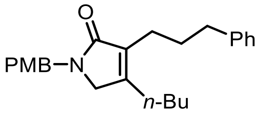

Formula: $C_{25}H_{31}NO_2$

MW = 377.2 g/mol

To a stirred suspension of (±)-(2*S*,3*S*)-2-butyl-3-(2-oxooxazolidine-3-carbonyl)-6-phenylhexanal (8.8 mg, 25 µmol, 1 equiv.) in a AcOH/PhMe mixture (1:1, 3 mL) was added 4-methoxybenzylamine (9.7 µL, 75 µmol, 3 equiv.) and the mixture was warmed at 50 °C. The mixture was stirred at the same temperature for 60 h. The mixture was allowed to cool to rt and was washed with water and sat. aq. $NaHCO_3$. The combined aqueous phases were extracted with CH_2Cl_2. The combined organic phases were dried over $mgSO_4$, filtered and concentrated *in vacuo*. The crude product (d.r. = 70:24:5:1) was purified by flash column chromatography on silica gel (EtOAc/heptane, 5% to 30%) to afford **16** (4.3 mg, 11.4 µmol, 45%) as a yellowish oil.

^{1}H NMR (600 MHz, $CDCl_3$): δ = 7.27 (d, *J* = 7.7 Hz, 2H), 7.20 (d, *J* = 7.2 Hz, 2H), 7.18-7.15 (m, 3H), 6.85 (d, *J* = 8.6 Hz, 2H), 4.54 (s, 2H), 3.79 (s, 3H), 3.60 (s, 2H), 2.66 (t, *J* = 7.8 Hz, 2H), 2.32 (t, *J* = 7.8 Hz, 2H), 2.26 (t, *J* = 7. 8 Hz, 2H), 1.85 (quint., *J* = 7.8 Hz, 2H), 1.39 – 1.32 (m, 2H), 1.31 – 1.24 (m, 2H), 0.87 (t, *J* = 7.2 Hz, 3H);

^{13}C NMR (151 MHz, $CDCl_3$): δ = 172.4, 159.1, 150.8, 142.4, 132.5, 130.0, 129.5 (2C), 128.6 (2C), 128.4 (2C), 125.8, 114.2 (2C), 55.4, 52.0, 45.6, 36.0, 30.9, 30.4, 27.5, 23.8, 22.8, 13.9;

FTIR (neat): 2953, 2930, 2858, 1677, 1612, 1513, 1456, 1246, 1034, 745, 700 cm^{-1};

HRMS (ESI): Calculated for $C_{25}H_{31}NO_2H^+$ $[M+H]^+$: 378.2428, Found: 378.2428.

2.8 Pictet-Spengler type annulation with tryptamines

2.8.1 General procedure F: Pictet-Spengler type annulation

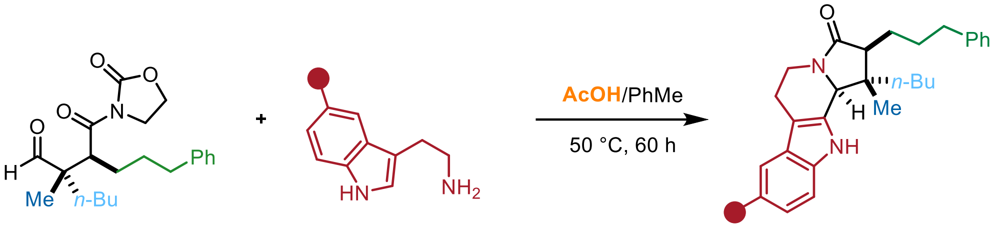

A vial was loaded with (±)-(2*R*,3*R*)-2-butyl-2-methyl-3-(2-oxooxazolidine-3-carbonyl)-6-phenylhexanal (18.0 mg, 50 µmol, 1 equiv.) dissolved in toluene/AcOH (1:1, 0.025 M). Then, the tryptamine derivative (150 µmol, 3.0 equiv.) was added and the resulting mixture was placed in a pre-heated sand bath of 110 °C. After 14 h, the vial was cooled to rt and carefully quenched with a saturated aqueous solution of $NaHCO_3$. Then, the aqueous phase was extracted with CH_2Cl_2 (2x). The organic phases were combined, dried over $mgSO_4$, filtered and the solvent removed under vacuum. The crude material was subjected to flash column chromatography on silica gel (Heptane/EtOAc) to yield the desired cyclized product.

2.8.2 Characterization of products

(±)-(1*R*,2*R*,11*bR*)-1-Butyl-1-methyl-2-(3-phenylpropyl)-1,2,5,6,11,11*b*-hexahydro-3*H*-indolizino[8,7-*b*]indol-3-one (18a)

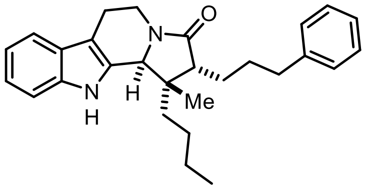

Formula: $C_{28}H_{34}N_2O$

MW = 414.6 g/mol

Following the general procedure **F** for the Pictet-Spengler-type cyclization using (±)-(2*R*,3*R*)-2-butyl-2-methyl-3-(2-oxooxazolidine-3-carbonyl)-6-phenylhexanal (18.0 mg, 50 µmol, 1 equiv.) and tryptamine (24 mg, 150 µmol, 3.0 equiv.), the titled compound was obtained after column chromatography (Heptane to Heptane/EtOAc (1:1)) as a colorless oil (18.1 mg, 45 µmol, 90%) as single diastereomer.

^{1}H NMR (600 MHz, $CDCl_3$): δ 7.82 (s, 1H), 7.51 (d, *J* = 7.8 Hz, 1H), 7.36 (d, *J* = 8.1 Hz, 1H), 7.30 – 7.26 (m, 2H), 7.23 – 7.16 (m, 4H), 7.13 (t, *J* = 7.4 Hz, 1H), 4.59 (s, 1H), 4.53 (dd, *J* = 11.9, 5.5 Hz, 1H), 2.90 – 2.83 (m,

2H), 2.80 – 2.73 (m, 1H), 2.69 (t, *J* = 7.4 Hz, 2H), 2.13 (dd, *J* = 10.9, 4.1 Hz, 1H), 2.08 – 2.01 (m, 1H), 1.85 – 1.78 (m, 1H), 1.76 – 1.70 (m, 1H), 1.66 – 1.49 (m, 3H), 1.42 – 1.25 (m, 3H), 1.12 – 1.04 (m, 1H), 0.92 (t, *J* = 7.2 Hz, 3H), 0.69 (s, 3H).

^{13}C NMR (151 MHz, $CDCl_3$): δ 175.8, 142.1, 136.5, 130.3, 128.7 (2C), 128.4 (2C), 127.0, 125.9, 122.3, 120.0, 118.4, 111.1, 111.0, 62.1, 52.8, 44.9, 37.2, 35.8, 35.3, 28.8, 26.8, 26.6, 23.6, 21.3, 21.2, 14.2.

FTIR (neat): 3338, 2956, 2926, 2859, 1667, 1467, 1445, 1422, 1467, 743 cm^{-1}.

HRMS (ESI): Calculated for $C_{28}H_{34}N_2ONa^+$ $[M+Na]^+$: 437.2563, Found: 437.2560.

(±)-(1*R*,2*R*,11b*R*)-1-Butyl-8-methoxy-1-methyl-2-(3-phenylpropyl)-1,2,5,6,11,11b-hexahydro-3*H*-indolizino[8,7-b]indol-3-one (18b)

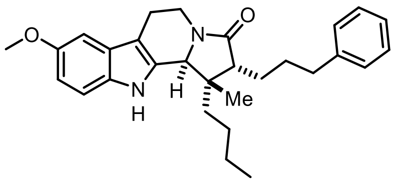

Formula: $C_{29}H_{36}N_2O_2$

MW = 444.6 g/mol

Following the general procedure **F** for the Pictet-Spengler-type cyclization using (±)-(2*R*,3*R*)-2-butyl-2-methyl-3-(2-oxooxazolidine-3-carbonyl)-6-phenylhexanal (18.0 mg, 50 µmol, 1 equiv.) and 5-methoxytryptamine (28.5 mg, 150 µmol, 3.0 equiv.), the titled compound was obtained after flash column chromatography on silica gel (Heptane/EtOAc, 0-50%) as a colorless oil (20.7 mg, 48 µmol, 96%) as single diastereomer.

^{1}H NMR (600 MHz, $CDCl_3$): δ 7.72 (d, *J* = 3.9 Hz, 1H), 7.27 (dd, *J* = 14.8, 6.9 Hz, 3H), 7.18 (dd, *J* = 17.9, 7.4 Hz, 3H), 6.95 (d, *J* = 2.1 Hz, 1H), 6.84 (dd, *J* = 8.7, 2.3 Hz, 1H), 4.57 (s, 1H), 4.52 (dd, *J* = 12.6, 5.2 Hz, 1H), 3.86 (s, 3H), 2.89 – 2.73 (m, 3H), 2.69 (t, *J* = 7.4 Hz, 2H), 2.12 (dd, *J* = 10.9, 4.0 Hz, 1H), 2.02 (d, *J* = 6.9 Hz, 1H), 1.80 (d, *J* = 3.0 Hz, 1H), 1.75 – 1.69 (m, 1H), 1.67 – 1.51 (m, 3H), 1.41 – 1.25 (m, 3H), 1.10 – 1.02 (m, 1H), 0.91 (t, *J* = 7.2 Hz, 3H), 0.68 (s, 3H);

^{13}C NMR (151 MHz, CDCl$_3$): δ 175.8, 154.5, 142.1, 131.5, 131.1, 128.7 (2C), 128.4 (2C), 127.4, 125.9, 112.2, 111.8, 110.9, 100.4, 62.1, 56.0, 52.8, 45.0, 37.2, 35.8, 35.2, 28.8, 26.8, 26.6, 23.6, 21.3 (2C), 14.2;

FTIR (neat): 3363, 1671, 1444, 1421, 1214, 700, 614 cm^{-1};

HRMS (ESI): Calculated for $C_{29}H_{36}N_2O_2Na^+$ [M+Na]$^+$: 467.2669, Found: 467.2673.

(±)-(1*R*,2*R*,11b*R*)-8-Bromo-1-butyl-1-methyl-2-(3-phenylpropyl)-1,2,5,6,11,11b-hexahydro-3*H*-indolizino[8,7-b]indol-3-one (18c)

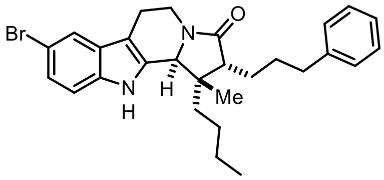

Formula: $C_{28}H_{33}BrN_2O$

MW = 493.5 g/mol

Following the general procedure **F** for the Pictet-Spengler-type cyclization using (±)-(2*R*,3*R*)-2-butyl-2-methyl-3-(2-oxooxazolidine-3-carbonyl)-6-phenylhexanal (18.0 mg, 50 µmol, 1 equiv.) and 5-bromotryptamine (35.9 mg, 0.15 mmol, 3 equiv.), the titled compound was obtained after column chromatography (heptane/EtOAc, 0-50%) as a white solid (20.6 mg, 43 µmol, 86%) as single diastereomer.

^{1}H NMR (600 MHz, CDCl$_3$): δ 7.94 (s, 1H), 7.62 (s, 1H), 7.28 – 7.25 (m, 3H), 7.22 (d, *J* = 8.6 Hz, 1H), 7.20 – 7.14 (m, 3H), 4.56 (s, 1H), 4.51 (dd, *J* = 12.8, 5.3 Hz, 1H), 2.87 – 2.76 (m, 2H), 2.74 – 2.66 (m, 3H), 2.12 (dd, *J* = 11.0, 4.0 Hz, 1H), 2.04 – 1.97 (m, 1H), 1.84 – 1.77 (m, 1H), 1.76 – 1.68 (m, 1H), 1.69 – 1.49 (m, 3H), 1.41 – 1.27 (m, 3H), 1.08 – 1.00 (m, 1H), 0.91 (t, *J* = 7.2 Hz, 3H), 0.66 (s, 3H);

^{13}C NMR (151 MHz, CDCl$_3$): δ 175.7, 142.1, 135.1, 131.7, 128.7 (2C), 128.6, 128.4 (2C), 125.9, 125.1, 121.1, 113.2, 112.5, 110.7, 62.0, 52.8, 45.0, 37.1, 35.8, 35.2, 28.8, 26.8, 26.6, 23.6, 21.3, 21.1, 14.2;

FTIR (neat): 3264, 2927, 2857, 1709, 1660, 1456, 1221, 739, 698 cm^{-1};

HRMS (ESI): Calculated for $C_{28}H_{33}BrN_2ONa^+$ [M+Na]$^+$: 517.1648, Found: 517.1644.

2.9 Total synthesis of paraconic acids

2.9.1 Synthesis of Me-ynamide (1b)

3-(Prop-1-yn-1-yl)oxazolidin-2-one (1b)

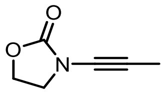

Formula: $C_6H_7NO_2$

MW = 125.1 g/mol

The title compound was prepared in 77% yield according to a procedure described in the literature.[5]

^{1}H NMR (400 MHz, CDCl$_3$): δ 4.45 – 4.36 (m, 2H), 3.89 – 3.77 (m, 2H), 1.93 (s, 3H);

^{13}C NMR (101 MHz, CDCl$_3$): δ 156.9, 69.2, 66.9, 62.9, 47.0, 3.4.

All NMR data were in accordance with the literature described above.

2.9.2 Synthesis of E-vinyl sulfoxides (2j)

(*E*)-1-Methoxy-4-[2-(*p*-tolylsulfinyl)vinyl]benzene (2j)

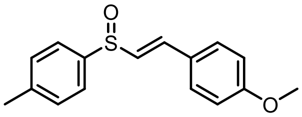

Formula: $C_{16}H_{16}O_2S$

MW = 272.4 g/mol

The title compound was prepared in 75% yield according to a procedure described in the literature.[6]

^{1}H NMR (400 MHz, CDCl$_3$): δ 7.56 (d, *J* = 8.2 Hz, 2H), 7.42 – 7.37 (m, 2H), 7.33 – 7.28 (m, 3H), 6.90 – 6.86 (m, 2H), 6.67 (d, *J* = 15.5 Hz, 1H), 3.82 (s, 3H), 2.40 (s, 3H);

^{13}C NMR (101 MHz, CDCl$_3$): δ 161.1, 141.7, 141.2, 136.7, 130.7, 130.2 (2C), 129.5 (2C), 126.7, 125.0 (2C), 114.5 (2C), 55.5, 21.6.

All NMR data were in accordance with the literature.

(*R*,*E*)-1-Methoxy-4-[2-(*p*-tolylsulfinyl)vinyl]benzene (2j)

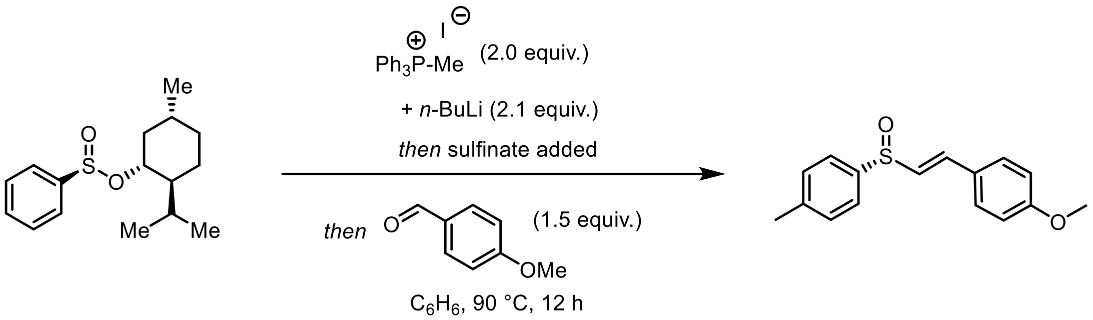

An adapted procedure from the literature was used to provide the desired compound in 48% yield.[7]

A flame-dried Schlenk flask under argon was loaded with methyltriphenylphosphonium iodide (2.4 g, 6.0 mmol, 2 equiv.) and benzene (50 mL) was added *via* syringe. Carefully, *n*-BuLi (1.6 M in hexane, 4.1 mL, 6.6 mmol, 2.2 equiv.) was added. The resulting mixture was stirred for 1 h at room temperature. Then, (1*R*,2*S*,5*R*)-(–)-menthyl (*S*)-*p*-toluenesulfinate was added in one portion and the solution was stirred for another 1h at room temperature. Subsequently, *p*-anisaldehyde (0.55 mL, 4.5 mmol, 1.5 equiv.) was added and the mixture refluxed (90 °C, oil bath) for 10 h. After cooling to rt, an aqueous solution of HCl (0.2M) was added to reach a pH value of 3-4. The aqueous phase was extracted with CH_2Cl_2 (3x). The combined organic phases were dried over $mgSO_4$, filtered and concentrated under reduced pressure. The crude material was purified by flash column chromatography on silica gel (Heptane/EtOAC, 0-70%) to obtain the titled compound as a white solid (388 mg, 1.42 mmol, 48%).

All NMR data was in accordance with the data described above.

Enantiomeric excess: 99% e.e. was determined by chiral HPLC analysis: Chiralpak IC, *n*-heptane+0.1%IPA/IPA 7:3, 1 mL/min, 25 °C, detection at 210 nm, retention time (min): 29.2 (major) and 25.8 (minor)

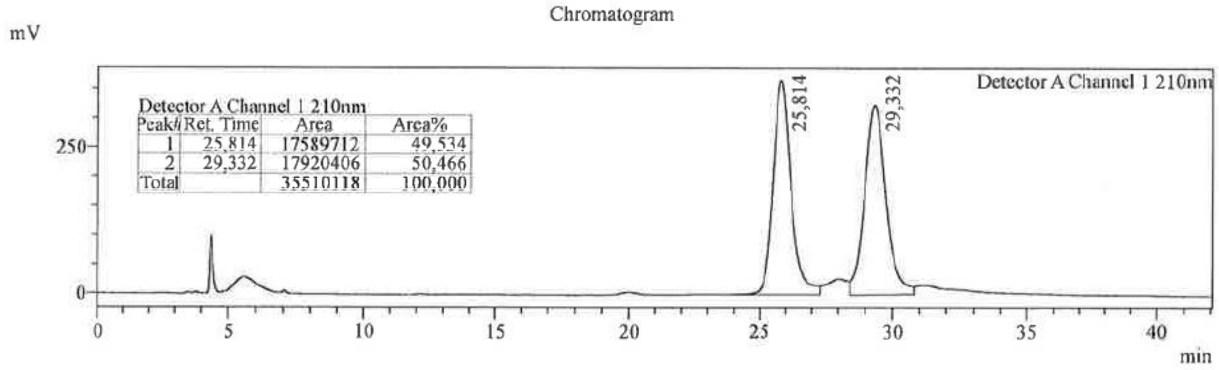

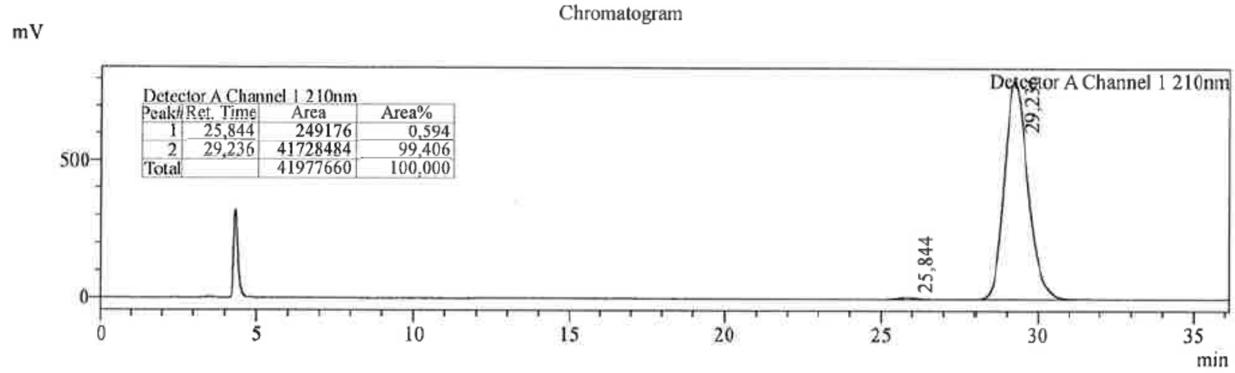

2.9.3 Synthesis of 1,4-dicarbonyls (**19**)

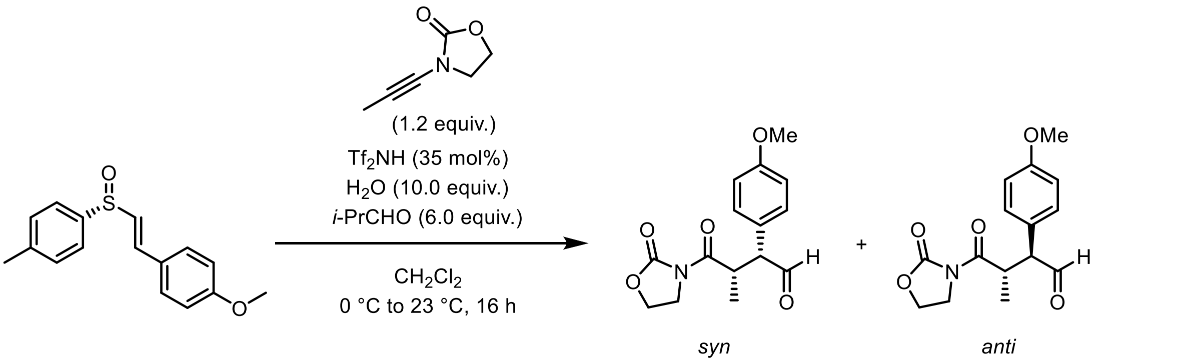

To a solution of the sulfoxide (436 mg, 1.6 mmol, 1 equiv.), ynamide (240 mg, 1.92 mmol, 1.2 equiv.) and *i*-PrCHO (0.88 mL, 9.6 mmol, 6 equiv.) in CH_2Cl_2 (16 mL, 0.1 M) was added water (288 µL, 16 mmol, 10 equiv.) under vigorous stirring and the resulting mixture was cooled to 0 °C. A solution of Tf_2NH (5.6 mL, 0.1 M in CH_2Cl_2, 35 mol%) was added over 30 min using a syringe pump. After the addition was complete, the mixture was left stirring at the same temperature for 16 h before being quenched with aq. $NaHCO_3$

(made by mixing 1:1 volume of water and sat. aq. $NaHCO_3$). The phases were separated and the aqueous phase was extracted with CH_2Cl_2. The combined organic phases were dried over mgSO$_4$, filtered and carefully concentrated *in vacuo*. The crude product was purified by flash column chromatography on silica gel (Heptane to EtOAc/Heptane (3:2). The desired syn-1,4-dicarbonyl was obtained as a white solid (203 mg, 0.70 mmol, 44%), which was separated by column chromatography from the *anti*-isomer (63 mg, 0.22 mmol, 14%), also a white solid.

(2*R*,3*S*)-2-(4-Methoxyphenyl)-3-methyl-4-oxo-4-(2-oxooxazolidin-3-yl)butanal (*syn*-19)

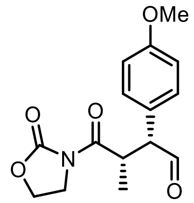

Formula: $C_{15}H_{17}NO_5$

MW = 291.3 g/mol

^{1}H NMR (600 MHz, CDCl$_3$): δ 9.59 (s, 1H), 7.18 – 7.12 (m, 2H), 6.98 – 6.92 (m, 2H), 4.54 – 4.40 (m, 2H), 4.25 (dq, *J* = 10.9, 7.0 Hz, 1H), 4.14 – 4.02 (m, 3H), 3.84 (s, 3H), 1.02 (d, *J* = 7.0 Hz, 3H);

^{13}C NMR (151 MHz, CDCl$_3$): δ 199.2, 176.8, 159.7, 153.3, 131.1 (2C), 124.6, 114.9 (2C), 62.1, 61.6, 55.4, 42.9, 38.1, 15.6;

FTIR (neat): 1768, 1719, 1698, 1514, 1388, 1250, 1226, 1179, 1031 cm^{-1};

HRMS (ESI): Calculated for $C_{15}H_{17}NO_5Na^+$ $[M+Na]^+$: 314.0999, Found: 341.1001;

Enantiomeric excess: 97% e.e. was determined by chiral HPLC analysis: Chiralpak IC, *n*-heptane+0.1%IPA/IPA 8:2, 1 mL/min, 25 °C, detection at 210 nm, retention time (min): 14.6 (major) and 19.0 (minor).

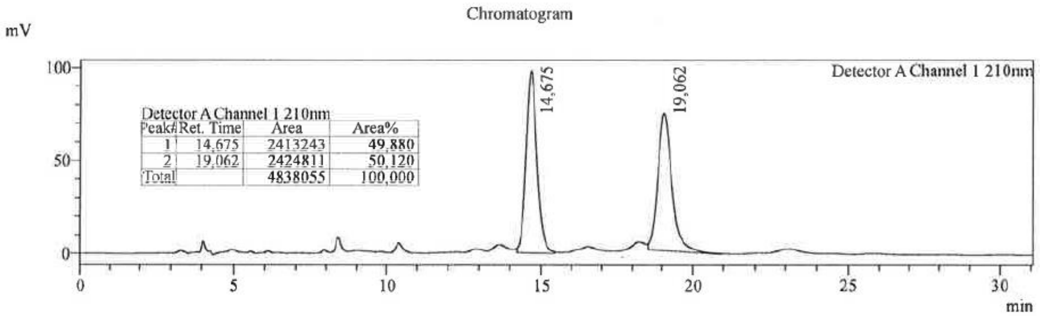

Peak#	Ret. Time	Area	Area%
1	14,675	2413243	49,880
2	19,062	2424811	50,120
Total		4838055	100,000

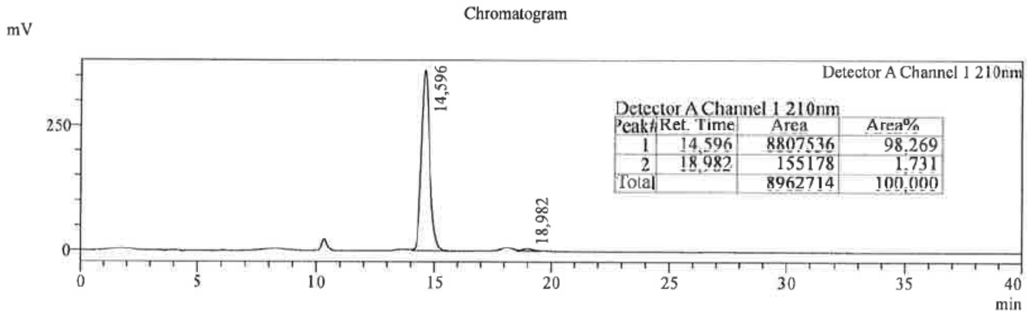

Peak#	Ret. Time	Area	Area%
1	14,596	8807536	98,269
2	18,982	155178	1,731
Total		8962714	100,000

(2*S*,3*S*)-2-(4-Methoxyphenyl)-3-methyl-4-oxo-4-(2-oxooxazolidin-3-yl)butanal (*anti*-19)

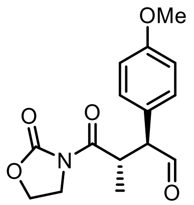

Formula: $C_{15}H_{17}NO_5$

MW = 291.3 g/mol

^{1}H NMR (400 MHz, $CDCl_3$): δ 9.67 (d, *J* = 2.5 Hz, 1H), 7.16 (d, *J* = 8.7 Hz, 2H), 6.86 (d, *J* = 8.8 Hz, 2H), 4.83 – 4.66 (m, 1H), 4.33 – 4.22 (m, 1H), 4.18 – 4.07 (m, 1H), 3.90 – 3.84 (m, 2H), 3.78 (s, 3H), 3.67 – 3.57 (m, 1H), 1.32 (d, *J* = 6.7 Hz, 3H);

^{13}C NMR (176 MHz, $CDCl_3$): δ 198.1, 175.1, 159.5, 153.1, 130.7 (2C), 125.9, 114.6 (2C), 61.9, 61.2, 55.4, 42.7, 38.3, 16.5;

FTIR (neat): 1769, 1717, 1693, 1510, 1383, 1248, 1219, 1179, 1029, 830 cm^{-1};

HRMS (ESI): Calculated for $C_{15}H_{17}NO_5Na^+$ $[M+Na]^+$: 314.0999, Found: 341.1002.

2.9.4 Synthesis of lactones (20, 21)

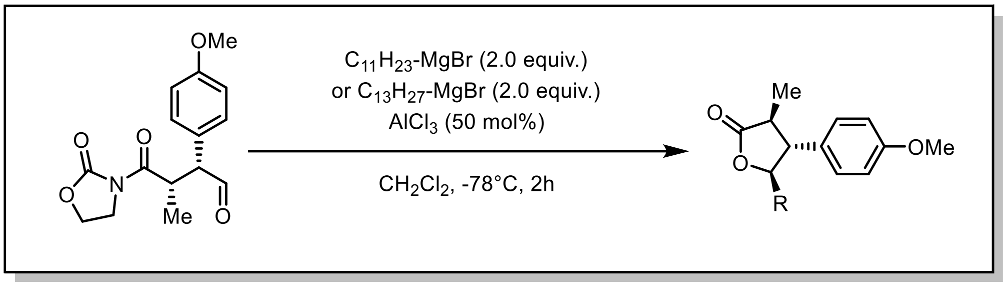

For this step the general Procedure C for the formation of γ-C lactones was followed. The crude materials were purified by column chromatography (heptane/EtOAc, 0-20%).

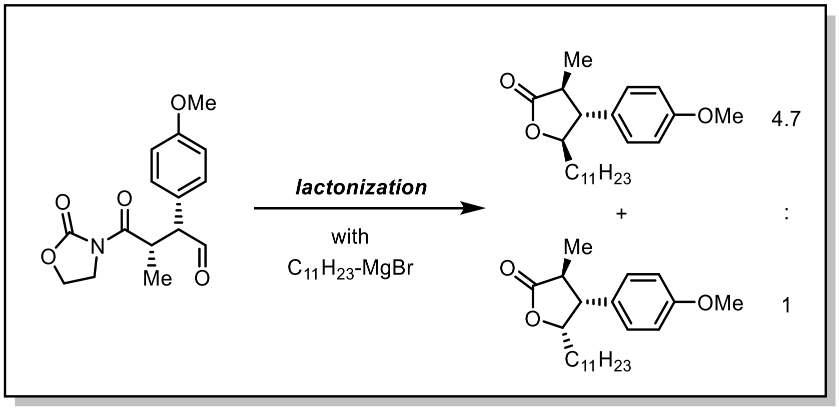

The general procedure was followed (0.12 mmol scale) to give the desired titled compound (29 mg, 80 µmol, 67%) as white solid in a d.r. of 4.7:1. The isomers were separated using HPLC (see below for exact conditions information).

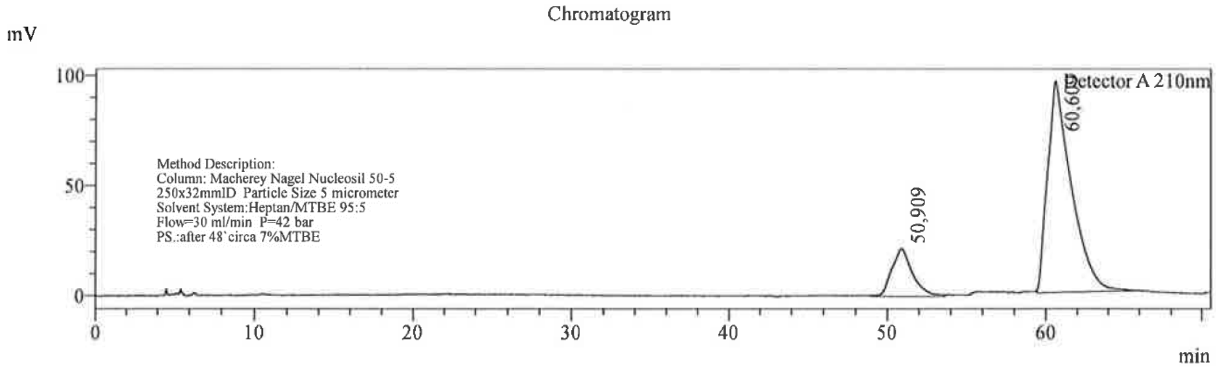

Major isomer:

(3*S*,4*R*,5*R*)-4-(4-Methoxyphenyl)-3-methyl-5-undecyldihydrofuran-2(3*H*)-one (20a)

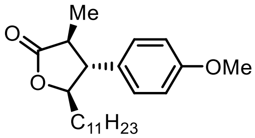

Formula: $C_{23}H_{36}O_3$

MW = 360.5 g/mol

All NMR data were in good accordance with the literature.[8]

^{1}H NMR (700 MHz, $CDCl_3$): δ 7.16 – 7.13 (m, 2H), 6.92 – 6.89 (m, 2H), 4.34 – 4.24 (m, 1H), 3.81 (s, 3H), 2.77 – 2.67 (m, 2H), 1.62 (m, 3H), 1.51 – 1.45 (m, 1H), 1.32 – 1.17 (m, 19H), 0.88 (t, *J* = 7.1 Hz, 3H);

^{13}C NMR (176 MHz, $CDCl_3$): δ 178.3, 159.3, 129.4, 128.8 (2C), 114.6 (2C), 84.6, 56.2, 55.4, 43.9, 33.6, 32.0, 29.8, 29.7 (2C), 29.6, 29.5 (2C), 25.9, 22.8, 14.3, 13.3;

FTIR (neat): 2924, 2854, 2770, 1514, 1458, 1251, 1225, 1179, 1034, 828 cm^{-1};

HRMS (ESI): Calculated for $C_{23}H_{36}O_3Na^+$ $[M+Na]^+$: 383.2557, Found: 383.2557;

Enantiomeric excess: 95% e.e. was determined by chiral HPLC analysis: Chiralpak IC, *n*-heptane+0.1%IPA/IPA 95:5, 1 mL/min, 25 °C, detection at 230 nm, retention time (min): 4.4 (major) and 5.6 (minor).

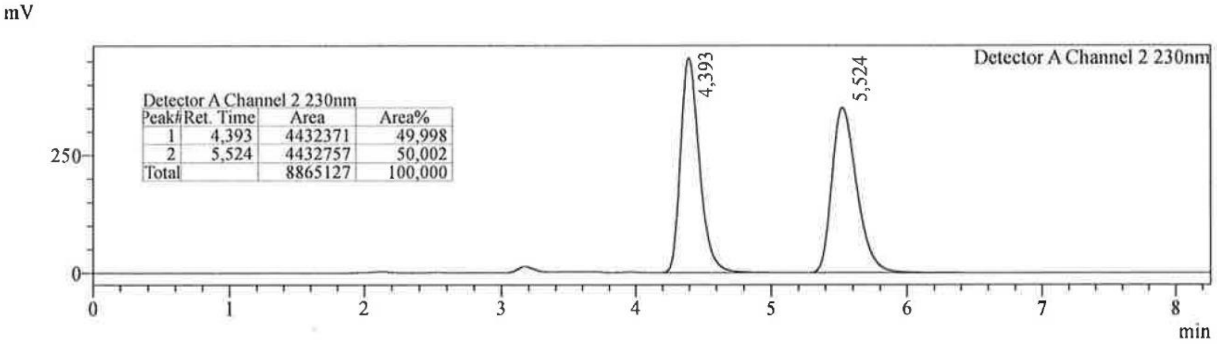

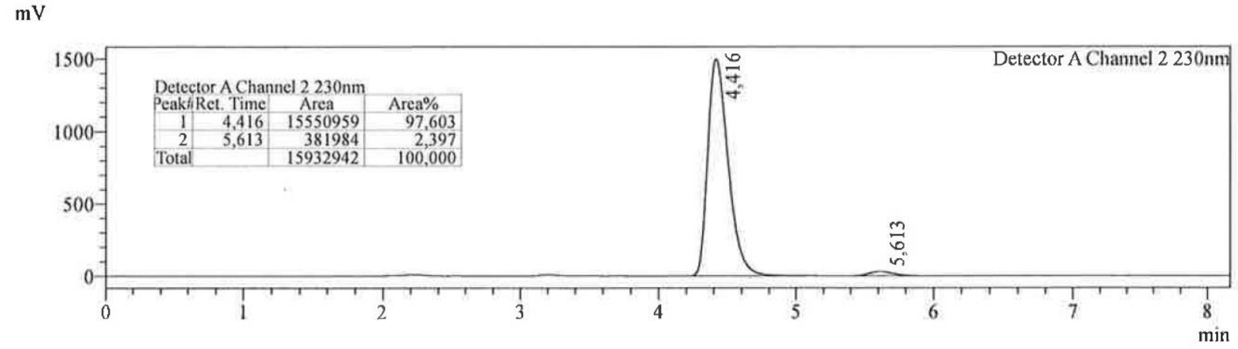

Minor isomer:

(3*S*,4*R*,5*S*)-4-(4-Methoxyphenyl)-3-methyl-5-undecyldihydrofuran-2(3*H*)-one (20b)

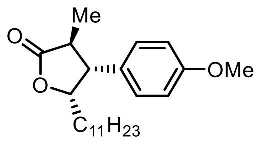

Formula: $C_{23}H_{36}O_3$

MW = 360.5 g/mol

^{1}H NMR (700 MHz, CDCl$_3$): δ 7.09 – 7.06 (m, 2H), 6.92 – 6.88 (m, 2H), 4.65 – 4.56 (m, 1H), 3.82 (s, 3H), 3.42 (dd, *J* = 10.6, 7.7 Hz, 1H), 2.96 – 2.90 (m, 1H), 1.29 – 1.22 (m, 23H), 0.87 (t, *J* = 7.2 Hz, 3H);

^{13}C NMR (176 MHz, CDCl$_3$): δ 179.3, 159.1, 129.0, 128.4 (2C), 114.4 (2C), 82.1, 55.4, 51.8, 38.3, 32.1, 31.1, 29.8 (2C), 29.7, 29.6, 29.5, 29.4, 26.0, 22.8, 14.3, 14.2;

FTIR (neat): 2954, 2922, 2853, 1775, 1515, 1462, 1253, 1180 cm^{-1};

HRMS (ESI): Calculated for $C_{23}H_{36}O_3Na^+$ $[M+Na]^+$: 383.2557, Found: 383.2556;

Enantiomeric excess: 97% e.e. was determined by chiral HPLC analysis: Chiralpak IC, *n*-heptane+0.1%IPA/IPA 95:5, 1 mL/min, 25 °C, detection at 230 nm, retention time (min): 4.6 (major) and 6.9 (minor).

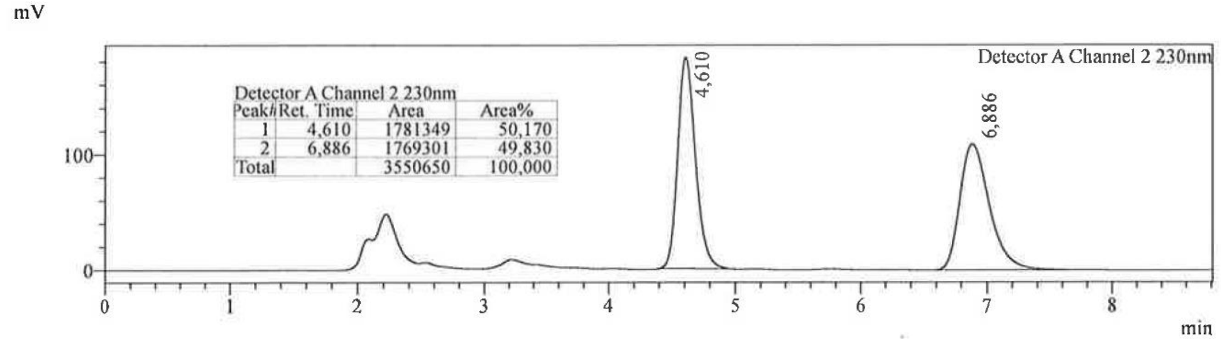

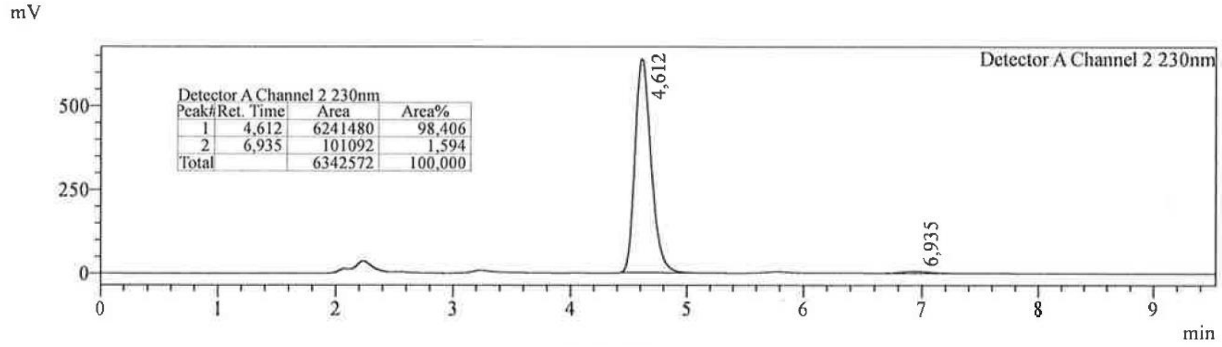

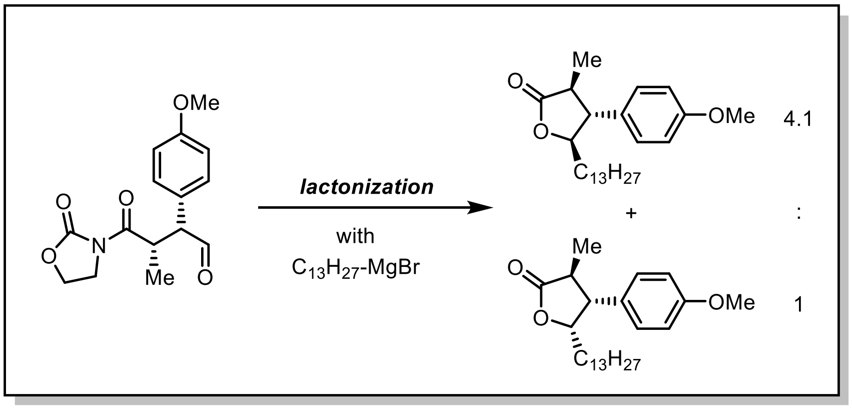

The general procedure was followed (0.12 mmol scale), to give the desired titled compound (26.5 mg, 68 µmol, 57%) as white solid in a d.r. of 4.1:1. The isomers were separated using HPLC (see below for exact conditions information).

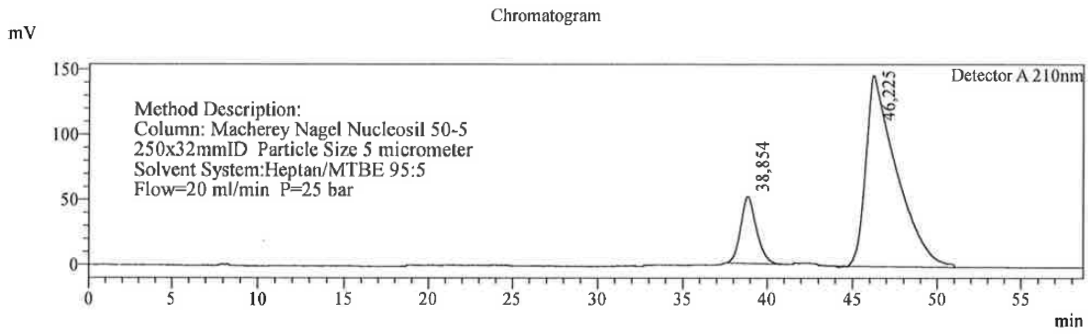

Major isomer:

(3*S*,4*R*,5*R*)-4-(4-Methoxyphenyl)-3-methyl-5-tridecyldihydrofuran-2(3*H*)-one (21a)

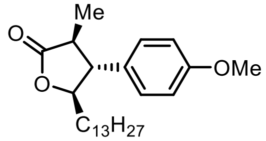

Formula: $C_{25}H_{40}O_3$

MW = 388.6 g/mol

^{1}H NMR (600 MHz, $CDCl_3$): δ 7.15 (d, *J* = 8.7 Hz, 2H), 6.91 (d, *J* = 8.7 Hz, 2H), 4.35 – 4.26 (m, 1H), 3.81 (s, 3H), 2.76 – 2.71 (m, 2H), 1.65 – 1.44 (m, 3H), 1.31 – 1.17 (m, 24H), 0.87 (t, *J* = 7.0 Hz, 3H);

^{13}C NMR (151 MHz, $CDCl_3$): δ 178.3, 159.3, 129.4, 128.8 (2C), 114.6 (2C), 84.6, 56.2, 55.4, 43.9, 33.6, 32.1, 29.9, 29.8 (2C), 29.7, 29.6, 29.52, 29.49, 29.46, 25.9, 22.8, 14.3, 13.3;

FTIR (neat): 2922, 2852, 1776, 1514, 1462, 1251, 1178, 1036, 828 cm^{-1};

HRMS (ESI): Calculated for $C_{25}H_{40}O_3Na^+$ $[M+Na]^+$: 411.2870, Found: 411.2870;

Enantiomeric excess: 95% e.e. was determined by chiral HPLC analysis: Chiralpak IC, 50% (60% *n*-Heptane + 5% EtOH + 35% MTBE):50% (n-Heptane + 0.1%IPA), 1 mL/min, 25 °C, detection at 230 nm, retention time (min): 3.3 (major) and 3.7 (minor).

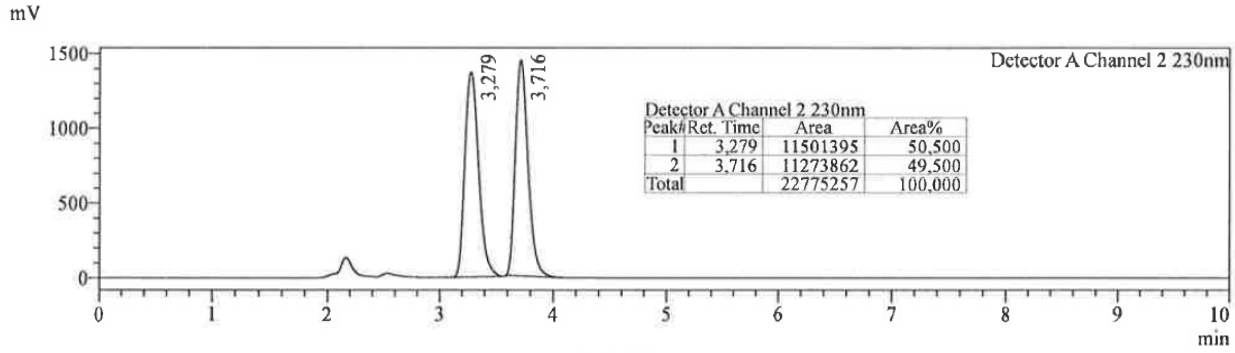

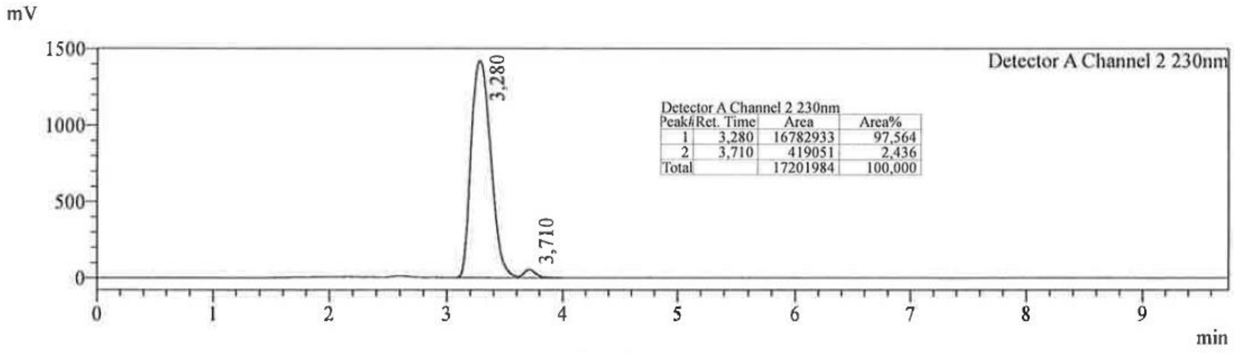

<u>Minor isomer:</u>

(3*S*,4*R*,5*S*)-4-(4-Methoxyphenyl)-3-methyl-5-tridecyldihydrofuran-2(3*H*)-one (21b)

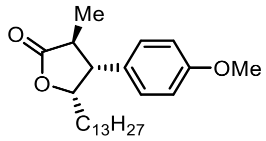

Formula: $C_{25}H_{40}O_3$

MW = 388.6 g/mol

^{1}H NMR (600 MHz, CDCl$_3$): δ 7.10 – 7.05 (m, 2H), 6.91 – 6.87 (m, 2H), 4.64 – 4.53 (m, 1H), 3.82 (s, 3H), 3.42 (dd, *J* = 10.6, 7.7 Hz, 1H), 2.92 (dq, *J* = 10.7, 7.1 Hz, 1H), 1.41 – 1.17 (m, 27H), 0.88 (t, *J* = 7.1 Hz, 3H);

^{13}C NMR (151 MHz, CDCl$_3$): δ 179.3, 159.1, 129.0 (2C), 128.4, 114.4 (2C), 82.1, 55.4, 51.8, 38.3, 32.1, 31.1, 29.9, 29.8 (2C), 29.7, 29.6 (2C), 29.5, 29.4, 26.0, 22.8, 14.3, 14.2;

FTIR (neat): 2921, 2852, 1779, 1515, 1461, 1377, 1253, 1179, 1040, 993, 721 cm^{-1};

HRMS (ESI): Calculated for $C_{25}H_{40}O_3Na^+$ $[M+Na]^+$: 411.2870, Found: 411.2867;

Enantiomeric excess: 97% e.e. was determined by chiral HPLC analysis: Chiralpak IC, 50% (60% *n*-Heptane + 5% EtOH + 35% MTBE):50% (n-Heptane + 0.1%IPA), 1 mL/min, 25 °C, detection at 230 nm, retention time (min): 3.3 (major) and 4.3 (minor).

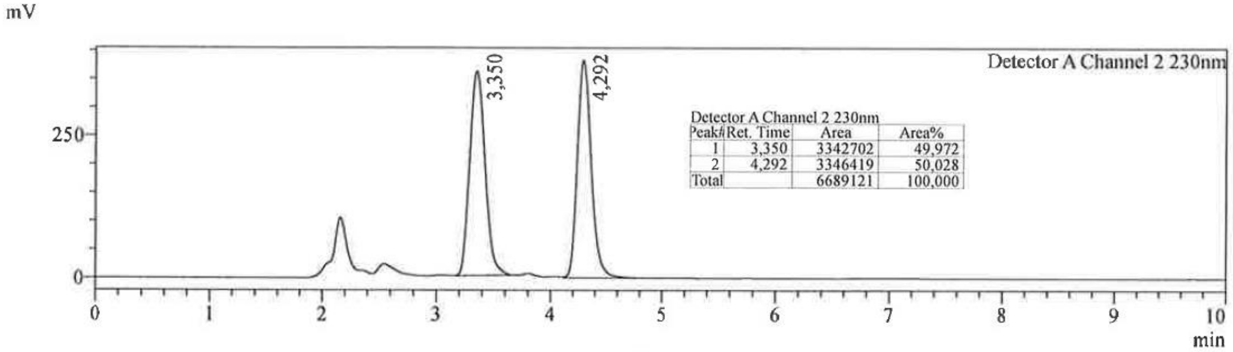

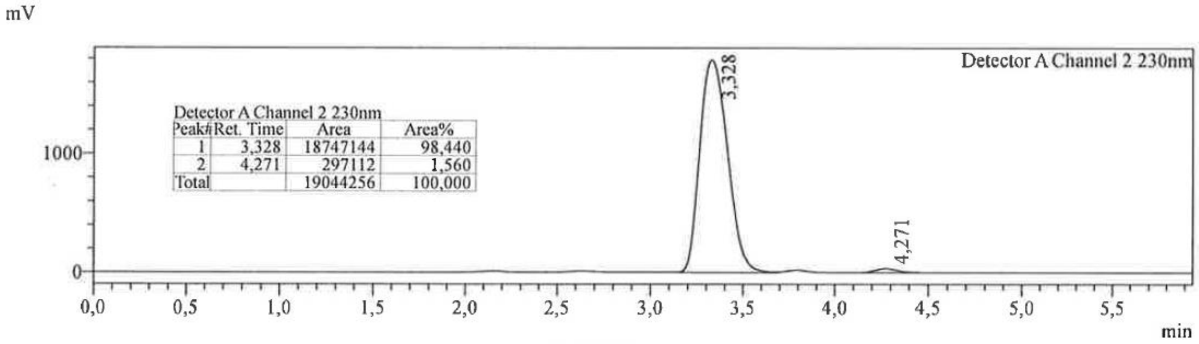

2.9.5 Synthesis of Paraconic Acids

General procedure

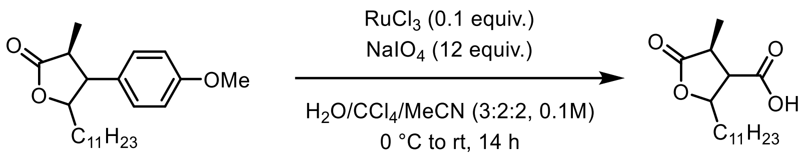

A procedure from literature was slightly modified.[8] To the corresponding lactone dissolved in a solvent mixture of H_2O, MeCN and CCl_4 (3:2:2, 0.1 M) was added $NaIO_4$ (12 equiv.). The resulting mixture was cooled to 0 °C, and $RuCl_3$ (0.1 equiv.) was added and a black colored solution was quickly observed. The cooling bath was removed after 30 min, and the reaction was further stirred at rt for 16 h. Then, the mixture was transferred to a separatory funnel. The aqueous phase was washed with CH_2Cl_2 and EtOAC (2x). The combined organic phases were dried over $mgSO_4$, filtered and concentrated under reduced pressure. The crude material was further purified by flash column chromatography on silica gel (heptane/Et_2O (1:1), 2% HOAc) to provide the corresponding desired acid products.

(+)-Nephrosteranic acid

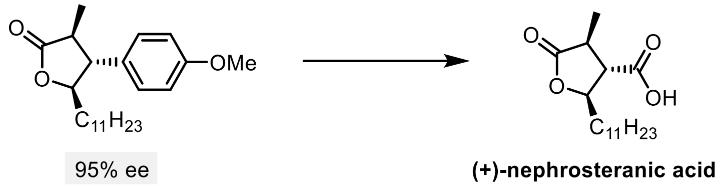

Formula: $C_{17}H_{30}O_4$

MW = 298.4 g/mol

The general procedure was followed using the corresponding lactone **20a** (22 mg, 61 µmol, 1 equiv.). The titled compound was obtained as a white solid (15 mg, 50 µmol, 82%).

All NMR data was in good accordance to the literature.[9]

^{1}H NMR (500 MHz, $CDCl_3$): δ 4.48 (td, *J* = 8.8, 3.9 Hz, 1H), 3.03 – 2.92 (m, 1H), 2.70 (dd, *J* = 11.4, 9.5 Hz, 1H), 1.85 – 1.78 (m, 1H), 1.77 – 1.66 (m, 1H), 1.58 – 1.47 (m, 1H), 1.43 – 1.25 (m, 20H), 0.88 (t, *J* = 7.0 Hz, 3H); *(proton of the carboxylic acid was not visible)*

^{13}C NMR (126 MHz, $CDCl_3$): δ 176.7, 175.9, 79.5, 54.0, 40.0, 35.1, 32.1, 29.8 (2C), 29.7, 29.5 (2C), 29.4, 25.5, 22.8, 14.7, 14.3;

FTIR (neat): 2917, 2850, 1730, 1247, 1175, 978, 697 cm^{-1};

HRMS (ESI): Calculated for $C_{17}H_{30}O_4Na^+$ $[M+Na]^+$: 321.2036, Found: 321.2041;

$[\alpha]^{20}_D$ +24.7 (c 0.4, $CHCl_3$), lit.[9] $[\alpha]_D^{23}$ +23.3 (c = 0.2, $CHCl_3$ for 96% ee).

(+)-Rocellaric acid

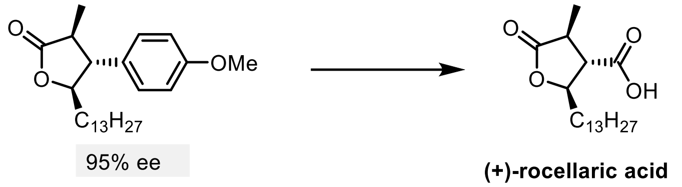

The general procedure was followed using the corresponding lactone **21a** (22 mg, 52 µmol, 1 equiv.). The titled compound was obtained as a white solid (13.1 mg, 40 µmol, 79%).

All NMR data was in good accordance to the literature.[9]

Formula: $C_{19}H_{34}O_4$

MW = 326.5 g/mol

^{1}H NMR (500 MHz, $CDCl_3$): δ 4.48 (td, J = 8.8, 3.9 Hz, 1H), 3.01 – 2.92 (m, 1H), 2.70 (dd, J = 11.1, 9.6 Hz, 1H), 1.87 – 1.78 (m, 1H), 1.74 – 1.67 (m, 1H), 1.56 – 1.49 (m, 1H), 1.43 – 1.25 (m, 24H), 0.88 (t, J = 6.9 Hz, 3H); *(proton of the carboxylic acid was not visible)*

^{13}C NMR (126 MHz, $CDCl_3$): δ 176.7, 176.0, 79.5, 54.0, 40.0, 35.1, 32.1, 29.8 (4C), 29.7, 29.5 (2C), 29.4, 25.5, 22.8, 14.7, 14.3;

FTIR (neat): 2953, 2917, 2850, 1745, 1715, 1469, 1255, 1203, 1171, 972, 697 cm^{-1};

HRMS (ESI): Calculated for $C_{19}H_{34}O_4Na^+$ $[M+Na]^+$: 349.2349, Found: 349.2348;

$[\alpha]^{20}_D$ +21.7 (c = 1.1, $CHCl_3$), lit.[9] $[\alpha]_D^{22}$ +16.7 (c = 0.2, $CHCl_3$ for 96% ee).

(–)-Nephromopsinic acid

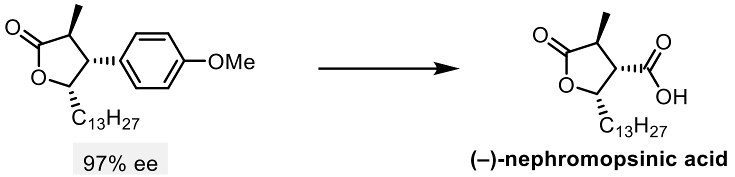

Formula: $C_{19}H_{34}O_4$

MW = 326.5 g/mol

The general procedure was followed using the corresponding lactone **21b** (5.0 mg, 13 µmol, 1 equiv.). The titled compound was obtained as a white solid (4.0 mg, 12 µmol, 91%).

All NMR data was in good accordance to the literature.[9]

^{1}H NMR (600 MHz, $CDCl_3$): δ 4.75 – 4.58 (m, 1H), 3.21 (t, *J* = 8.6 Hz, 1H), 3.04 (td, *J* = 9.8, 7.2 Hz, 1H), 1.63 – 1.48 (m, 3H), 1.41 – 1.25 (m, 24H), 0.88 (d, *J* = 6.9 Hz, 3H); *(proton of the carboxylic acid was not visible)*

^{13}C NMR (151 MHz, $CDCl_3$): δ 177.5, 173.6, 77.4, 51.6, 36.6, 32.1, 31.3, 29.8 (4C), 29.7, 29.6, 29.5, 29.4, 25.8, 22.8, 14.6, 14.3;

FTIR (neat): 2954, 2919, 2851, 1744, 1466, 1200, 1180, 696 cm^{-1};

HRMS (ESI): Calculated for $C_{19}H_{34}O_4Na^+$ $[M+Na]^+$: 349.2349, Found: 349.2348;

$[\alpha]^{20}_D$ –76.0 (c = 0.25, $CHCl_3$), lit.[9] $[\alpha]_D^{23}$ –85.7 (c = 0.5, $CHCl_3$ for 95% ee).

3 DFT study

3.1 Computational details

Preliminary calculations of the considered transition states were performed at the B3LYP-D3BJ-SMD(DCM)/def2-TZVP//B3LYP-D3BJ-SMD(DCM)/def2-SVP[10–16] level of theory. The obtained structures were used for further conformational search exploration and subsequent quantum chemical re-optimization (vide infra).

The conformational space of all molecules has been searched using meta-dynamics simulations based on tight-binding quantum chemical calculations as implemented in CREST.[17,18] The structures located with the CREST have then been subjected to PBE0-D3BJ/def2-SVP[12,19] (first refinement of the conformational space) and, at the next stage, to the PBE0-D3BJ/def2-TZVP geometry optimization. The nature of all stationary points (minima and transition states) was verified through the computation of the vibrational frequencies at the level applied for the geometry optimization. The thermal corrections to the Gibbs free energy were used to obtain final Gibbs free energies (G_{273}) at 273.15 K, with quasi-harmonic correction (frequency cut-off of 100.0 wavenumbers) and concentration correction to 1 M performed using the GoodVibes program developed by the Paton group.[20] All energies are reported in kcal mol^{-1}. The polarizable continuum model (PCM) with SMD parameters[21,22] was applied to consider solvent (DCM) effects. The DFT calculations have been performed with the Gaussian 16 program package.[23]

3.2 Kinetics of alternative diastereoselective pathways – computation of transition states

Figure S1 presents the computed relative Gibbs free energies ($\Delta\Delta G^{\ddagger}_{273}$) for possible transition states of all considered reactions. The most stable transition state of each reaction serves as a reference (0.0 kcal/mol).

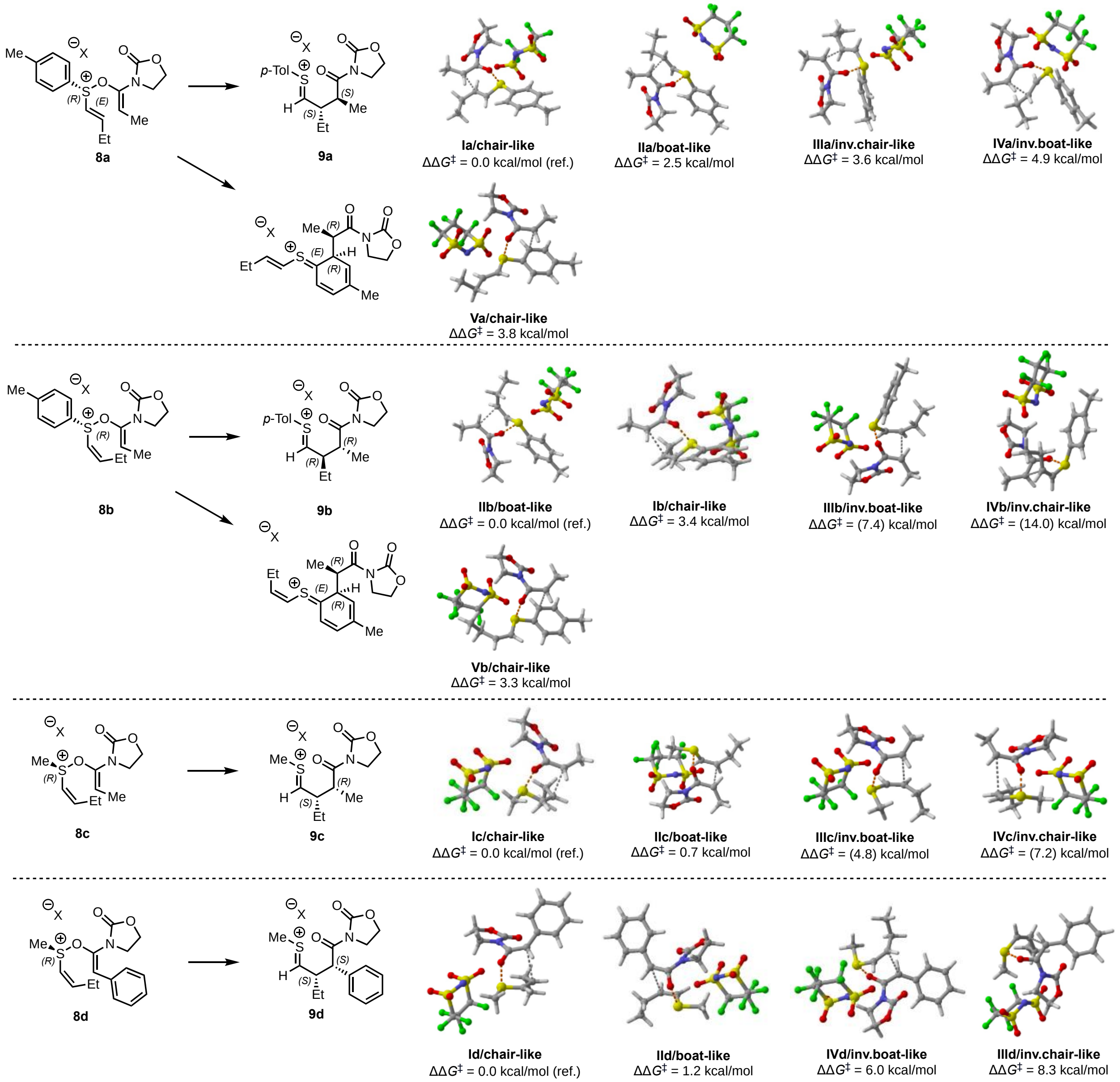

Figure S1 The systems investigated in silico and the relative Gibbs free energies ($\Delta\Delta G^{\ddagger}_{273}$) for the most stabilized conformations of the respective transition states. The $\Delta\Delta G^{\ddagger}_{273}$ values in parentheses are computed at the B3LYP-D3BJ-SMD(DCM)/def2-TZVP//B3LYP-D3BJ-SMD(DCM)/def2-SVP level of theory and without quasi-harmonic correction.

3.3 Analysis of the stereoselectivity of sulfoxide addition – *E*- vs. *Z*-enolonium ion formation

The protonation of an ynamide can theoretically lead to two different enolonium isomers, with either *E*- or *Z*-configuration. The complexity of the system studied herein initially forced us to adopt simplifying approximations, one of which is the assumption of predominant formation of the *E*-configured enolonium intermediate, with the corresponding *Z*-configured counterpart being considered negligible.

We followed up on this assumption with *in silico* analysis of the addition step,[24] which suggests a considerable probability that the sulfoxide reagent (**2h**) undergoes protonation (**2h**→**2h-H**, Scheme S1), prior to reaction with the ynamide (**1b**). Figure S2 depicts the relaxed scan of the protonation event, computed to be barrierless.

The rapid protonation of the sulfoxide, with **2h-H** acting as the proton source for ynamide activation, suggests a high probability of stereocontrol in the sulfoxide-addition step (**1b-H/2h**, Scheme S1). As protonation concomitantly generates the electrophilic keteniminium ion and a suitable nucleophile (the regenerated sulfoxide), *syn*-addition—leading to the *E*-configured enolonium isomer **8c**—becomes the dominant pathway.

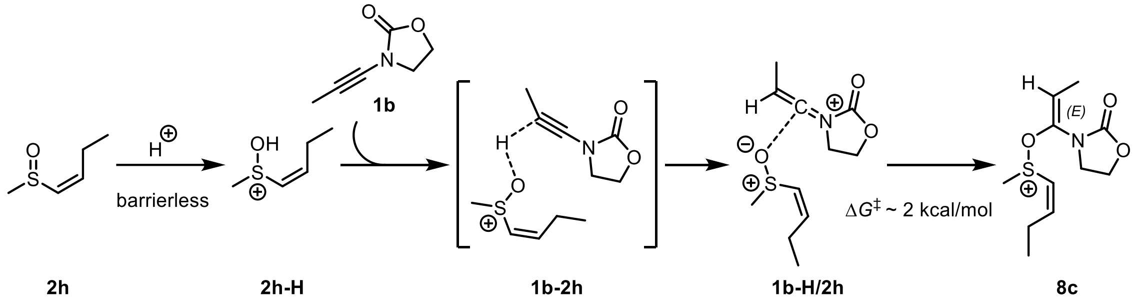

Scheme S1 The computationally suggested formation of the E-configured enolonium species via a protonated sulfoxide.

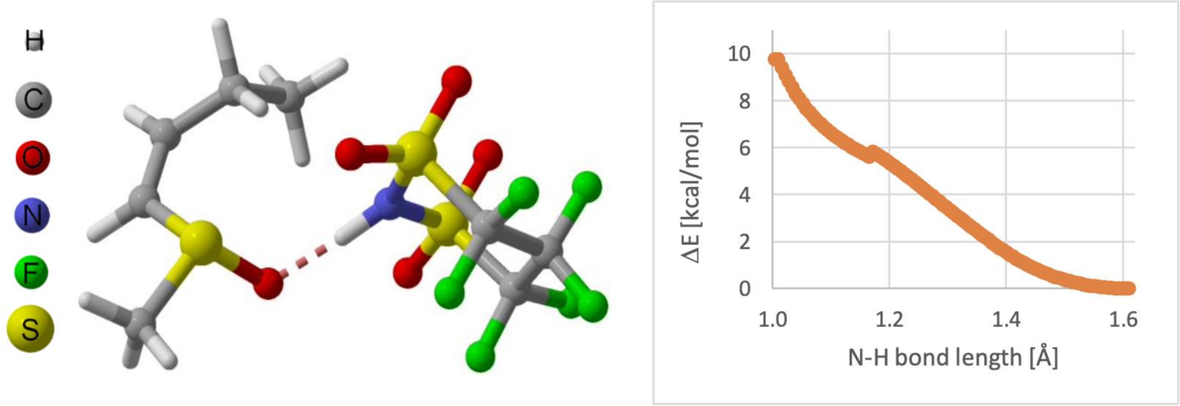

Figure S2 Relaxed energy scan of the sulfoxide protonation by the applied acid showing a barrierless event.

We would also like to refer to the work of Cao and coworkers,[25] who have experimentally shown that the reaction of ynamides and diphenylsulfonimides preferentially yields ketene aminals with high levels of stereoselectivity with regard to double-bond configuration (*E*/*Z* , 90:10 or higher). Although our sulfoxide-ynamide adducts cannot be isolated as intermediates, this report provides strong support to the assumption of *E*-configuration for the intermediates invoked in our transformation.

Furthermore, we believe that *E*- vs. *Z*-selectivity for the general case of ynamide protonation should be studied in more detail, both experimentally and computationally, and this is one of the foci of our ongoing research.

3.4 XYZ coordinates for the most stabilized conformations

8a

8	-3.391173000	-0.336310000	-0.326786000
6	-2.007577000	0.127541000	1.890658000
6	-1.086267000	-0.695919000	2.398710000
6	-0.965562000	-1.024392000	3.835604000
6	-3.444184000	-1.722884000	-0.204074000
16	-1.962243000	0.348765000	0.185630000
6	-4.447367000	-2.260114000	0.478816000
6	-2.054608000	-2.172595000	-2.243468000
6	-1.638277000	-3.358027000	-0.263765000
6	-1.144608000	-3.372060000	-2.492989000
1	-1.524580000	-1.224802000	-2.364510000
1	-2.932808000	-2.174920000	-2.889161000
1	-1.642729000	-4.152692000	-3.069974000
1	-0.206247000	-3.096846000	-2.969041000
7	-2.427650000	-2.397622000	-0.857690000
8	-0.847507000	-3.903282000	-1.193191000
8	-1.628526000	-3.685295000	0.892376000
1	-5.079483000	-1.570056000	1.027954000
6	-4.769434000	-3.706398000	0.530120000
1	-5.843967000	-3.856328000	0.394625000
1	-4.237745000	-4.274100000	-0.234314000
1	-4.505175000	-4.127263000	1.505439000
1	-1.733746000	-0.503415000	4.411734000
1	-1.148063000	-2.101557000	3.936538000
1	-2.773459000	0.636045000	2.464508000
1	-0.352860000	-1.138963000	1.729035000
6	0.430088000	-0.707448000	4.364095000
1	0.648316000	0.359592000	4.283572000
1	0.506841000	-0.994959000	5.414478000
1	1.193627000	-1.252511000	3.804581000
6	-2.385878000	2.009209000	-0.115002000
6	-3.403134000	2.666361000	0.573941000
6	-1.646297000	2.649434000	-1.101220000
6	-3.662280000	3.984354000	0.265727000
1	-3.989902000	2.157188000	1.328516000
6	-1.920795000	3.975063000	-1.389645000
1	-0.863360000	2.121739000	-1.633624000
6	-2.923850000	4.661432000	-0.711999000
1	-4.453199000	4.507317000	0.792341000
1	-1.341842000	4.483341000	-2.152094000
6	-3.208963000	6.096591000	-1.006304000
1	-2.649288000	6.445052000	-1.874565000
1	-4.275134000	6.252616000	-1.189481000
1	-2.935695000	6.721464000	-0.150475000
6	3.367067000	-1.060965000	-0.726209000
6	3.730269000	-0.292982000	0.569262000
6	3.507722000	1.238493000	0.502091000
7	1.626888000	0.853495000	-1.349061000
16	1.802134000	1.715649000	-0.036283000
16	1.646074000	-0.725149000	-1.311819000
9	3.745170000	1.751747000	1.716387000
9	4.388212000	1.761833000	-0.359981000
9	5.032056000	-0.511877000	0.829537000
9	3.013365000	-0.790250000	1.584091000
9	4.226579000	-0.708537000	-1.690516000
9	3.505283000	-2.371947000	-0.491526000
8	1.589050000	-1.210568000	-2.658096000
8	0.797034000	-1.341335000	-0.332395000
8	0.945057000	1.353556000	1.057414000
8	1.888317000	3.095460000	-0.406485000

9a

8	1.607382000	-3.095423000	0.244449000
6	2.315444000	0.380943000	-0.866772000
6	3.127301000	-0.747404000	-1.348134000
6	4.290931000	-0.364999000	-2.260783000
6	2.314814000	-2.175138000	0.584953000
16	2.498156000	1.911959000	-1.403519000
6	3.524121000	-1.735844000	-0.218542000
6	1.005738000	-2.041170000	2.669625000
6	2.561877000	-0.348761000	2.253616000
6	1.228899000	-1.180629000	3.903778000
1	1.144378000	-3.103005000	2.864405000
1	0.027700000	-1.879201000	2.221465000
1	0.309086000	-0.760846000	4.303017000
1	1.775729000	-1.704272000	4.688611000
7	2.049358000	-1.543610000	1.787483000
8	2.052643000	-0.087650000	3.450849000
8	3.322398000	0.398492000	1.687529000
1	4.216068000	-1.219125000	0.443443000
6	4.190372000	-2.975997000	-0.795922000
1	4.396795000	-3.692362000	0.001409000
1	3.552896000	-3.462799000	-1.535010000
1	5.140275000	-2.718001000	-1.263629000
1	4.582011000	-1.252984000	-2.823850000
1	3.922169000	0.340857000	-3.012433000
1	1.470012000	0.197081000	-0.207086000
1	2.373609000	-1.284729000	-1.955477000
6	5.504268000	0.211543000	-1.549703000
1	5.256769000	1.077626000	-0.931342000
1	5.980135000	-0.527903000	-0.901902000
1	6.246262000	0.532933000	-2.283435000
6	1.226134000	2.818914000	-0.598723000
6	1.089394000	2.796241000	0.784515000
6	0.382805000	3.580775000	-1.396798000
6	0.062938000	3.517204000	1.362002000
1	1.777177000	2.226169000	1.396513000
6	-0.639293000	4.293792000	-0.795125000
1	0.501805000	3.596817000	-2.474167000
6	-0.822993000	4.264930000	0.585470000
1	-0.059029000	3.493463000	2.439218000
1	-1.317108000	4.871895000	-1.413121000
6	-1.964241000	4.985177000	1.222918000
1	-1.672813000	5.425246000	2.178791000
1	-2.353607000	5.771503000	0.574888000
1	-2.778754000	4.281038000	1.421736000
6	-2.970936000	-1.199082000	0.847937000
6	-3.408005000	-1.530619000	-0.601338000
6	-2.256963000	-1.559756000	-1.638735000
7	-0.873304000	0.120731000	-0.123679000
16	-1.262582000	-0.002221000	-1.651230000
16	-1.975324000	0.357975000	0.987682000
9	-1.446881000	-2.587199000	-1.355598000
9	-2.787624000	-1.758725000	-2.852381000
9	-4.324092000	-0.638405000	-0.992514000
9	-3.985041000	-2.746569000	-0.590488000
9	-4.070508000	-1.086493000	1.604187000
9	-2.241838000	-2.222612000	1.314903000
8	-2.905660000	1.416478000	0.736063000
8	-1.337892000	0.287084000	2.269461000
8	-0.067047000	-0.309561000	-2.384650000
8	-2.141788000	1.001412000	-2.169204000

Ia

8	2.508937000	-0.315203000	1.375975000

6	3.093880000	-0.420524000	-1.314507000
6	2.806162000	-1.734969000	-1.529934000
6	3.682084000	-2.673703000	-2.266792000
6	2.453510000	-1.605581000	1.353373000
16	1.960066000	0.447884000	-0.437988000
6	3.571080000	-2.325121000	0.997096000
6	0.315565000	-1.540869000	2.613106000
6	0.618668000	-3.213077000	1.000226000
6	-0.837200000	-2.538818000	2.625476000
1	0.004148000	-0.564214000	2.249377000
1	0.783712000	-1.435493000	3.592210000
1	-0.853393000	-3.147034000	3.530959000
1	-1.805715000	-2.063264000	2.483063000
7	1.227330000	-2.180638000	1.678053000
8	-0.600459000	-3.411428000	1.510060000
8	1.065040000	-3.859913000	0.089661000
1	4.421720000	-1.709839000	0.731349000
6	3.796532000	-3.766910000	1.189939000
1	3.014542000	-4.239665000	1.781590000
1	3.844733000	-4.290835000	0.227950000
1	4.766746000	-3.917646000	1.672870000
1	3.639776000	-3.644018000	-1.760205000
1	3.178982000	-2.854934000	-3.227819000
1	4.047948000	0.034099000	-1.551384000
1	1.808170000	-2.099720000	-1.301476000
6	5.111753000	-2.228964000	-2.486120000
1	5.665031000	-2.998077000	-3.027796000
1	5.163424000	-1.309078000	-3.073519000
1	5.624355000	-2.054589000	-1.536195000
6	2.590630000	2.042179000	-0.199957000
6	1.638127000	3.057686000	-0.153770000
6	3.944369000	2.313748000	-0.004032000
6	2.054757000	4.359086000	0.061910000
1	0.585718000	2.835626000	-0.293856000
6	4.335015000	3.617740000	0.208953000
1	4.677249000	1.516616000	-0.000332000
6	3.401994000	4.660880000	0.242058000
1	1.318021000	5.153767000	0.092066000
1	5.385524000	3.838820000	0.364382000
6	3.854059000	6.063983000	0.473905000
1	3.011156000	6.754553000	0.505232000
1	4.403539000	6.142396000	1.416199000
1	4.534557000	6.384912000	-0.320275000
6	-3.209386000	-0.745794000	-1.325487000
6	-4.171451000	0.351664000	-0.803662000
6	-3.894097000	0.831704000	0.643371000
7	-1.351378000	0.133253000	0.345825000
16	-2.145526000	1.371617000	0.917140000
16	-1.426975000	-0.277183000	-1.176864000
9	-4.723715000	1.847371000	0.915797000
9	-4.166401000	-0.174381000	1.486082000
9	-5.422426000	-0.143903000	-0.841031000
9	-4.115848000	1.404648000	-1.626963000
9	-3.417902000	-1.868469000	-0.625285000
9	-3.502760000	-0.986206000	-2.610052000
8	-0.688715000	-1.493528000	-1.350057000
8	-1.229846000	0.781283000	-2.123574000
8	-2.003201000	2.596400000	0.184684000
8	-1.970192000	1.400470000	2.338753000

IIa

8	2.687924000	-0.434826000	1.911207000
6	1.493697000	-1.018355000	-0.605367000
6	1.211860000	-2.312071000	-0.309910000
6	1.430766000	-3.422875000	-1.262881000
6	3.510219000	-1.289526000	1.400338000
16	1.230738000	0.144429000	0.580147000
6	3.281307000	-2.635903000	1.527480000
6	5.218615000	0.497298000	1.117253000
6	5.027287000	-1.137839000	-0.534028000
6	6.399265000	0.542156000	0.161586000
1	5.526187000	0.475337000	2.162019000
1	4.528791000	1.329843000	0.963814000
1	6.586846000	1.533669000	-0.245055000
1	7.311003000	0.146436000	0.613273000
7	4.613920000	-0.764871000	0.724580000
8	6.022904000	-0.324786000	-0.920837000
8	4.591948000	-2.017678000	-1.227240000
1	2.362775000	-2.885557000	2.045876000
6	4.227895000	-3.721002000	1.229158000
1	5.236386000	-3.358489000	1.034482000
1	4.245114000	-4.430693000	2.061456000
1	3.903515000	-4.286002000	0.346664000
1	2.037760000	-3.079224000	-2.101810000
1	1.992226000	-4.212072000	-0.751565000
1	1.983914000	-0.739116000	-1.528547000
1	0.670373000	-2.541454000	0.604993000
6	0.103539000	-4.004733000	-1.747875000
1	-0.480228000	-3.257071000	-2.286957000
1	0.297107000	-4.844785000	-2.418124000
1	-0.501987000	-4.363599000	-0.913315000
6	1.811299000	1.646689000	-0.062098000
6	2.729620000	1.770763000	-1.105161000
6	1.319636000	2.777056000	0.590935000
6	3.129423000	3.030251000	-1.496537000
1	3.139389000	0.902996000	-1.604917000
6	1.739730000	4.029592000	0.186067000
1	0.602340000	2.672399000	1.397213000
6	2.643632000	4.179298000	-0.863608000
1	3.841651000	3.129790000	-2.308462000
1	1.352880000	4.908286000	0.689565000
6	3.073988000	5.532078000	-1.321571000
1	2.864853000	6.293387000	-0.569359000
1	4.140346000	5.548326000	-1.557083000
1	2.537680000	5.807834000	-2.235741000
6	-4.567734000	-1.052218000	-0.273364000
6	-4.805385000	0.472431000	-0.405865000
6	-4.065615000	1.339206000	0.643040000
7	-2.222082000	-0.565774000	0.879934000
16	-2.247513000	1.004656000	0.719202000
16	-2.776815000	-1.517874000	-0.254089000
9	-4.266432000	2.629223000	0.341067000
9	-4.597036000	1.099443000	1.848956000
9	-6.125945000	0.699431000	-0.273373000
9	-4.437296000	0.865035000	-1.630663000
9	-5.135481000	-1.476015000	0.863849000
9	-5.173434000	-1.662768000	-1.301162000
8	-2.738700000	-2.864613000	0.232020000
8	-2.306331000	-1.251368000	-1.581774000
8	-1.716624000	1.517535000	-0.510840000
8	-1.801370000	1.593481000	1.947741000

IIIa

8	3.328833000	1.305873000	-1.919700000
6	1.022979000	1.911657000	-0.538480000
6	1.535673000	2.069032000	0.710880000
6	1.138821000	3.211756000	1.567464000
6	3.964787000	1.513138000	-0.807401000
16	1.428139000	0.721690000	-1.644720000
6	3.919428000	2.744234000	-0.202543000
6	5.351654000	-0.527823000	-1.145723000

6	4.599790000	-0.034745000	1.003364000
6	6.052068000	-1.390210000	-0.107065000
1	6.050122000	-0.029990000	-1.817604000
1	4.629664000	-1.096853000	-1.733741000
1	6.000764000	-2.454526000	-0.326914000
1	7.090346000	-1.091464000	0.047232000
7	4.682080000	0.431938000	-0.285372000
8	5.332193000	-1.149416000	1.113129000
8	3.970582000	0.429531000	1.918885000
1	3.321017000	3.481968000	-0.722425000
6	4.770682000	3.192693000	0.914620000
1	4.190185000	3.295690000	1.838052000
1	5.587475000	2.500540000	1.117968000
1	5.177192000	4.182229000	0.688834000
1	2.017763000	3.586002000	2.100152000
1	0.733669000	4.023105000	0.958912000
1	0.440689000	2.699182000	-1.006483000
1	2.110389000	1.286654000	1.191059000
6	0.100662000	2.745108000	2.590570000
1	0.491972000	1.933942000	3.208802000
1	-0.168719000	3.575335000	3.246680000
1	-0.803091000	2.388170000	2.092932000
6	1.676941000	-0.813974000	-0.857668000
6	1.311867000	-1.108762000	0.453861000
6	2.174169000	-1.811026000	-1.697766000
6	1.501750000	-2.390305000	0.927315000
1	0.849227000	-0.368407000	1.088493000
6	2.359721000	-3.086252000	-1.199701000
1	2.412983000	-1.589849000	-2.732615000
6	2.038941000	-3.395316000	0.121029000
1	1.209155000	-2.620779000	1.945707000
1	2.751236000	-3.859289000	-1.851375000
6	2.240886000	-4.769965000	0.664639000
1	2.950945000	-4.752496000	1.496876000
1	1.301990000	-5.172541000	1.054433000
1	2.619814000	-5.450351000	-0.098462000
6	-4.025039000	-0.375343000	1.307834000
6	-4.828774000	-0.139772000	0.004857000
6	-4.160318000	0.822595000	-1.008208000
7	-1.787135000	0.182518000	-0.004722000
16	-2.426546000	0.342516000	-1.438923000
16	-2.284704000	-0.926244000	1.005807000
9	-4.906384000	0.846878000	-2.120874000
9	-4.146238000	2.055477000	-0.482196000
9	-6.023891000	0.380319000	0.343138000
9	-5.042261000	-1.317583000	-0.592487000
9	-3.999402000	0.768055000	2.007079000
9	-4.665094000	-1.300573000	2.035707000
8	-1.604407000	-0.736037000	2.252798000
8	-2.394464000	-2.254928000	0.480733000
8	-2.544110000	-0.855915000	-2.215472000
8	-1.854922000	1.502348000	-2.059758000

IVa

8	2.172180000	0.935800000	-1.630374000
6	1.554462000	0.331150000	1.058030000
6	2.761639000	0.663433000	1.582151000
6	2.913329000	1.417456000	2.844874000
6	2.309745000	2.075869000	-1.027868000
16	1.354749000	-0.460988000	-0.410278000
6	3.507584000	2.397828000	-0.444817000
6	0.199036000	2.869019000	-2.076418000
6	0.733775000	3.611982000	0.073069000
6	-0.697852000	4.023467000	-1.651592000
1	-0.331571000	1.917406000	-2.082199000
1	0.660084000	3.036754000	-3.049475000
1	-0.471281000	4.944008000	-2.192813000
1	-1.758049000	3.795759000	-1.739027000
7	1.195396000	2.904440000	-1.017803000
8	-0.398322000	4.237920000	-0.264627000
8	1.223424000	3.678906000	1.168075000
1	4.247042000	1.606921000	-0.489520000
6	3.939393000	3.728250000	0.012143000
1	3.964741000	3.770322000	1.107828000
1	4.957884000	3.923370000	-0.334810000
1	3.276698000	4.523103000	-0.328068000
1	3.618012000	2.239022000	2.677573000
1	1.959952000	1.855140000	3.141473000
1	0.622496000	0.722785000	1.446352000
1	3.662996000	0.228782000	1.162444000
6	3.466755000	0.507173000	3.941420000
1	4.427248000	0.073932000	3.651217000
1	3.615306000	1.080757000	4.858433000
1	2.774549000	-0.309673000	4.157378000
6	2.712573000	-1.565187000	-0.571251000
6	3.130493000	-2.322642000	0.519118000
6	3.240811000	-1.789358000	-1.838298000
6	4.113516000	-3.278325000	0.336209000
1	2.684309000	-2.178556000	1.494921000
6	4.224426000	-2.748137000	-1.996420000
1	2.898623000	-1.213018000	-2.688264000
6	4.682034000	-3.502939000	-0.916361000
1	4.438431000	-3.869471000	1.185548000
1	4.644552000	-2.916606000	-2.982059000
6	5.763441000	-4.517464000	-1.095423000
1	5.670716000	-5.327777000	-0.370379000
1	5.747942000	-4.940183000	-2.101405000
1	6.745871000	-4.056199000	-0.948713000
6	-3.172144000	-2.203838000	0.155710000
6	-3.019876000	-1.315246000	-1.104137000
6	-3.468877000	0.155714000	-0.921383000
7	-2.963312000	-0.036498000	1.683945000
16	-2.676812000	0.992467000	0.525365000
16	-2.363177000	-1.497749000	1.663384000
9	-3.166791000	0.829720000	-2.041655000
9	-4.797110000	0.181502000	-0.758323000
9	-3.762605000	-1.857286000	-2.086912000
9	-1.739674000	-1.331916000	-1.495241000
9	-4.477526000	-2.366694000	0.406525000
9	-2.638726000	-3.403971000	-0.111462000
8	-2.904861000	-2.229461000	2.767117000
8	-0.953297000	-1.589618000	1.408790000
8	-3.447259000	2.175490000	0.753807000
8	-1.296990000	1.139305000	0.149224000

Va

8	2.131529000	0.716034000	-1.807999000
6	1.822464000	3.059066000	-0.149452000
6	0.745839000	3.850270000	-0.090490000
6	0.740998000	5.279087000	-0.479136000
6	2.500578000	-0.502430000	-1.811008000
16	1.608813000	1.394059000	0.216369000
6	3.836791000	-0.802077000	-1.571260000
6	0.315767000	-1.128930000	-2.792900000
6	1.390591000	-2.673749000	-1.418145000
6	-0.335335000	-2.498976000	-2.893563000
1	0.548438000	-0.704687000	-3.769436000
1	-0.291306000	-0.428661000	-2.221540000
1	-1.407321000	-2.475430000	-2.710180000
1	-0.132149000	-2.989272000	-3.847758000
7	1.534022000	-1.468594000	-2.073371000
8	0.281181000	-3.276567000	-1.854778000

8	2.111358000	-3.150552000	-0.581431000
1	4.435450000	0.069241000	-1.332191000
6	4.541696000	-2.062143000	-1.821303000
1	3.925855000	-2.814630000	-2.307457000
1	5.429884000	-1.853375000	-2.427821000
1	4.914797000	-2.480275000	-0.877811000
1	0.451291000	5.866030000	0.401024000
1	1.745748000	5.594109000	-0.770867000
1	2.794458000	3.392874000	-0.493506000
1	-0.201659000	3.433898000	0.247751000
6	-0.259521000	5.545630000	-1.600717000
1	0.010035000	4.993614000	-2.504014000
1	-1.267939000	5.243820000	-1.308069000
1	-0.279239000	6.610395000	-1.841899000
6	3.074536000	0.819028000	0.860619000
6	4.209137000	1.602387000	1.129575000
6	3.119883000	-0.573764000	1.053583000
6	5.331798000	0.994971000	1.628768000
1	4.191488000	2.673845000	0.978493000
6	4.269789000	-1.157625000	1.578886000
1	2.234602000	-1.178282000	0.896407000
6	5.384247000	-0.393953000	1.868933000
1	6.202086000	1.600412000	1.859298000
1	4.281496000	-2.226271000	1.757609000
6	6.624416000	-1.003304000	2.429525000
1	6.875476000	-0.549846000	3.392756000
1	6.511544000	-2.078320000	2.570616000
1	7.474634000	-0.826694000	1.763862000
6	-2.855157000	-1.371785000	1.572537000
6	-3.859390000	-1.238719000	0.400596000
6	-4.217070000	0.217413000	0.011463000
7	-1.863016000	1.018969000	0.957091000
16	-2.735966000	1.271618000	-0.334070000
16	-1.295728000	-0.412333000	1.311188000
9	-4.999076000	0.181491000	-1.076150000
9	-4.911750000	0.769839000	1.014400000
9	-4.996735000	-1.863897000	0.756938000
9	-3.360380000	-1.864163000	-0.672528000
9	-3.444908000	-0.936954000	2.693631000
9	-2.556195000	-2.668186000	1.725570000
8	-0.659886000	-0.344392000	2.592192000
8	-0.614870000	-1.097351000	0.249490000
8	-2.195353000	0.767236000	-1.563838000
8	-3.210156000	2.622571000	-0.302752000

Ib

8	-2.971400000	0.958990000	0.586700000
6	-2.620610000	0.183490000	-1.992200000
6	-2.071250000	1.266420000	-2.604530000
6	-0.686510000	1.770450000	-2.466590000
16	-1.922010000	-0.402230000	-0.582070000
6	-3.485790000	2.779150000	-0.722340000
6	-2.728150000	2.183640000	0.267760000
6	-1.394060000	2.324610000	2.337300000
6	-0.817100000	3.748520000	0.575930000
6	-0.374660000	3.363920000	2.782050000
1	-2.278220000	2.308530000	2.974740000
1	-0.966670000	1.324640000	2.293870000
1	0.516500000	2.920280000	3.221580000
1	-0.799460000	4.097720000	3.469150000
7	-1.728240000	2.810670000	1.007590000
8	0.010580000	4.049190000	1.582590000
8	-0.724640000	4.251690000	-0.511670000
1	-4.230610000	2.117960000	-1.143860000
1	-0.176630000	1.330770000	-1.607460000
1	-0.715430000	2.850020000	-2.309950000
1	-3.591270000	-0.200110000	-2.280610000
1	-2.653930000	1.686540000	-3.422140000
6	0.093470000	1.466540000	-3.746160000
1	1.109630000	1.854620000	-3.652100000
1	-0.373810000	1.937640000	-4.614990000
1	0.156170000	0.391940000	-3.923740000
6	3.942640000	-0.172840000	-0.284800000
6	3.536410000	-1.572640000	-0.809330000
6	3.392160000	0.179350000	1.119630000
7	1.345840000	-1.472500000	0.699710000
16	1.708700000	-1.859620000	-0.790090000
16	1.557310000	-0.009540000	1.252930000
8	1.535080000	-3.272540000	-0.942550000
8	1.183610000	-0.991380000	-1.803490000
8	1.015330000	1.050830000	0.451260000
8	1.268180000	-0.010420000	2.656400000
9	3.726530000	1.445790000	1.399090000
9	3.972940000	-0.621910000	2.021890000
9	3.531240000	0.753530000	-1.158070000
9	5.286320000	-0.126040000	-0.225670000
9	3.989400000	-1.698950000	-2.063750000
9	4.126690000	-2.504120000	-0.049520000
6	-3.568990000	4.212800000	-1.057990000
1	-4.603570000	4.472260000	-1.294270000
1	-2.963200000	4.452480000	-1.938490000
1	-3.207530000	4.846420000	-0.248200000
6	-2.859470000	-1.769180000	-0.079620000
6	-2.152790000	-2.735550000	0.633740000
6	-4.230550000	-1.900050000	-0.300000000
6	-2.821040000	-3.854850000	1.096130000
1	-1.090910000	-2.614480000	0.818350000
6	-4.875030000	-3.022830000	0.171690000
1	-4.790880000	-1.128840000	-0.813280000
6	-4.184700000	-4.020630000	0.869720000
1	-2.271760000	-4.612950000	1.642780000
1	-5.941690000	-3.130680000	0.006540000
6	-4.906580000	-5.232290000	1.356720000
1	-4.263810000	-5.857800000	1.976440000
1	-5.257190000	-5.833240000	0.511920000
1	-5.789230000	-4.952740000	1.937960000

IIb

8	-3.242510000	0.948860000	1.748850000
6	-1.543680000	1.300780000	-0.469410000
6	-1.506150000	2.654690000	-0.500260000
6	-0.949770000	3.552340000	0.540790000
16	-1.326940000	0.503460000	0.994460000
6	-4.147680000	2.688530000	0.526620000
6	-4.033690000	1.349700000	0.817340000
6	-5.075900000	-0.908300000	0.744840000
6	-4.827550000	0.269480000	-1.249240000
6	-5.874110000	-1.553570000	-0.374640000
1	-4.178690000	-1.479120000	0.994420000
1	-5.666130000	-0.755000000	1.647440000
1	-6.946830000	-1.378270000	-0.271760000
1	-5.680480000	-2.618510000	-0.483880000
7	-4.733460000	0.359770000	0.121840000
8	-5.423950000	-0.889470000	-1.566860000
8	-4.437550000	1.053340000	-2.073010000
1	-3.500840000	3.324410000	1.118250000
1	-0.943450000	3.079210000	1.525510000
1	-1.566720000	4.452000000	0.609010000
1	-1.842550000	0.735310000	-1.339590000
1	-1.749580000	3.111750000	-1.456330000
6	0.475380000	3.951000000	0.147280000
1	0.880920000	4.641190000	0.889620000

1	0.490660000	4.446130000	-0.826390000
1	1.123740000	3.074570000	0.097310000
6	4.183470000	0.725090000	0.788650000
6	4.594810000	0.079120000	-0.558170000
6	3.499230000	0.096510000	-1.653240000
7	1.650600000	0.165600000	0.244520000
16	2.617190000	0.030010000	1.486270000
16	1.892900000	-0.633720000	-1.096340000
9	5.181720000	0.542880000	1.663760000
9	4.023150000	2.042460000	0.599640000
9	5.661930000	0.751720000	-1.029710000
9	4.966320000	-1.186830000	-0.336960000
9	3.287240000	1.366760000	-2.025440000
9	3.954160000	-0.583820000	-2.714260000
8	2.193160000	0.962510000	2.488110000
8	2.942870000	-1.306460000	1.888470000
8	2.141240000	-2.037850000	-0.957550000
8	0.923490000	-0.200460000	-2.058590000
6	-5.180640000	3.333080000	-0.301930000
1	-4.740940000	3.755830000	-1.211690000
1	-5.615390000	4.171680000	0.251220000
1	-5.970660000	2.646810000	-0.602070000
6	-1.381490000	-1.195990000	0.657620000
6	-1.824270000	-1.776690000	-0.530060000
6	-0.925950000	-1.993080000	1.708770000
6	-1.789990000	-3.149060000	-0.656560000
1	-2.188150000	-1.179870000	-1.354910000
6	-0.904250000	-3.365790000	1.562210000
1	-0.574810000	-1.537130000	2.628190000
6	-1.326760000	-3.966950000	0.378030000
1	-2.126690000	-3.600910000	-1.583110000
1	-0.540010000	-3.981830000	2.376460000
6	-1.257370000	-5.446140000	0.200470000
1	-1.251840000	-5.964170000	1.160360000
1	-0.335770000	-5.714820000	-0.327020000
1	-2.092390000	-5.813760000	-0.399170000

IIIb

8	1.877350000	-1.246340000	1.428560000
6	1.224630000	-0.472140000	-1.218550000
6	2.222690000	-0.974640000	-1.990670000
6	3.707570000	-0.868220000	-1.921660000
16	1.302960000	0.249010000	0.346650000
6	2.756520000	-3.107280000	0.342650000
6	1.690650000	-2.440850000	0.870080000
6	-0.490350000	-2.818160000	2.055550000
6	-0.264660000	-3.467710000	-0.210990000
6	-1.817920000	-3.358060000	1.492070000
1	-0.565410000	-1.777590000	2.391580000
1	-0.105660000	-3.433720000	2.883020000
1	-2.171960000	-4.250490000	2.026310000
1	-2.610680000	-2.599980000	1.484470000
7	0.386130000	-2.933200000	0.893040000
8	-1.540080000	-3.730850000	0.129510000
8	0.198620000	-3.683350000	-1.301590000
1	3.678440000	-2.526580000	0.274960000
1	4.055020000	-0.489770000	-0.954120000
1	4.112780000	-1.885920000	-2.047670000
1	0.185330000	-0.667160000	-1.490440000
1	1.847730000	-1.490780000	-2.883880000
6	4.242940000	0.006690000	-3.067960000
1	3.872860000	1.039210000	-2.988970000
1	5.342810000	0.032320000	-3.034360000
1	3.939190000	-0.391690000	-4.048930000
6	-2.334520000	2.159850000	0.844490000
6	-3.235630000	2.166990000	-0.417800000
6	-2.785840000	1.207840000	-1.549150000
7	-1.601790000	-0.390230000	0.284660000
16	-2.110590000	0.443150000	1.579300000
16	-2.588050000	-0.580000000	-0.988860000
9	-2.880960000	2.963000000	1.768540000
9	-1.120180000	2.634800000	0.514110000
9	-3.242590000	3.423570000	-0.915140000
9	-4.488970000	1.844770000	-0.053970000
9	-1.594200000	1.624480000	-2.018280000
9	-3.686980000	1.259320000	-2.540530000
8	-0.983610000	0.566100000	2.508810000
8	-3.429320000	0.068400000	2.095200000
8	-3.940390000	-1.051910000	-0.692140000
8	-1.815120000	-1.217810000	-2.058720000
6	2.799410000	-4.540150000	-0.038590000
1	3.726650000	-5.003500000	0.336810000
1	2.812140000	-4.640700000	-1.138200000
1	1.932040000	-5.099920000	0.335660000
6	2.843400000	1.156620000	0.421450000
6	3.741570000	0.970260000	1.477680000
6	3.038930000	2.166710000	-0.526210000
6	4.873880000	1.779590000	1.544350000
1	3.571140000	0.191820000	2.219880000
6	4.178520000	2.966460000	-0.435860000
1	2.321030000	2.324260000	-1.333100000
6	5.116690000	2.785140000	0.591830000
1	5.587890000	1.626210000	2.357730000
1	4.339860000	3.746400000	-1.183750000
6	6.347080000	3.644690000	0.689300000
1	7.260250000	3.027070000	0.685390000
1	6.351480000	4.215860000	1.632670000
1	6.409530000	4.359000000	-0.144040000

IVb

8	3.617080000	-0.146370000	1.844160000
6	4.479650000	-1.424830000	-0.567110000
6	3.875590000	-1.045670000	-1.727850000
6	2.449130000	-1.030400000	-2.147370000
16	3.801650000	-1.874620000	0.950980000
6	4.989390000	1.424870000	0.797730000
6	3.742410000	0.952300000	1.102140000
6	1.515420000	1.994300000	1.674710000
6	2.338290000	2.143270000	-0.529260000
6	0.832010000	3.122600000	0.890690000
1	1.951090000	2.344380000	2.620360000
1	0.830540000	1.160700000	1.872850000
1	-0.260940000	3.087320000	0.932390000
1	1.183530000	4.117140000	1.206500000
7	2.567510000	1.609850000	0.729320000
8	1.241180000	2.918530000	-0.477480000
8	2.995260000	1.968460000	-1.527250000
1	5.817640000	0.758360000	1.052320000
1	2.120020000	-2.085260000	-2.189110000
1	1.807920000	-0.580700000	-1.373320000
1	5.570920000	-1.416620000	-0.500980000
1	4.599170000	-0.772030000	-2.506910000
6	2.225360000	-0.372780000	-3.505720000
1	2.828500000	-0.865310000	-4.286320000
1	1.166160000	-0.442540000	-3.784980000
1	2.494480000	0.691250000	-3.466630000
6	-4.093500000	0.237960000	-0.429060000
6	-2.999950000	0.035310000	-1.510220000
6	-3.651630000	-0.043810000	1.031280000
7	-1.091910000	0.551710000	0.331020000
16	-1.429400000	1.019120000	-1.181740000
16	-2.104700000	0.904040000	1.535860000

8	-0.420870000	0.435210000	-2.065570000
8	-1.828540000	2.417860000	-1.349110000
8	-2.545920000	2.301260000	1.623020000
8	-1.611960000	0.242190000	2.749410000
9	-4.655800000	0.292280000	1.854570000
9	-3.406860000	-1.361150000	1.162770000
9	-4.536250000	1.505410000	-0.501960000
9	-5.121670000	-0.592410000	-0.716300000
9	-3.506550000	0.402060000	-2.698130000
9	-2.675630000	-1.270540000	-1.563220000
6	5.311020000	2.747880000	0.211980000
1	5.420950000	2.665710000	-0.884350000
1	6.266570000	3.119700000	0.612440000
1	4.520430000	3.488730000	0.401480000
6	2.122960000	-2.420920000	0.689790000
6	1.075130000	-1.850610000	1.412910000
6	1.926280000	-3.548090000	-0.118700000
6	-0.200930000	-2.395870000	1.285870000
1	1.247070000	-0.987890000	2.053950000
6	0.639900000	-4.071650000	-0.232400000
1	2.756380000	-4.008480000	-0.658090000
6	-0.443680000	-3.504750000	0.460140000
1	-1.018840000	-1.928650000	1.832300000
1	0.476670000	-4.942100000	-0.872430000
6	-1.835530000	-4.054000000	0.310880000
1	-2.454240000	-3.369330000	-0.290580000
1	-2.330210000	-4.154700000	1.289510000
1	-1.832640000	-5.035760000	-0.184220000

Vb

8	-2.329371000	0.290708000	-2.085198000
6	-1.671853000	-2.526503000	-2.131504000
6	-0.566013000	-2.879914000	-2.800031000
6	0.818656000	-2.397650000	-2.588953000
6	-2.705754000	1.253060000	-1.340439000
16	-1.558103000	-1.359657000	-0.874899000
6	-4.011146000	1.278533000	-0.870061000
6	-0.645996000	2.497928000	-1.937640000
6	-1.535997000	2.792684000	0.198275000
6	-0.001272000	3.678476000	-1.229798000
1	-0.999231000	2.753620000	-2.936203000
1	0.017709000	1.635823000	-1.996245000
1	1.085782000	3.641870000	-1.224944000
1	-0.335111000	4.635667000	-1.635183000
7	-1.763209000	2.236551000	-1.042932000
8	-0.461128000	3.585456000	0.127803000
8	-2.159404000	2.618767000	1.211660000
1	-4.589968000	0.408171000	-1.157511000
6	-4.726653000	2.403386000	-0.259669000
1	-5.651341000	2.578705000	-0.821023000
1	-5.040379000	2.145527000	0.759273000
1	-4.140386000	3.317772000	-0.216468000
1	0.885286000	-1.723072000	-1.732393000
1	1.445595000	-3.268065000	-2.361608000
1	-2.648755000	-2.903272000	-2.402158000
1	-0.707846000	-3.586051000	-3.615953000
6	1.355689000	-1.710249000	-3.841256000
1	1.320100000	-2.380622000	-4.703811000
1	0.769341000	-0.819231000	-4.076177000
1	2.390515000	-1.402956000	-3.686284000
6	-2.991055000	-1.451077000	0.042094000
6	-4.052758000	-2.349748000	-0.155971000
6	-3.097606000	-0.448613000	1.024915000
6	-5.157818000	-2.262090000	0.649928000
1	-4.000854000	-3.124582000	-0.908113000
6	-4.227147000	-0.398369000	1.838657000

1	-2.260473000	0.206453000	1.228763000
6	-5.267044000	-1.290583000	1.666609000
1	-5.968885000	-2.968993000	0.510061000
1	-4.279152000	0.354525000	2.616399000
6	-6.482583000	-1.251115000	2.529098000
1	-6.422096000	-0.449270000	3.265103000
1	-7.380671000	-1.100653000	1.922598000
1	-6.611632000	-2.200043000	3.057746000
6	2.477272000	-1.070623000	1.799218000
6	3.886610000	-0.771905000	1.231151000
6	3.909832000	-0.342964000	-0.257251000
7	1.413798000	0.557120000	-0.003940000
16	2.769248000	1.065167000	-0.638939000
16	1.280901000	0.317833000	1.553044000
9	5.163595000	0.004653000	-0.576269000
9	3.561238000	-1.396899000	-1.008559000
9	4.627283000	-1.890448000	1.350381000
9	4.458790000	0.185904000	1.968254000
9	1.996156000	-2.167590000	1.195385000
9	2.596963000	-1.328752000	3.108270000
8	-0.009891000	-0.248898000	1.812993000
8	1.749043000	1.373847000	2.398541000
8	3.364843000	2.210499000	-0.016763000
8	2.622805000	1.062332000	-2.064842000

Ic

8	-2.322797000	-0.410833000	-1.933846000
6	-2.138652000	-2.714608000	-0.548078000
6	-2.885890000	-2.487913000	0.571653000
6	-2.492120000	-1.661949000	1.738141000
6	-0.311845000	-2.194860000	-2.496265000
16	-0.963523000	-1.600789000	-0.961158000
6	-4.361751000	-0.802332000	-0.942935000
6	-3.251553000	-0.000406000	-1.137769000
6	-2.170623000	2.212205000	-1.224224000
6	-3.397924000	1.677495000	0.694194000
6	-2.355680000	3.431183000	-0.330862000
1	-2.502095000	2.392497000	-2.246999000
1	-1.141181000	1.858021000	-1.241302000
1	-1.414498000	3.905749000	-0.059617000
1	-3.027593000	4.170910000	-0.769240000
7	-3.041011000	1.249047000	-0.566296000
8	-2.966921000	2.931112000	0.866365000
8	-3.992679000	1.071479000	1.543985000
1	-4.341313000	-1.706043000	-1.535976000
1	0.241414000	-1.365757000	-2.938038000
1	0.371251000	-3.018335000	-2.281759000
1	-1.127468000	-2.516563000	-3.142007000
1	-1.770618000	-0.888272000	1.467863000
1	-3.372915000	-1.152529000	2.129120000
1	-2.429403000	-3.448423000	-1.291875000
1	-3.742193000	-3.143891000	0.712274000
6	-1.898601000	-2.566543000	2.819981000
1	-1.641903000	-1.966039000	3.694978000
1	-2.610363000	-3.335419000	3.130942000
1	-0.992587000	-3.062533000	2.465038000
6	3.407415000	-0.360900000	0.917548000
6	2.033364000	-0.109725000	1.587923000
6	3.350913000	-0.613909000	-0.609530000
7	1.105414000	0.785525000	-0.743517000
16	1.051688000	1.231005000	0.772509000
16	2.466762000	0.715460000	-1.543344000
8	-0.292542000	1.053252000	1.238714000
8	1.734078000	2.448188000	1.092873000
8	3.310507000	1.868879000	-1.457888000
8	2.194562000	0.154800000	-2.834604000

9	4.604662000	-0.722535000	-1.065575000
9	2.719761000	-1.779063000	-0.826017000
9	4.202612000	0.687758000	1.153584000
9	3.957750000	-1.444848000	1.495974000
9	2.241038000	0.213004000	2.870248000
9	1.324988000	-1.247607000	1.548773000
6	-5.638390000	-0.433414000	-0.299626000
1	-6.466828000	-0.886038000	-0.848746000
1	-5.688426000	-0.798380000	0.731189000
1	-5.775876000	0.646878000	-0.253862000

IIc

8	3.672212000	-0.503855000	1.585622000
6	1.854903000	1.340761000	0.549933000
6	2.645860000	2.038305000	-0.309111000
6	3.758340000	2.955933000	0.045647000
6	1.058712000	0.073642000	2.812445000
16	2.393986000	1.000622000	2.099516000
6	4.461323000	-0.074425000	-0.537412000
6	3.636225000	-0.766625000	0.315789000
6	2.335174000	-2.853389000	0.728094000
6	1.978819000	-1.677743000	-1.263336000
6	1.579171000	-3.697358000	-0.286831000
1	1.692707000	-2.531409000	1.547890000
1	3.207051000	-3.367290000	1.131744000
1	2.203338000	-4.483186000	-0.716731000
1	0.662547000	-4.124287000	0.112005000
7	2.733681000	-1.731713000	-0.106141000
8	1.228087000	-2.783639000	-1.336653000
8	1.956037000	-0.805839000	-2.086353000
1	5.029464000	0.709902000	-0.052362000
1	1.486263000	-0.532093000	3.612242000
1	0.354693000	0.792079000	3.238854000
1	0.571490000	-0.547706000	2.063693000
1	4.206664000	2.702822000	1.009829000
1	4.541628000	2.883178000	-0.712372000
1	0.961785000	0.817119000	0.223508000
1	2.292885000	2.074545000	-1.336552000
6	3.228737000	4.391254000	0.077442000
1	2.472315000	4.510809000	0.855805000
1	4.049611000	5.081827000	0.281104000
1	2.779767000	4.666041000	-0.879745000
6	-3.016015000	-0.568221000	1.068118000
6	-3.734706000	0.505185000	0.213912000
6	-2.794298000	1.526918000	-0.471763000
7	-0.853939000	-0.278981000	-0.438334000
16	-1.699014000	-1.478312000	0.141621000
16	-1.458740000	0.741720000	-1.484894000
9	-3.932228000	-1.436601000	1.514909000
9	-2.453560000	0.033910000	2.127380000
9	-4.568635000	1.186953000	1.022105000
9	-4.468771000	-0.106085000	-0.722057000
9	-2.220591000	2.280652000	0.478683000
9	-3.535570000	2.324358000	-1.251726000
8	-0.912300000	-2.125297000	1.153782000
8	-2.393602000	-2.290276000	-0.810046000
8	-2.122923000	0.164196000	-2.613366000
8	-0.497812000	1.781972000	-1.701032000
6	4.827040000	-0.422732000	-1.920126000
1	4.464809000	-1.403252000	-2.222090000
1	4.428941000	0.313706000	-2.626051000
1	5.917022000	-0.388250000	-2.015394000

IIIc

8	2.563481000	0.048719000	-1.769864000
6	1.783610000	1.363062000	0.605913000
6	2.921221000	1.777299000	1.218168000
6	4.070771000	2.598228000	0.740694000
6	2.149274000	2.721102000	-1.916285000
16	1.332569000	1.360768000	-1.055578000
6	4.347038000	-0.596857000	-0.420334000
6	3.074448000	-0.790427000	-0.873059000
6	1.393990000	-2.586971000	-1.378750000
6	2.033914000	-2.180961000	0.865013000
6	0.596326000	-3.468889000	-0.399664000
1	0.755033000	-1.912681000	-1.960577000
1	2.006918000	-3.182067000	-2.072798000
1	0.769823000	-4.541599000	-0.562611000
1	-0.481059000	-3.266013000	-0.430171000
7	2.250805000	-1.839017000	-0.463678000
8	1.080504000	-3.130282000	0.913023000
8	2.586993000	-1.734461000	1.836807000
1	4.809279000	0.344866000	-0.721889000
1	1.937804000	3.651411000	-1.370424000
1	1.674935000	2.752162000	-2.906954000
1	3.224449000	2.542150000	-2.016459000
1	4.218552000	2.520759000	-0.341720000
1	4.982259000	2.211939000	1.223656000
1	1.043201000	0.799088000	1.179727000
1	2.937273000	1.546644000	2.290859000
6	3.880748000	4.072277000	1.140904000
1	2.999084000	4.504575000	0.643030000
1	4.766464000	4.657226000	0.849261000
1	3.743248000	4.177921000	2.228368000
6	-2.849463000	0.786265000	-1.123578000
6	-3.528140000	0.752327000	0.270102000
6	-2.551742000	0.678299000	1.471224000
7	-0.722060000	-0.550781000	-0.101520000
16	-1.676875000	-0.655740000	-1.406361000
16	-1.348333000	-0.768253000	1.378457000
9	-3.801527000	0.768019000	-2.067717000
9	-2.142959000	1.924931000	-1.236083000
9	-4.265147000	1.877854000	0.400272000
9	-4.354218000	-0.306961000	0.321320000
9	-1.828890000	1.813148000	1.509082000
9	-3.267544000	0.578611000	2.601002000
8	-0.882883000	-0.279204000	-2.580357000
8	-2.533153000	-1.843152000	-1.472115000
8	-2.165983000	-1.967145000	1.562539000
8	-0.304148000	-0.442037000	2.353930000
6	5.174344000	-1.577558000	0.325798000
1	6.199609000	-1.593485000	-0.078576000
1	5.248549000	-1.284941000	1.387662000
1	4.751633000	-2.590868000	0.292264000

IVc

8	3.467195000	0.019933000	1.858329000
6	3.417080000	2.531990000	0.571559000
6	3.070107000	2.465378000	-0.740566000
6	1.863348000	1.926945000	-1.416893000
6	0.946838000	1.513174000	1.679039000
16	2.697312000	1.817628000	1.959360000
6	4.849068000	-0.283542000	0.017024000
6	3.660594000	-0.523009000	0.662905000
6	1.877634000	-2.221515000	1.111056000
6	2.292421000	-1.569604000	-1.123059000
6	1.163427000	-3.147431000	0.113108000
1	2.541582000	-2.772662000	1.792070000
1	1.171014000	-1.627146000	1.702854000
1	0.082826000	-3.211941000	0.284811000
1	1.593208000	-4.160218000	0.099190000
7	2.656667000	-1.376712000	0.205363000

8	1.376981000	-2.553157000	-1.179583000
8	2.708970000	-0.986944000	-2.091187000
1	5.457057000	0.499301000	0.475514000
1	0.489679000	2.464753000	1.370873000
1	0.553448000	1.197236000	2.652362000
1	0.759685000	0.731802000	0.936047000
1	1.315427000	1.203796000	-0.810606000
1	2.199050000	1.377778000	-2.307457000
1	4.355340000	3.017338000	0.857359000
1	3.775585000	2.984222000	-1.402882000
6	0.922581000	3.064906000	-1.849882000
1	0.530349000	3.612588000	-0.978485000
1	0.075227000	2.633191000	-2.399019000
1	1.435370000	3.788336000	-2.503640000
6	-3.664219000	0.479365000	-0.059572000
6	-2.701955000	0.731307000	-1.248511000
6	-2.970067000	0.293822000	1.314203000
7	-0.743469000	-0.600807000	0.054934000
16	-1.374132000	-0.592674000	-1.444081000
16	-1.666179000	-1.059757000	1.303959000
8	-0.391588000	-0.010580000	-2.355825000
8	-2.099125000	-1.803082000	-1.830427000
8	-2.406924000	-2.311695000	1.130852000
8	-0.897886000	-0.854568000	2.537768000
9	-3.900695000	0.003980000	2.234338000
9	-2.363905000	1.447675000	1.654268000
9	-4.399776000	-0.614727000	-0.321213000
9	-4.495714000	1.541072000	0.036576000
9	-3.423135000	0.796964000	-2.377609000
9	-2.087194000	1.915026000	-1.054772000
6	5.432554000	-1.039520000	-1.118029000
1	5.253953000	-0.516955000	-2.073789000
1	6.523138000	-1.120354000	-0.985797000
1	4.999969000	-2.044356000	-1.217269000

Id

8	-1.659360000	-0.332986000	-1.879633000
6	-1.394106000	-2.663156000	-0.419490000
6	-2.001937000	-2.556011000	0.786027000
6	-1.568462000	-1.719940000	1.928174000
6	0.364961000	-2.138955000	-2.435795000
16	-0.219125000	-1.538288000	-0.874263000
6	-3.753296000	-0.671276000	-1.020598000
6	-2.585751000	0.086952000	-1.106235000
6	-1.397341000	2.250754000	-0.958541000
6	-2.690964000	1.561821000	0.868644000
6	-1.518557000	3.356356000	0.082627000
1	-1.698636000	2.582135000	-1.952350000
1	-0.393816000	1.832060000	-1.010220000
1	-0.554412000	3.728571000	0.422485000
1	-2.132909000	4.188560000	-0.264188000
7	-2.344049000	1.276289000	-0.437207000
8	-2.184705000	2.751723000	1.201908000
8	-3.344001000	0.889247000	1.618718000
1	-3.649714000	-1.644884000	-1.482998000
1	0.940751000	-1.327888000	-2.882496000
1	1.015115000	-2.997024000	-2.257382000
1	-0.479676000	-2.412783000	-3.066467000
1	-0.881151000	-0.930408000	1.617101000
1	-2.445154000	-1.231016000	2.356839000
1	-1.724506000	-3.375059000	-1.168297000
1	-2.819550000	-3.248662000	0.974553000
6	-0.902623000	-2.595479000	2.991478000
1	-0.003372000	-3.075046000	2.598544000
1	-0.617448000	-1.979283000	3.846517000
1	-1.580458000	-3.376602000	3.344734000

6	4.303142000	-0.387211000	0.748092000
6	2.975294000	-0.181963000	1.519536000
6	4.145949000	-0.574114000	-0.781324000
7	1.888457000	0.812667000	-0.699778000
16	1.934328000	1.186909000	0.835056000
16	3.190350000	0.785005000	-1.594572000
8	0.624256000	0.979785000	1.380138000
8	2.631615000	2.392951000	1.166399000
8	4.032244000	1.940842000	-1.521682000
8	2.831026000	0.275384000	-2.885688000
9	5.367118000	-0.647703000	-1.324872000
9	3.512323000	-1.735779000	-1.006290000
9	5.103299000	0.659457000	0.976604000
9	4.900792000	-1.488427000	1.240556000
9	3.267444000	0.081966000	2.799050000
9	2.273447000	-1.323229000	1.474198000
6	-5.077751000	-0.319611000	-0.611089000
6	-5.517293000	0.996511000	-0.387783000
6	-6.011707000	-1.365151000	-0.497278000
6	-6.831891000	1.245112000	-0.050553000
1	-4.838032000	1.827695000	-0.518872000
6	-7.318356000	-1.113087000	-0.131864000
1	-5.684120000	-2.381601000	-0.687205000
6	-7.732454000	0.194649000	0.093809000
1	-7.161360000	2.265829000	0.104873000
1	-8.020615000	-1.932254000	-0.030240000
1	-8.760179000	0.397604000	0.372853000

IId

8	2.541050000	0.298050000	2.566445000
6	1.024475000	1.736308000	0.682118000
6	1.934061000	2.410892000	-0.056853000
6	2.895132000	3.438394000	0.405605000
6	-0.326380000	0.812815000	2.844839000
16	1.114725000	1.731144000	2.367300000
6	3.946437000	0.280489000	0.748670000
6	2.881106000	-0.254565000	1.441890000
6	1.554464000	-2.304928000	1.946999000
6	1.679303000	-1.556238000	-0.267645000
6	1.097063000	-3.378092000	0.971507000
1	0.720220000	-1.878196000	2.503567000
1	2.307270000	-2.668896000	2.645472000
1	1.831206000	-4.179481000	0.866265000
1	0.123031000	-3.792123000	1.217913000
7	2.130795000	-1.340969000	1.022754000
8	0.990243000	-2.703572000	-0.291604000
8	1.850889000	-0.863161000	-1.230276000
1	4.268387000	1.238407000	1.140924000
1	-0.147736000	0.439489000	3.853540000
1	-1.167062000	1.510549000	2.858822000
1	-0.515964000	-0.000715000	2.148482000
1	3.027278000	3.415252000	1.490296000
1	3.868872000	3.236263000	-0.050404000
1	0.302040000	1.067020000	0.229065000
1	1.864694000	2.264285000	-1.132070000
6	2.422046000	4.824041000	-0.036511000
1	1.466810000	5.076389000	0.428408000
1	3.158149000	5.576154000	0.253976000
1	2.296115000	4.868541000	-1.120667000
6	-3.883210000	-0.640335000	0.703662000
6	-4.483254000	0.212674000	-0.440946000
6	-3.500670000	1.224796000	-1.079513000
7	-1.472869000	-0.325107000	-0.359565000
16	-2.325870000	-1.517653000	0.225908000
16	-1.922514000	0.453913000	-1.661727000
9	-4.801037000	-1.535761000	1.089678000

9	-3.616900000	0.165449000	1.743010000
9	-5.523872000	0.904589000	0.060422000
9	-4.945793000	-0.604130000	-1.393548000
9	-3.206106000	2.165238000	-0.168923000
9	-4.116180000	1.815587000	-2.111879000
8	-1.731082000	-1.918341000	1.469773000
8	-2.727733000	-2.531792000	-0.700138000
8	-2.284595000	-0.355797000	-2.784661000
8	-1.022921000	1.551518000	-1.858334000
6	4.778249000	-0.264273000	-0.286140000
6	5.691728000	0.614357000	-0.891390000
6	4.778260000	-1.609214000	-0.688560000
6	6.542619000	0.179702000	-1.888955000
1	5.714576000	1.649619000	-0.567656000
6	5.646583000	-2.044076000	-1.669332000
1	4.120152000	-2.323215000	-0.212446000
6	6.520957000	-1.152361000	-2.282348000
1	7.230205000	0.875360000	-2.355688000
1	5.642166000	-3.086893000	-1.964487000
1	7.191029000	-1.500052000	-3.060401000

IIId

8	2.541050000	0.298050000	2.566445000
6	1.024475000	1.736308000	0.682118000
6	1.934061000	2.410892000	-0.056853000
6	2.895132000	3.438394000	0.405605000
6	-0.326380000	0.812815000	2.844839000
16	1.114725000	1.731144000	2.367300000
6	3.946437000	0.280489000	0.748670000
6	2.881106000	-0.254565000	1.441890000
6	1.554464000	-2.304928000	1.946999000
6	1.679303000	-1.556238000	-0.267645000
6	1.097063000	-3.378092000	0.971507000
1	0.720220000	-1.878196000	2.503567000
1	2.307270000	-2.668896000	2.645472000
1	1.831206000	-4.179481000	0.866265000
1	0.123031000	-3.792123000	1.217913000
7	2.130795000	-1.340969000	1.022754000
8	0.990243000	-2.703572000	-0.291604000
8	1.850889000	-0.863161000	-1.230276000
1	4.268387000	1.238407000	1.140924000
1	-0.147736000	0.439489000	3.853540000
1	-1.167062000	1.510549000	2.858822000
1	-0.515964000	-0.000715000	2.148482000
1	3.027278000	3.415252000	1.490296000
1	3.868872000	3.236263000	-0.050404000
1	0.302040000	1.067020000	0.229065000
1	1.864694000	2.264285000	-1.132070000
6	2.422046000	4.824041000	-0.036511000
1	1.466810000	5.076389000	0.428408000
1	3.158149000	5.576154000	0.253976000
1	2.296115000	4.868541000	-1.120667000
6	-3.883210000	-0.640335000	0.703662000
6	-4.483254000	0.212674000	-0.440946000
6	-3.500670000	1.224796000	-1.079513000
7	-1.472869000	-0.325107000	-0.359565000
16	-2.325870000	-1.517653000	0.225908000
16	-1.922514000	0.453913000	-1.661727000
9	-4.801037000	-1.535761000	1.089678000
9	-3.616900000	0.165449000	1.743010000
9	-5.523872000	0.904589000	0.060422000
9	-4.945793000	-0.604130000	-1.393548000
9	-3.206106000	2.165238000	-0.168923000
9	-4.116180000	1.815587000	-2.111879000
8	-1.731082000	-1.918341000	1.469773000
8	-2.727733000	-2.531792000	-0.700138000
8	-2.284595000	-0.355797000	-2.784661000
8	-1.022921000	1.551518000	-1.858334000
6	4.778249000	-0.264273000	-0.286140000
6	5.691728000	0.614357000	-0.891390000
6	4.778260000	-1.609214000	-0.688560000
6	6.542619000	0.179702000	-1.888955000
1	5.714576000	1.649619000	-0.567656000
6	5.646583000	-2.044076000	-1.669332000
1	4.120152000	-2.323215000	-0.212446000
6	6.520957000	-1.152361000	-2.282348000
1	7.230205000	0.875360000	-2.355688000
1	5.642166000	-3.086893000	-1.964487000
1	7.191029000	-1.500052000	-3.060401000

IVd

8	1.861142000	0.612083000	-2.089145000
6	0.840541000	1.603798000	0.326511000
6	1.848087000	2.275658000	0.927972000
6	2.656078000	3.435645000	0.488259000
6	0.716449000	3.105333000	-2.102152000
16	0.307315000	1.581216000	-1.277204000
6	3.742246000	0.474424000	-0.783819000
6	2.552244000	-0.062703000	-1.217457000
6	1.161808000	-2.074706000	-1.666453000
6	1.968258000	-1.703344000	0.507565000
6	0.882200000	-3.265836000	-0.757024000
1	0.254260000	-1.539838000	-1.940396000
1	1.697650000	-2.362098000	-2.571200000
1	1.484607000	-4.137258000	-1.017925000
1	-0.169819000	-3.542061000	-0.729345000
7	2.012123000	-1.266754000	-0.805167000
8	1.266221000	-2.839804000	0.558868000
8	2.464702000	-1.184197000	1.467316000
1	3.865744000	1.514450000	-1.062040000
1	0.431783000	3.941244000	-1.462679000
1	0.107912000	3.110803000	-3.007239000
1	1.770598000	3.140336000	-2.365456000
1	2.740690000	3.508056000	-0.593318000
1	3.664976000	3.313371000	0.891868000
1	0.329044000	0.825117000	0.885456000
1	2.003187000	1.982096000	1.964658000
6	2.053558000	4.724733000	1.054076000
1	1.054112000	4.897067000	0.649180000
1	2.686840000	5.574483000	0.791622000
1	1.975719000	4.679001000	2.142570000
6	-3.849804000	0.319319000	-0.937148000
6	-4.350547000	0.375050000	0.527308000
6	-3.229725000	0.417085000	1.594289000
7	-1.592768000	-0.815531000	-0.096682000
16	-2.651479000	-1.055372000	-1.247932000
16	-1.994851000	-0.951921000	1.427720000
9	-4.911121000	0.171837000	-1.740538000
9	-3.256862000	1.485218000	-1.231465000
9	-5.098619000	1.485886000	0.666554000
9	-5.131372000	-0.685254000	0.760009000
9	-2.582635000	1.587563000	1.486895000
9	-3.797137000	0.358156000	2.805536000
8	-2.028004000	-0.743095000	-2.500384000
8	-3.424410000	-2.257007000	-1.146756000
8	-2.692097000	-2.147351000	1.792672000
8	-0.874291000	-0.536603000	2.219610000
6	4.893372000	-0.144285000	-0.187860000
6	5.924089000	0.706693000	0.242617000
6	5.074764000	-1.530073000	-0.058037000
6	7.073205000	0.198580000	0.817529000
1	5.803672000	1.778096000	0.122679000

6	6.234224000	-2.034136000	0.496070000
1	4.318497000	-2.214673000	-0.417458000
6	7.231166000	-1.175201000	0.947482000
1	7.851603000	0.871795000	1.157570000
1	6.364416000	-3.106736000	0.582115000
1	8.133576000	-1.579098000	1.392194000

4 X-ray structural analysis data

General information:

The X-ray intensity data were measured on STOE STADIVARI diffractometer equipped with multilayer monochromator, Mo K/α Primux 100 micro, micro focus sealed tube and Oxford cooling system. The structures were solved by *Intrinsic Phasing, Charge Flipping or Direct Methods*. Non-hydrogen atoms were refined with *anisotropic displacement parameters*. Hydrogen atoms were inserted at calculated positions and refined with riding model. The following software was used: X-Area Recipe[26], *X-Area Pilatus3_SV*[27], *OLEX2*[28] for structure solution, refinement, molecular diagrams and graphical user-interface, *Shelxl*[29] for refinement and graphical user-interface *SHELXT-2015*[30] for structure solution, *SHELXL-2015*[30] for refinement, *Platon*[31] for symmetry check.

Compound 18c:

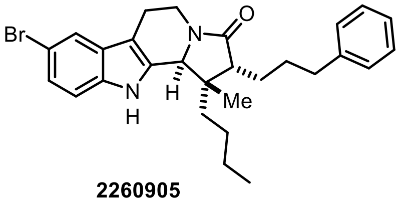

2260905

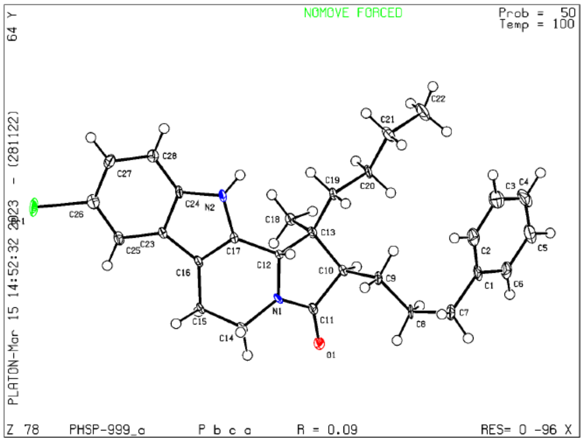

Bond precision: C-C = 0.0086 A Wavelength=1.54186

Cell:	a=18.5666(4)	b=8.1284(2)	c=32.1891(8)
	alpha=90	beta=90	gamma=90
Temperature:	100 K		

	Calculated	Reported
Volume	4857.9(2)	4857.9(2)
Space group	P b c a	P b c a
Hall group	-P 2ac 2ab	-P 2ac 2ab
Moiety formula	C28 H33 Br N2 O	?
Sum formula	C28 H33 Br N2 O	C28 H33 Br N2 O
Mr	493.46	493.48
Dx,g cm-3	1.349	1.349
Z	8	8
Mu (mm-1)	2.468	2.468
F000	2064.0	2064.0
F000'	2063.24	
h,k,lmax	22,9,39	9,22,39
Nref	4552	4501
Tmin,Tmax	0.762,0.821	0.178,0.744
Tmin'	0.691	

Correction method= # Reported T Limits: Tmin=0.178 Tmax=0.744
AbsCorr = MULTI-SCAN

Data completeness= 0.989 Theta(max)= 69.520

R(reflections)= 0.0912(3643) wR2(reflections)= 0.2817(4501)

S = 1.129 Npar= 292

(+)-nephrosteranic acid

Crystal Data for $C_{17}H_{30}O_4$ (M =298.41 g/mol): monoclinic, space group $P2_1$ (no. 4), a = 5.3984(3) Å, b = 29.119(3) Å, c = 5.4950(4) Å, β = 92.369(5)°, V = 863.05(11) Å^3, Z = 2, T = 100 K, μ(Mo Kα) = 0.080 mm^{-1}, Dcalc = 1.148 g/cm^3, 27322 reflections measured (7.422° ≤ 2Θ ≤ 66.018°), 5941 unique (R_{int} = 0.0390, R_{sigma} = 0.0461) which were used in all calculations. The final R_1 was 0.0529 (I > 2σ(I)) and wR_2 was 0.1425 (all data).

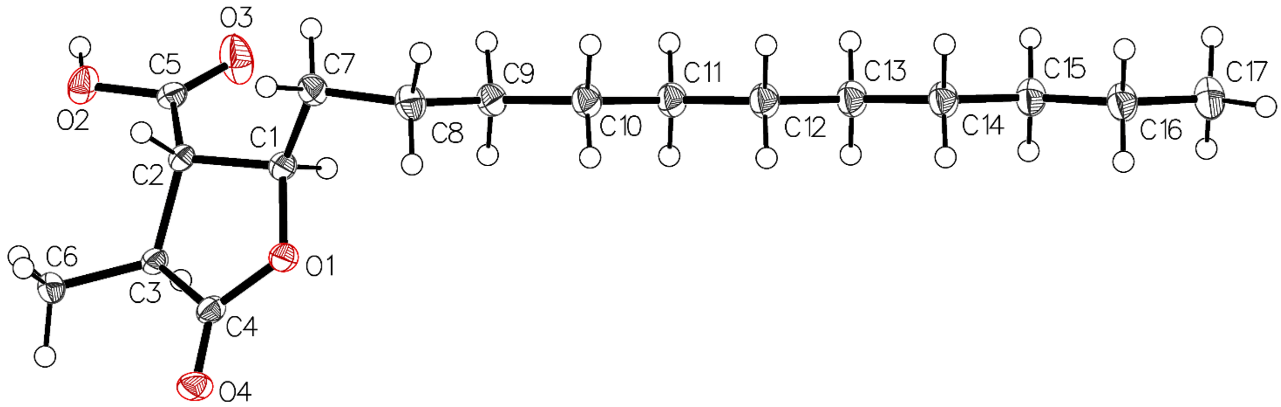

Figure S3 Asymmetric Unit drawn with 50% displacement ellipsoid. The bond precision for C-C single bonds is 0.0029Å. The chirality could not be proven by crystallographic methods. The packing has the same order as in rocellaric acid (see below) and is not visualized. The Hydrogen Bond O2H – O4 (2.616 **Å**, 160.854) is from the same class as in PHSP1087. The unit cell of both samples is almost the same. Most significant difference is the change in axis b, which is obviously related to the change in the length of the CH_2 chain (see packing in **Figure S**4).

Table S1 Sample and crystal data, data collection and structure refinement. More detailed information can be found in the Cif Code of CCDC: 2290758

Identification code	(+)-nephrosteranic acid
Empirical formula	$C_{17}H_{30}O_4$
Formula weight	298.41
Temperature/K	100
Crystal system	monoclinic
Space group	$P2_1$
a/Å	5.3984(3)
b/Å	29.119(3)

c/Å	5.4950(4)
α/°	90
β/°	92.369(5)
γ/°	90
Volume/Å^3	863.05(11)
Z	2
ρ_{calc}g/cm^3	1.148
μ/mm^{-1}	0.080
F(000)	328.0
Crystal size/mm^3	0.28 × 0.183 × 0.1
Radiation	Mo Kα (λ = 0.71073)
2Θ range for data collection/°	7.422 to 66.018
Index ranges	-8 ≤ h ≤ 8, -44 ≤ k ≤ 44, -8 ≤ l ≤ 8
Reflections collected	27322
Independent reflections	5941 [R_{int} = 0.0390, R_{sigma} = 0.0461]
Data/restraints/parameters	5941/1/193
Goodness-of-fit on F^2	1.006
Final R indexes [I>=2σ (I)]	R_1 = 0.0529, wR_2 = 0.1330
Final R indexes [all data]	R_1 = 0.0694, wR_2 = 0.1425
Largest diff. peak/hole / e Å^{-3}	0.42/-0.54
Flack parameter	-1.5(10)

(+)-rocellaric acid

Crystal Data for $C_{19}H_{34}O_4$ (M =326.46 g/mol): monoclinic, space group $P2_1$ (no. 4), a = 5.3983(6) Å, b = 32.282(4) Å, c = 5.4869(6) Å, β = 92.836(9)°, V = 955.03(18) Å^3, Z = 2, T = 100 K, μ(Mo Kα) = 0.077 mm^{-1}, Dcalc = 1.135 g/cm^3, 20266 reflections measured (7.436° ≤ 2Θ ≤ 60.05°), 5012 unique (R_{int} = 0.0316, R_{sigma} = 0.0308) which were used in all calculations. The final R_1 was 0.0529 (I > 2σ(I)) and wR_2 was 0.1559 (all data).

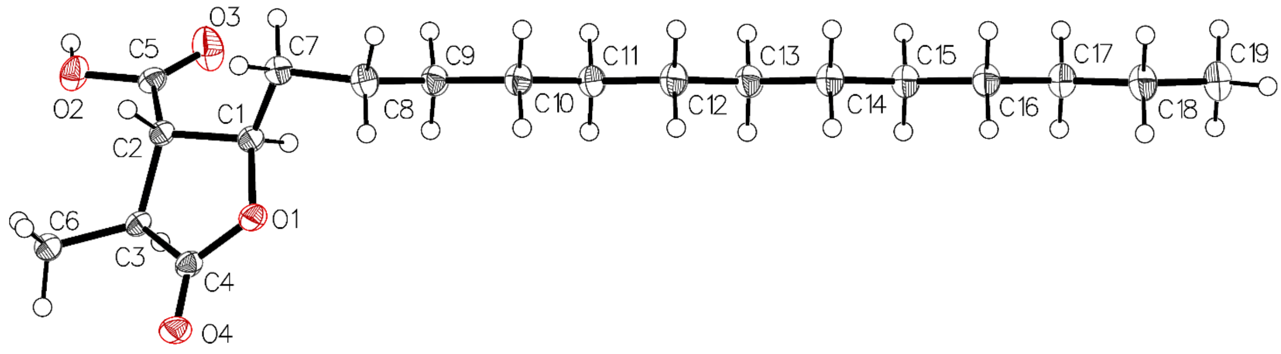

Figure S4 Asymmetric Unit drawn with 50% displacement ellipsoid. The bond precision for C-C single bonds is 0.0035Å. The chirality could not be proven by crystallographic methods.

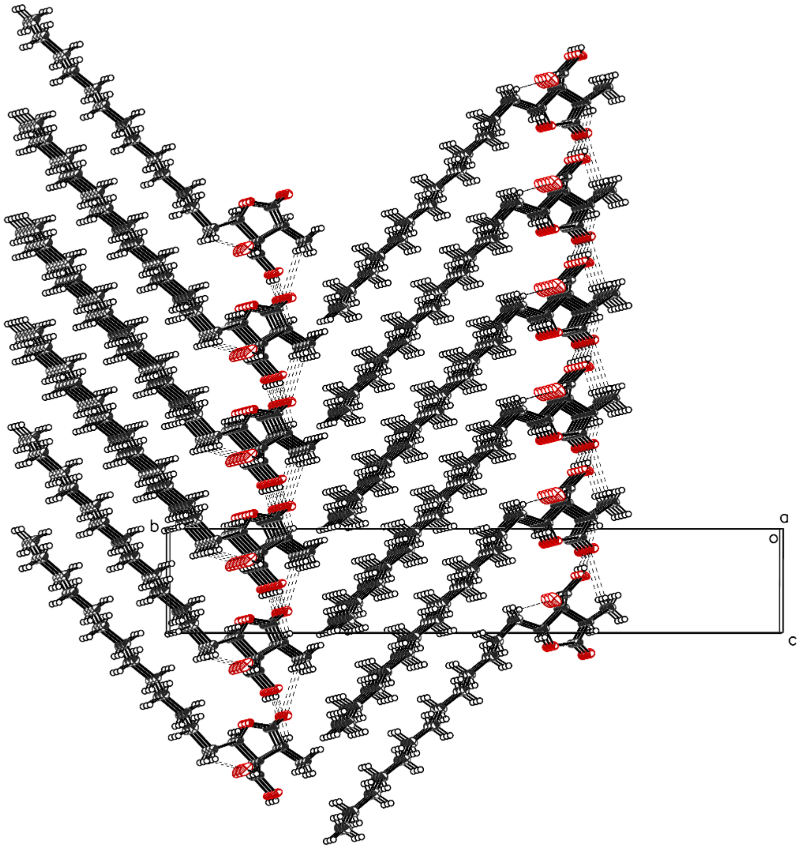

Figure S5 Packing view. The electrophilic part, which shows one linear "infinite" Hydrogen Bond system, is located in planes parallel to the a-c (1, 0, 1) plane. Responsible for this is the Hydrogen Bond O2H – O4 (2.622 **Å**, 162.540°), which can be registered as of moderate character and is in the range of 5 to 15 kcal/mol/bond. The second part is characterized by van der Waals interactions along the parallel CH_2-chain packing.

Table S2 Sample and crystal data, data collection and structure refinement. More detailed information can be found in the Cif Code of CCDC: 2290757

Identification code	(+)-rocellaric acid
Empirical formula	$C_{19}H_{34}O_4$
Formula weight	326.46
Temperature/K	100
Crystal system	monoclinic
Space group	$P2_1$
a/Å	5.3983(6)

b/Å	32.282(4)
c/Å	5.4869(6)
α/°	90
β/°	92.836(9)
γ/°	90
Volume/Å^3	955.03(18)
Z	2
ρ_{calc}g/cm^3	1.135
μ/mm^{-1}	0.077
F(000)	360.0
Crystal size/mm^3	0.32 × 0.217 × 0.06
Radiation	Mo Kα (λ = 0.71073)
2Θ range for data collection/°	7.436 to 60.05
Index ranges	-6 ≤ h ≤ 7, -45 ≤ k ≤ 45, -7 ≤ l ≤ 7
Reflections collected	20266
Independent reflections	5012 [R_{int} = 0.0316, R_{sigma} = 0.0308]
Data/restraints/parameters	5012/1/212
Goodness-of-fit on F^2	1.087
Final R indexes [I>=2σ (I)]	R_1 = 0.0529, wR_2 = 0.1455
Final R indexes [all data]	R_1 = 0.0611, wR_2 = 0.1559
Largest diff. peak/hole / e Å^{-3}	0.48/-0.30
Flack parameter	1.7(10)

5 NMR spectra

(*S*)-4-(*tert*-Butyl)-3-(5-phenylpent-1-yn-1-yl)oxazolidin-2-one (S1)

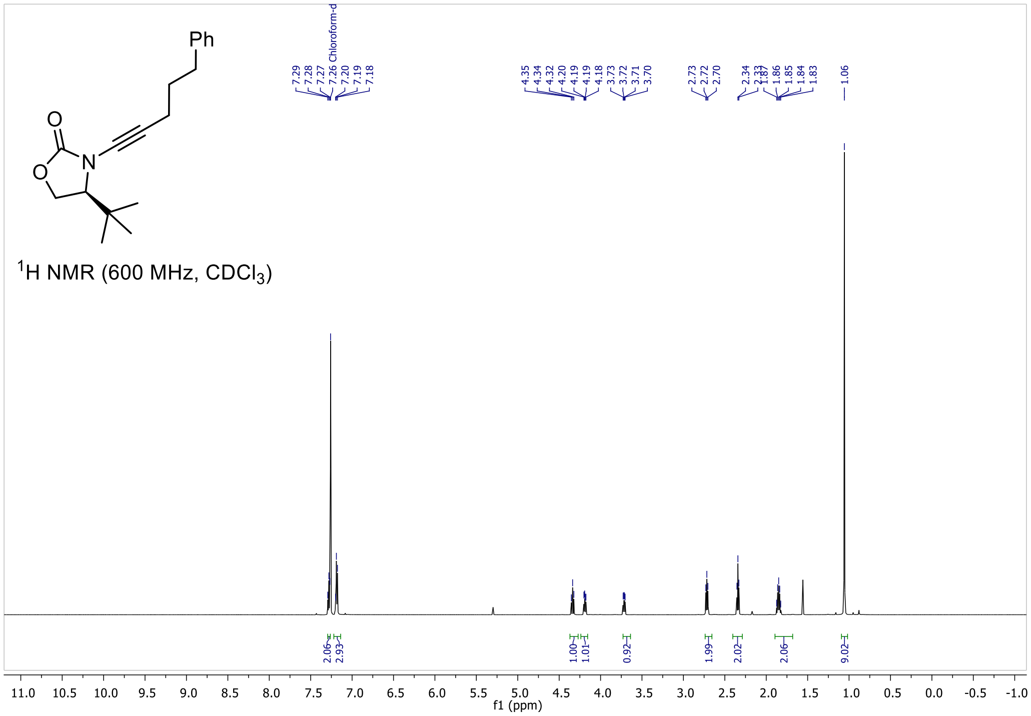

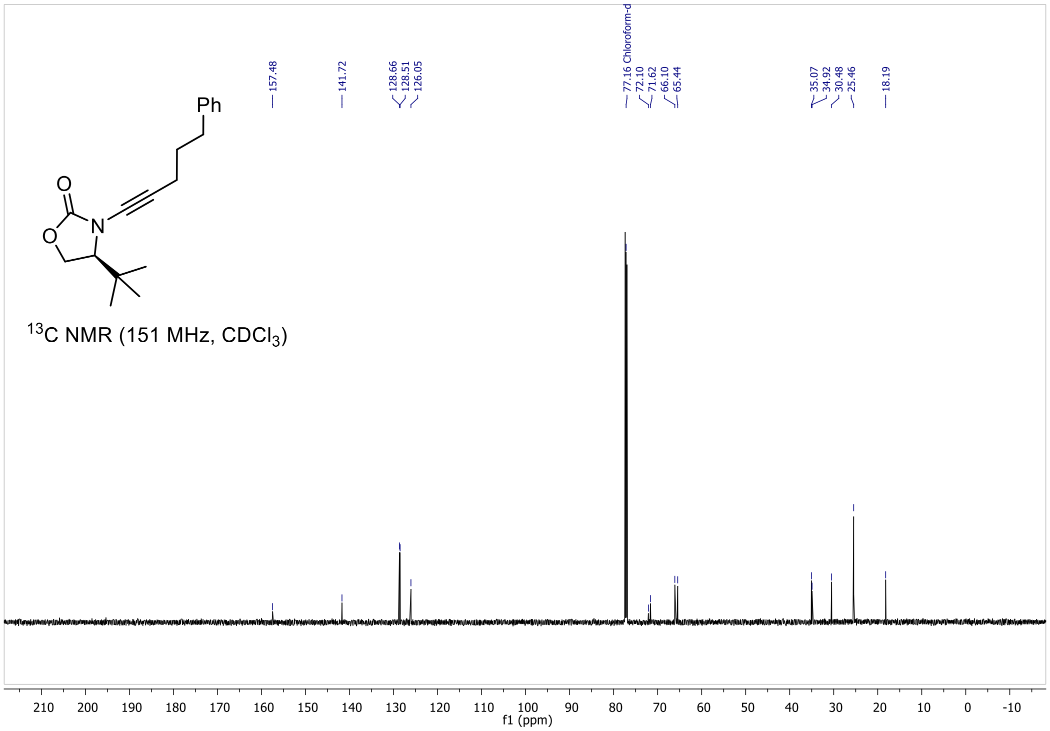

(±)-(3*S*,4*S*,5*R*)-4-Isopropyl-5-methyl-3-(3-phenylpropyl)dihydrofuran-2(3*H*)-one (11a)

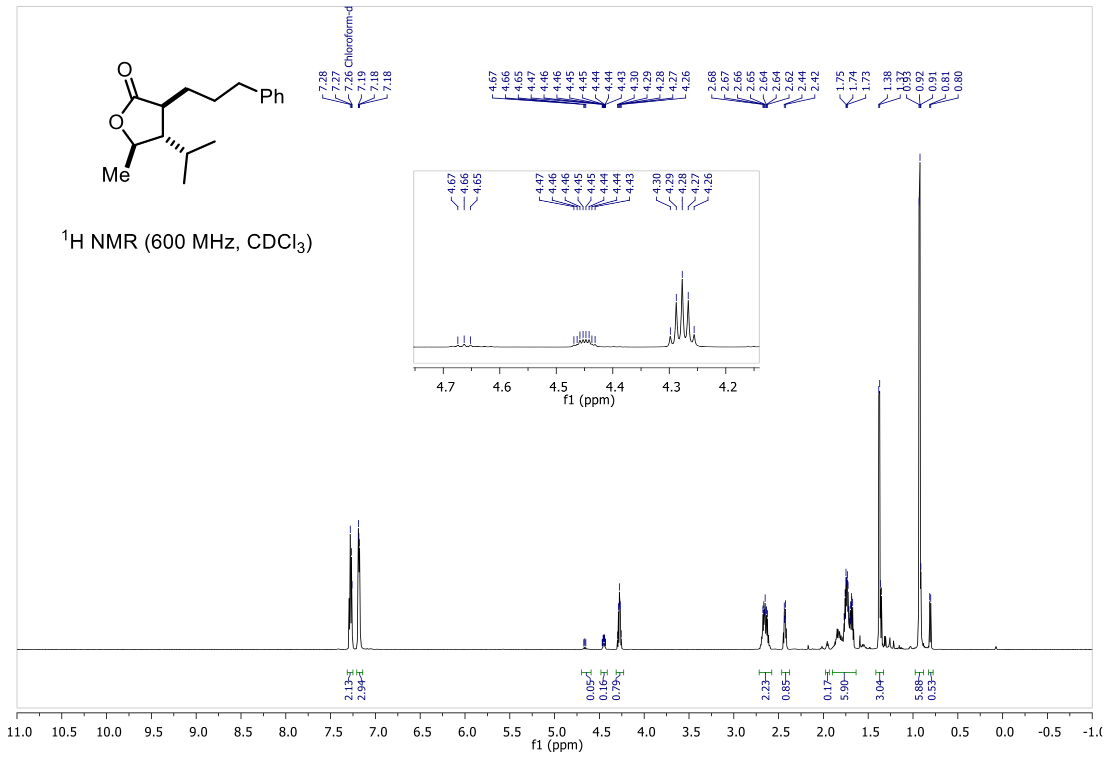

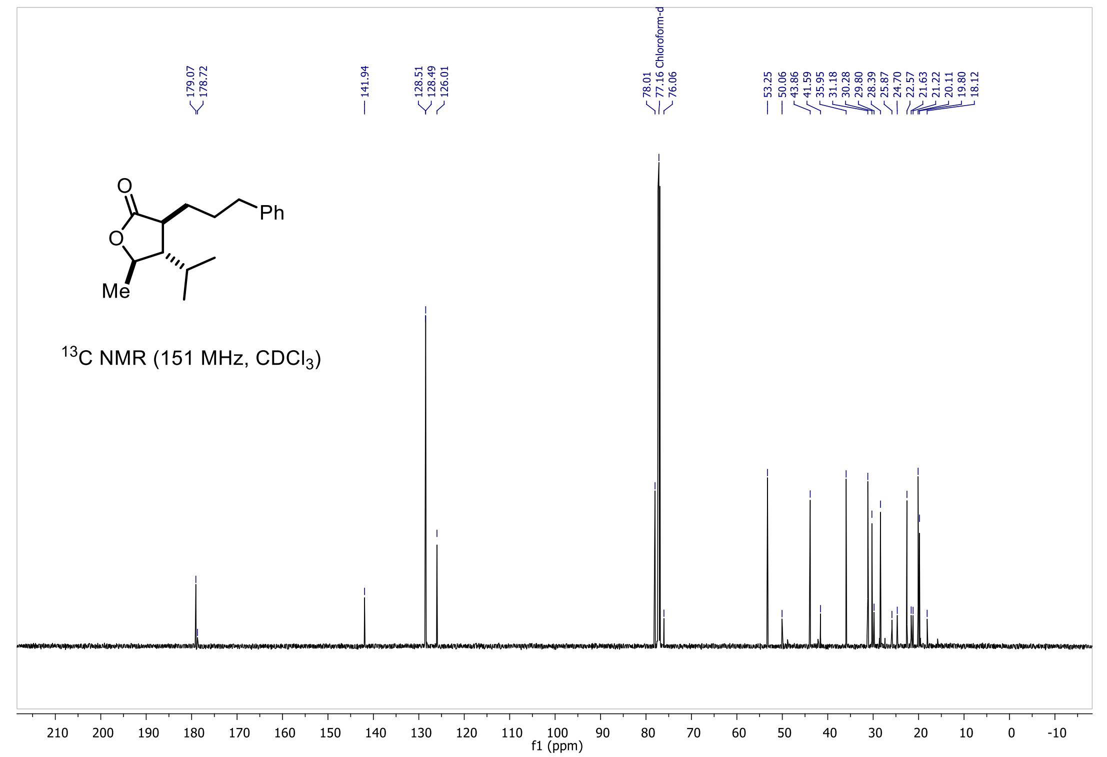

(±)-(3*S*,4*S*,5*R*)-5-Ethyl-4-isopropyl-3-(3-phenylpropyl)dihydrofuran-2(3*H*)-one (11b)

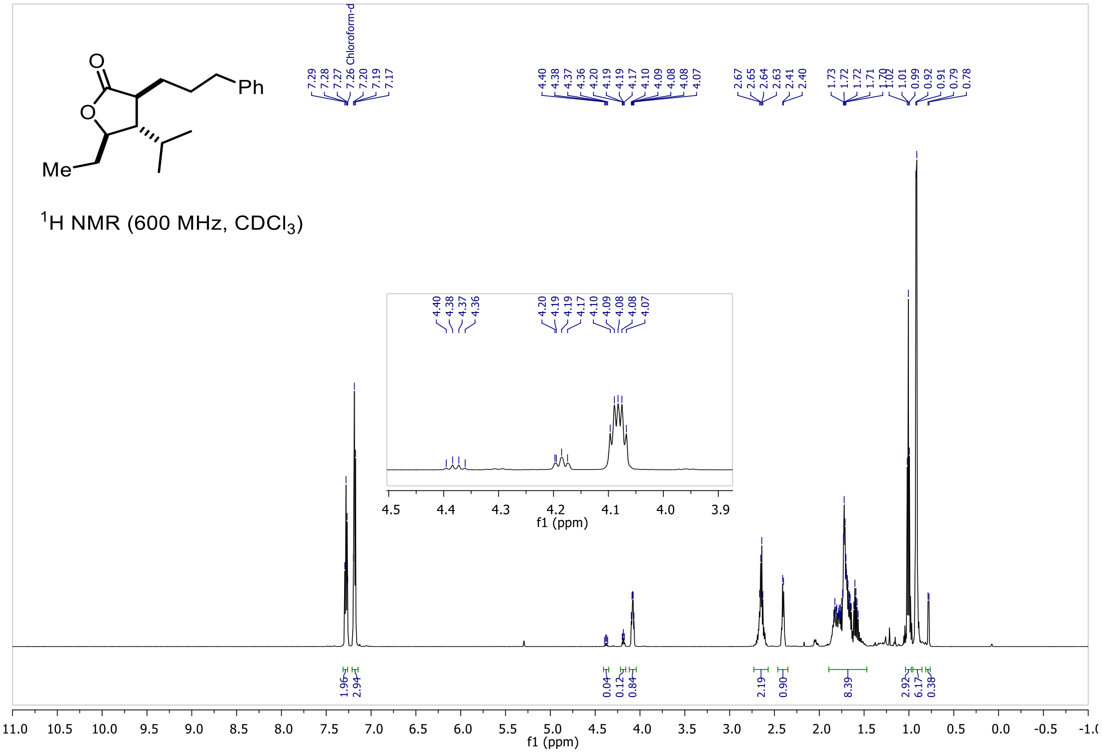

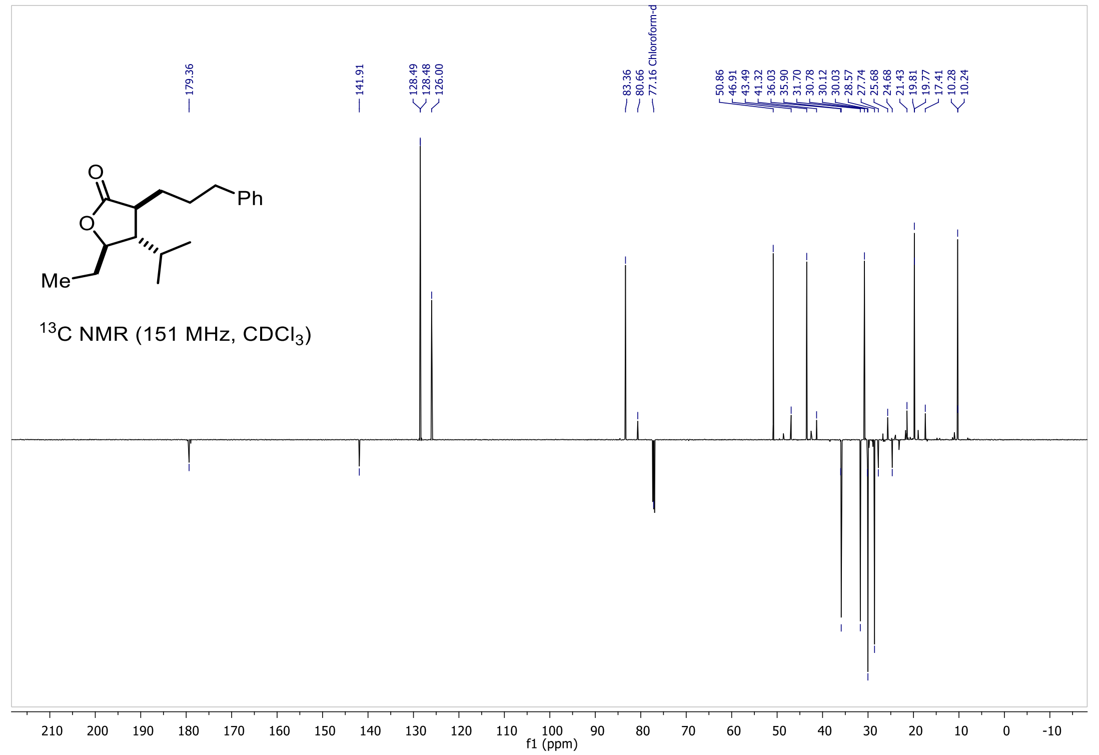

(±)-(3*S*,4*S*,5*R*)- 4-Isopropyl-5-phenethyl-3-(3-phenylpropyl)dihydrofuran-2(3*H*)-one (11c)

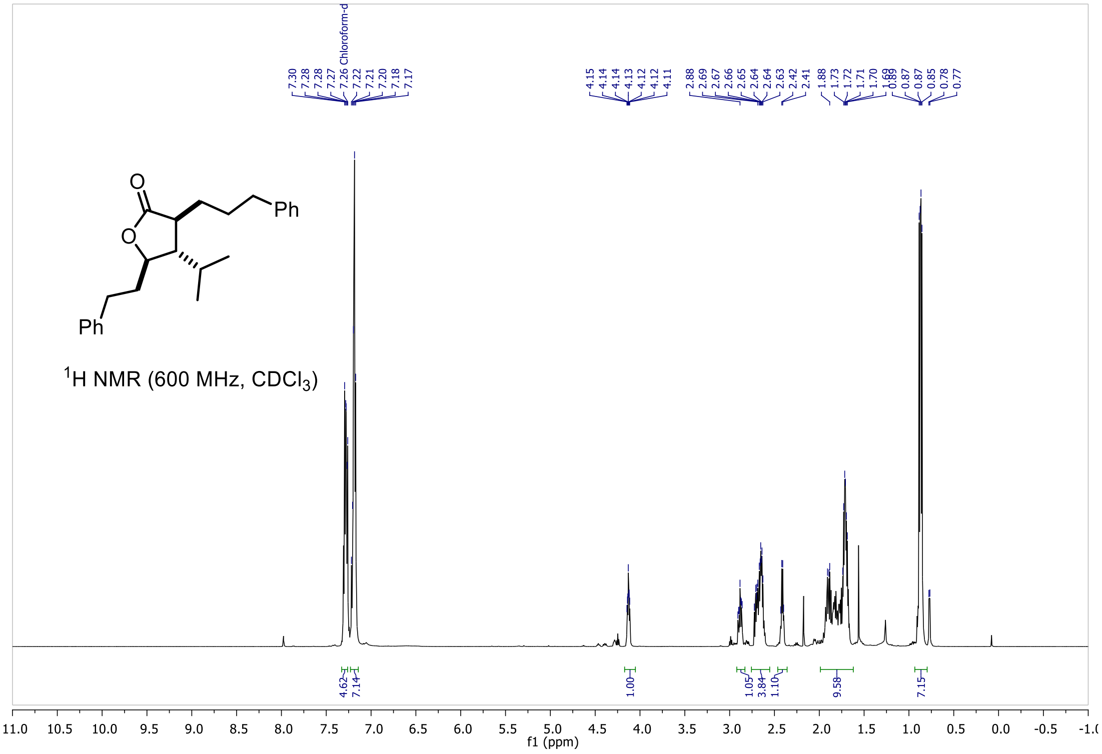

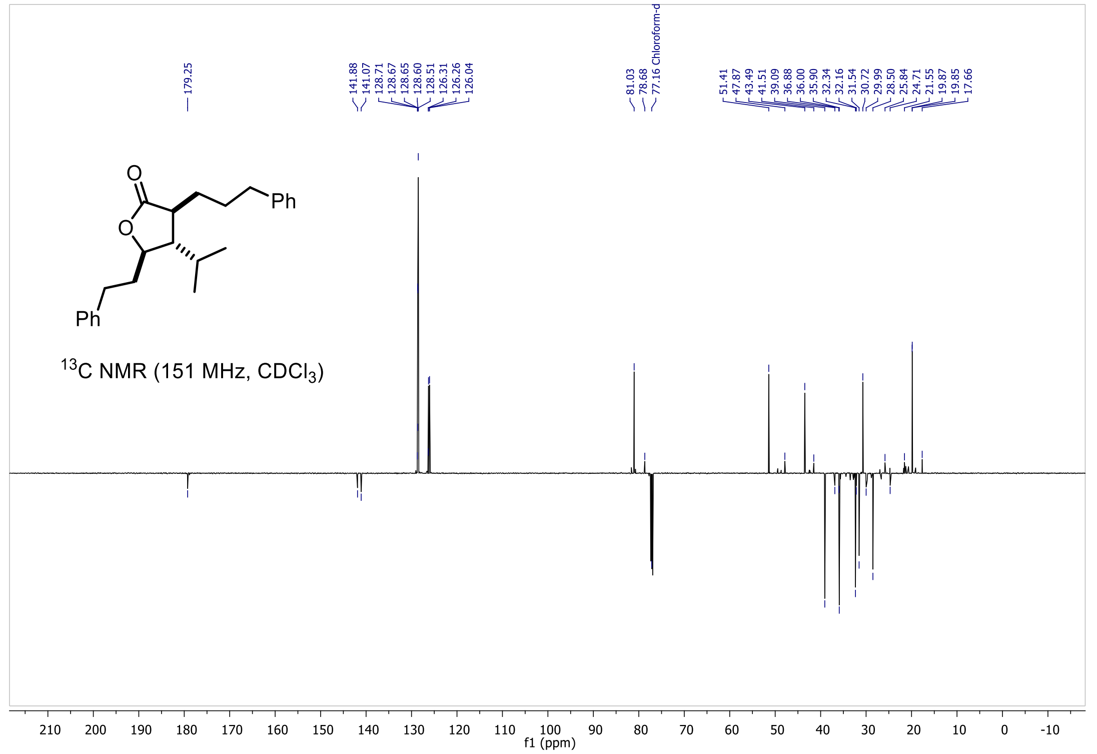

(±)-(3*S*,4*S*,5*R*)-4-Isopropyl-3-(3-phenylpropyl)-5-vinyldihydrofuran-2(3*H*)-one (11d)

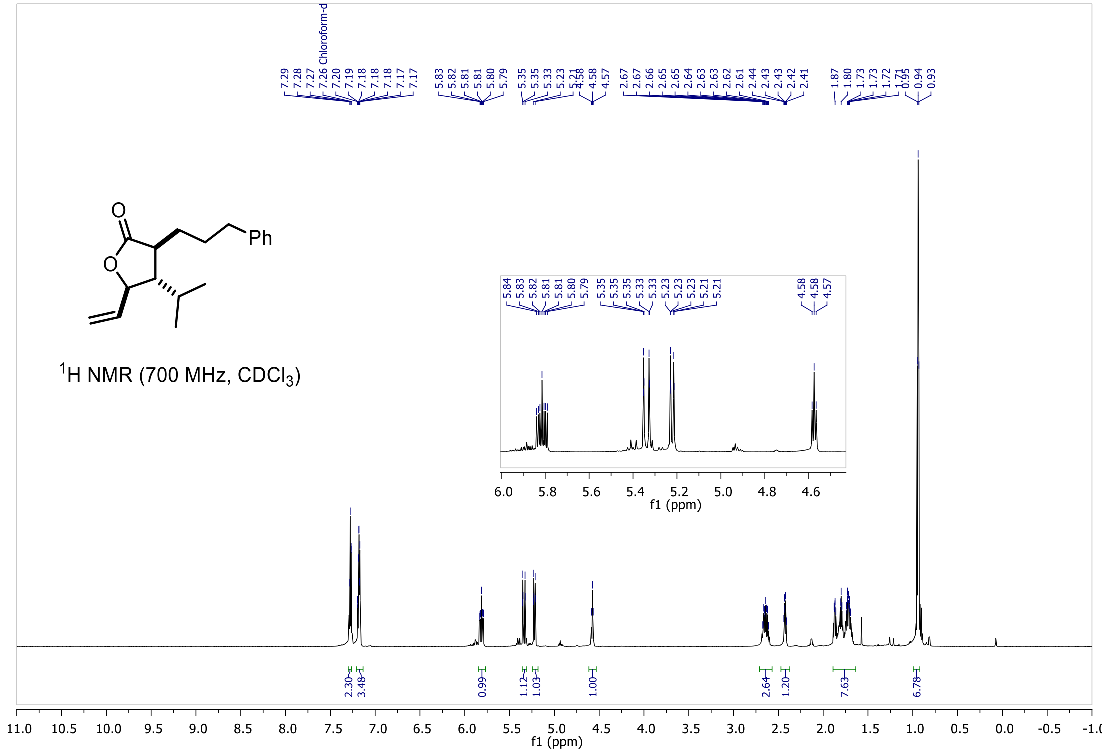

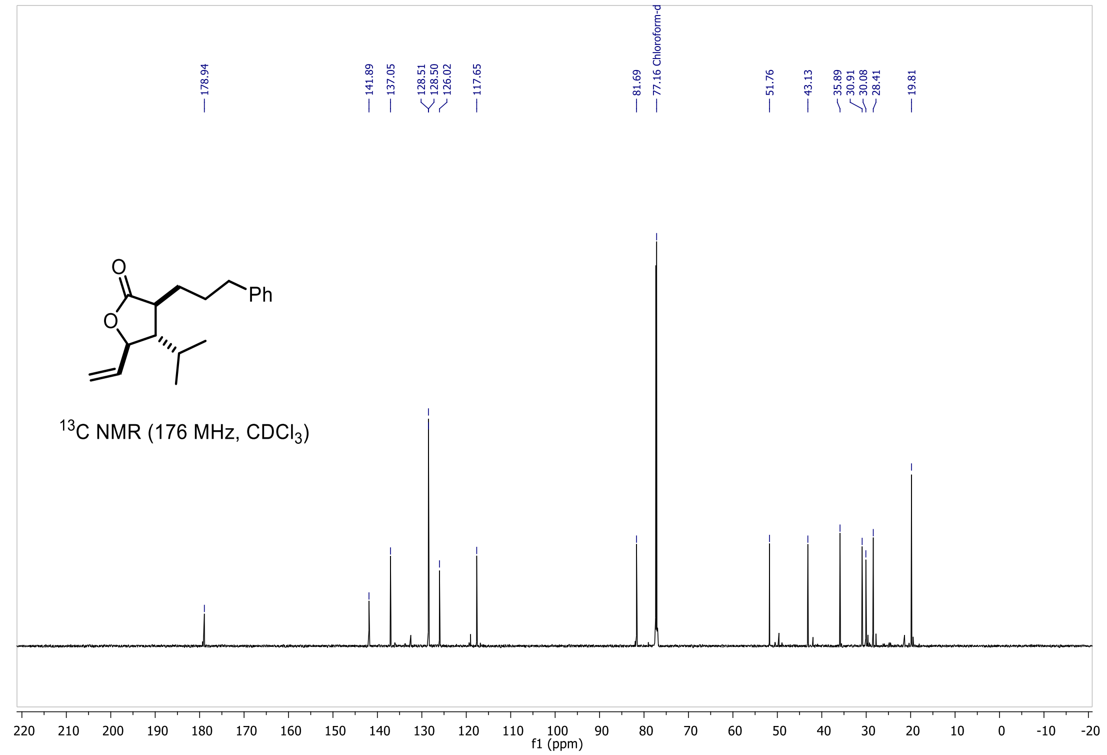

(±)-(3*S*,4*S*,5*S*)-4-Butyl-3-(3-phenylpropyl)-5-[(trimethylsilyl)ethynyl]dihydrofuran-2(3*H*)-one (11e)

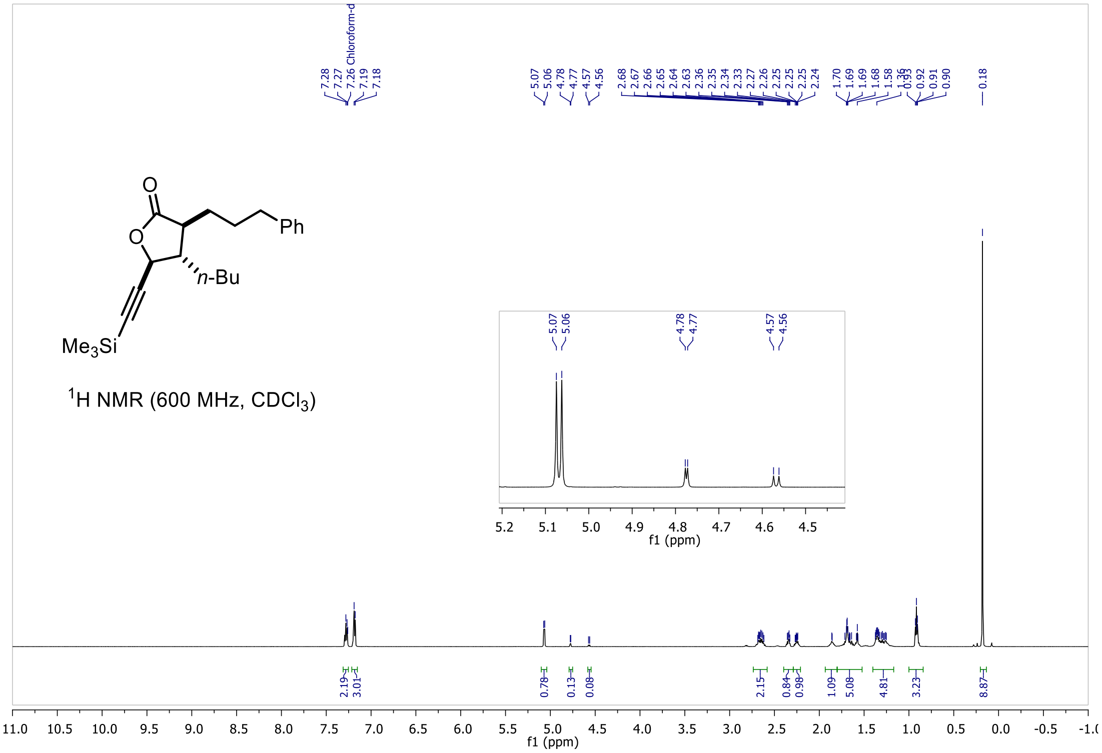

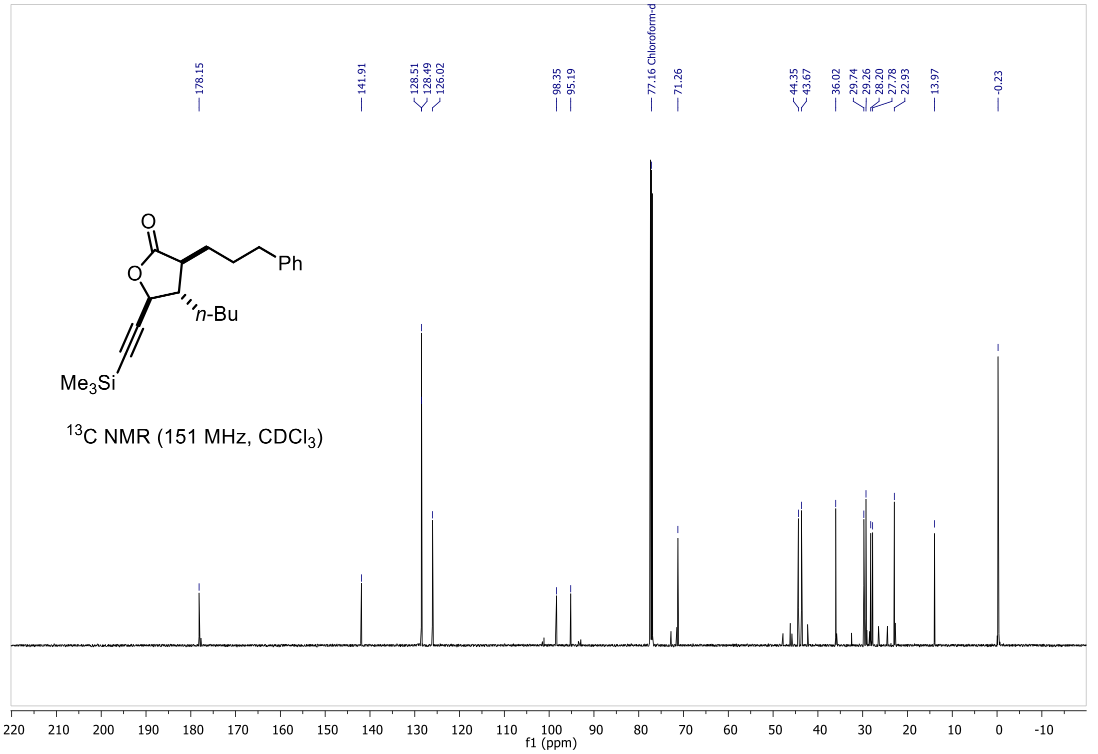

(±)-(3*S*,4*S*,5*R*)-4-Isopropyl-5-(4-methoxyphenyl)-3-(3-phenylpropyl)dihydrofuran-2(3*H*)-one (11f)

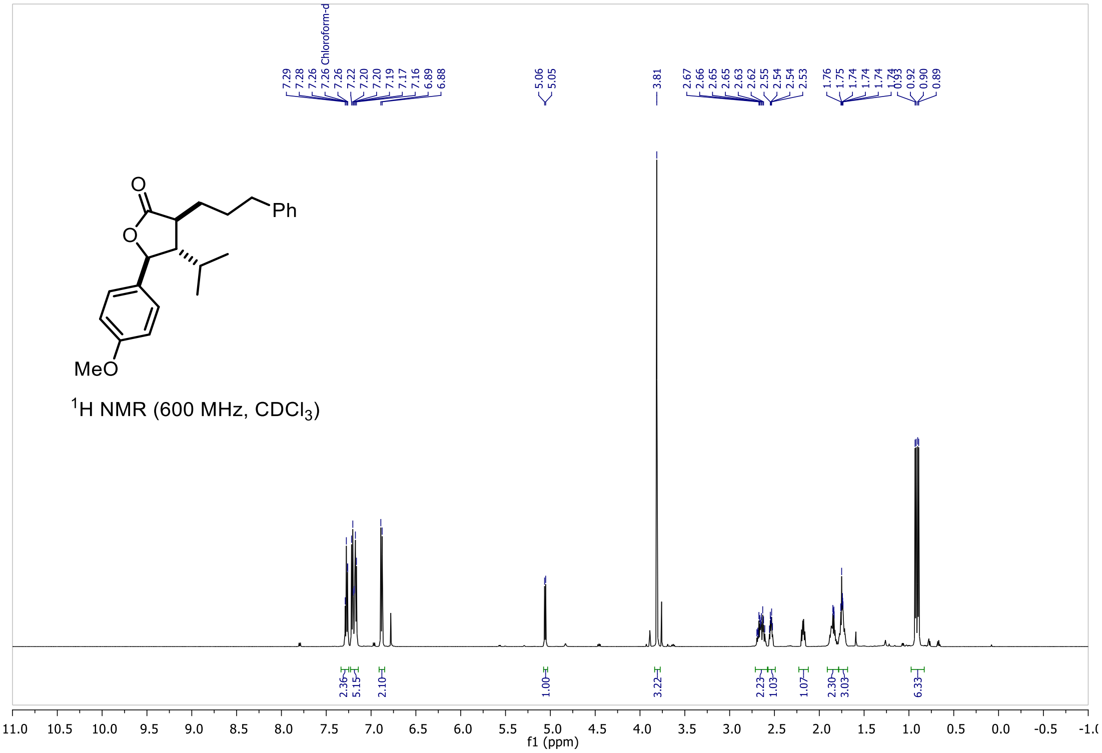

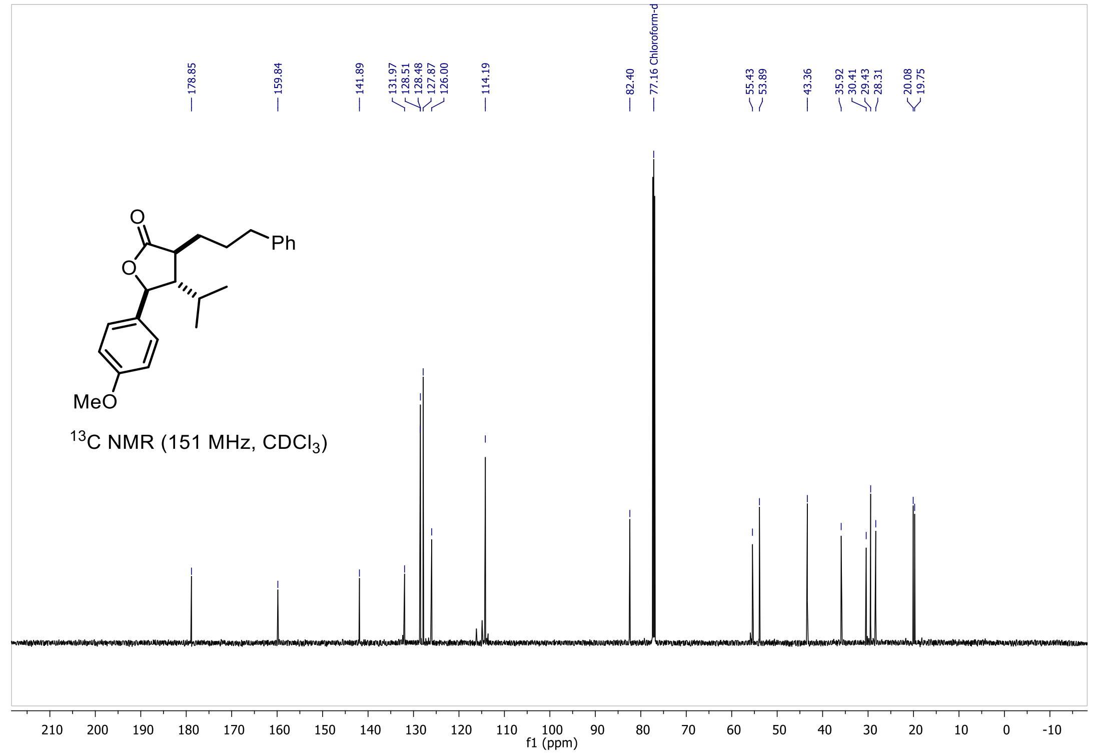

(±)-(3*S*,4*R*,5*S*)-4-Butyl-5-methyl-3-(3-phenylpropyl)dihydrofuran-2(3*H*)-one (11g)

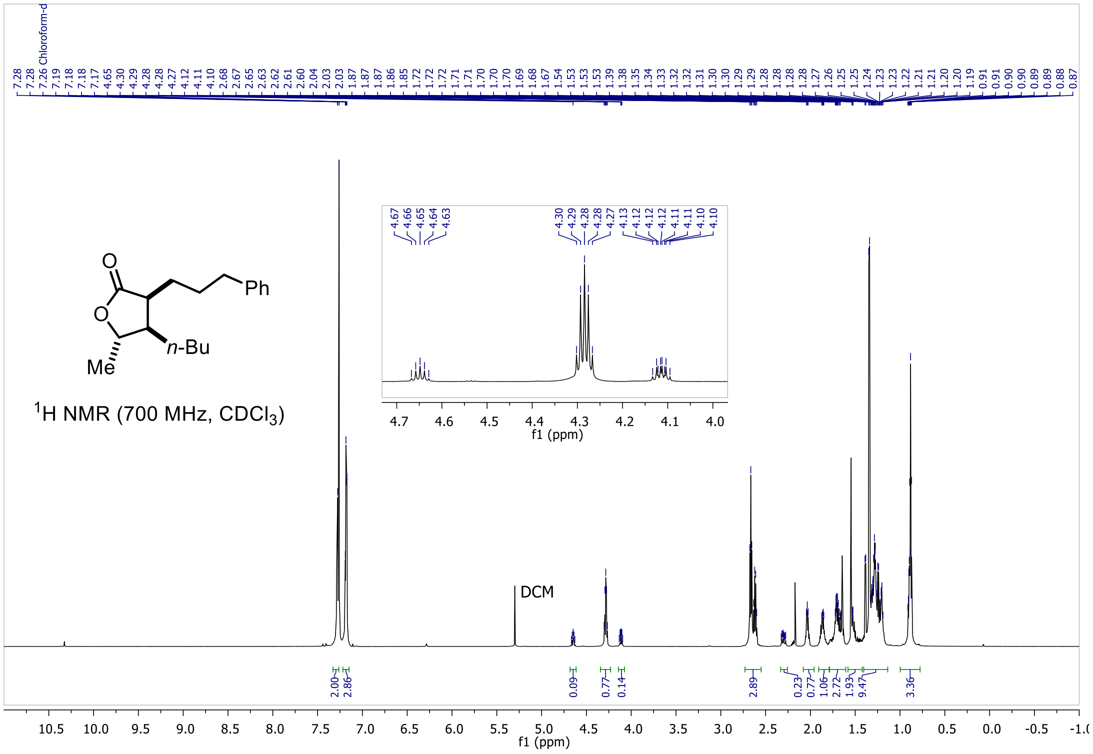

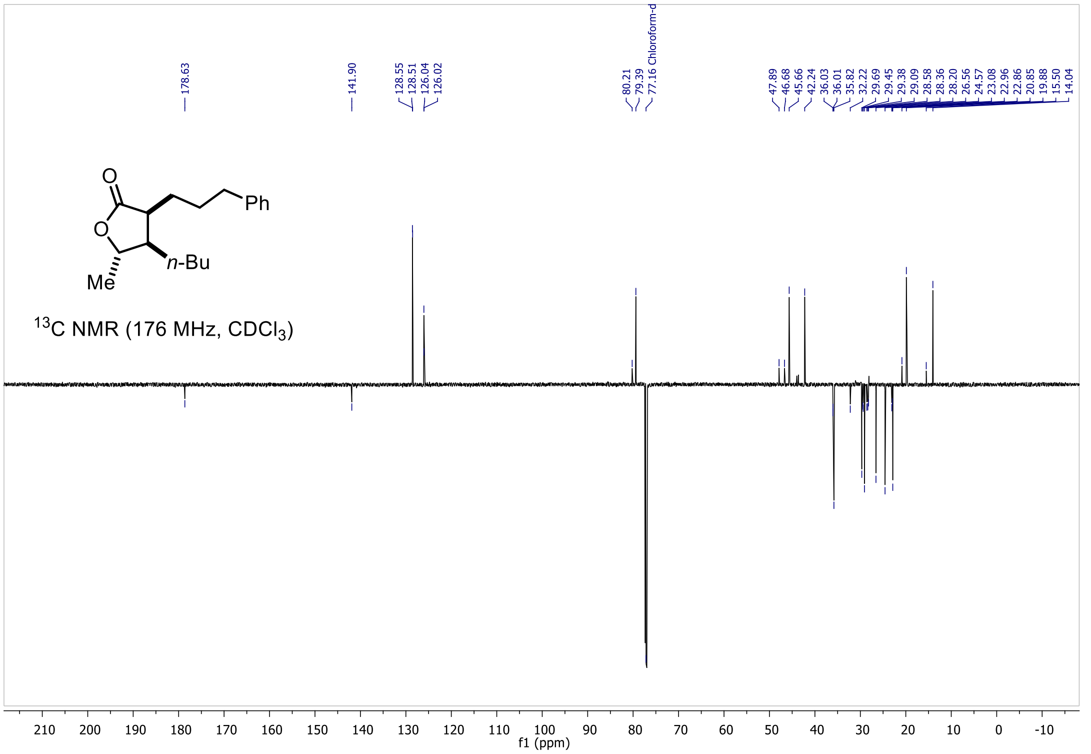

(±)-(3*S*,4*R*,5*S*)-4-Butyl-5-ethyl-3-(3-phenylpropyl)dihydrofuran-2(3*H*)-one (11h)

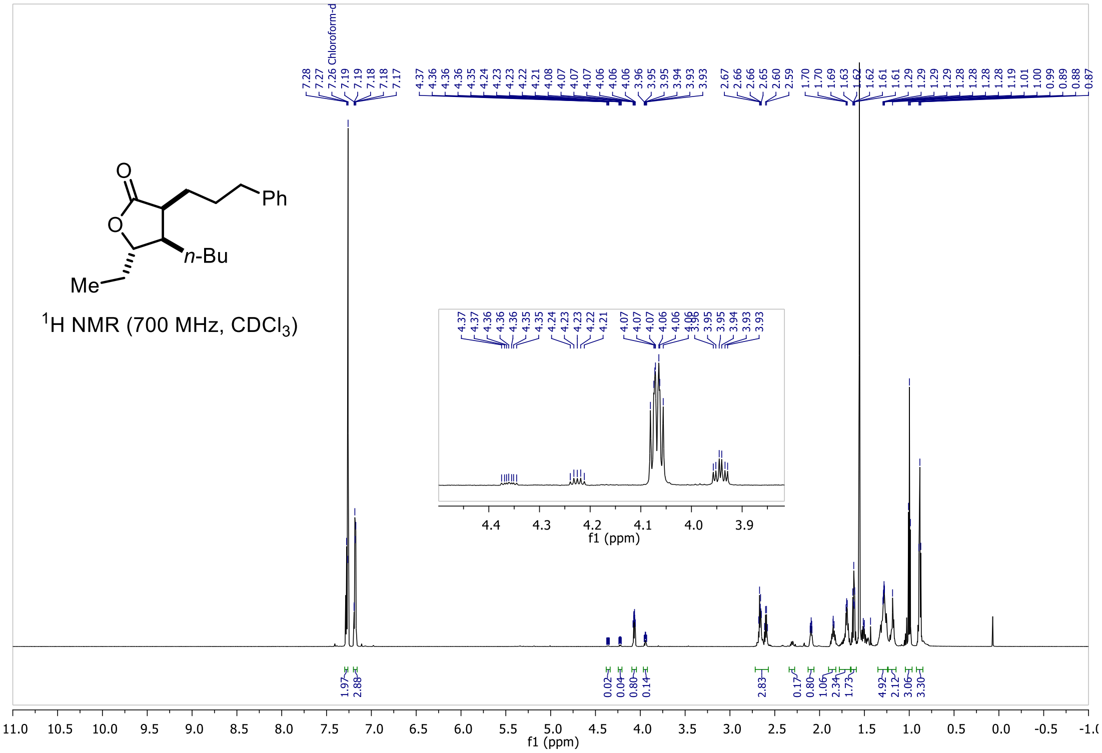

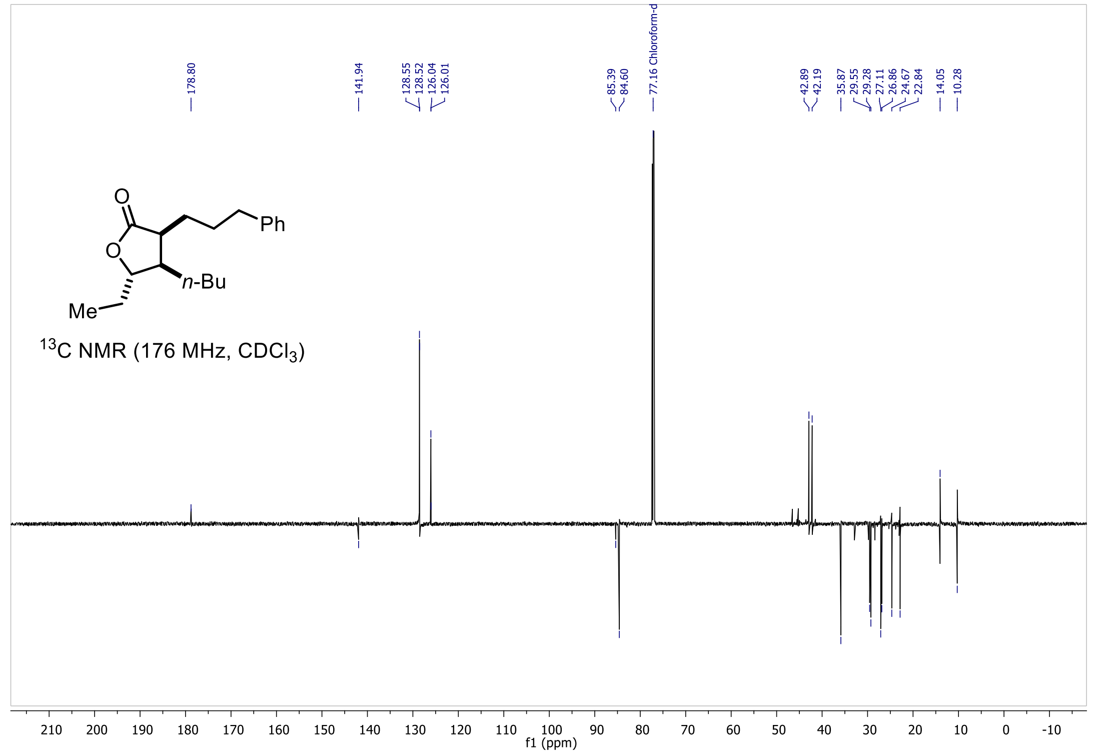

(±)-(3*S*,4*R*,5*R*)-4-Butyl-3-(3-phenylpropyl)-5-[4-(trifluoromethyl)phenyl]dihydrofuran-2(3*H*)-one (11i)

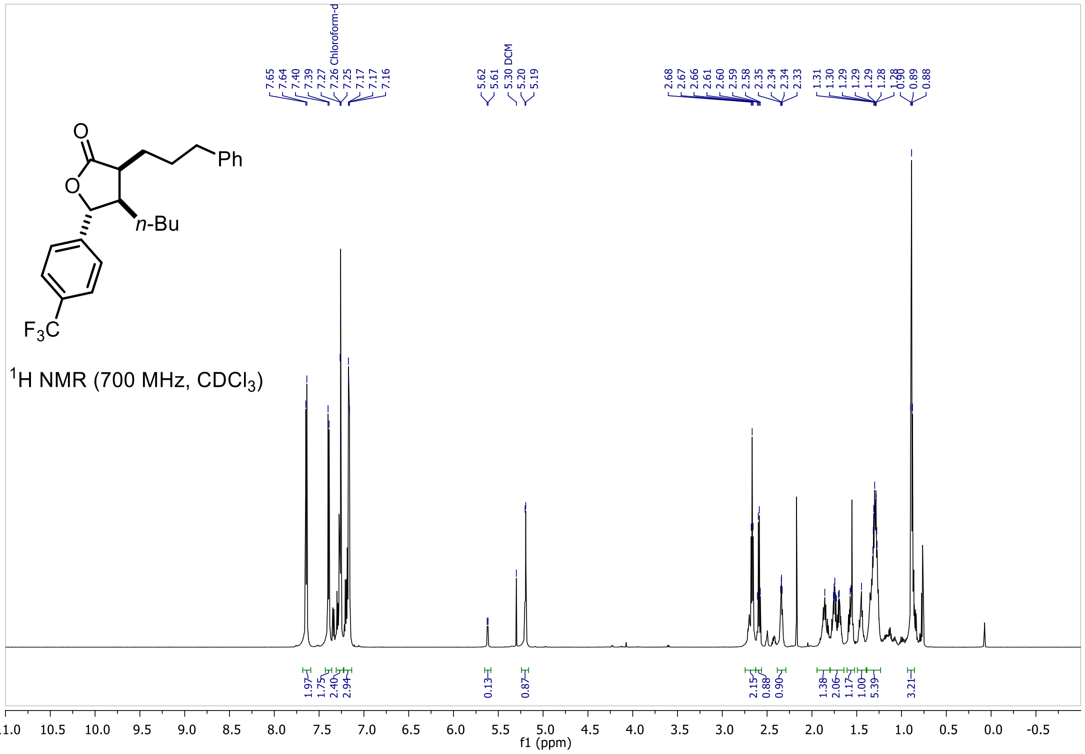

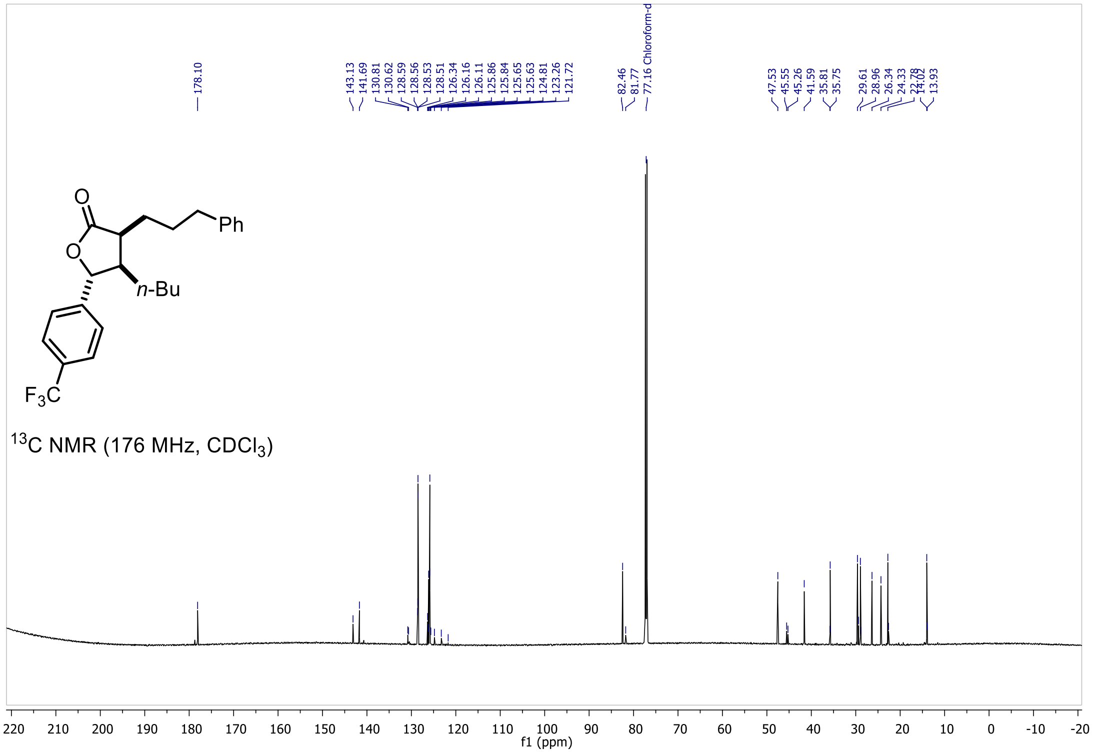

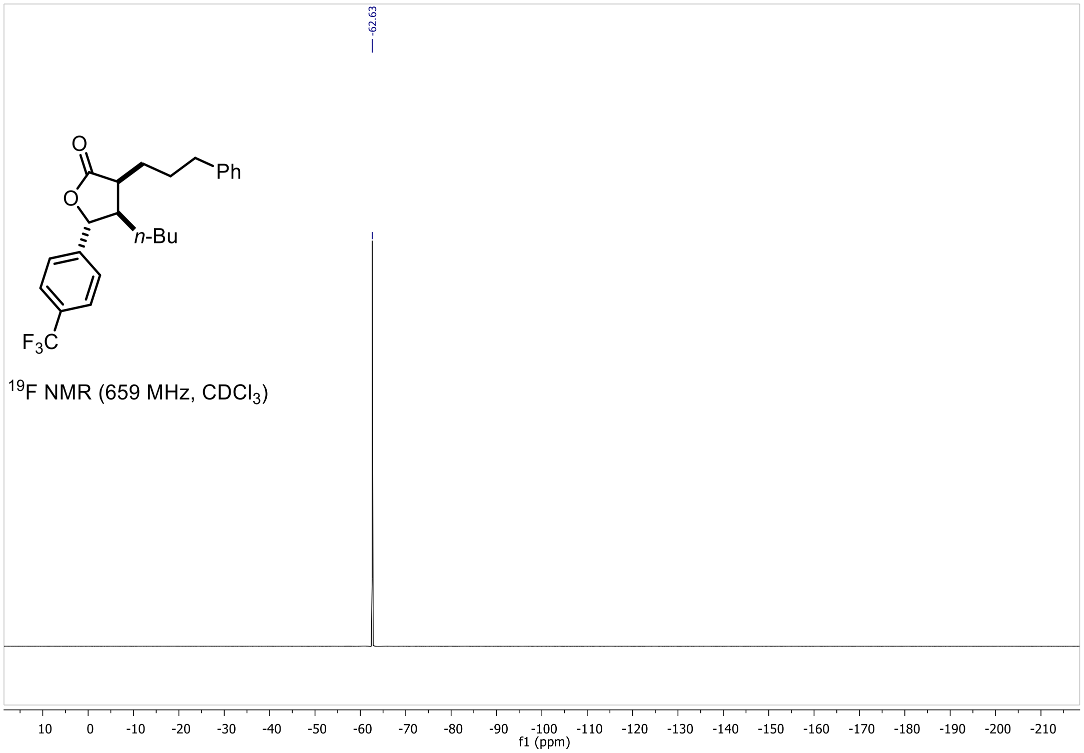

-62.63
O
O
Ph
n-Bu
F3C
19F NMR (659 MHz, CDCl3)
10
0
-10
-20
-30
-40
-50
-60
-70
-80
-90
-100
-110
-120
-130
-140
-150
-160
-170
-180
-190
-200
-210
f1 (ppm)

(±)-(3*S*,4*S*,5*R*)-4-Butyl-5-ethyl-4-methyl-3-(3-phenylpropyl)dihydrofuran-2(3*H*)-one (11j)

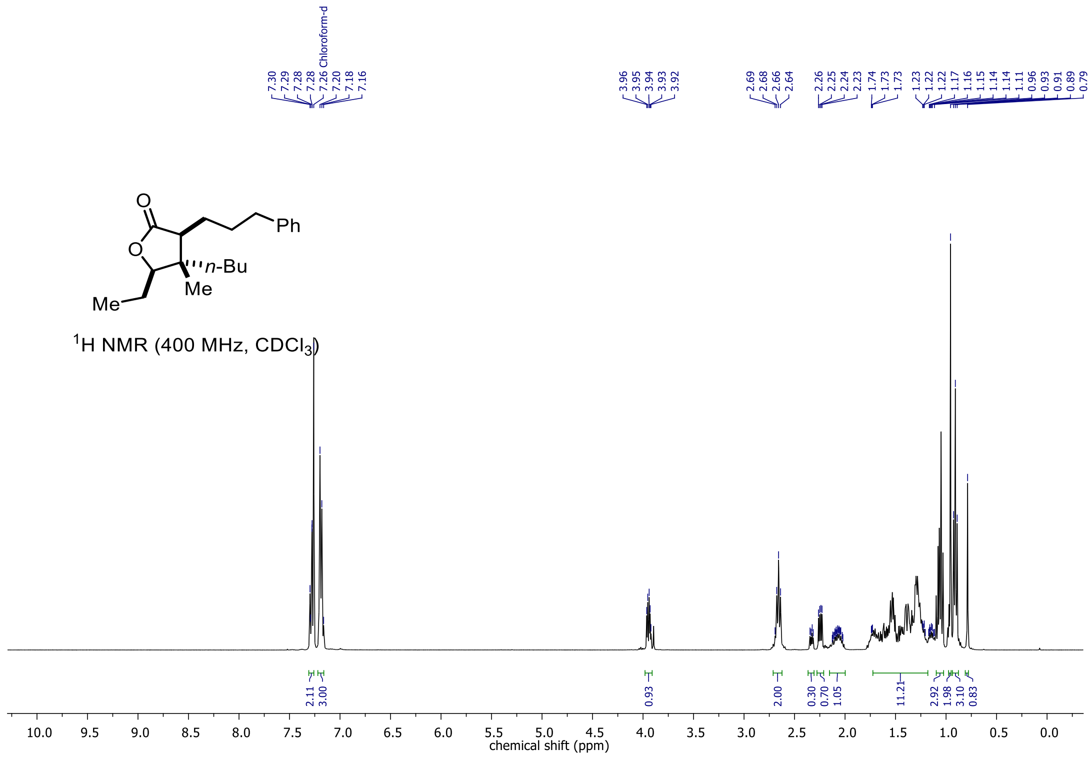

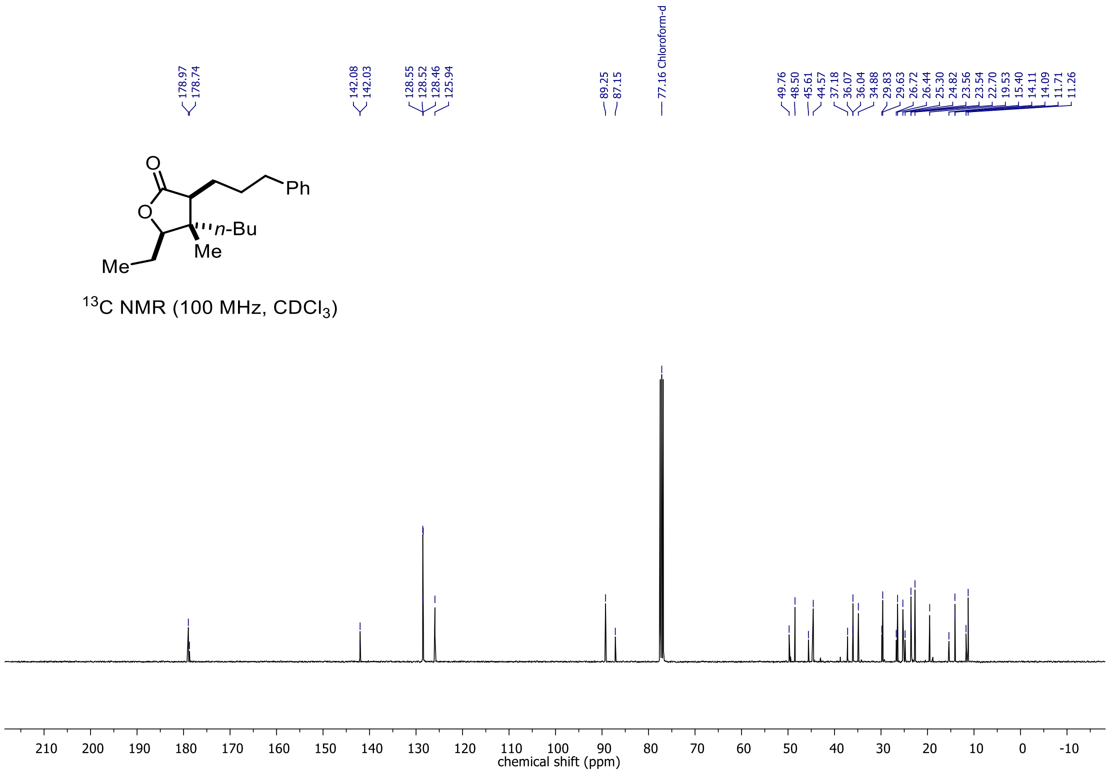

(±)-(3*S*,4*R*,5*R*)-4-Butyl-5-ethyl-4-methyl-3-(3-phenylpropyl)dihydrofuran-2(3*H*)-one (11k)

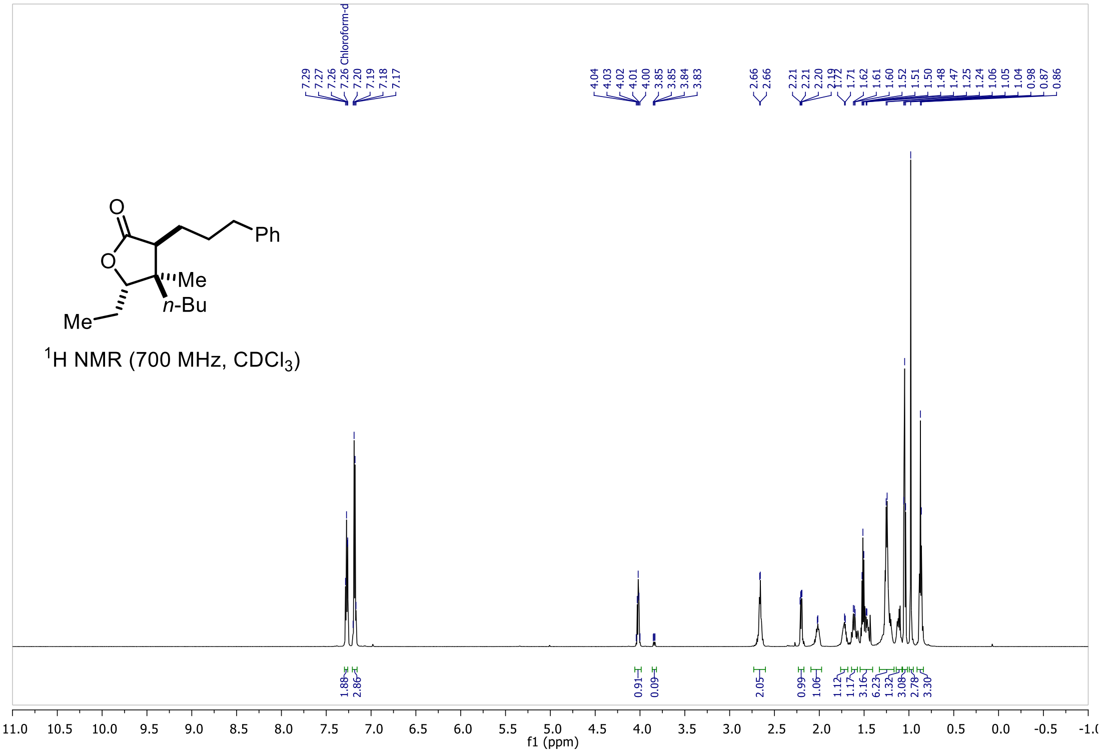

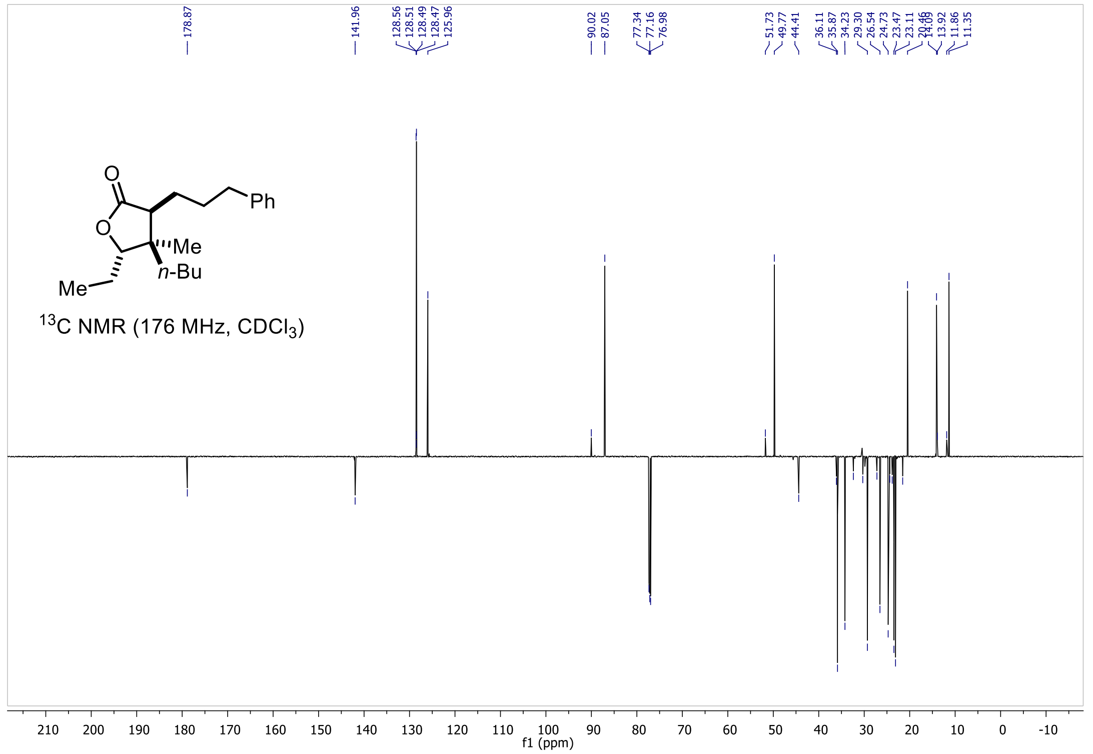

(±)-(3*S*,4*R*,5*S*)-4-Butyl-4-methyl-3-(3-phenylpropyl)-5-vinyldihydrofuran-2(3*H*)-one (11l)

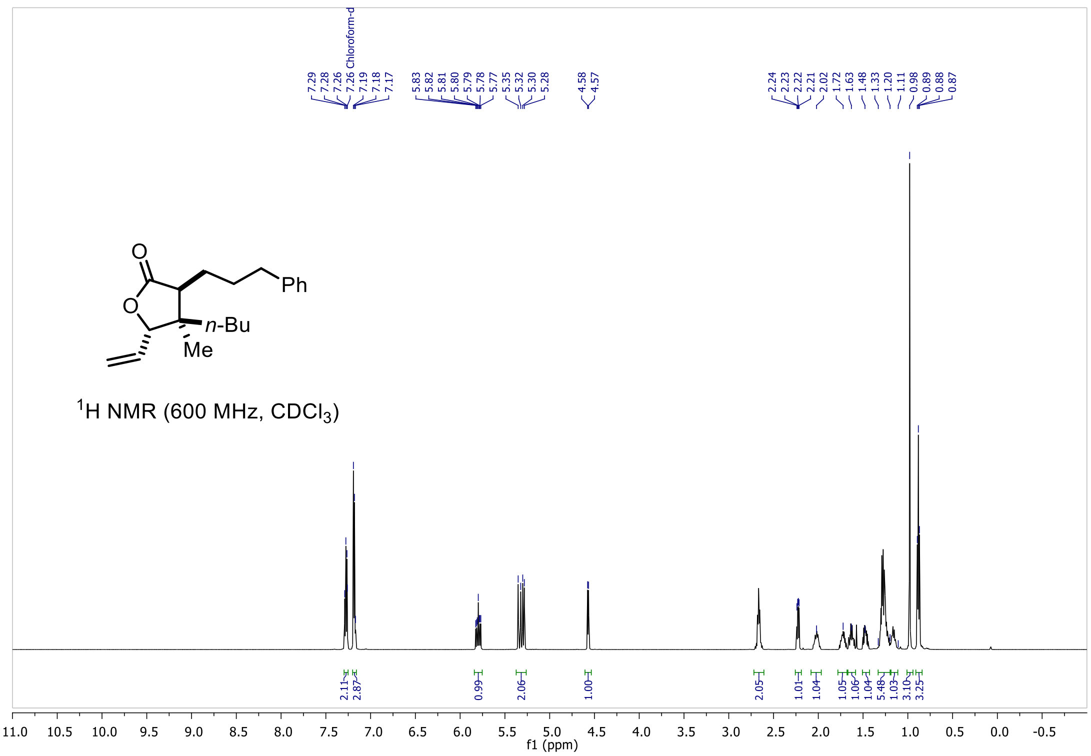

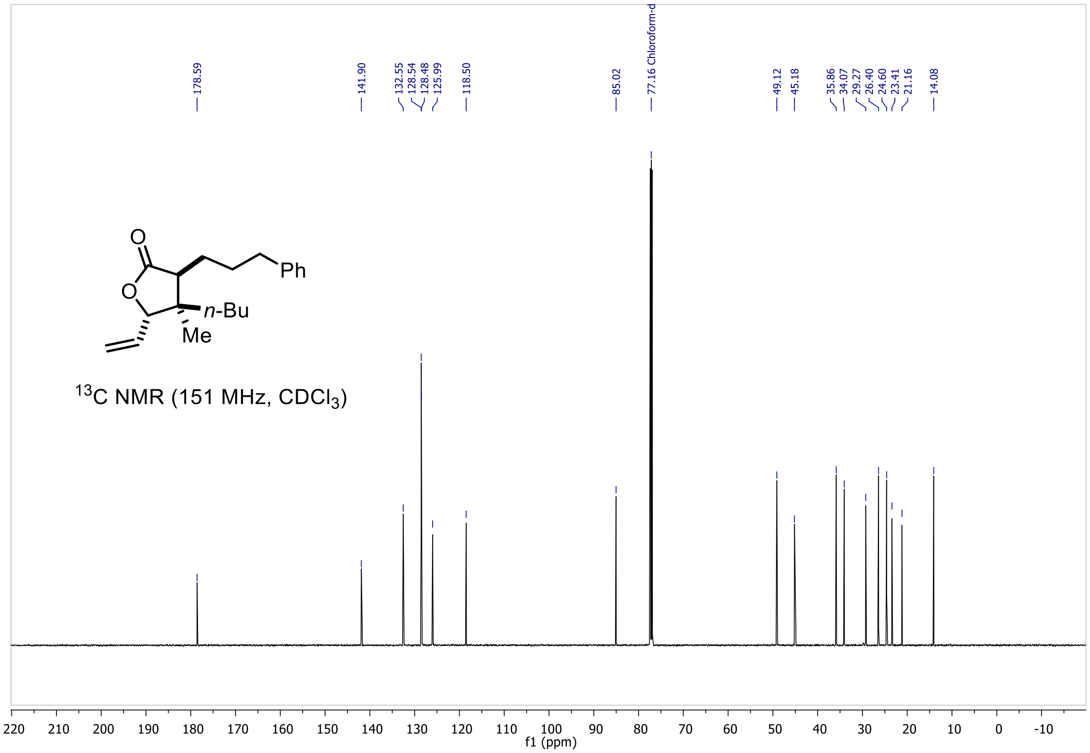

(±)-(3*S*,4*R*,5*R*)-4-Butyl-4-methyl-3-(3-phenylpropyl)-5-[(trimethylsilyl)ethynyl]dihydrofuran-2(3*H*)-one (11m)

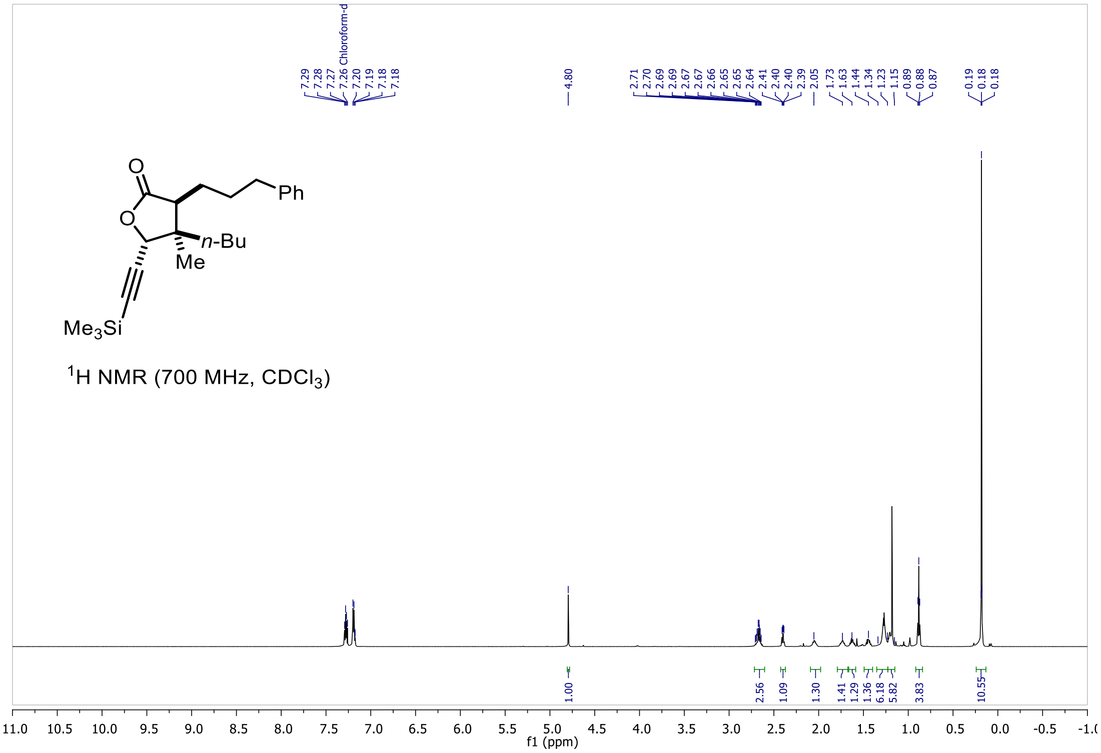

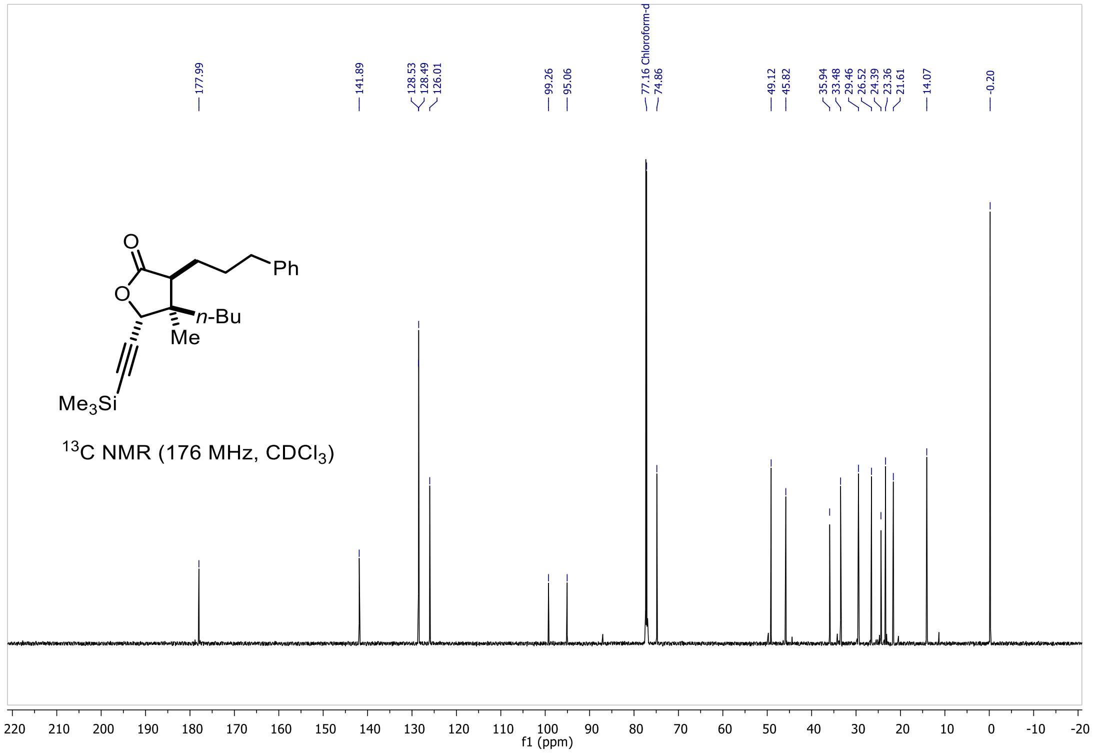

(±)-(3*S*,4*R*,5*R*)-4-Butyl-5-(4-methoxyphenyl)-4-methyl-3-(3-phenylpropyl)dihydrofuran-2(3*H*)-one (11n)

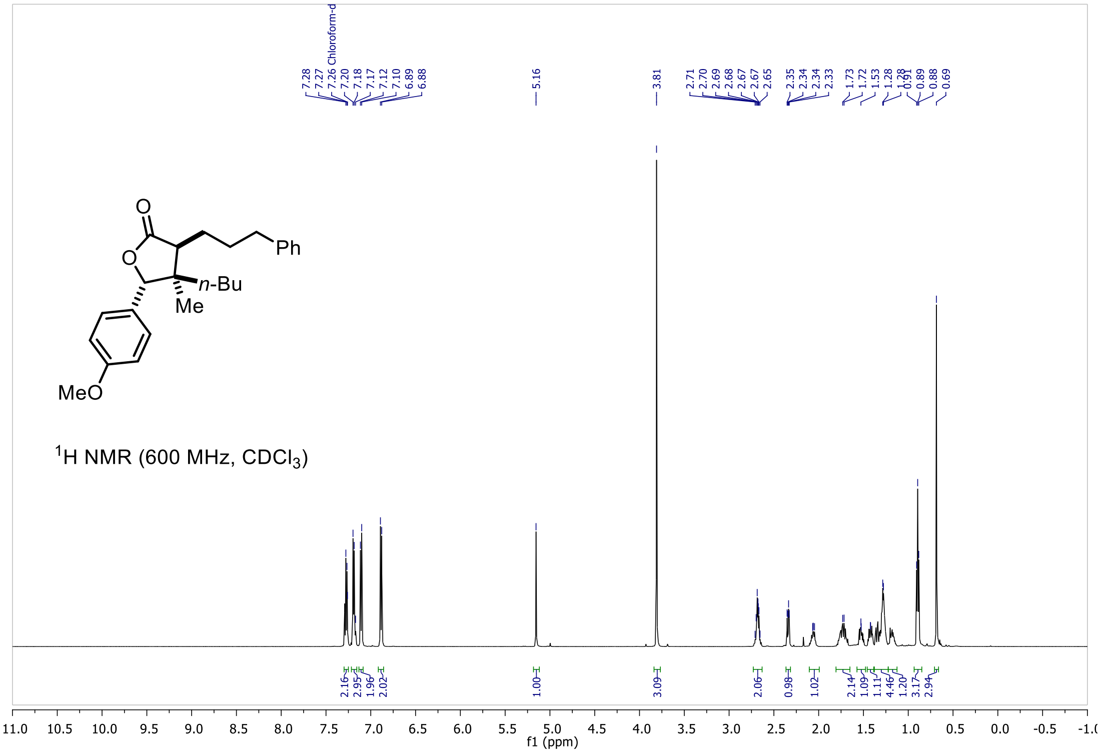

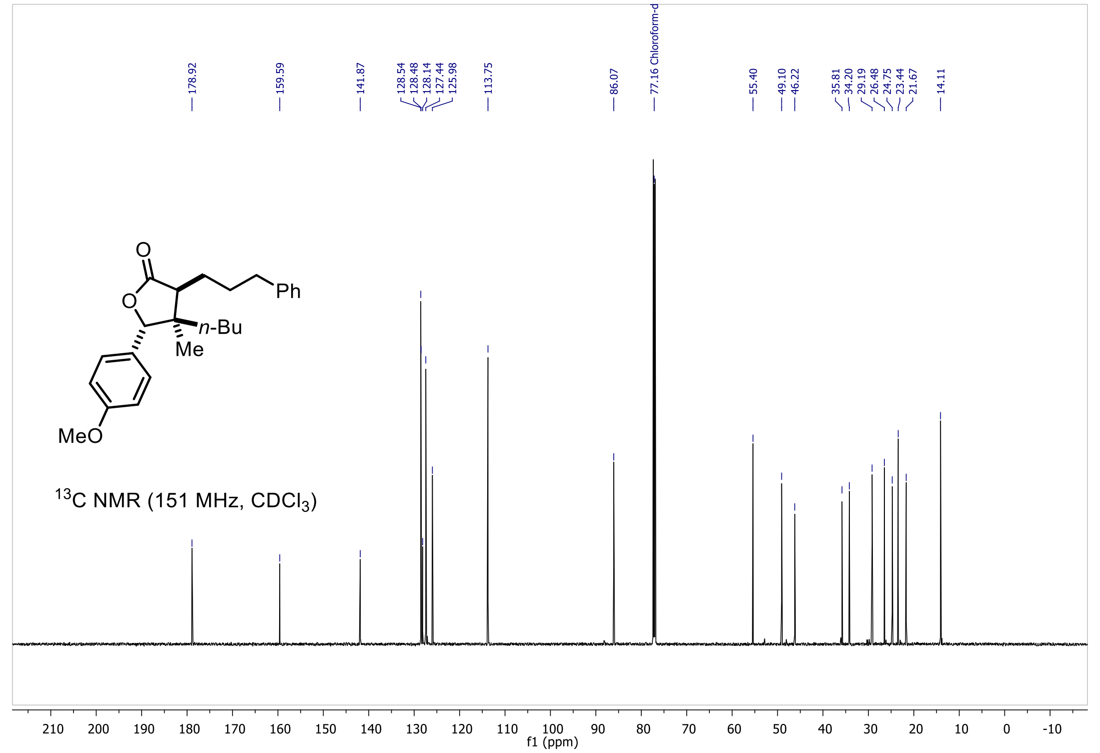

(±)-(3*S*,4*R*,5*R*)-4-Butyl-4-methyl-3-(3-phenylpropyl)-5-[4-(trifluoromethyl)phenyl]dihydrofuran-2(3*H*)-one (11o)

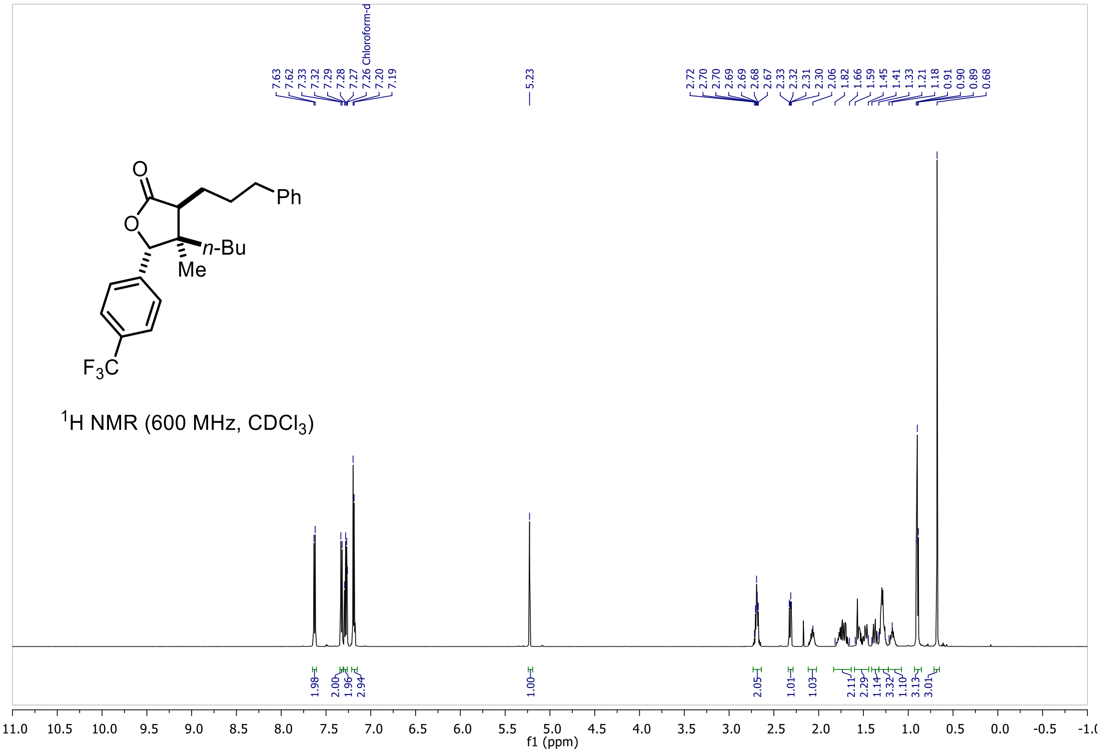

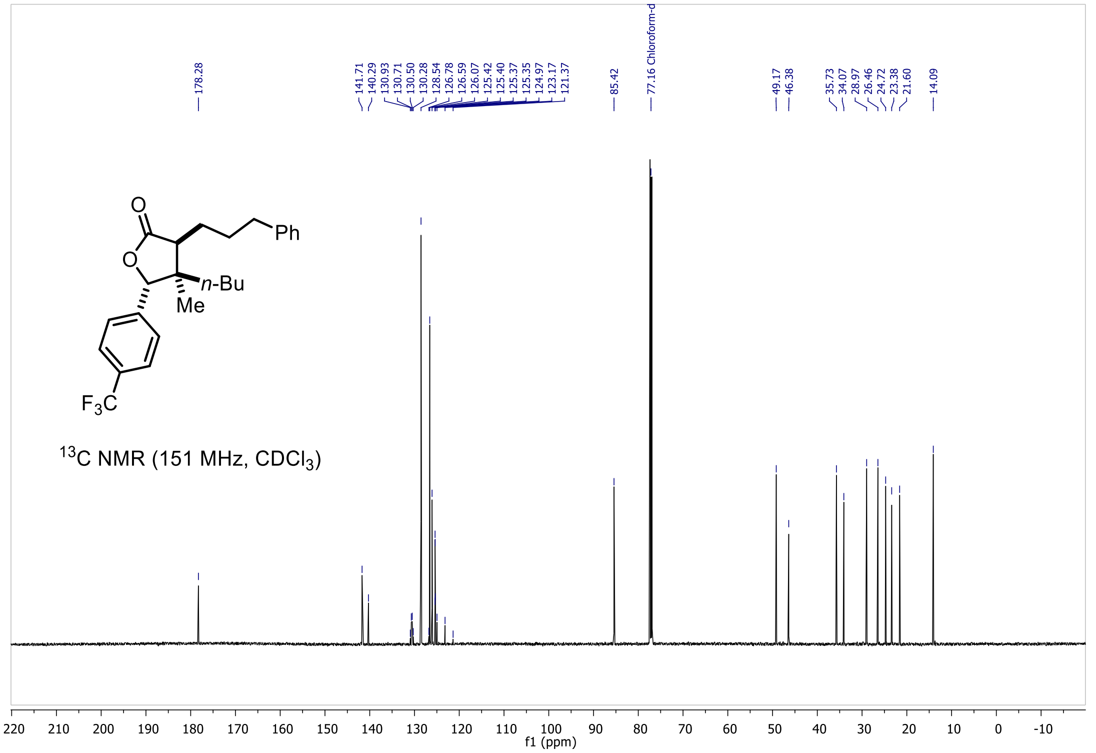

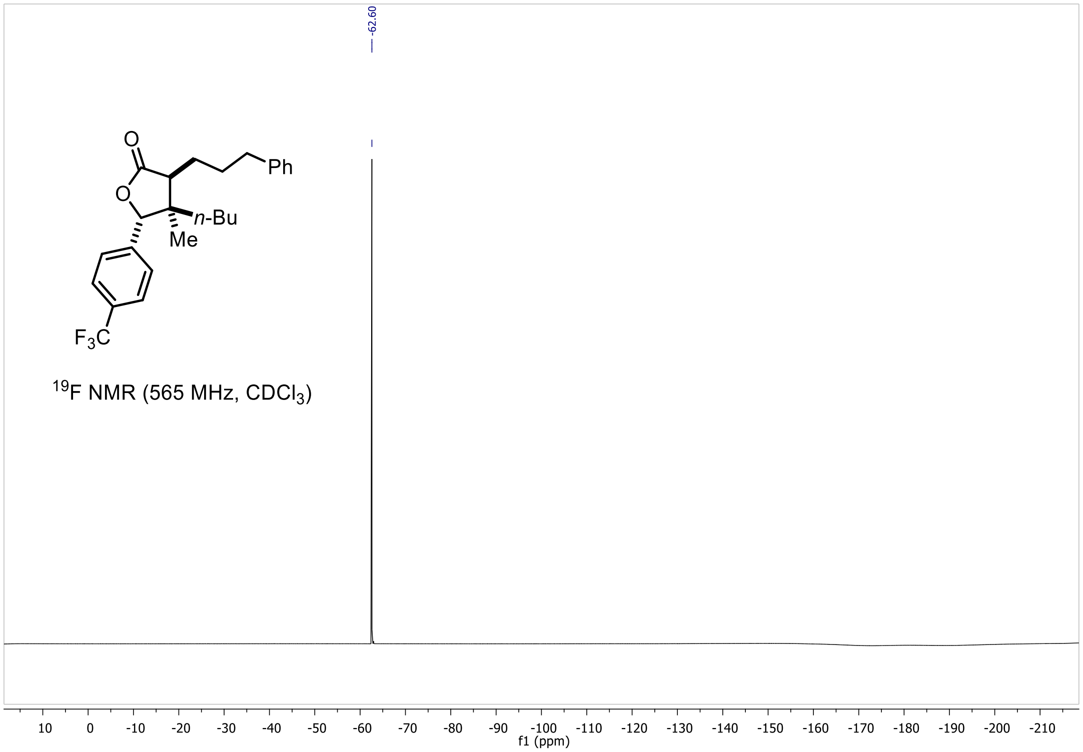
-62.60
Ph
n-Bu
Me
F3C
19F NMR (565 MHz, CDCl3)
f1 (ppm)

(±)–(3*S*,4*S*,5*R*)-5-Allyl-3-butyl-4-isopropyldihydrofuran-2(3*H*)-one (11p)

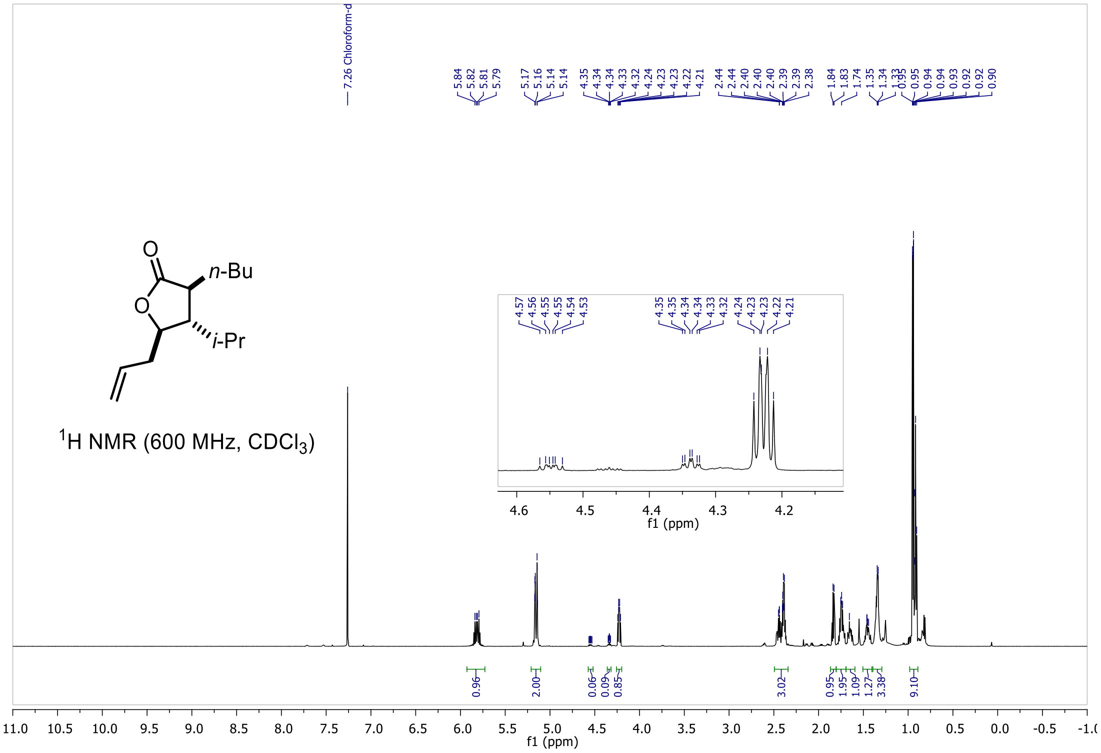

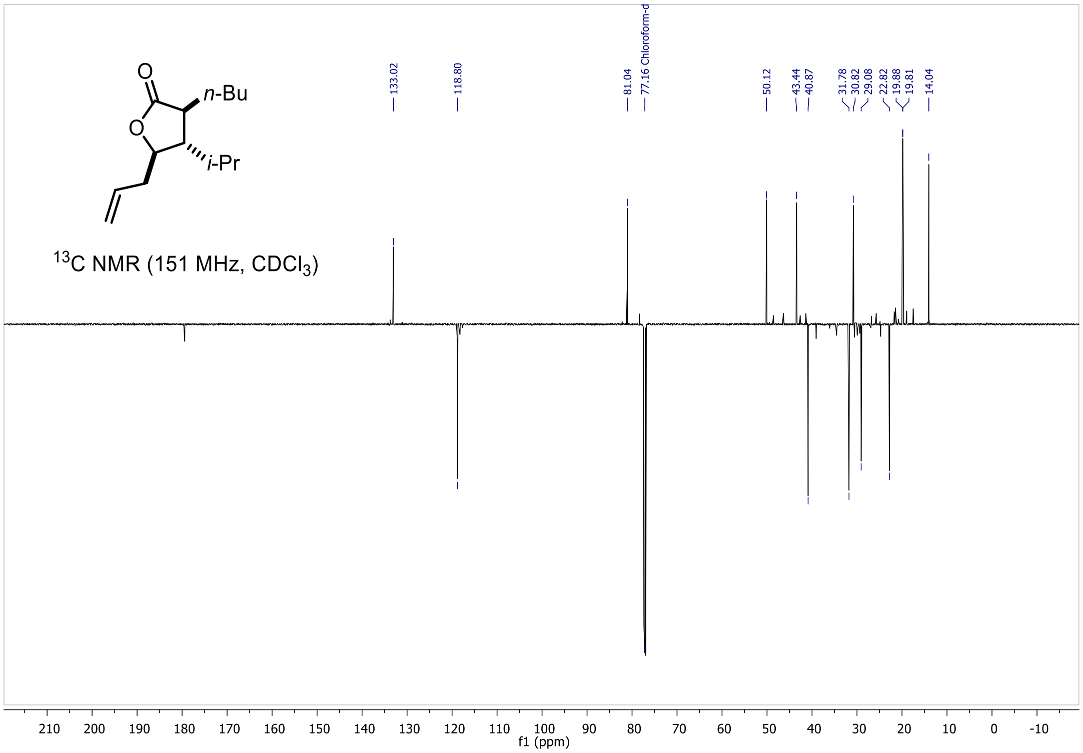

(±)-(3*S*,4*S*,5*R*)-5-Allyl-4-isopropyl-3-(3-phenylpropyl)dihydrofuran-2(3*H*)-one (11q)

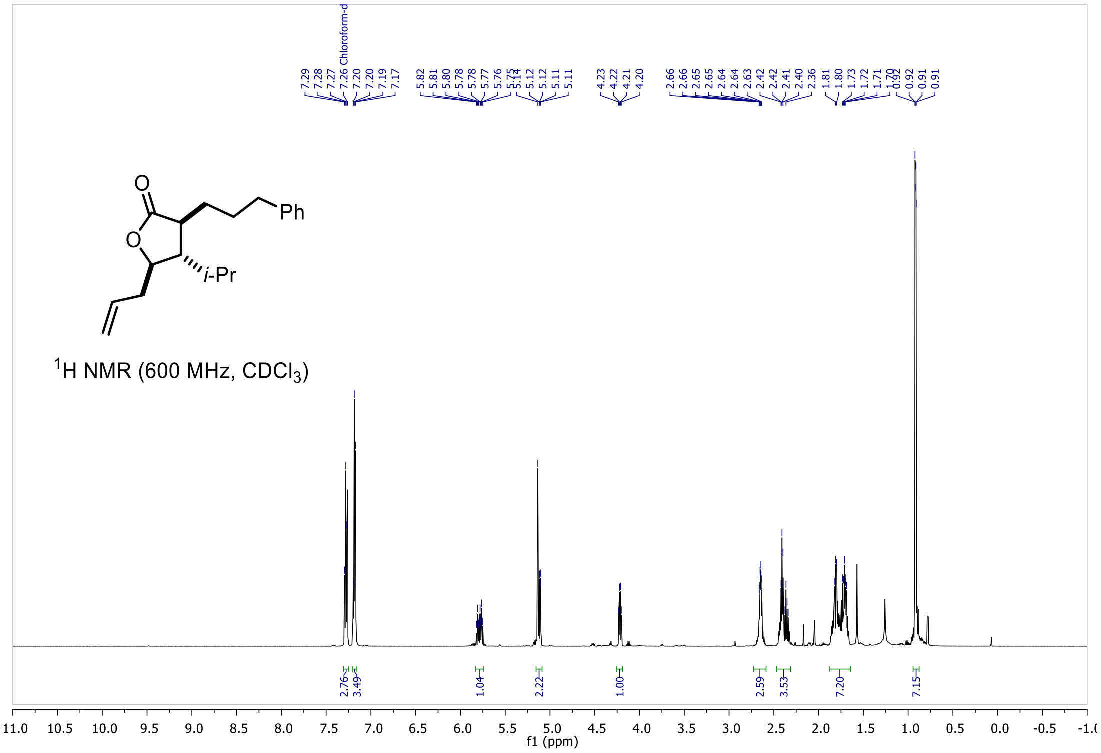

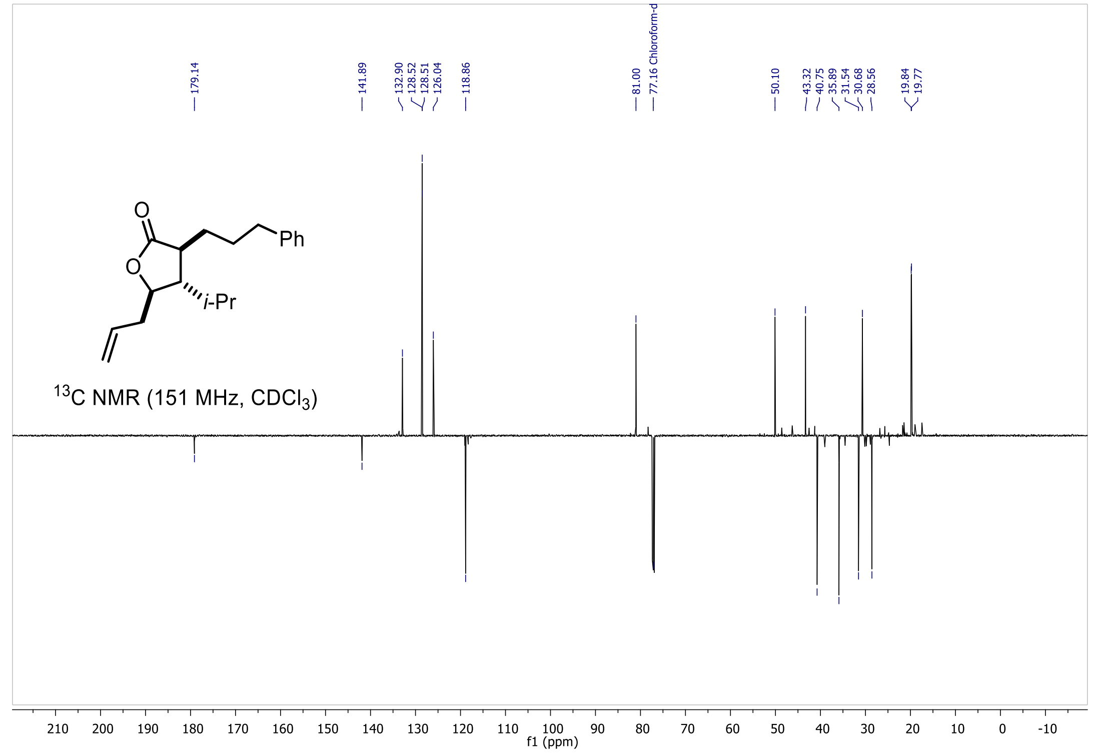

(±)–(3*S*,4*R*,5*S*)-5-Allyl-3,4-dibutyldihydrofuran-2(3*H*)-one (11r)

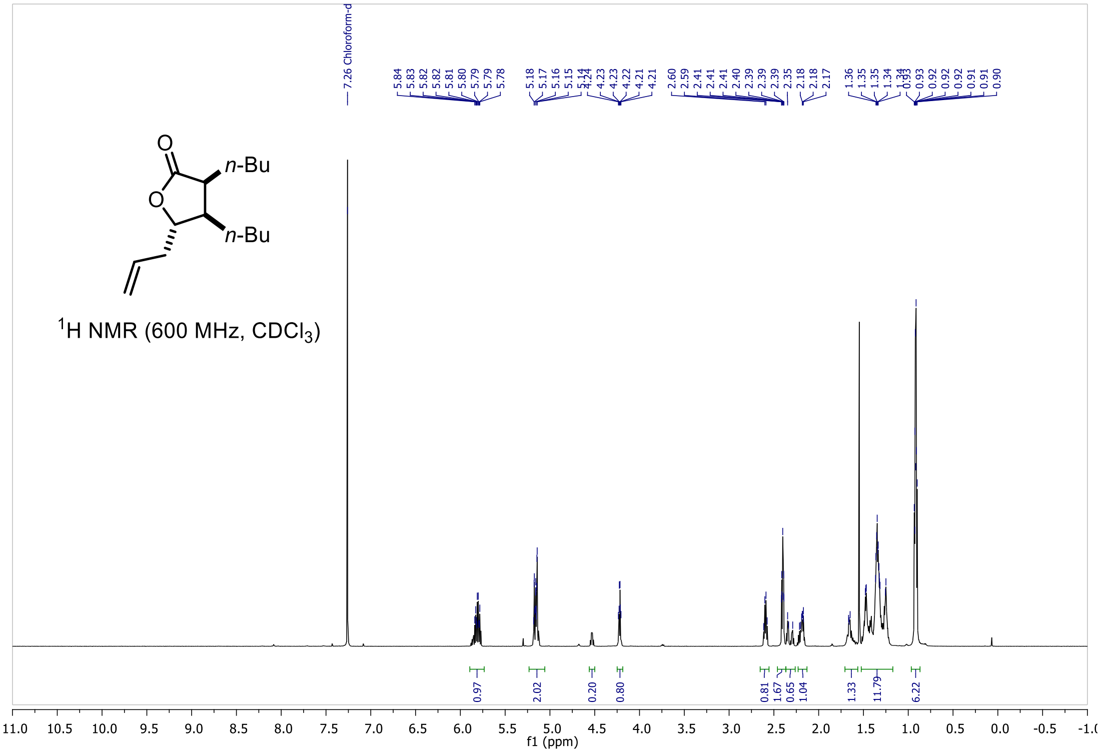

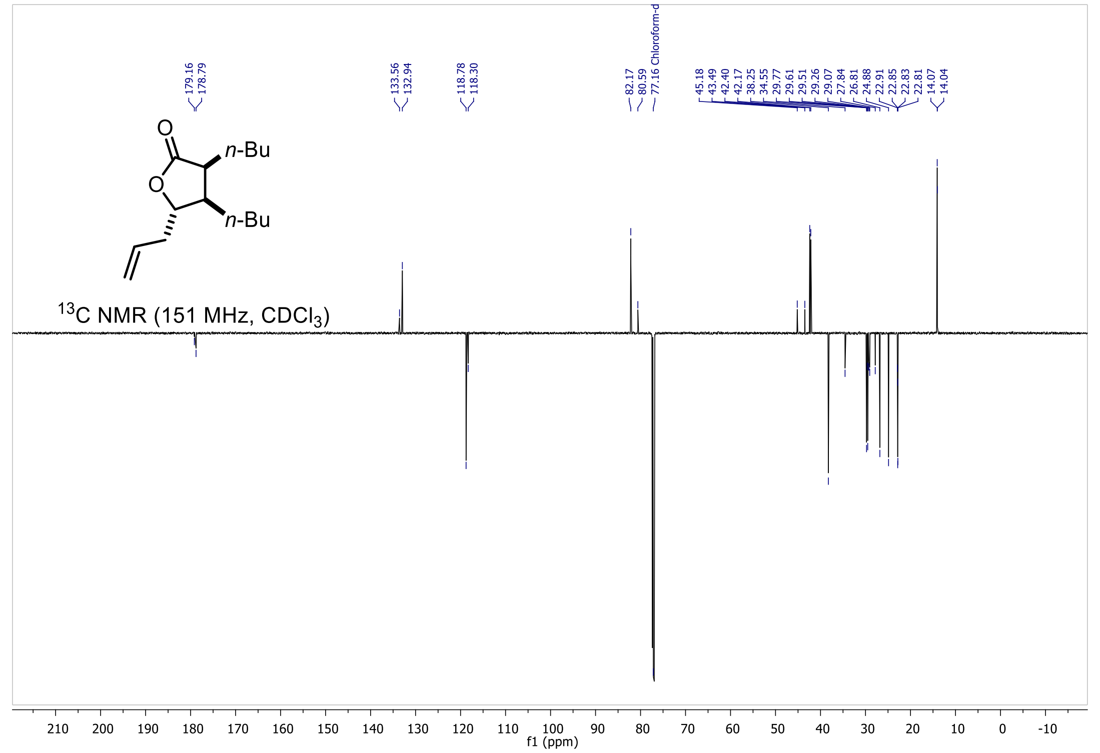

(±)-(3*S*,4*S*,5*S*)-4-Isopropyl-5-methoxy-3-(3-phenylpropyl)dihydrofuran-2(3*H*)-one (12a)

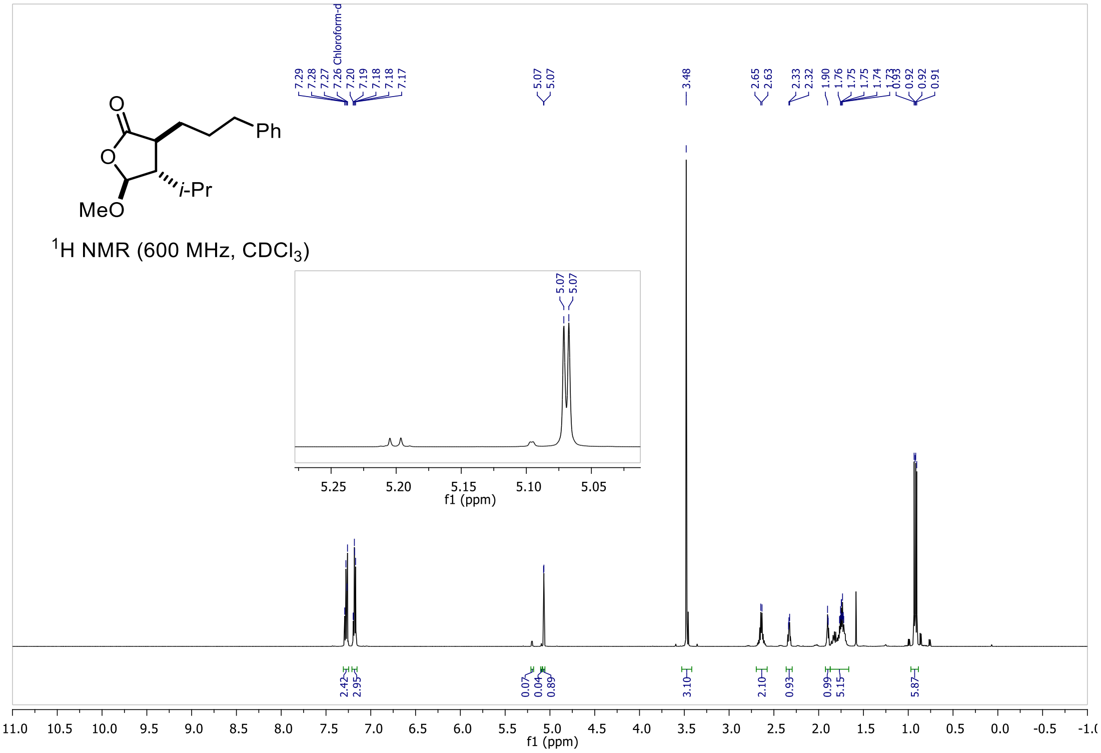

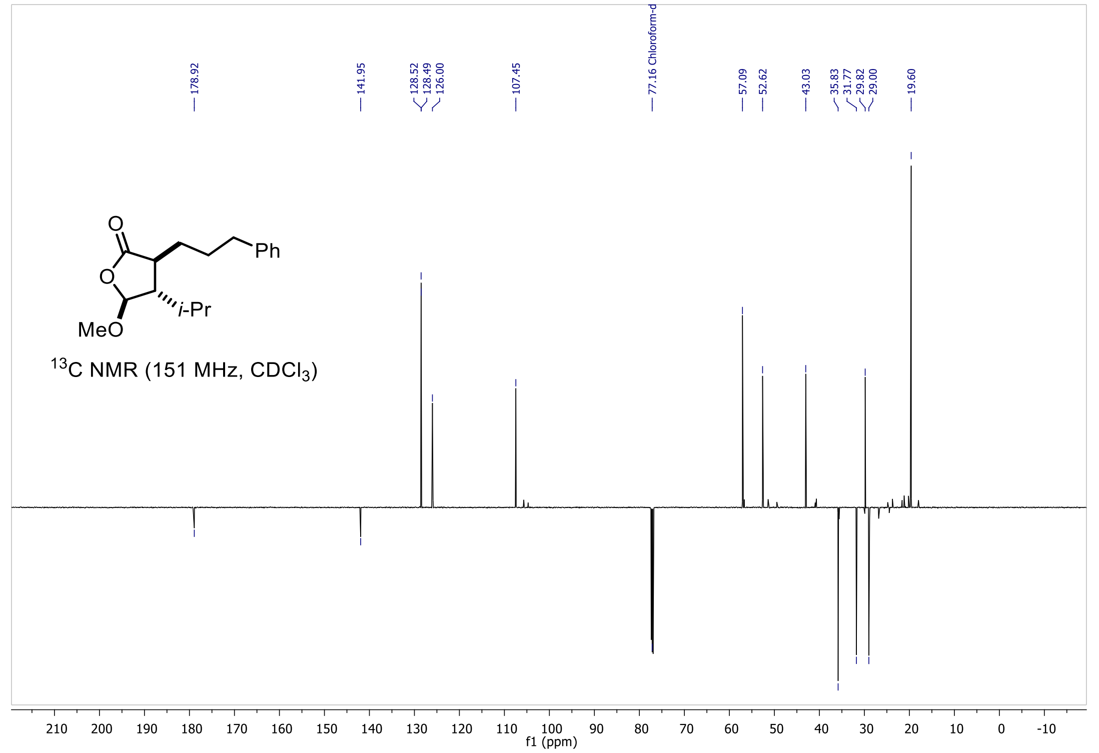

(±)–(3*S*,4*S*,5*S*)-4-Butyl-5-methoxy-3-(3-phenylpropyl)dihydrofuran-2(3*H*)-one (12b)

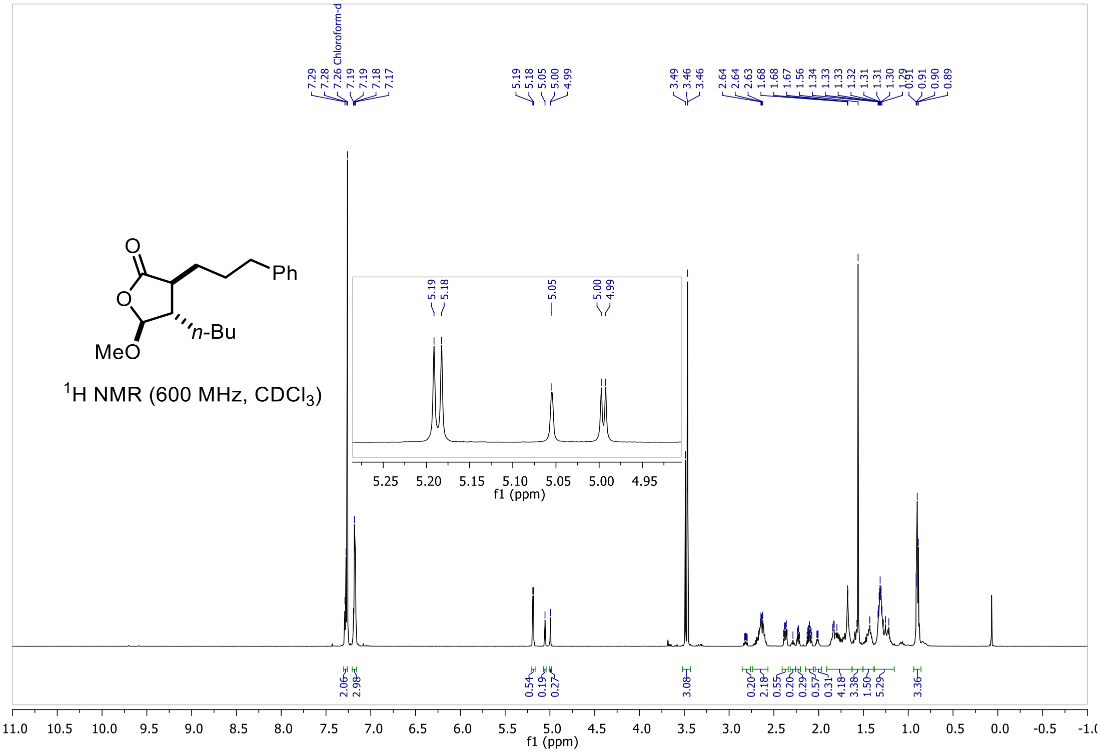

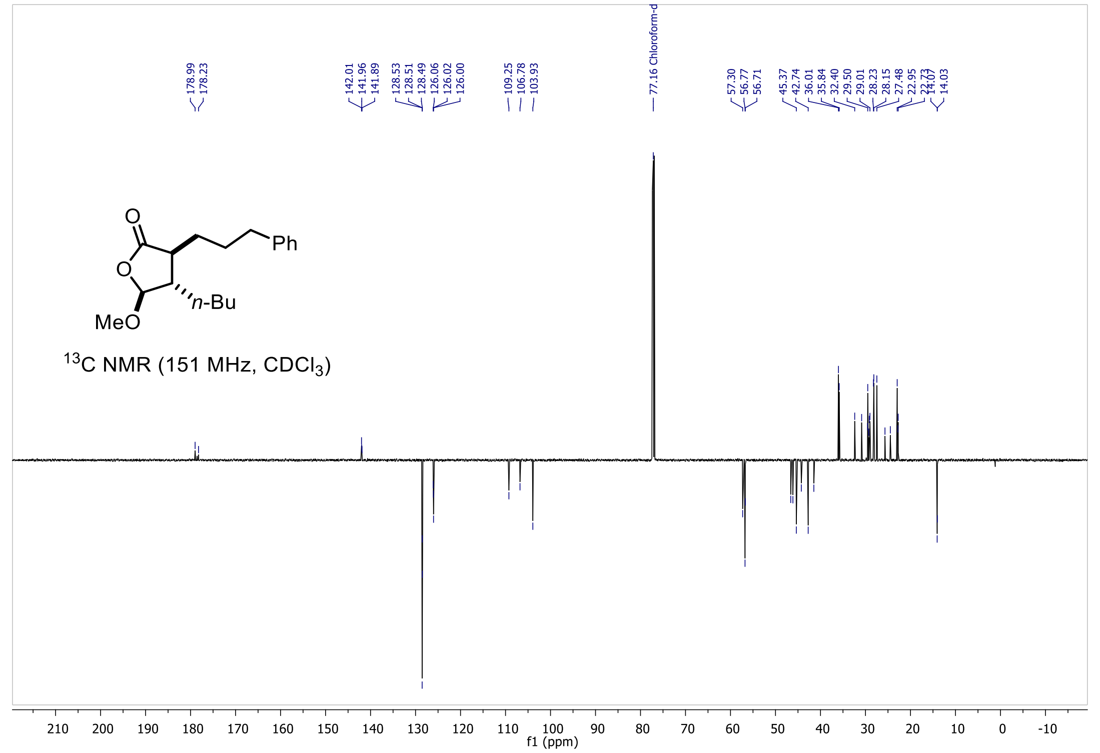

(±)–(3*S*,4*S*,5*S*)-4-Butyl-5-isopropoxy-3-(3-phenylpropyl)dihydrofuran-2(3*H*)-one (12c)

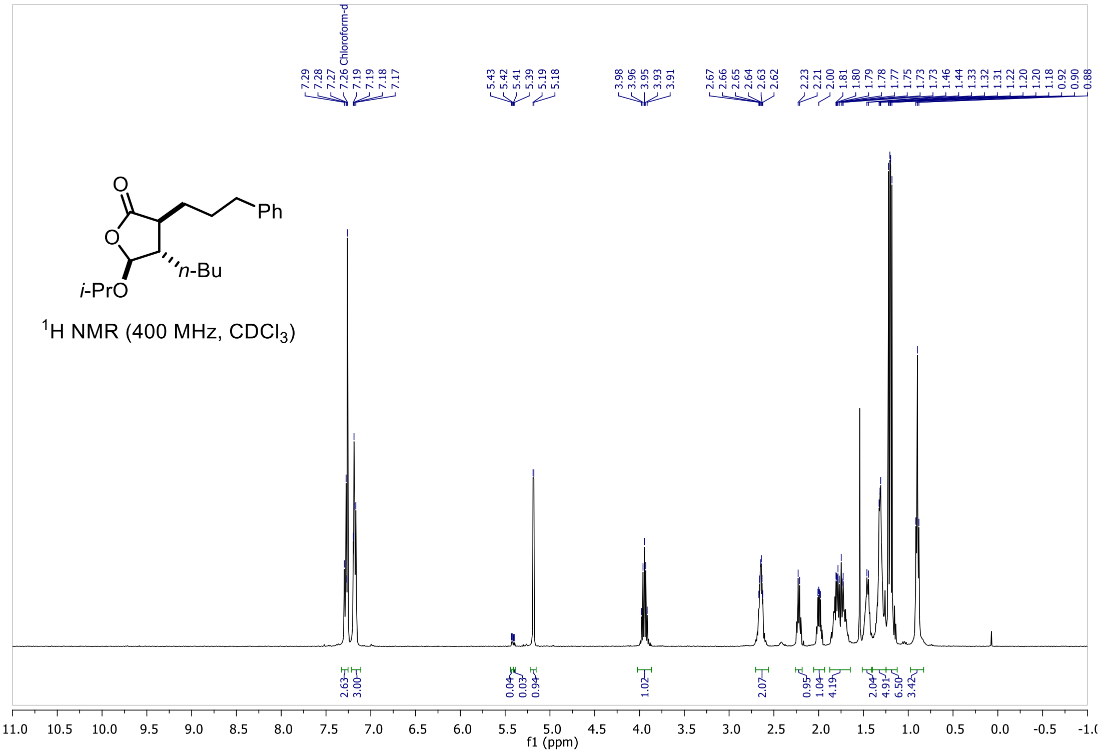

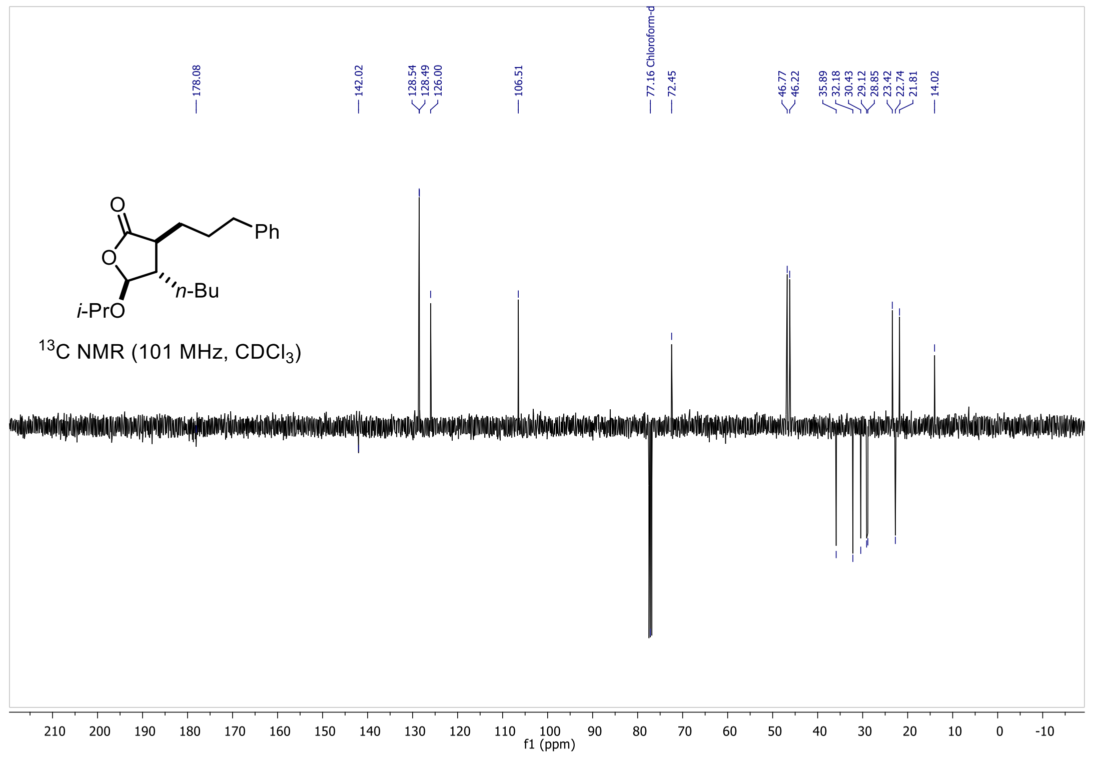

(3*S*,3a*R*,6a*S*)-3-(3-Phenylpropyl)tetrahydrofuro[2,3-b]furan-2(3*H*)-one (13)

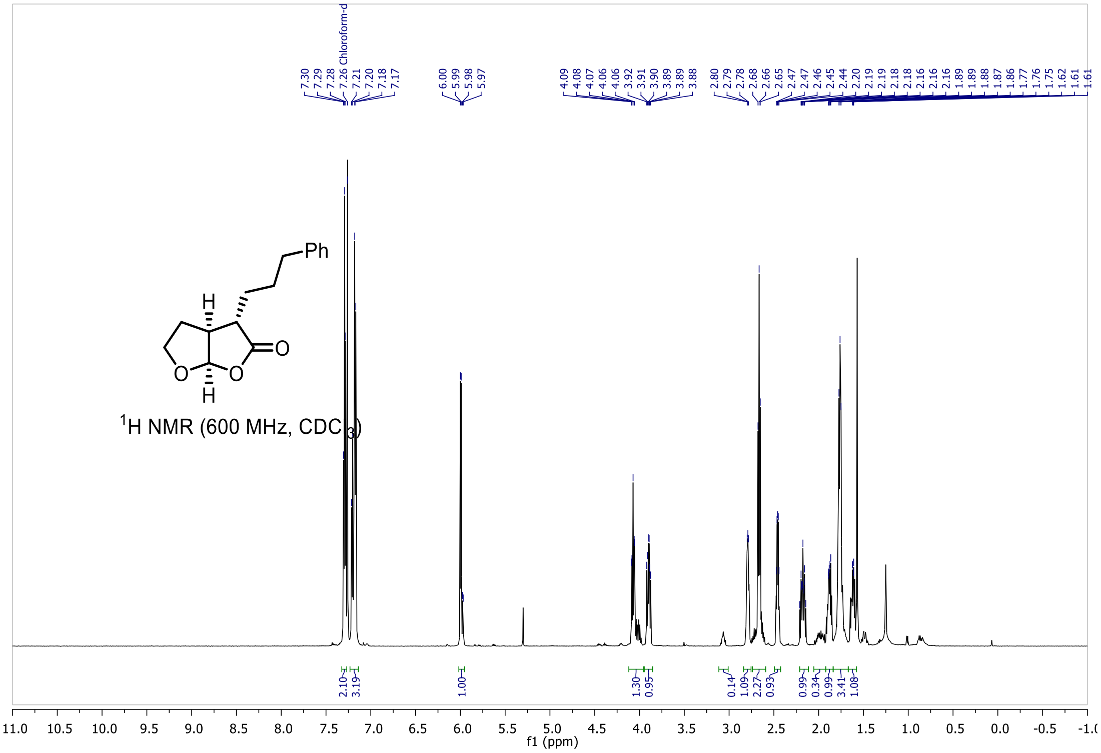

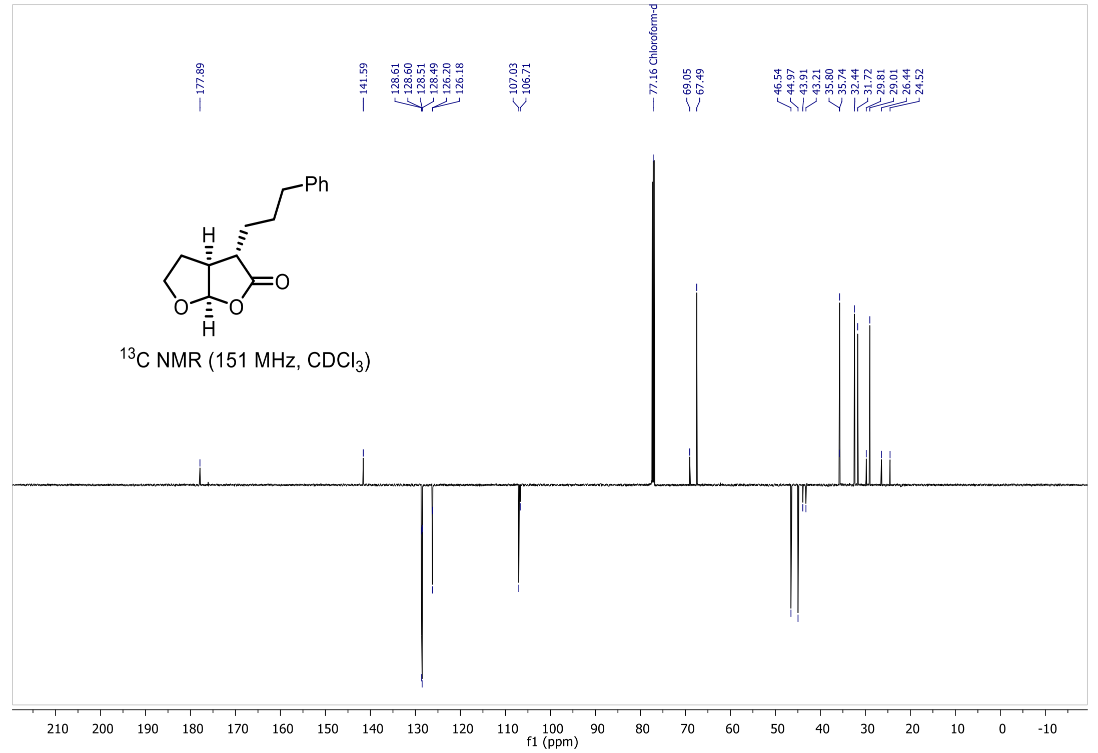

(±)–(3*S*,4*S*)-1-Benzyl-4-isopropyl-3-(3-phenylpropyl)pyrrolidin-2-one (14a)

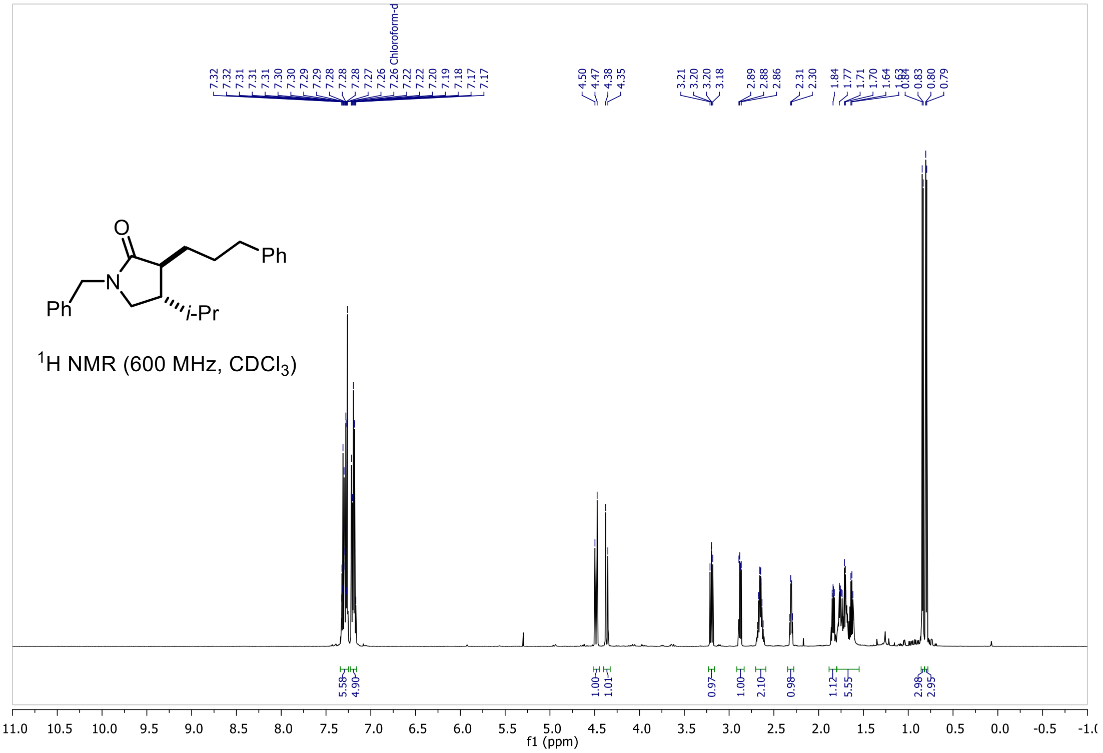

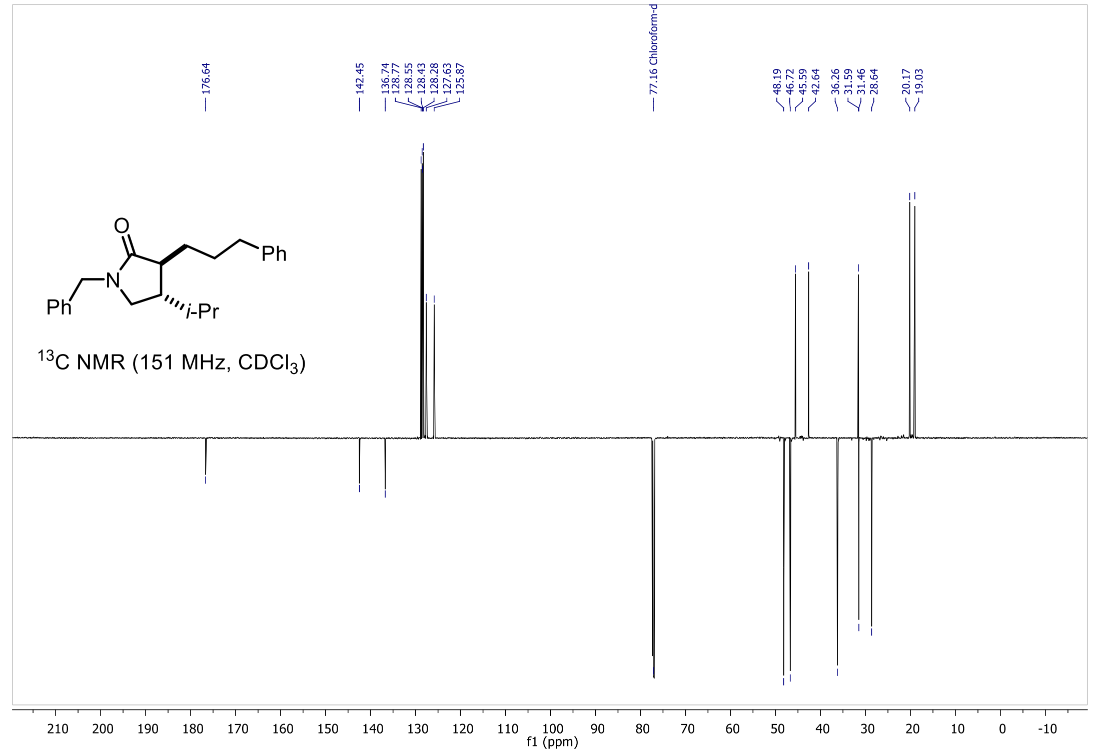

(±)–(3*S*,4*S*)-1-Allyl-4-butyl-3-(3-phenylpropyl)pyrrolidin-2-one (14b)

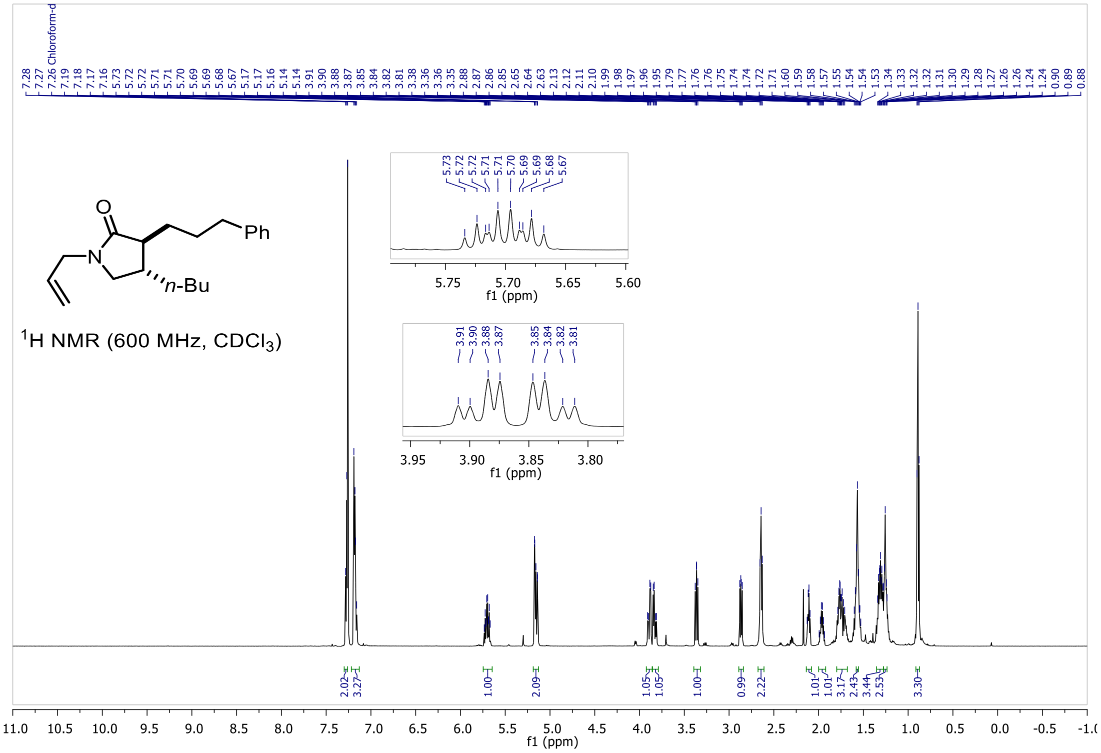

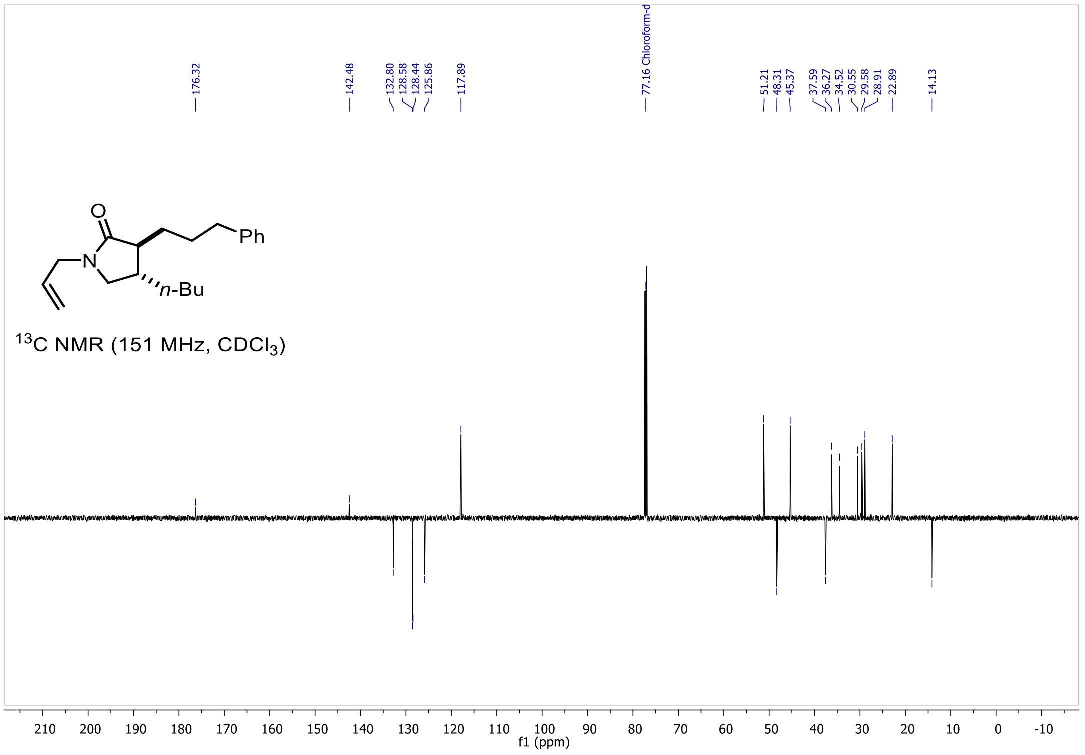

(±)–(3*S*,4*S*)-4-Butyl-1-(4-methoxybenzyl)-3-(3-phenylpropyl)pyrrolidin-2-one (14c)

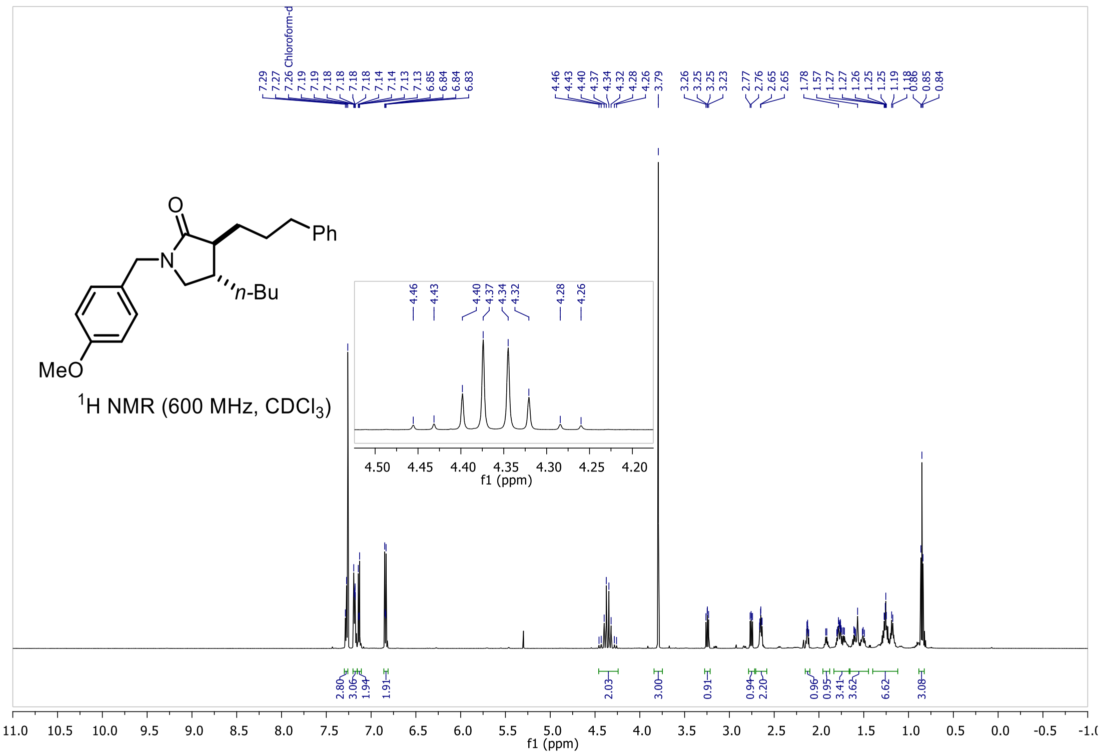

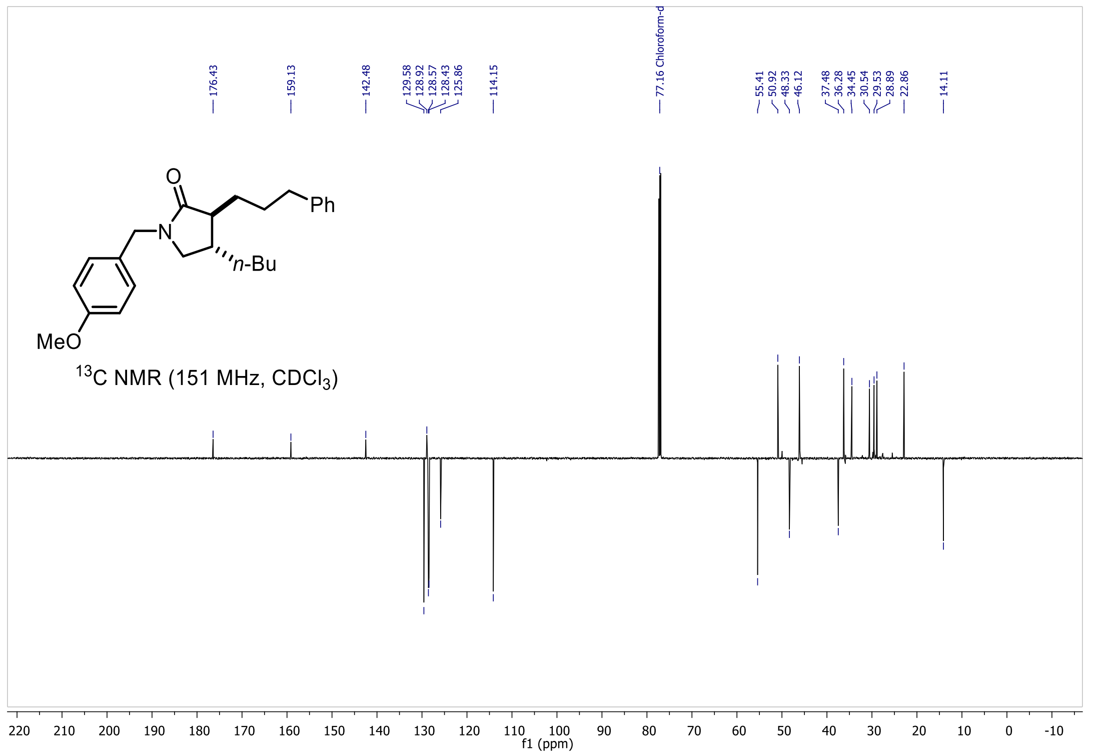

(±)-(3*S*,4*S*)-1-Benzyl-4-butyl-4-methyl-3-(3-phenylpropyl)pyrrolidin-2-one (14d)

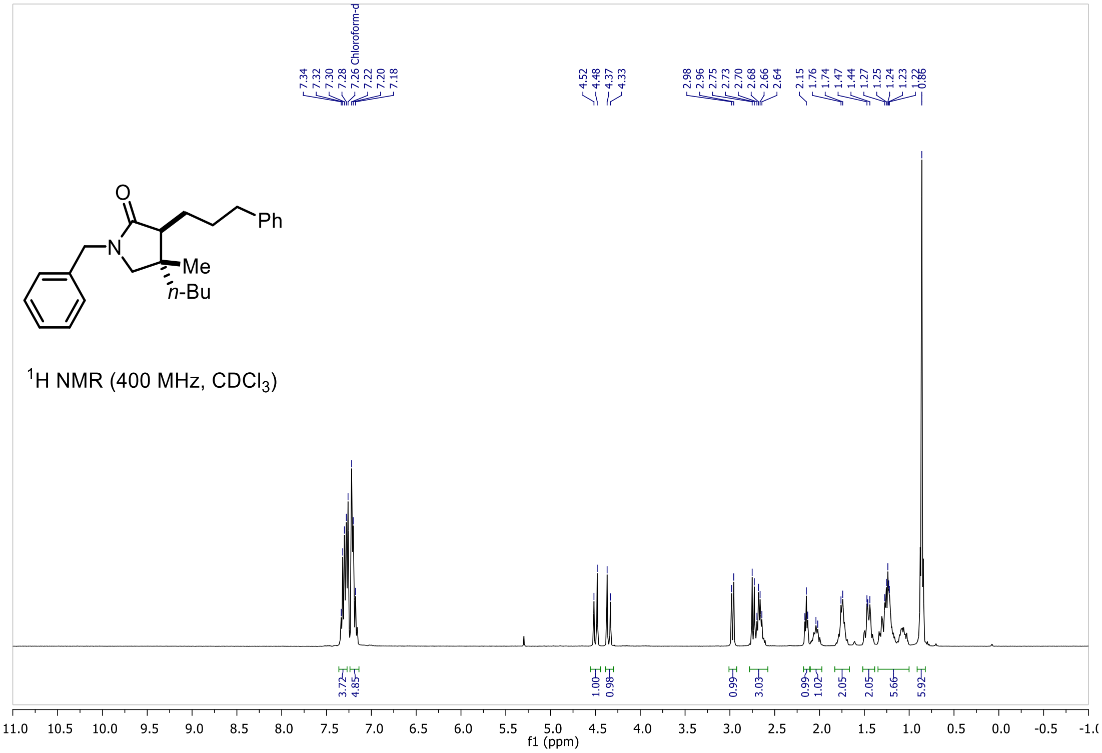

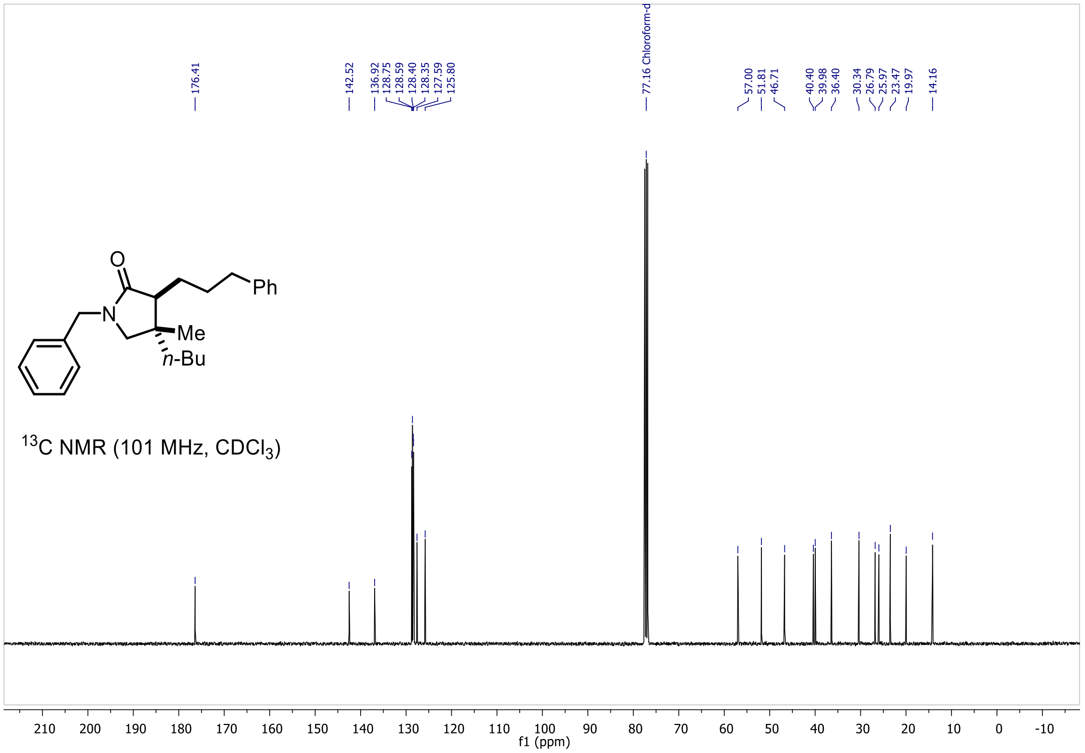

(±)-(3*S*,4*R*)-1-Benzyl-4-butyl-4-methyl-3-(3-phenylpropyl)pyrrolidin-2-one (14e)

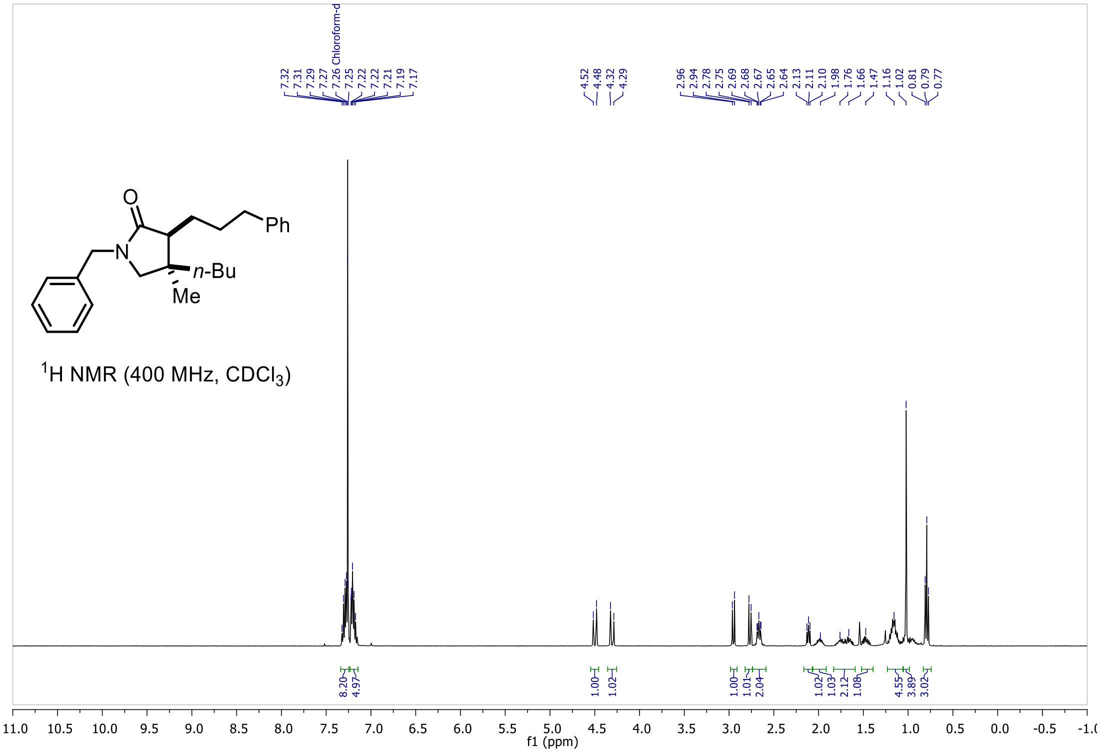

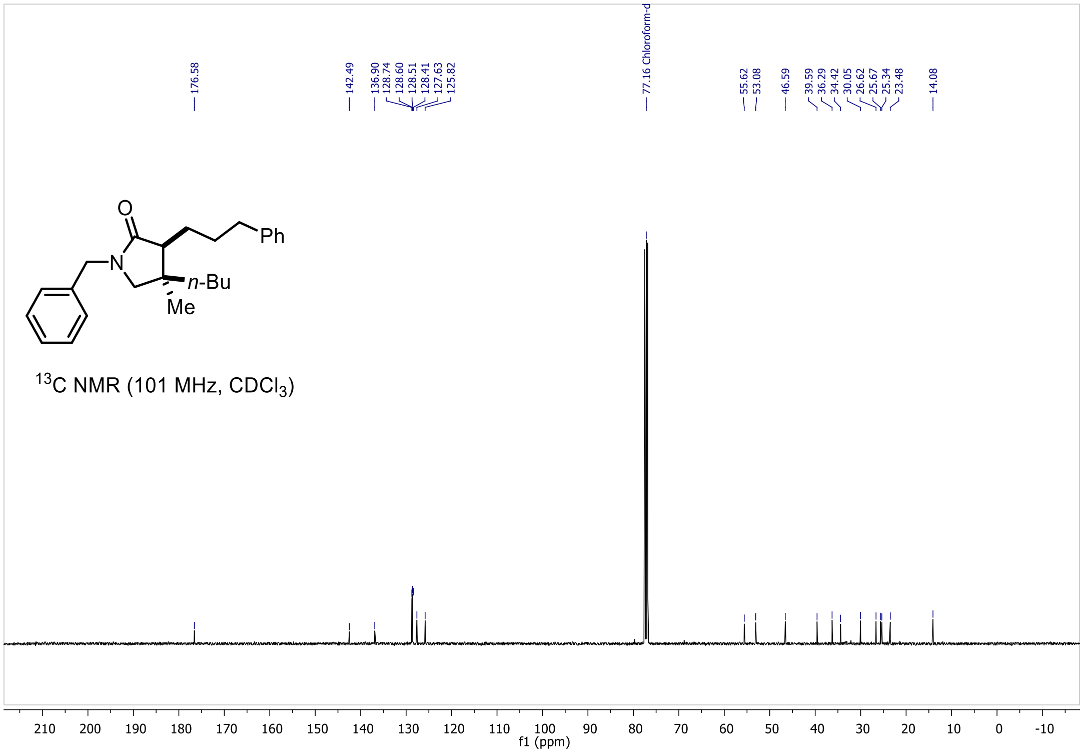

(±)-(3*S*,4*R*)-1-Benzyl-4-butyl-3-(3-phenylpropyl)pyrrolidin-2-one (14f)

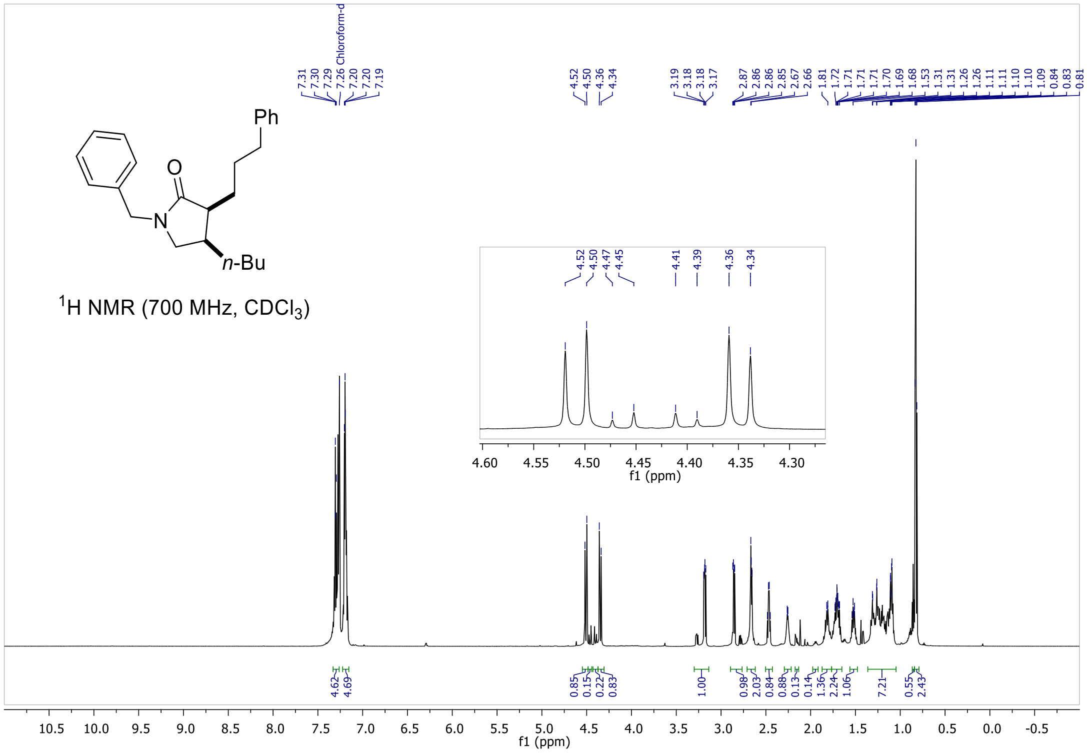

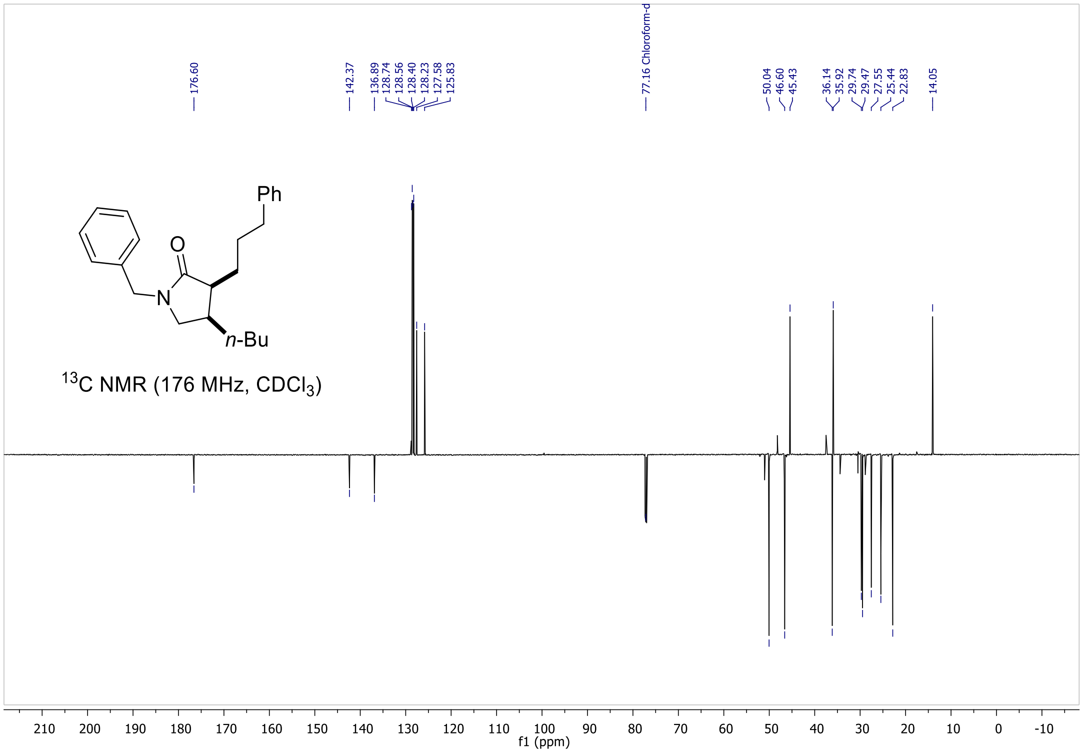

(±)-(3*S*,4*R*)-4-Butyl-1-(4-methoxybenzyl)-3-(3-phenylpropyl)pyrrolidin-2-one (14g)

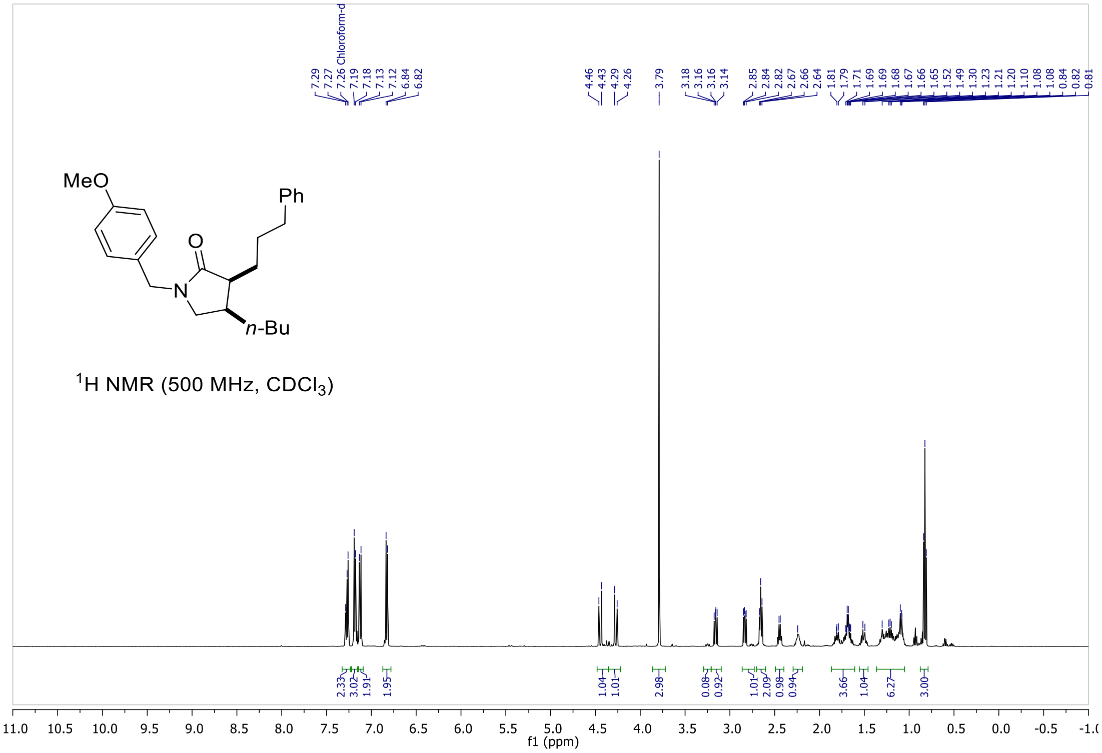

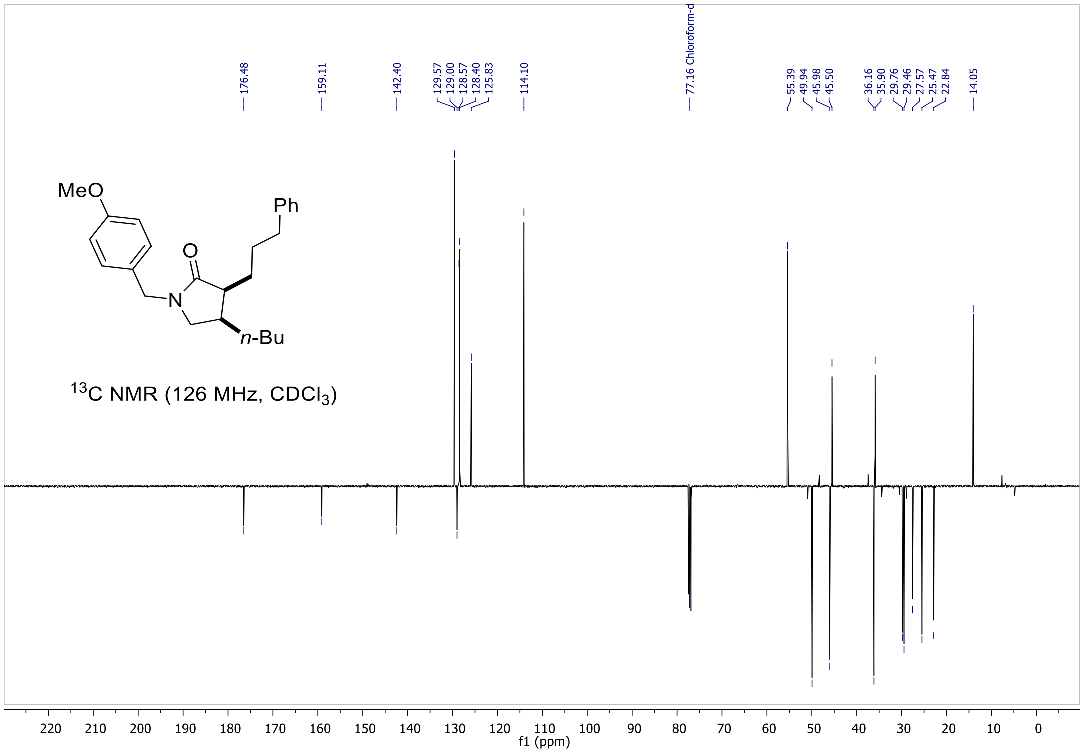

(±)-(3*S*,4*R*)-1-Allyl-4-butyl-3-(3-phenylpropyl)pyrrolidin-2-one (14h)

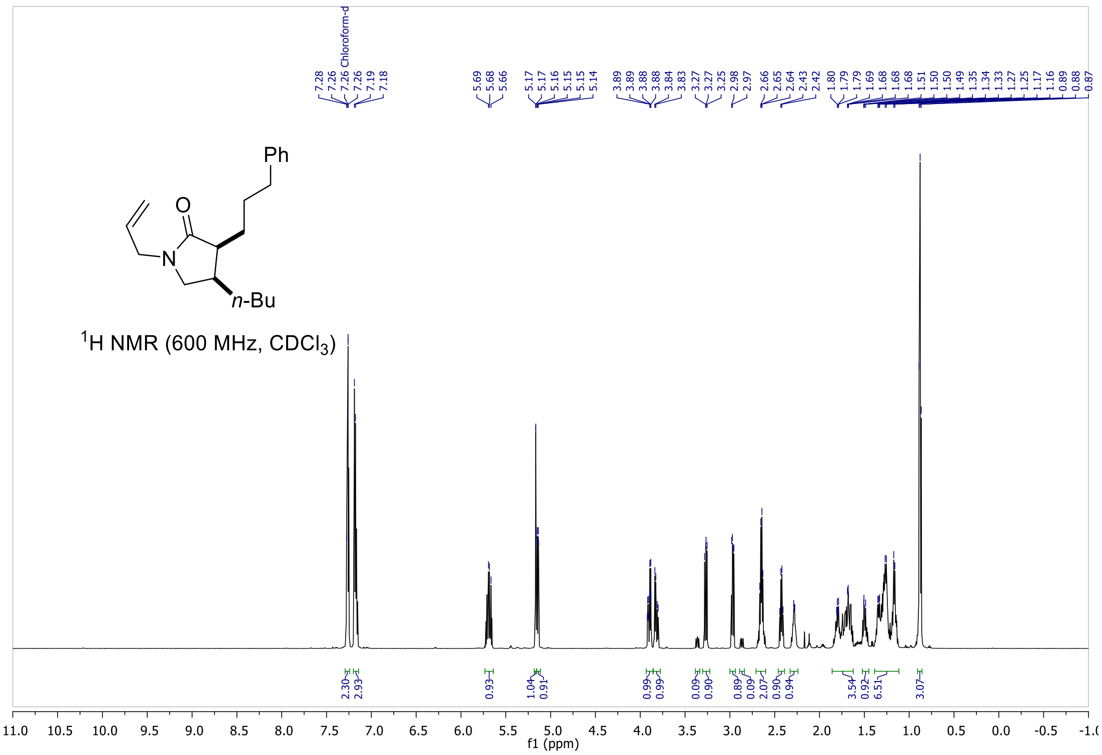

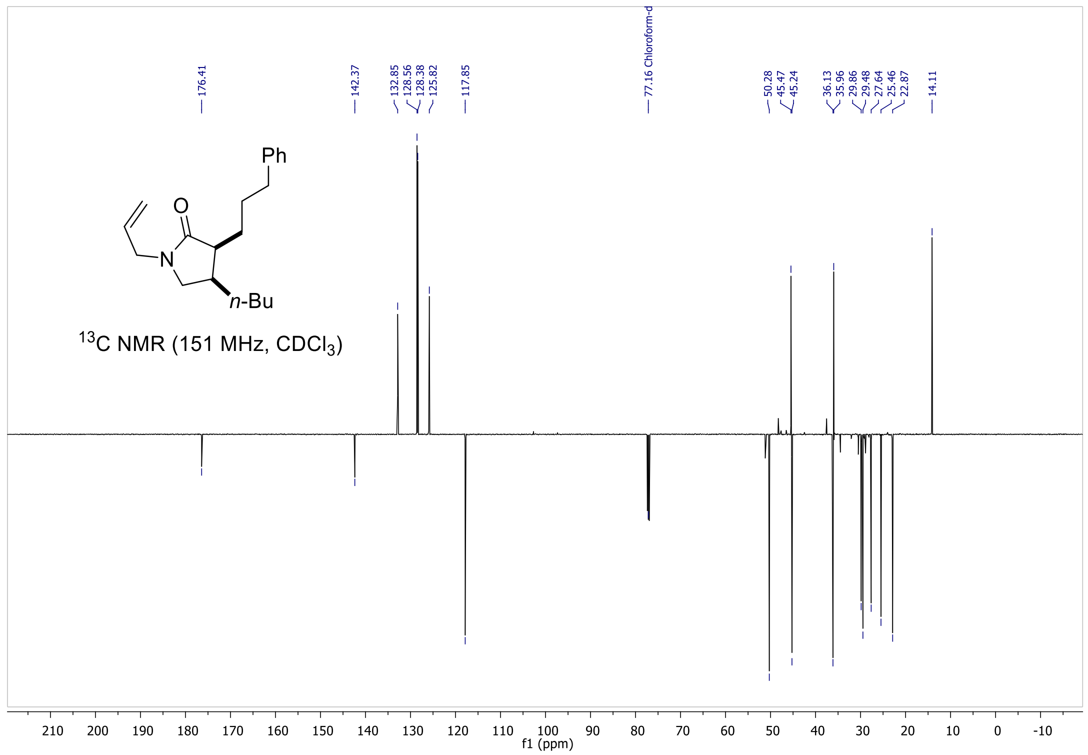

(±)-(3*S*,4*S*)-4-Isopropyl-1-phenyl-3-(3-phenylpropyl)pyrrolidin-2-one (14i)

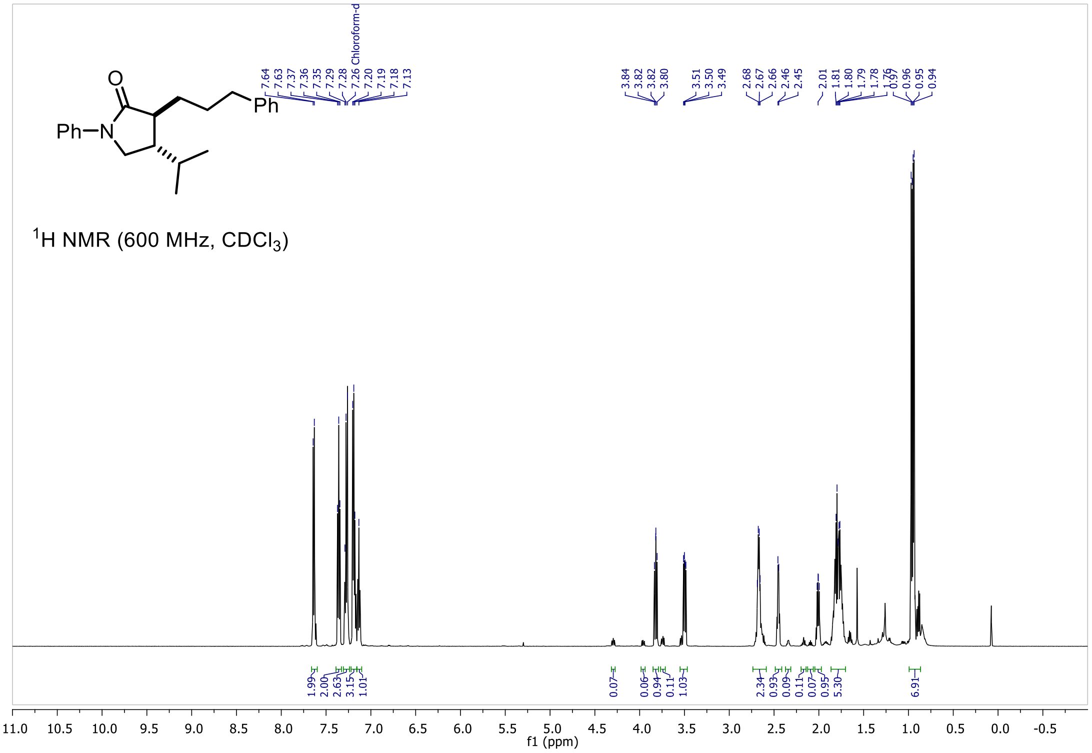

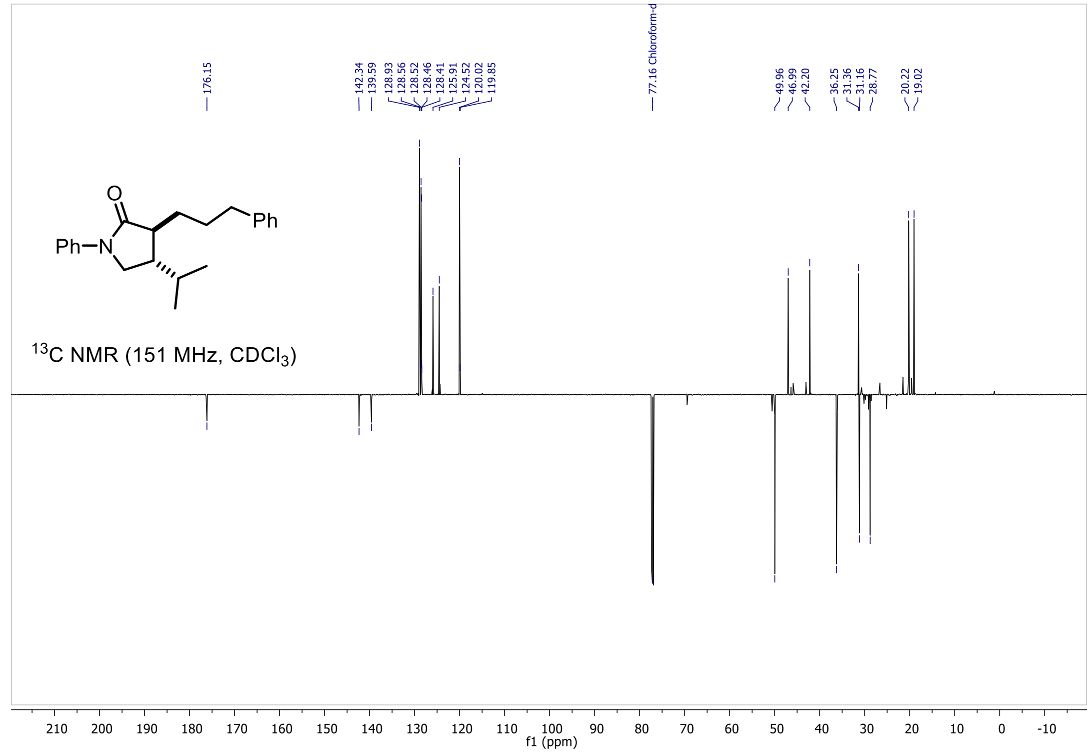

(±)-(3*S*,4*S*)-4-butyl-5-hydroxy-1-(4-methoxybenzyl)-4-methyl-3-(3-phenylpropyl)pyrrolidin-2-one (15)

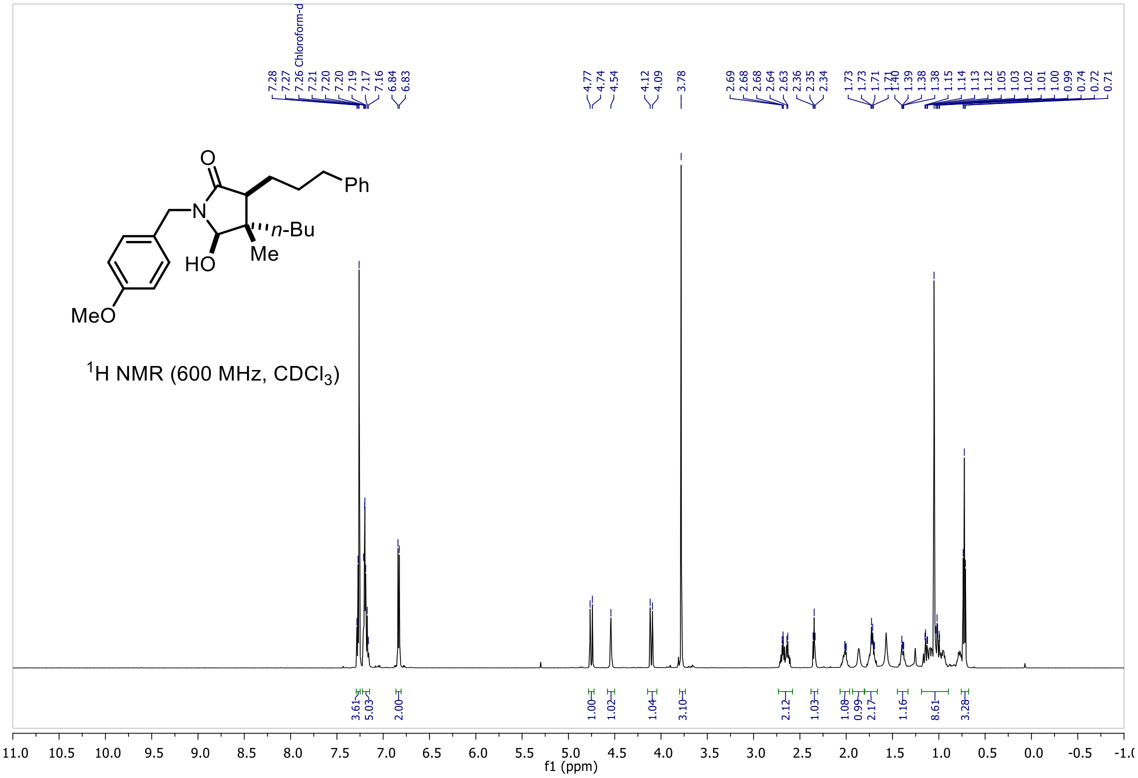

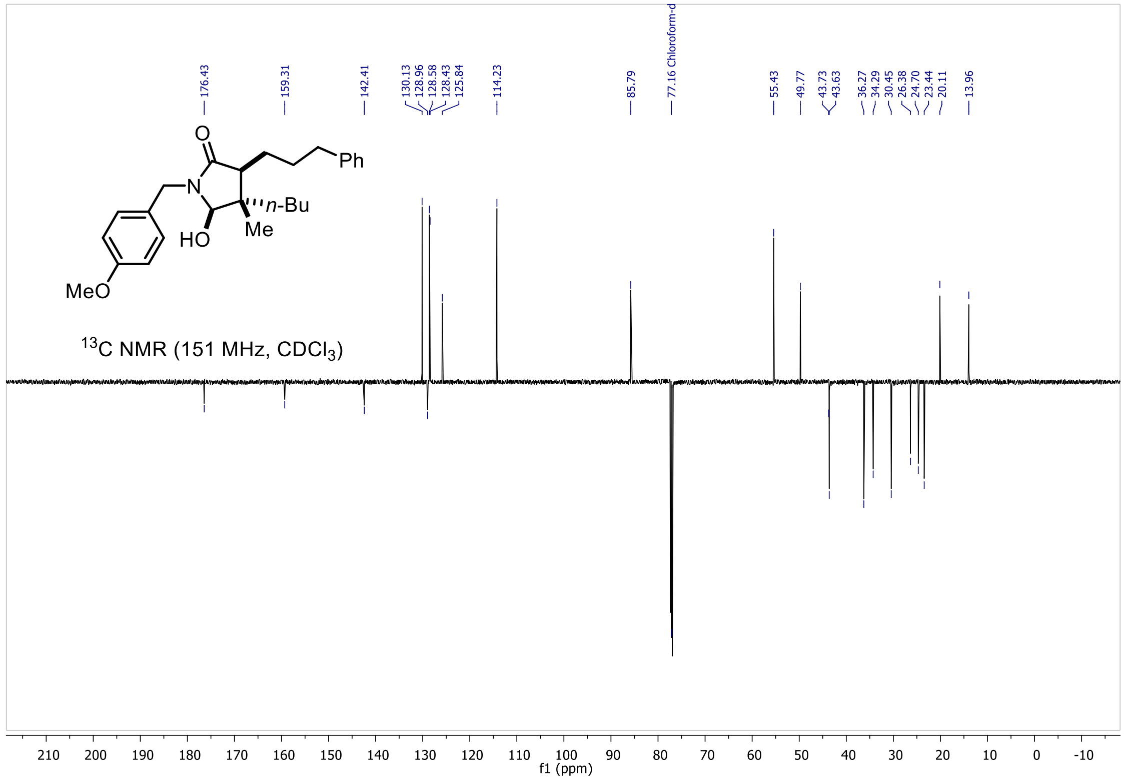

4-Butyl-1-(4-methoxybenzyl)-3-(3-phenylpropyl)-1,5-dihydro-2*H*-pyrrol-2-one (16)

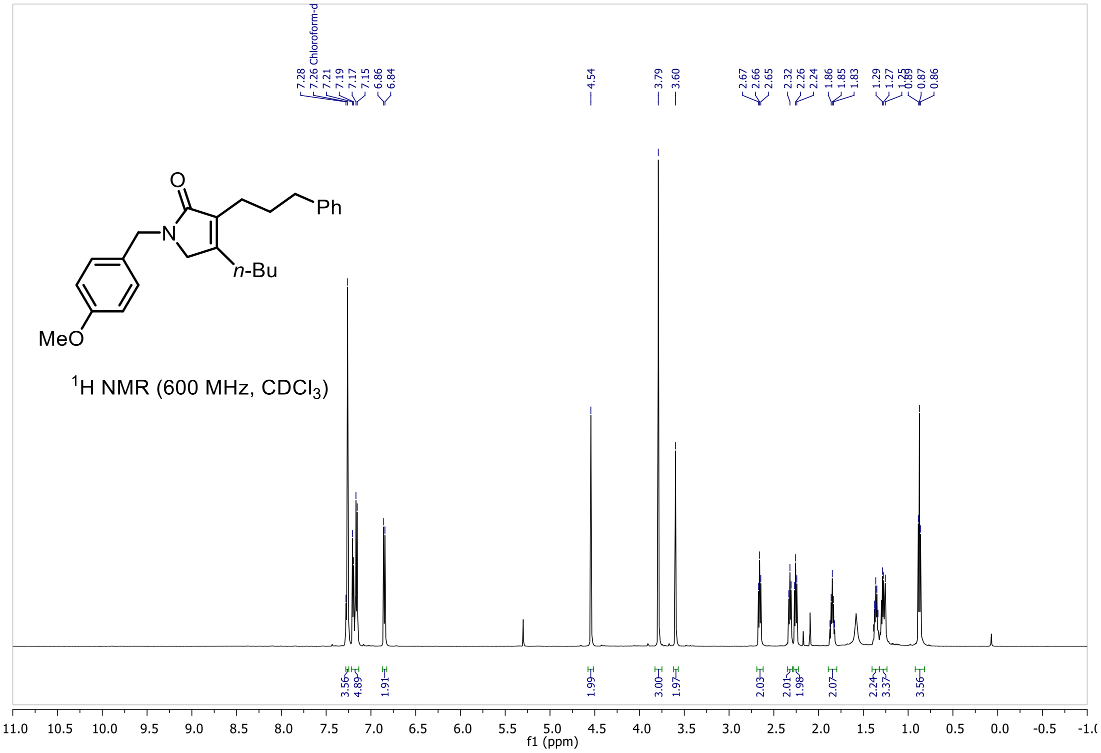

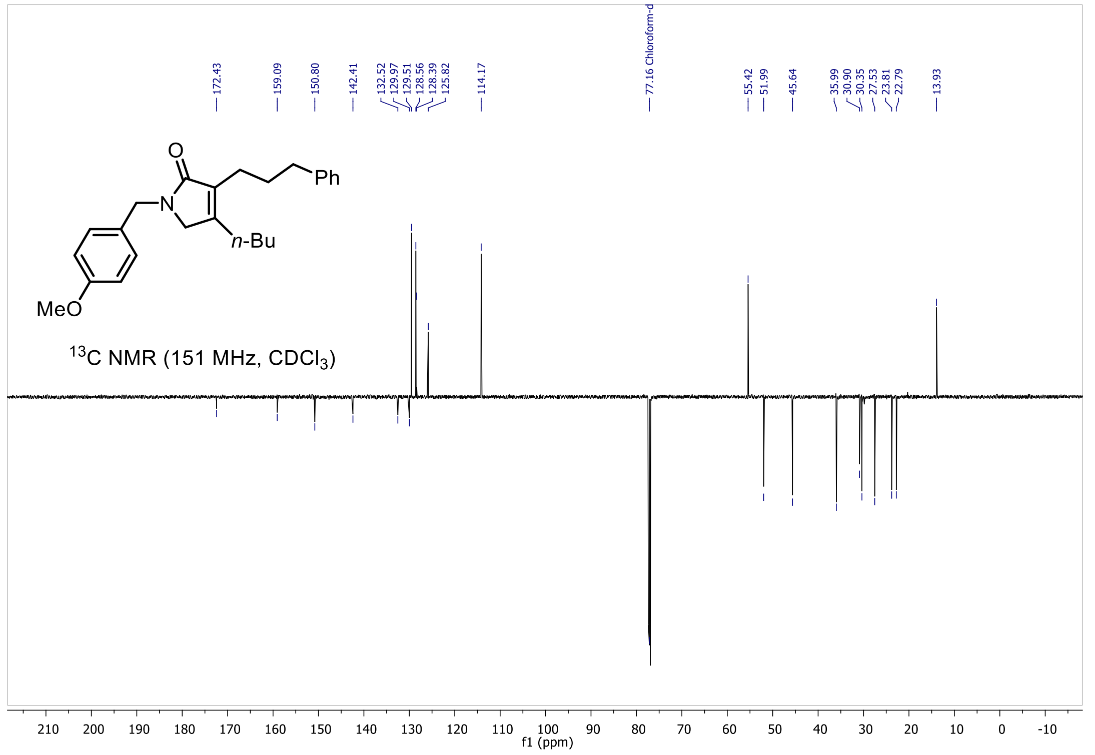

(±)-(1*R*,2*R*,11*bR*)-1-Butyl-1-methyl-2-(3-phenylpropyl)-1,2,5,6,11,11*b*-hexahydro-3*H*-indolizino[8,7-*b*]indol-3-one (18a)

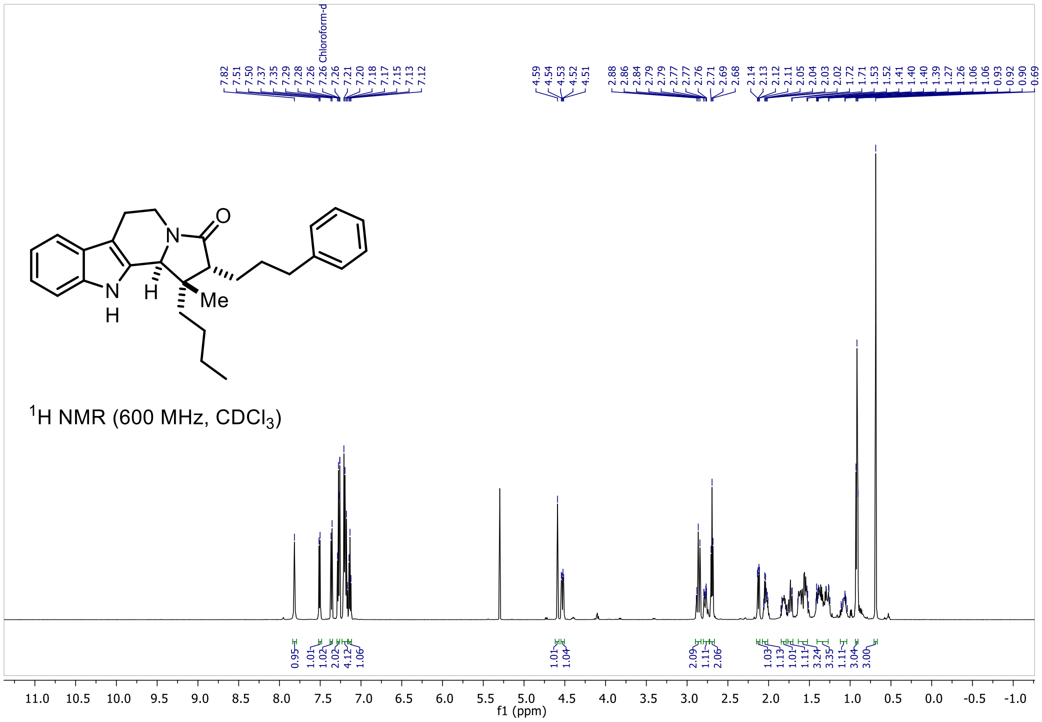

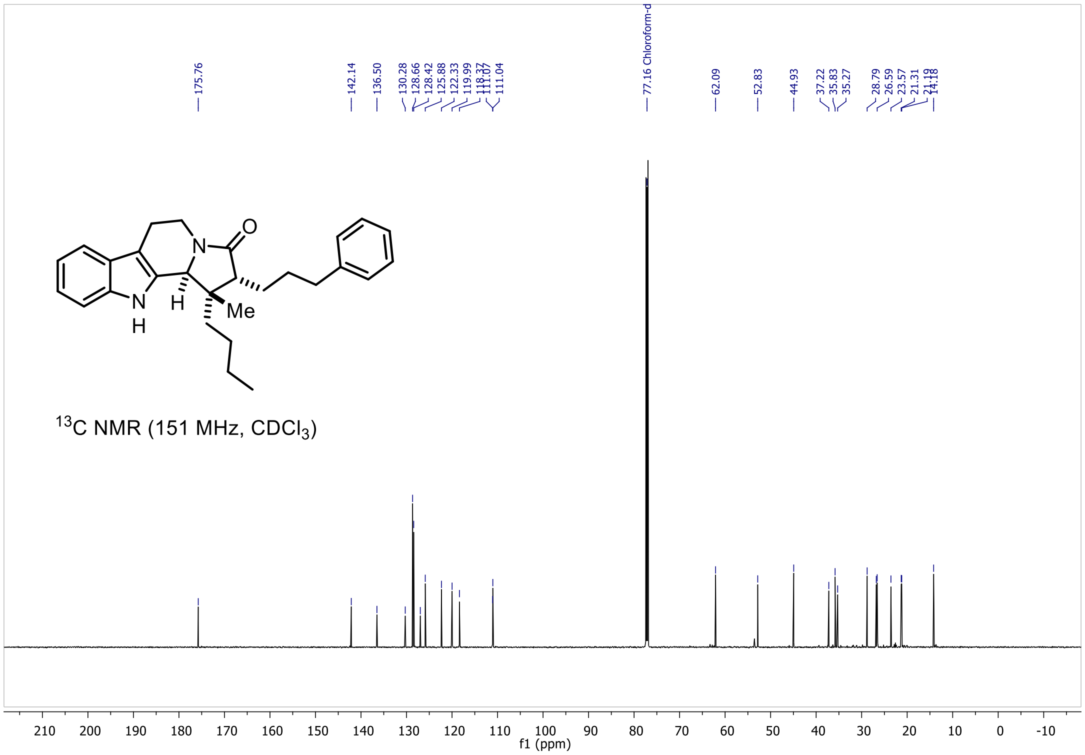

(±)-(1*R*,2*R*,11b*R*)-1-Butyl-8-methoxy-1-methyl-2-(3-phenylpropyl)-1,2,5,6,11,11b-hexahydro-3H-indolizino[8,7-b]indol-3-one (18b)

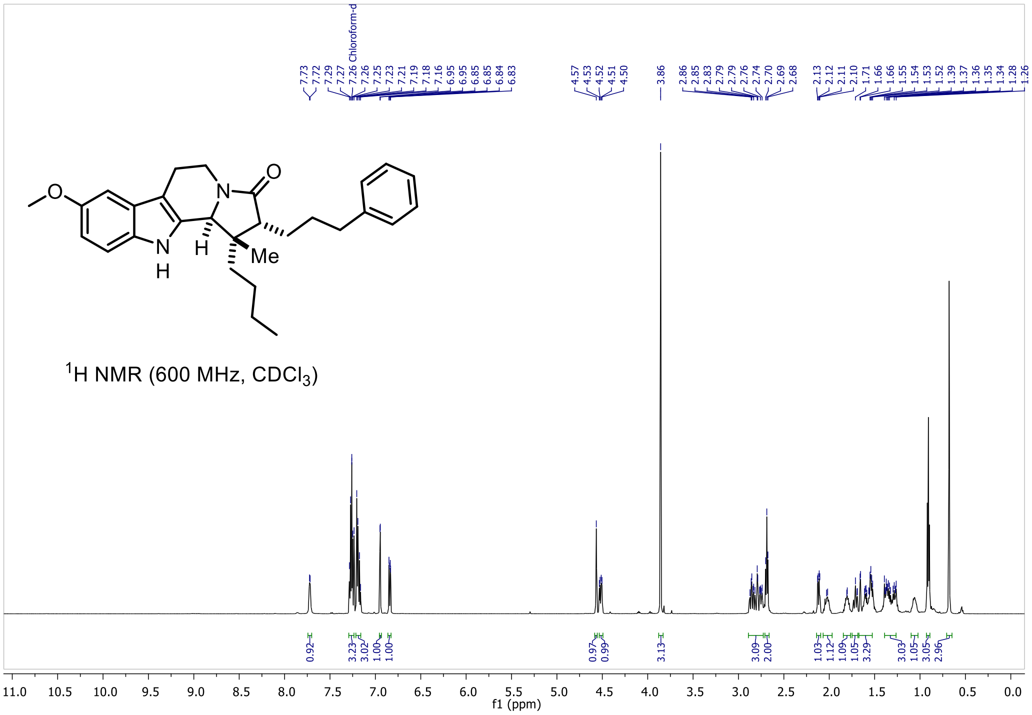

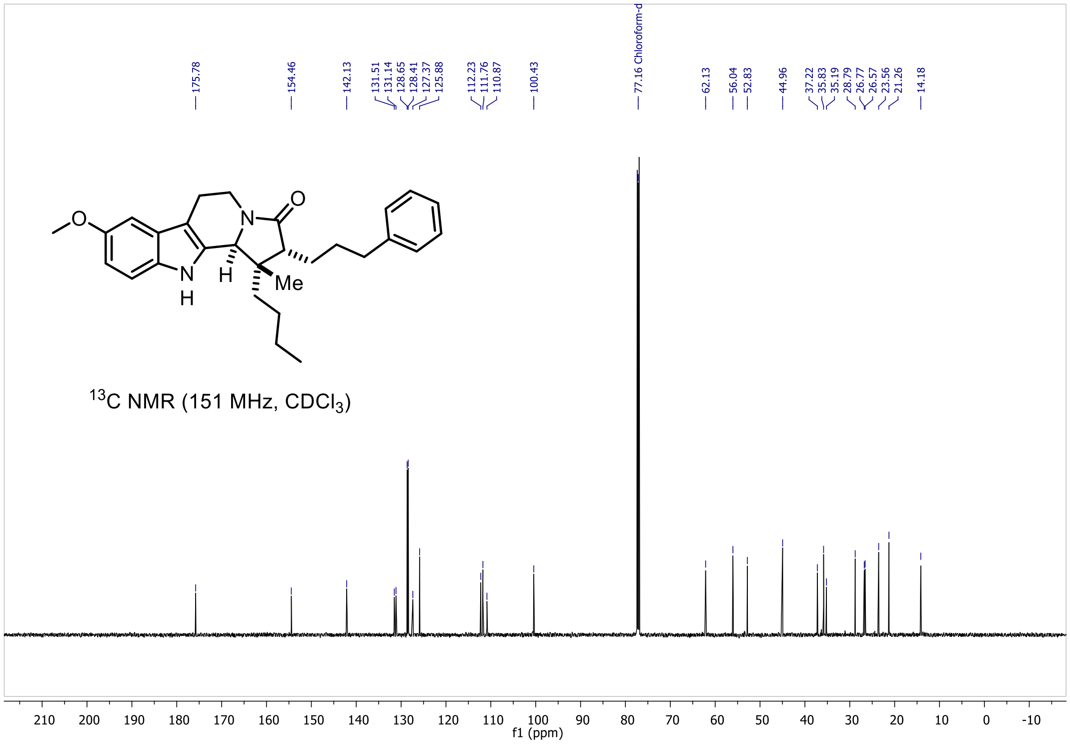

(±)-(1*R*,2*R*,11b*R*)-8-Bromo-1-butyl-1-methyl-2-(3-phenylpropyl)-1,2,5,6,11,11b-hexahydro-3*H*-indolizino[8,7-b]indol-3-one (18c)

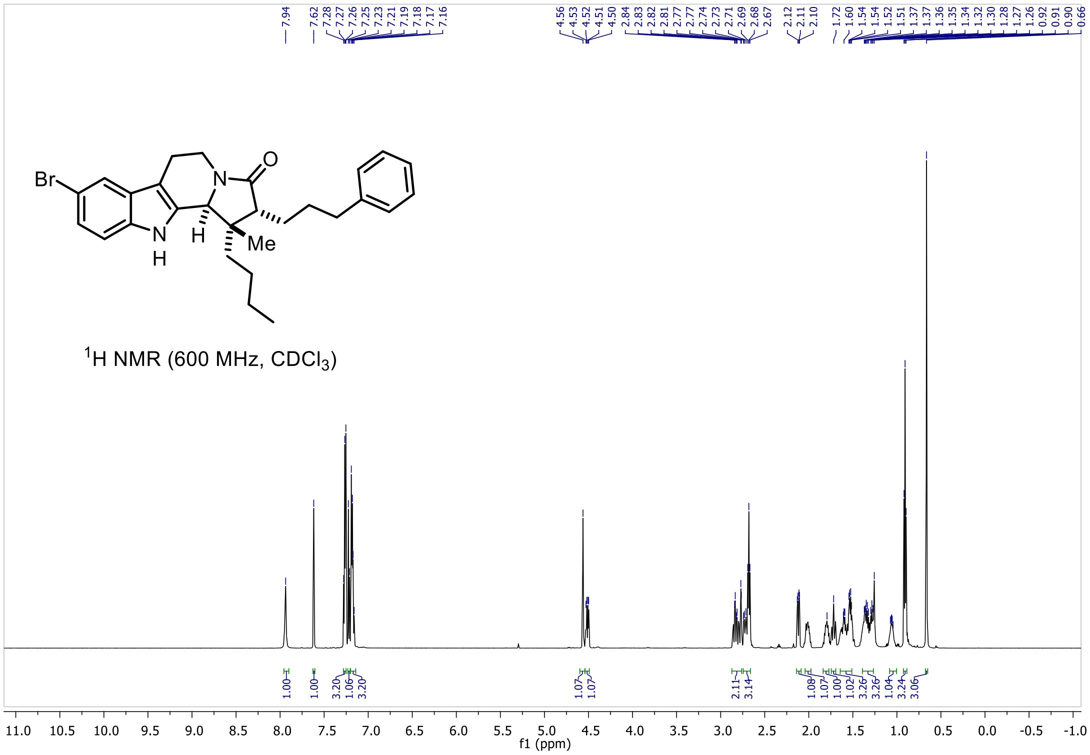

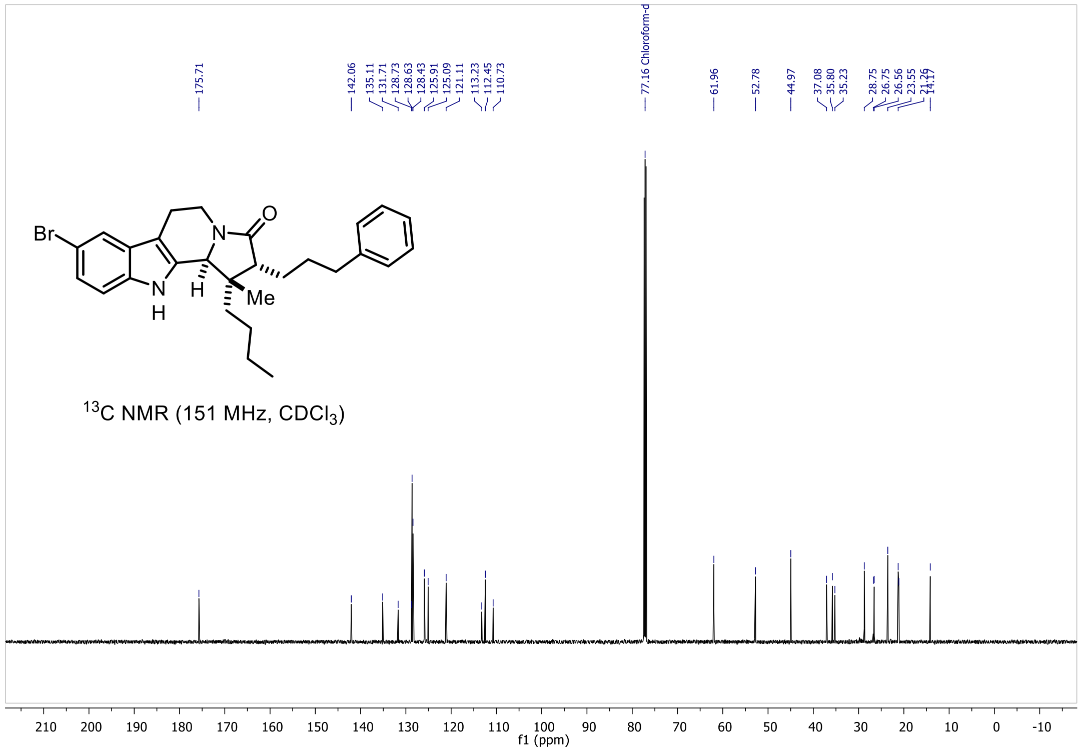

(2*R*,3*S*)-2-(4-Methoxyphenyl)-3-methyl-4-oxo-4-(2-oxooxazolidin-3-yl)butanal (*syn*-19)

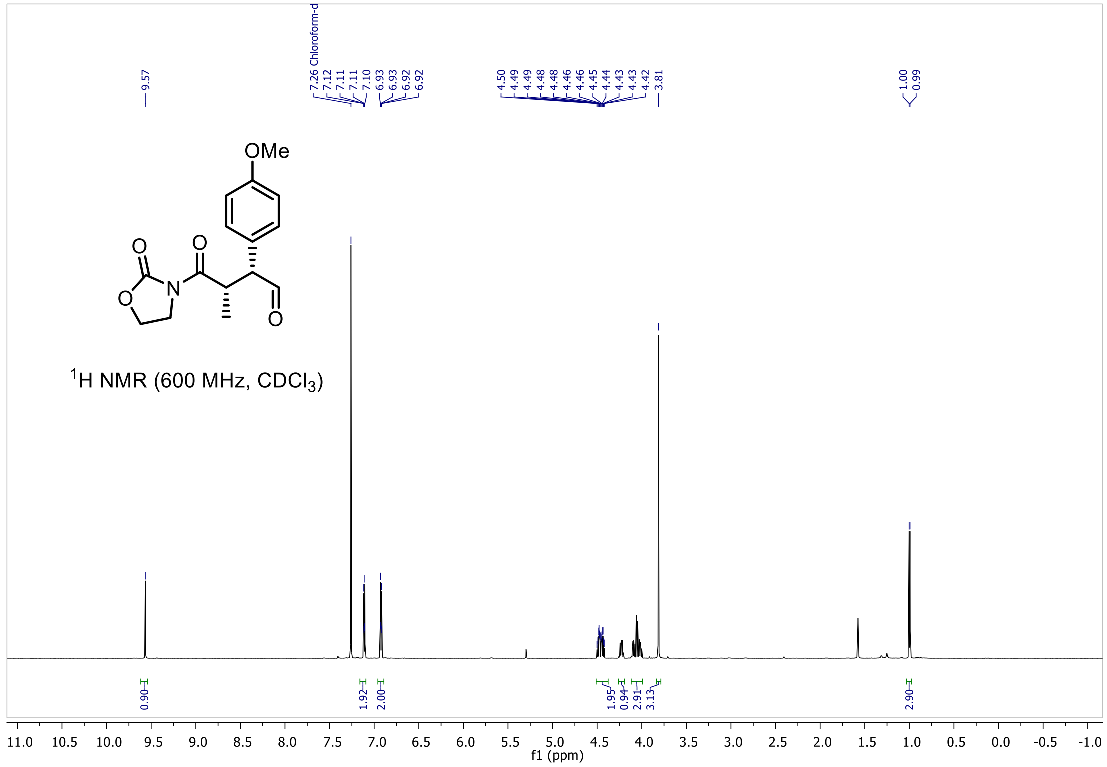

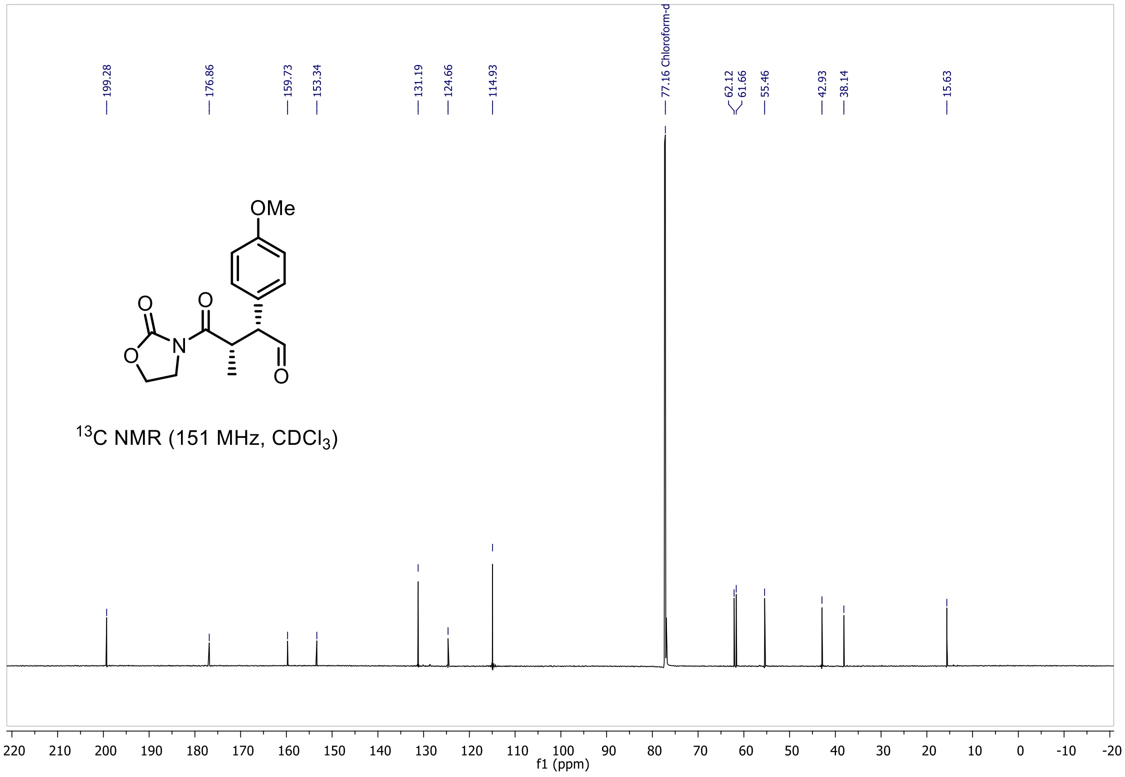

(2*S*,3*S*)-2-(4-Methoxyphenyl)-3-methyl-4-oxo-4-(2-oxooxazolidin-3-yl)butanal (*anti*-19)

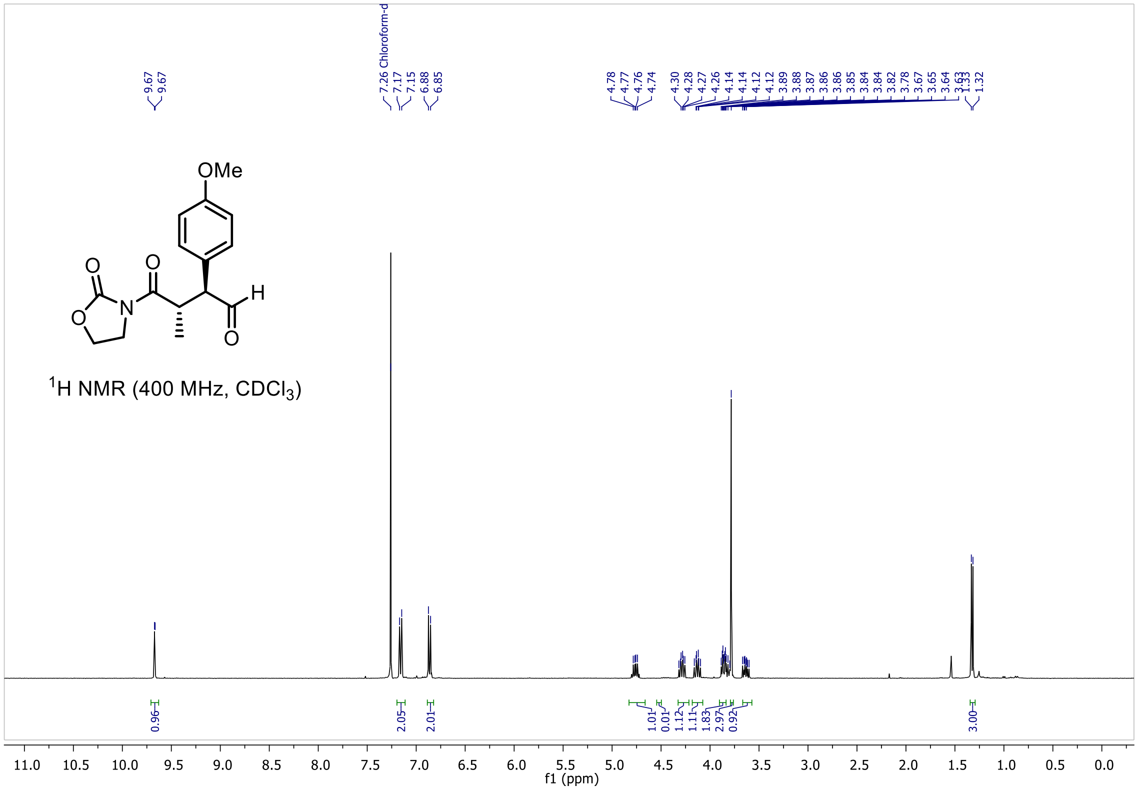

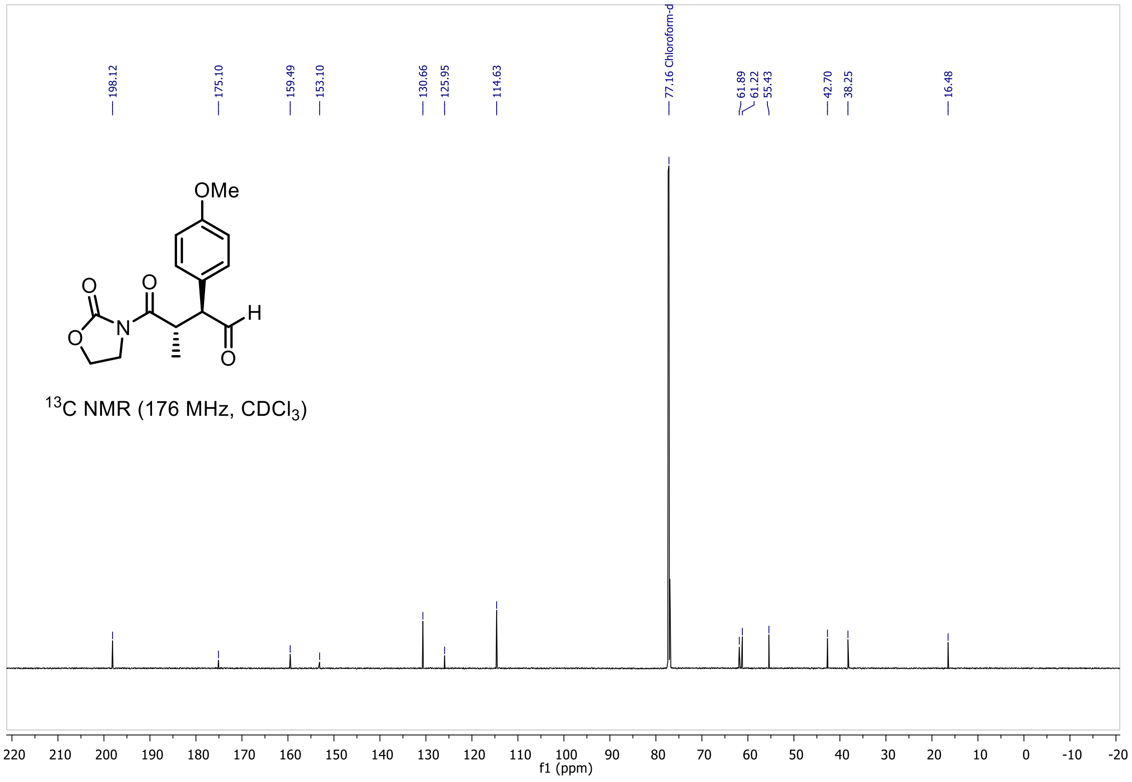

(3*S*,4*R*,5*R*)-4-(4-Methoxyphenyl)-3-methyl-5-undecyldihydrofuran-2(3*H*)-one (20a)

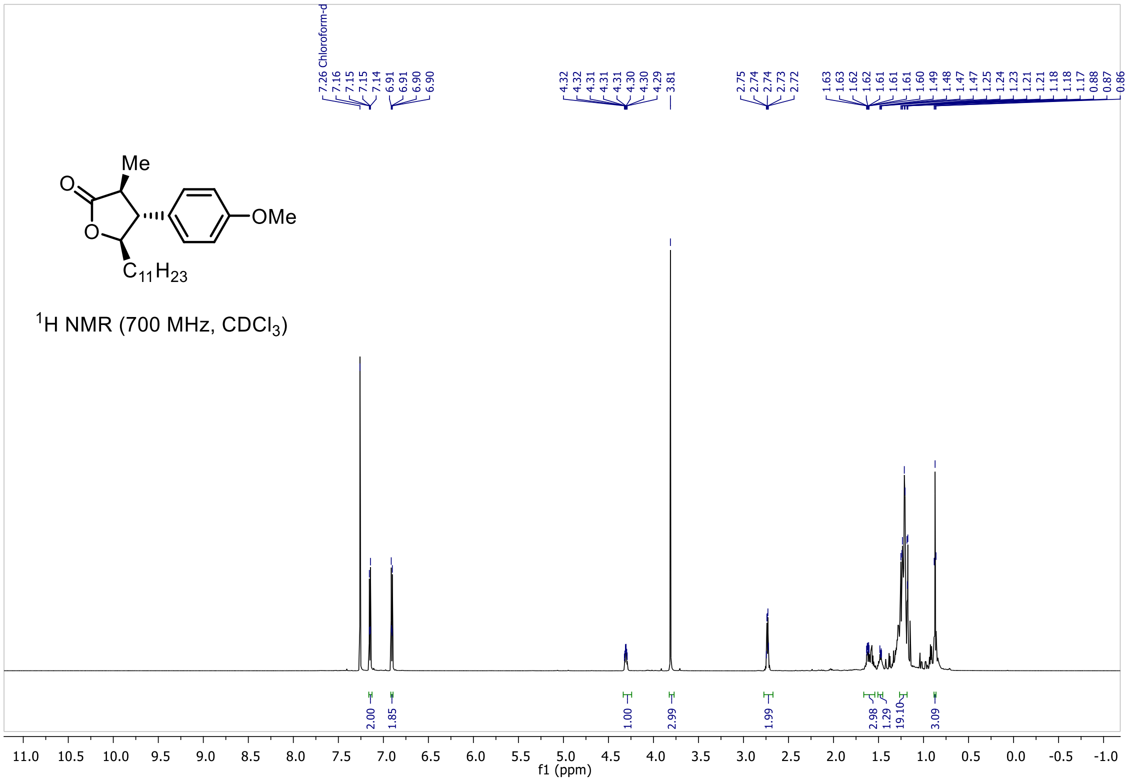

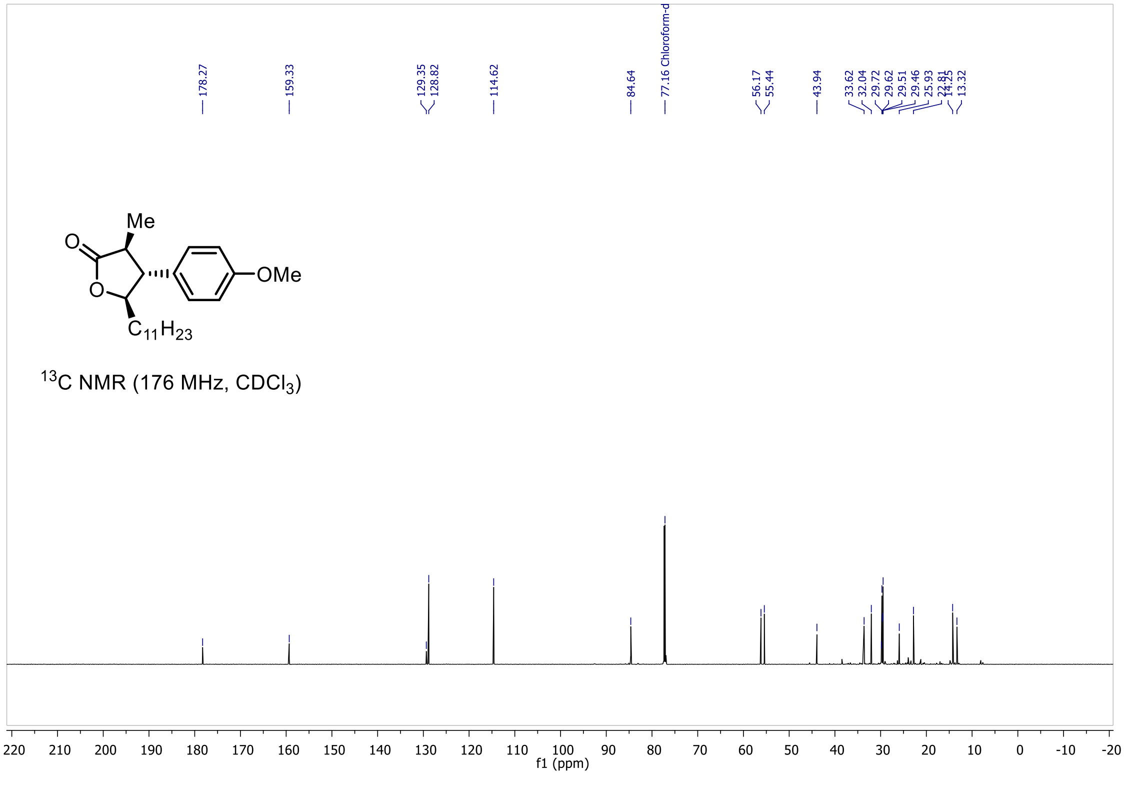

(3*S*,4*R*,5*S*)-4-(4-Methoxyphenyl)-3-methyl-5-undecyldihydrofuran-2(3*H*)-one (20b)

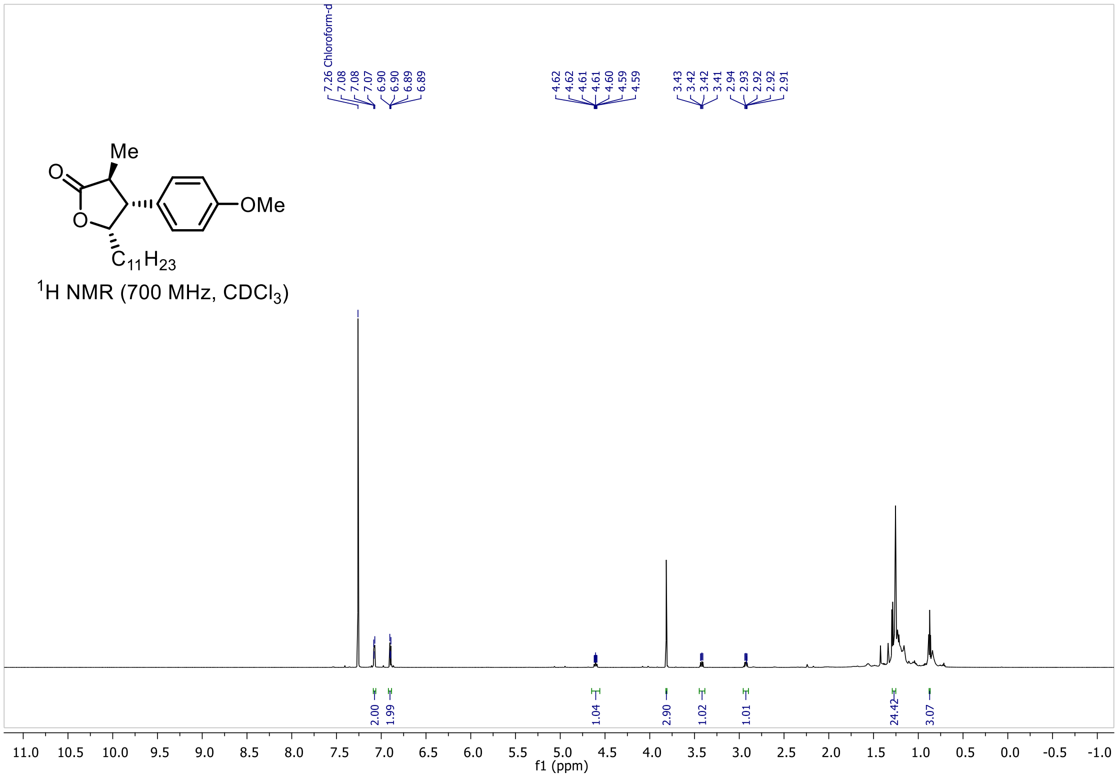

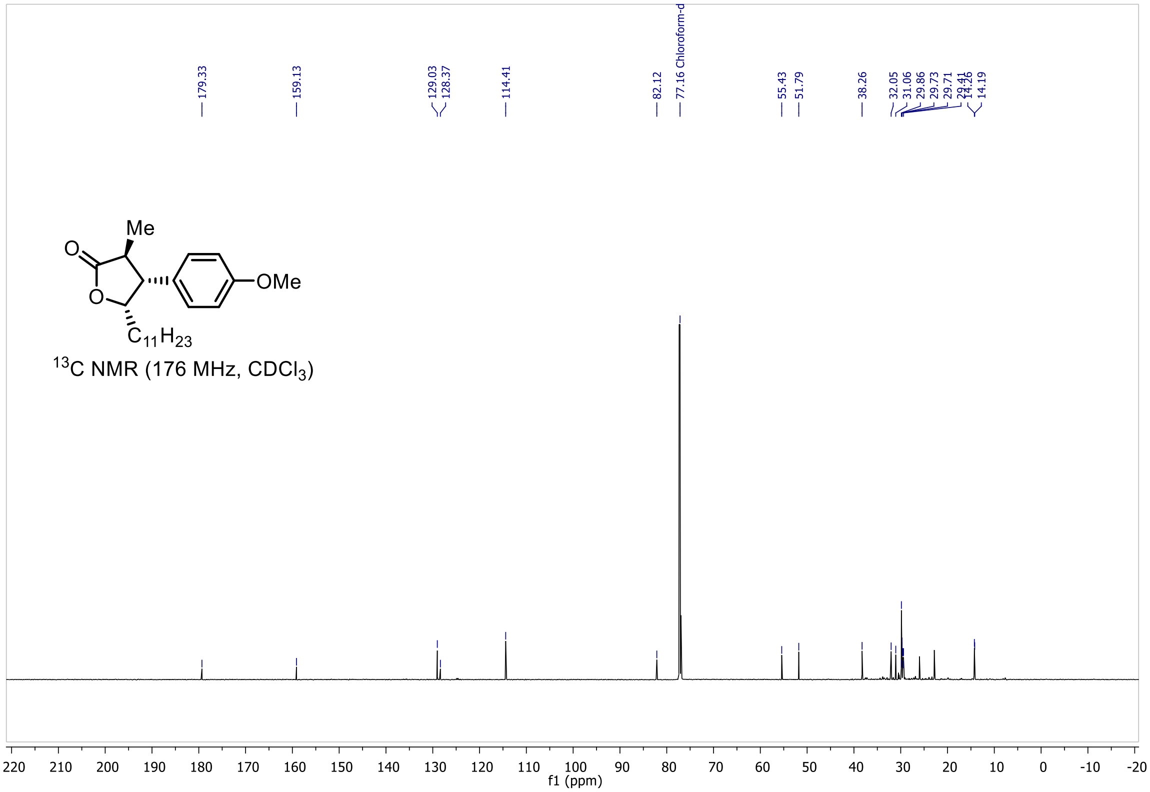

(3*S*,4*R*,5*R*)-4-(4-Methoxyphenyl)-3-methyl-5-tridecyldihydrofuran-2(3*H*)-one (21a)

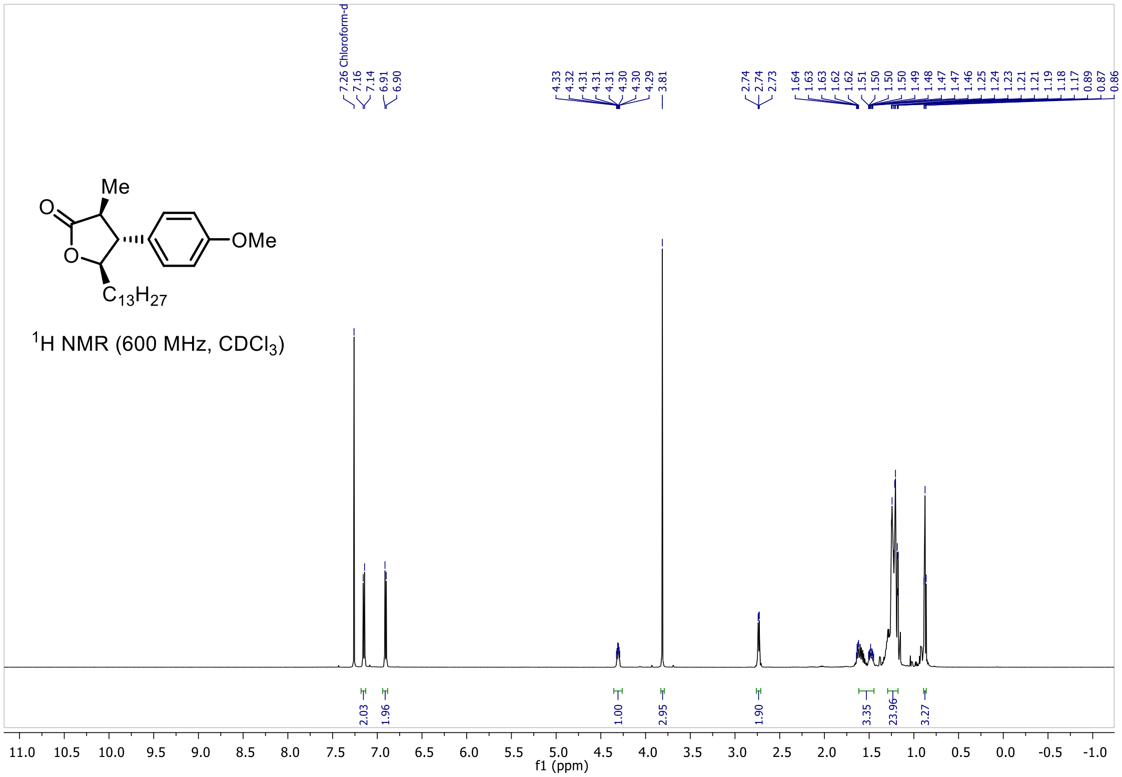

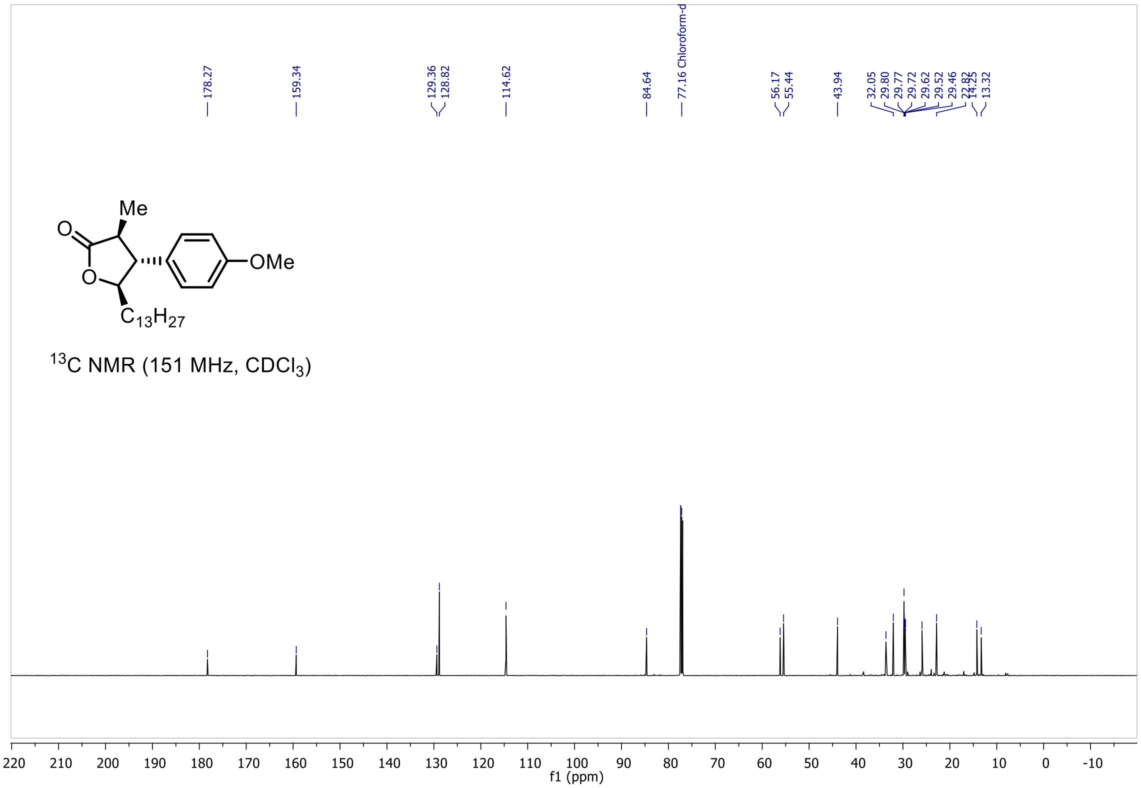

(3*S*,4*R*,5*S*)-4-(4-Methoxyphenyl)-3-methyl-5-tridecyldihydrofuran-2(3*H*)-one (21b)

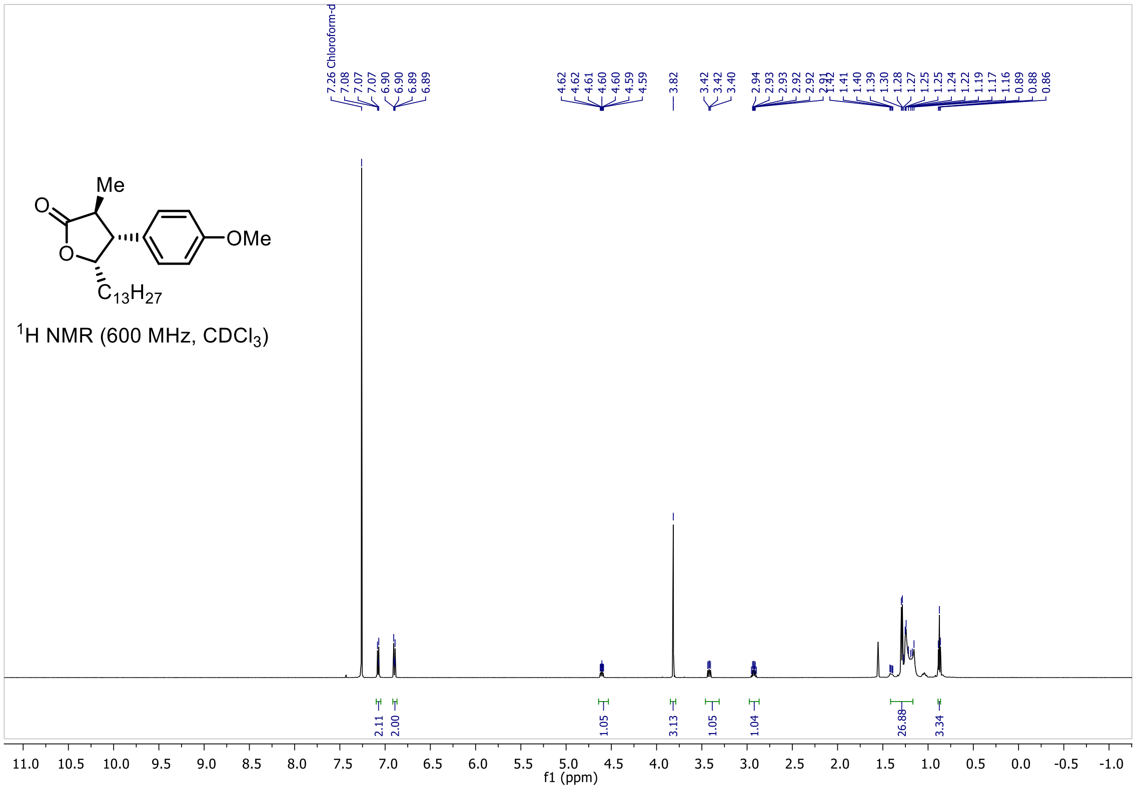

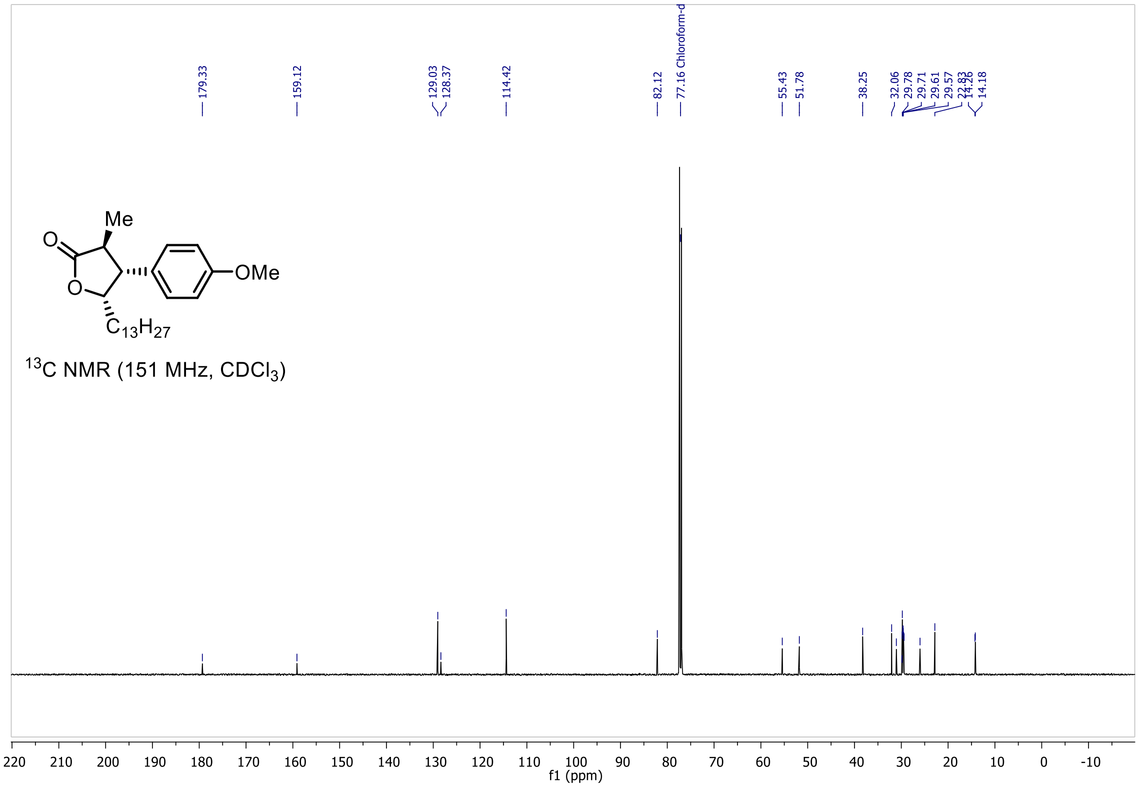

(+)-Nephrosteranic acid

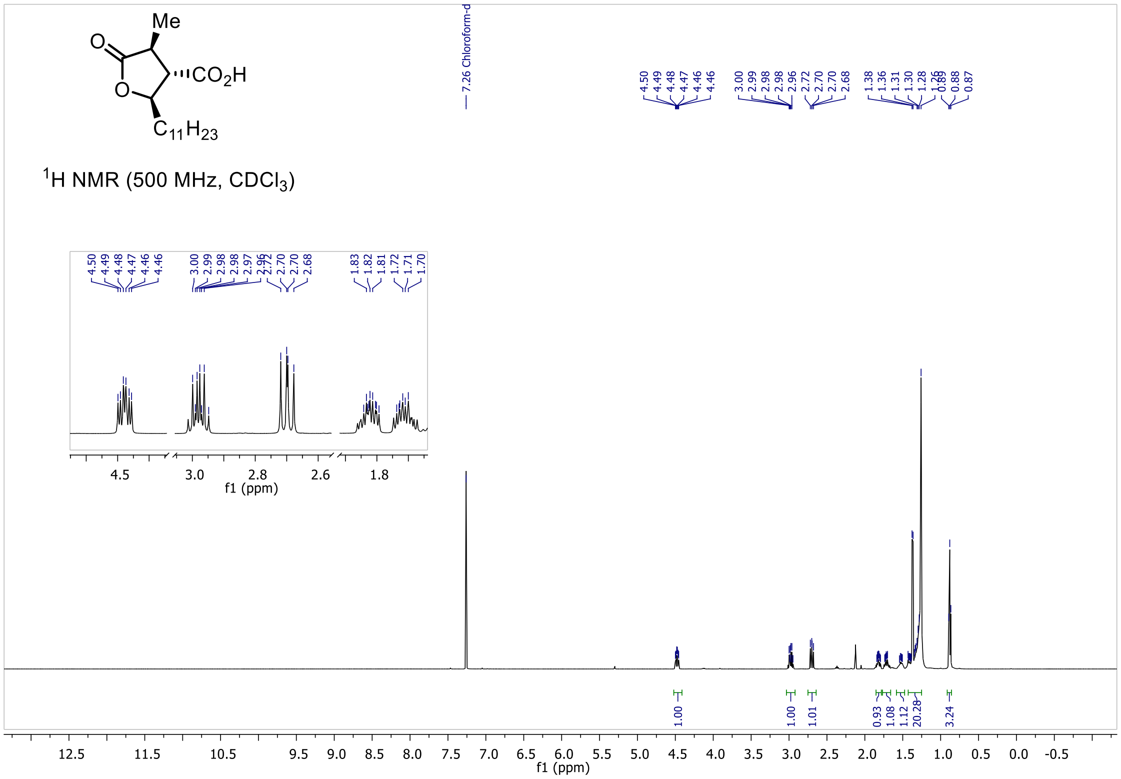

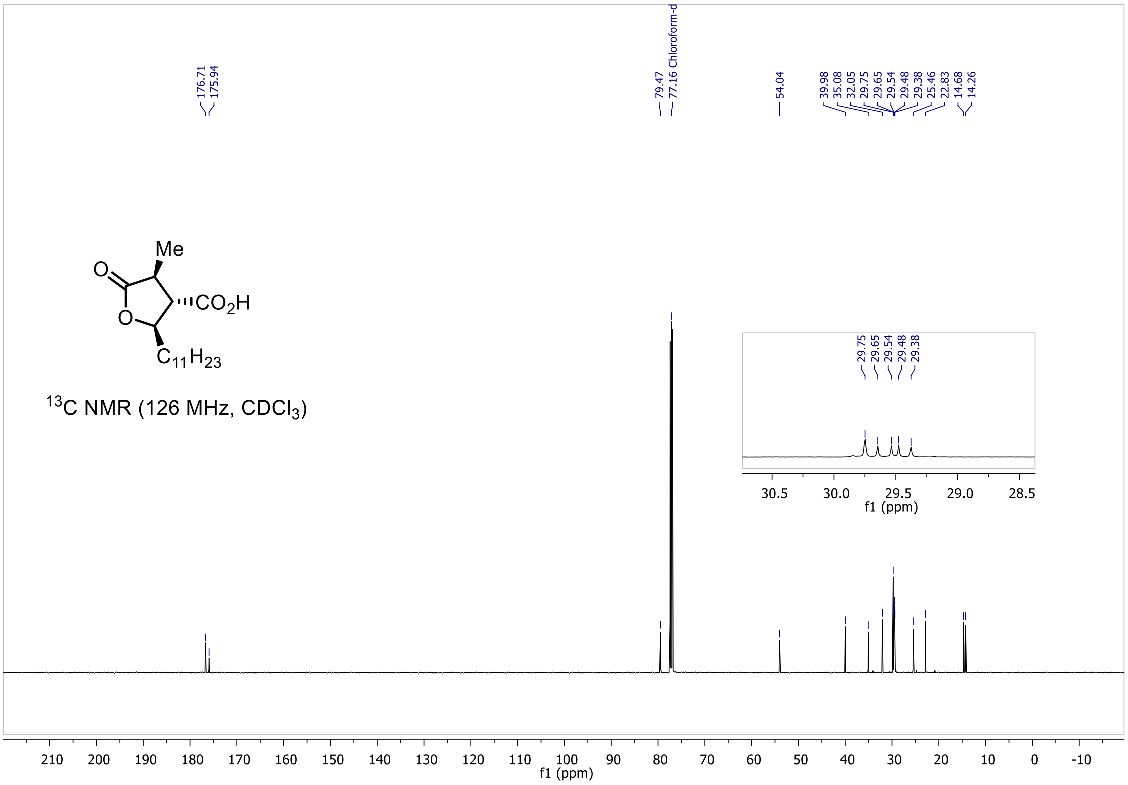

(+)-Rocellaric acid

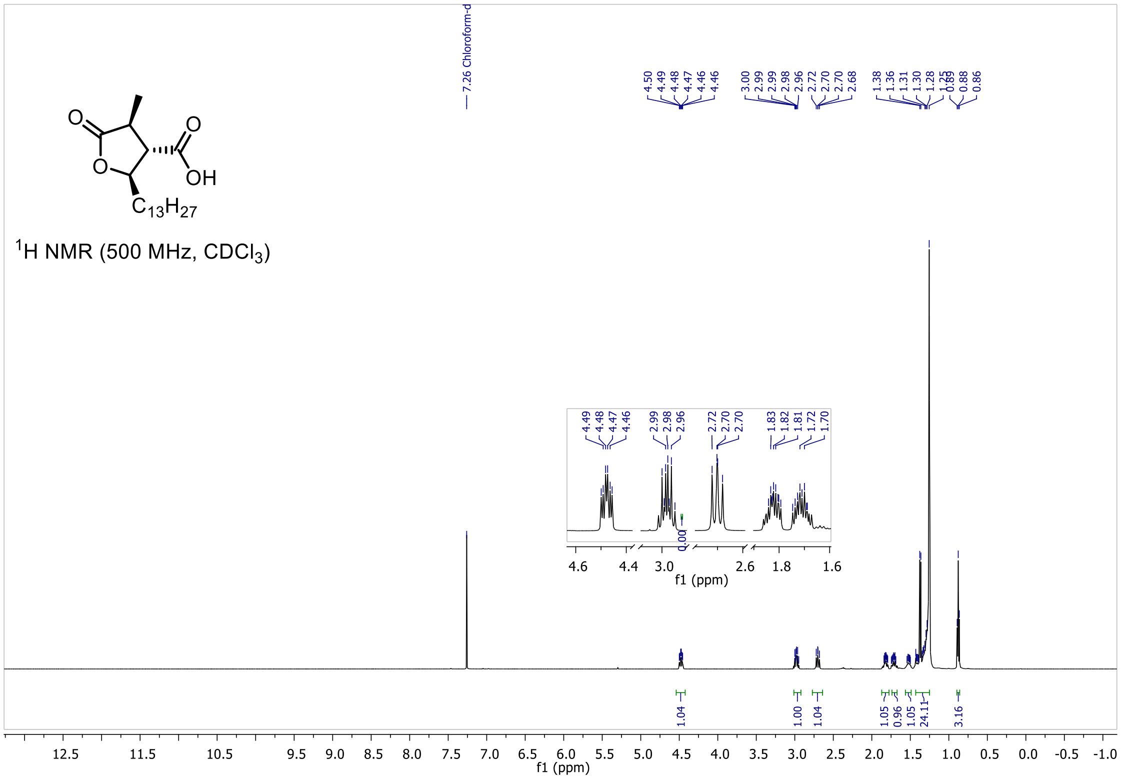

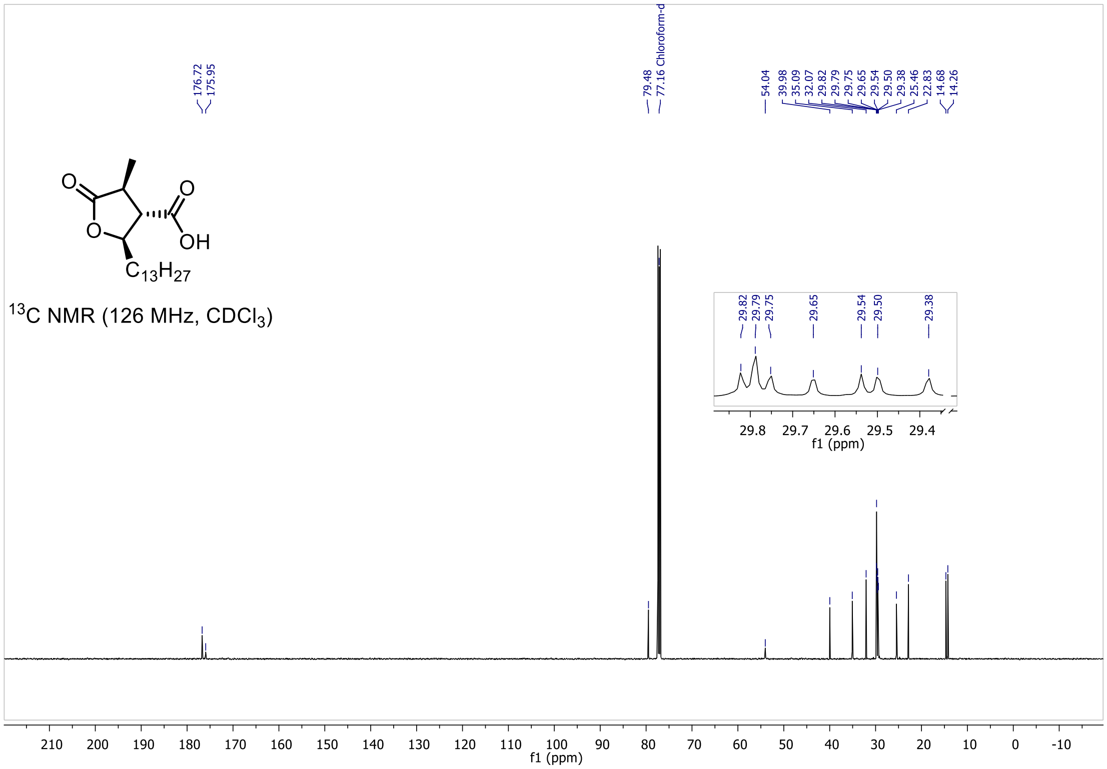

(–)-Nephromopsinic acid

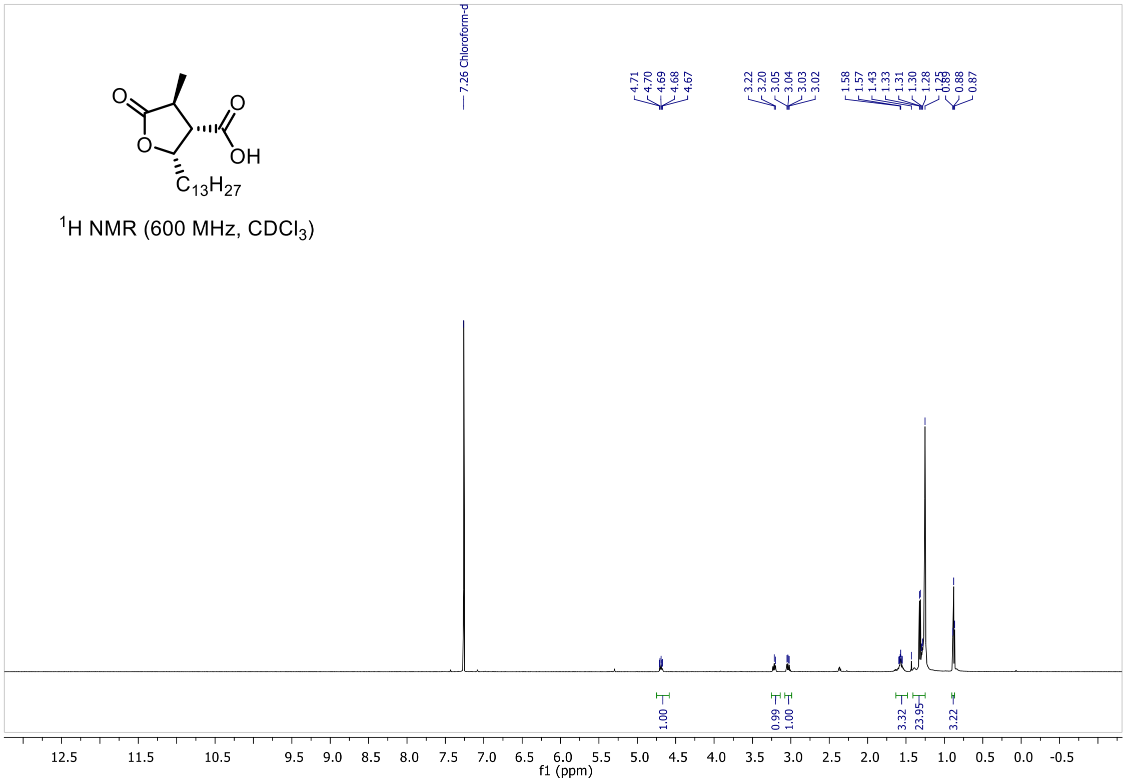

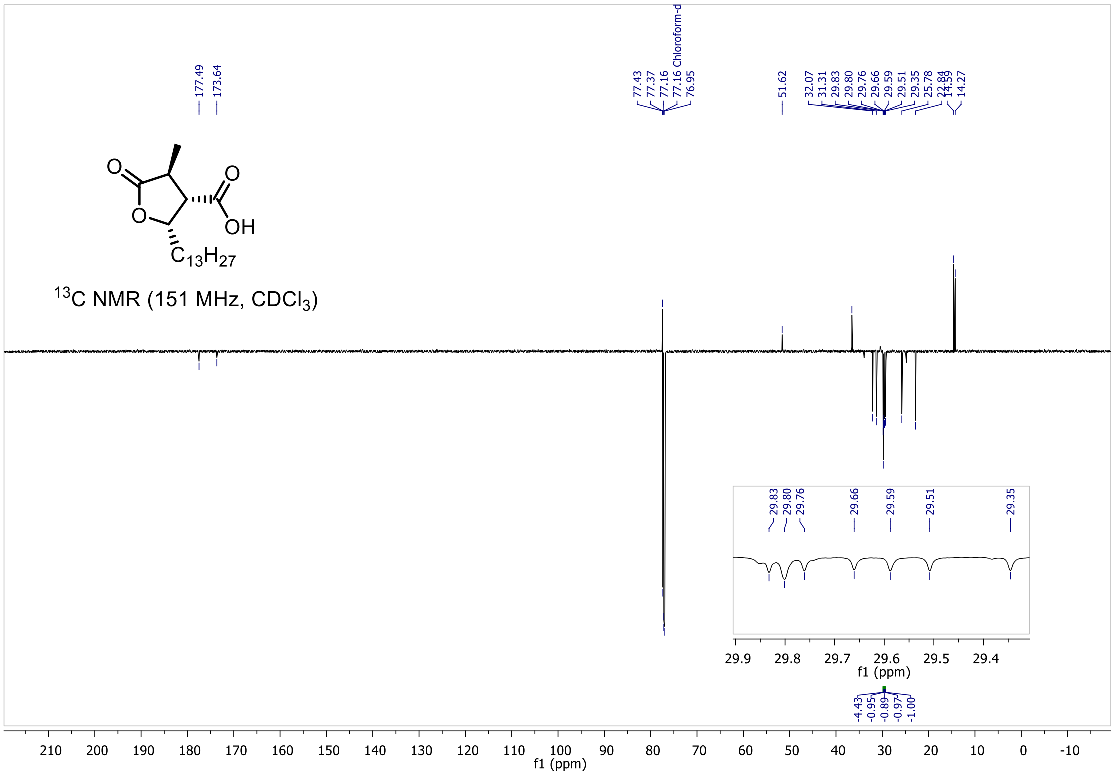

6 References

(1) Kaldre, D.; Klose, I.; Maulide, N. Stereodivergent Synthesis of 1,4-Dicarbonyls by Traceless Charge–Accelerated Sulfonium Rearrangement. *Science* **2018**, *361* (6403), 664–667.

(2) Peng, B.; Huang, X.; Xie, L.-G.; Maulide, N. A Brønsted Acid Catalyzed Redox Arylation. *Angew. Chem. Int. Ed.* **2014**, *53* (33), 8718–8721.

(3) Takahashi, T.; Li, S.; Huang, W.; Kong, F.; Nakajima, K.; Shen, B.; Ohe, T.; Kanno, K.-I. Homologation Method for Preparation of Substituted Pentacenes and Naphthacenes. *J. Org. Chem.* **2006**, *71* (21), 7967–7977.

(4) Love, B. E.; Jones, E. G. The Use of Salicylaldehyde Phenylhydrazone as an Indicator for the Titration of Organometallic Reagents. *J. Org. Chem.* **1999**, *64* (10), 3755–3756.

(5) Starkov, P.; Moore, J. T.; Duquette, D. C.; Stoltz, B. M.; Marek, I. Enantioselective Construction of Acyclic Quaternary Carbon Stereocenters: Palladium-Catalyzed Decarboxylative Allylic Alkylation of Fully Substituted Amide Enolates. *J. Am. Chem. Soc.* **2017**, *139* (28), 9615–9620.

(6) Xue, Q.; Mao, Z.; Shi, Y.; Mao, H.; Cheng, Y.; Zhu, C. Metal-Free, One-Pot Highly Selective Synthesis of (E)-Vinyl Sulfones and Sulfoxides via Addition–Oxidation of Thiols with Alkynes. *Tetrahedron Lett.* **2012**, *53* (14), 1851–1854.

(7) Mikołajczyk, M.; Perlikowska, W.; Omelańczuk, J.; Cristau, H.-J.; Perraud-Darcy, A. Stereoselective Synthesis of Racemic and Optically Active E-Vinyl and E-Dienyl Sulfoxides via Wittig Reaction of α-Sulfinyl Phosphonium Ylides. *J. Org. Chem.* **1998**, *63* (26), 9716–9722.

(8) Wang, H.; Tang, P.; Zhou, Q.; Zhang, D.; Chen, Z.; Huang, H.; Qin, Y. One-Pot Synthesis of Multisubstituted Butyrolactonimidates: Total Synthesis of (−)-Nephrosteranic Acid. *J. Org. Chem.* **2015**, *80* (5), 2494–2502.

(9) Sarkale, A. M.; Maurya, V.; Giri, S.; Appayee, C. Stereodivergent Synthesis of Chiral Paraconic Acids via Dynamic Kinetic Resolution of 3-Acylsuccinimides. *Org. Lett.* **2019**, *21* (11), 4266–4270.

(10) Becke, A. Density Functional Thermochemistry III The Role of Exact Exchange. *J. Chem. Phys.* **1993**, *98*, 5648–5652.

(11) Lee, C.; Yang, W.; Parr, R. G. Development of the Colle-Salvetti Correlation-Energy Formula into a Functional of the Electron Density. *Physical Review B* **1988**, *37* (2), 785–789.

(12) Vosko, S. H.; Wilk, L.; Nusair, M. Accurate Spin-Dependent Electron Liquid Correlation Energies for Local Spin Density Calculations: A Critical Analysis. *Canadian Journal of Physics* **1980**, *58* (8), 1200–1211.

(13) Stephens, P. J.; Devlin, F. J.; Chabalowski, C. F.; Frisch, M. J. Ab Initio Calculation of Vibrational Absorption and Circular Dichroism Spectra Using Density Functional Force Fields. *The Journal of Physical Chemistry* **1994**, *98* (45), 11623–11627.

(14) Grimme, S.; Antony, J.; Ehrlich, S.; Krieg, H. A Consistent and Accurate Ab Initio Parametrization of Density Functional Dispersion Correction (DFT-D) for the 94 Elements H-Pu. *The Journal of chemical physics* **2010**, *132* (15), 154104.

(15) Weigend, F.; Ahlrichs, R. Balanced Basis Sets of Split Valence, Triple Zeta Valence and Quadruple Zeta Valence Quality for H to Rn: Design and Assessment of Accuracy. *Phys. Chem. Chem. Phys.* **2005**, *7* (18), 3297–3305.

(16) Grimme, S.; Ehrlich, S.; Goerigk, L. Effect of the Damping Function in Dispersion Corrected Density Functional Theory. *J. Comput. Chem.* **2011**, *32* (7), 1456–1465.

(17) Pracht, P.; Bohle, F.; Grimme, S. Automated Exploration of the Low-Energy Chemical Space with Fast Quantum Chemical Methods. *Phys. Chem. Chem. Phys.* **2020**, *22* (14), 7169–7192.

(18) Grimme, S. Exploration of Chemical Compound, Conformer, and Reaction Space with Meta-Dynamics Simulations Based on Tight-Binding Quantum Chemical Calculations. *J. Chem. Theory Comput.* **2019**, *15* (5), 2847–2862.

(19) Adamo, C.; Barone, V. Toward Reliable Density Functional Methods without Adjustable Parameters: The PBE0 Model. *J. Chem. Phys.* **1999**, *110* (13), 6158–6170.

(20) Luchini, G.; Alegre-Requena, J. V; Funes-Ardoiz, I.; Paton, R. S. GoodVibes: Automated Thermochemistry for Heterogeneous Computational Chemistry Data [Version 1; Peer Review: 2 Approved with Reservations]. *F1000Research* **2020**, *9* (291), 1–14.

(21) Cancès, E.; Mennucci, B.; Tomasi, J. A New Integral Equation Formalism for the Polarizable Continuum Model: Theoretical Background and Applications to Isotropic and Anisotropic Dielectrics. *J. Chem. Phys.* **1997**, *107* (8), 3032–3041.

(22) Marenich, A. V.; Cramer, C. J.; Truhlar, D. G. Universal Solvation Model Based on Solute Electron Density and on a Continuum Model of the Solvent Defined by the Bulk Dielectric Constant and Atomic Surface Tensions. *J. Phys. Chem. B* **2009**, *113* (18), 6378–6396.

(23) Frisch, M. J.; Trucks, G. W.; Schlegel, H. B.; Scuseria, G. E.; Robb, M. A.; Cheeseman, J. R.; Scalmani, G.; Barone, V.; Mennucci, B.; Petersson, G. A.; Nakatsuji, H.; Caricato, M.; Li, X.; Hratchian, H. P.; Izmaylov, A. F.; Bloino, J.; Zheng, G.; Sonnenberg, J. L.; Hada, M.; Ehara, M.; Toyota, K.; Fukuda, R.; Hasegawa, J.; Ishida, M.; Nakajima, T.; Honda, Y.; Kitao, O.; Nakai, H.; Vreven, T.; Montgomery, J. A.; Peralta, J. E.; Ogliaro, F.; Bearpark, M.; Heyd, J. J.; Brothers, E.; Kudin, K. N.; Staroverov, V. N.; Kobayashi, R.; Normand, J.; Raghavachari, K.; Rendell, A.; Burant, J. C.; Iyengar, S. S.; Tomasi, J.; Cossi, M.; Rega, N.; Millam, J. M.; Klene, M.; Knox, J. E.; Cross, J. B.; Bakken, V.; Adamo, C.; Jaramillo, J.; Gomperts, R.; Stratmann, R. E.; Yazyev, O.; Austin, A. J.; Cammi, R.; Pomelli, C.; Ochterski, J. W.; Martin, R. L.; Morokuma, K.; Zakrzewski, V. G.; Voth, G. A.; Salvador, P.; Dannenberg, J. J.; Dapprich, S.; Daniels, A. D.; Farkas; Foresman, J. B.; Ortiz, J. V.; Cioslowski, J.; Fox, D. J. Gaussian 16, Revision A.03.

(24) Pollesböck, L. Synthesis of 1,4-Dicarbonyl via [3,3]-Sulfonium Rearrangement: Computational Investigation and Application to Heterocycle Formation, Univeristy of Vienna, 2022. DOI: 10.25365/thesis.72019.

(25) Yu, L.; Deng, Y.; Cao, J. Synthesis of Ketenaminals via Catalyst-Free Hydroamination of Ynamides and Diphenylsulfonimide. *Synthesis* **2015**, *47*, 783–788.

(26) Version 1.37.0.0 (STOE, 2021).

(27) Version 1.31.186.0 (STOE, 2022).

(28) Dolomanov, O. V.; Bourhis, L. J.; Gildea, R. J.; Howard, J. A. K.; Puschmann, H. OLEX2: A Complete Structure Solution, Refinement and Analysis Program. *Journal of Applied Crystallography* **2009**, *42* (2), 339–341.

(29) Hübschle, C. B.; Sheldrick, G. M.; Dittrich, B. ShelXle: A Qt Graphical User Interface for SHELXL. *J Appl Cryst* **2011**, *44* (6), 1281–1284.

(30) Sheldrick, G. M. (2015). SHELXS v 2016/4 University of Göttingen, Germany.

(31) Spek, A. L. Structure Validation in Chemical Crystallography. *Acta Cryst D* **2009**, *65* (2), 148–155.
